# A general strategy for C(sp^3^)–H functionalization with nucleophiles using methyl radical as a hydrogen atom abstractor

**DOI:** 10.1038/s41467-021-27165-z

**Published:** 2021-11-29

**Authors:** Isabelle Nathalie-Marie Leibler, Makeda A. Tekle-Smith, Abigail G. Doyle

**Affiliations:** 1 Department of Chemistry, Princeton University, Princeton, NJ 08544 USA; 2 Department of Chemistry and Biochemistry, University of Los Angeles, Los Angeles, CA 90095 USA

**Keywords:** Photocatalysis, Synthetic chemistry methodology

## Abstract

Photoredox catalysis has provided many approaches to C(sp^3^)–H functionalization that enable selective oxidation and C(sp^3^)–C bond formation via the intermediacy of a carbon-centered radical. While highly enabling, functionalization of the carbon-centered radical is largely mediated by electrophilic reagents. Notably, nucleophilic reagents represent an abundant and practical reagent class, motivating the interest in developing a general C(sp^3^)–H functionalization strategy with nucleophiles. Here we describe a strategy that transforms C(sp^3^)–H bonds into carbocations via sequential hydrogen atom transfer (HAT) and oxidative radical-polar crossover. The resulting carbocation is functionalized by a variety of nucleophiles—including halides, water, alcohols, thiols, an electron-rich arene, and an azide—to effect diverse bond formations. Mechanistic studies indicate that HAT is mediated by methyl radical—a previously unexplored HAT agent with differing polarity to many of those used in photoredox catalysis—enabling new site-selectivity for late-stage C(sp^3^)–H functionalization.

## Abstract (web summary)

When carbon-based units are functionalized in photoredox catalysis, electrophilic coupling partners are often used, such that the polarities of the two fragments are appropriately matched. Here the authors show a generalized methodology to instead use nucleophilic coupling partners, which are cheaper and often simpler, via successive hydrogen atom transfer and oxidative radical-polar crossover.

## Introduction

**Fig. 1:**
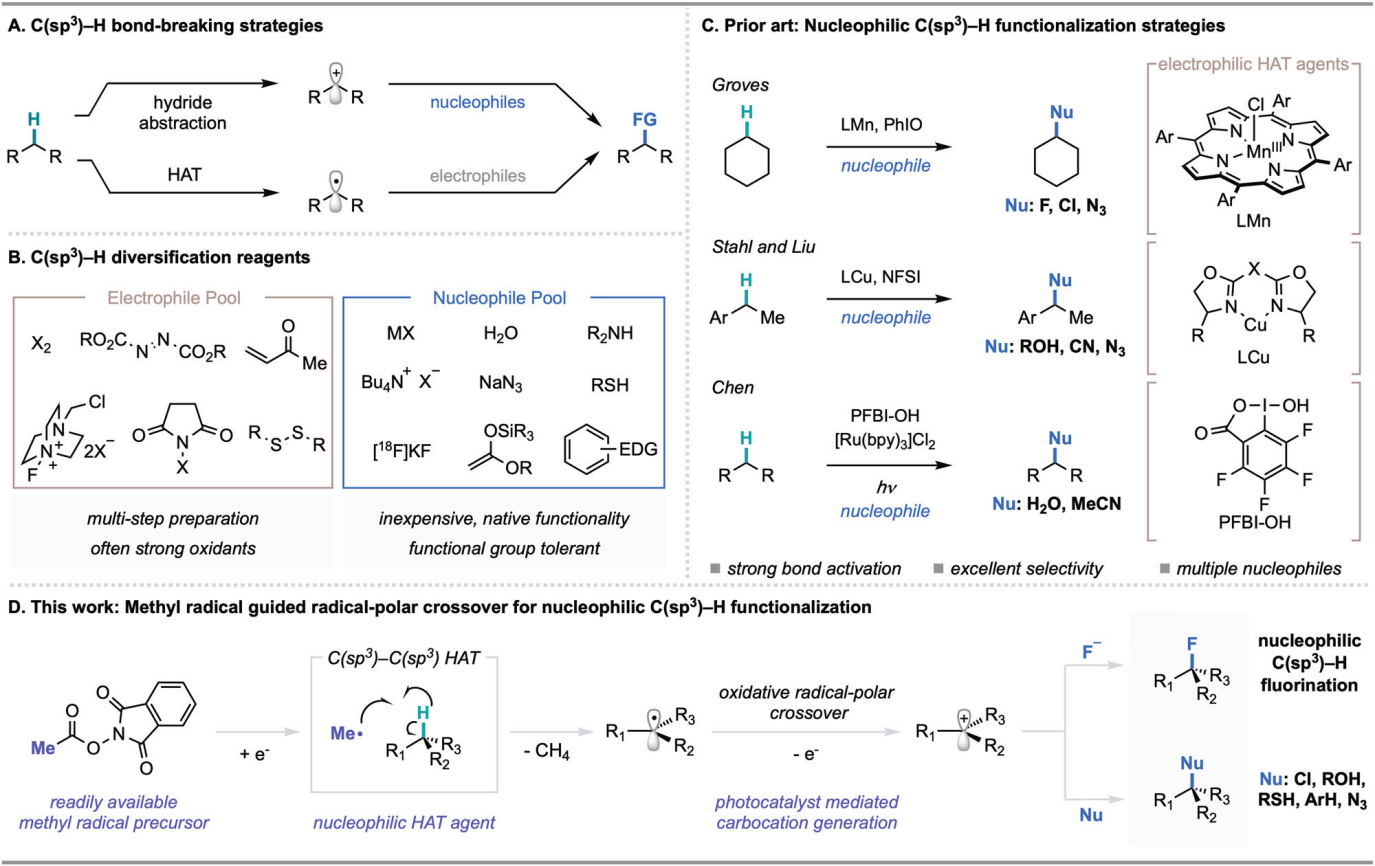
Prior art in nucleophilic C(sp^3^)–H functionalization and overview of this work. **A** Current mechanisms employed for C(sp^3^)–H activation and subsequent functionalization. **B** Array of common electrophilic and nucleophilic functionalizing reagents. **C** Recent examples of nucleophilic C(sp^3^)–H functionalization^15–17,31–34^. **D** This work. HAT=hydrogen atom transfer.

Catalytic methods for C(sp^3^)–H functionalization are of broad value for the construction of synthetic building blocks from feedstock chemicals and for the late-stage derivatization of complex molecules^1^. While significant progress has been made in this area, interfacing the cleavage of strong bonds with diverse and useful functionalization remains an outstanding challenge. Chemists have identified multiple strategies for C(sp^3^)–H bond cleavage: oxidative addition with a transition metal, concerted C(sp^3^)–H insertion, heterolytic cleavage via deprotonation or hydride abstraction, and homolytic cleavage via hydrogen atom transfer (HAT) (Fig. 1A)^2–8^. Among these tactics, hydride abstraction has seen limited development as a result of the requirement for strong Lewis acids, which are often incompatible with desirable substrates and functionalization reagents^5^. Nevertheless, access to a carbocation from a C(sp^3^)–H bond represents a valuable disconnection due to the versatility of the functionalization step, which can be general for a variety of heteroatom and carbon-centered nucleophiles in their native state.

In contrast to hydride abstraction, HAT can offer a mild and versatile approach to C(sp^3^)–H cleavage through the conversion of C(sp^3^)–H bonds to radical intermediates^7,9^. While strategies for the homolytic cleavage of C(sp^3^)–H bonds have been highly enabling, radical functionalization in these methodologies is dominated by electrophilic reagents (e.g., Selectfluor^TM^ for fluorination, peroxides for alkoxylation, azodicarboxylates for amination, and electron-deficient arenes for C–C bond formation) (Fig. 1B)^10–12^. Electrophilic reagents are often strong oxidants, expensive to purchase, or require multi-step synthesis, posing limitations to their use in certain contexts^12,13^. Nucleophilic reagents represent an abundant and practical reagent class, and offer an opportunity to access functional group compatibility complementary to that provided by electrophilic reagents. However, productively engaging a nucleophilic carbon-centered radical with a nucleophilic functionalizing reagent presents an inherent challenge due to polarity matching^3,6,14–17^.

Recently, we disclosed a photocatalytic strategy for the decarboxylative nucleophilic fluorination of redox-active esters^18^. This methodology leveraged *N*-acyloxyphthalimides as alkyl radical precursors and an oxidative radical-polar crossover (ORPC) mechanism for the generation of a carbocation poised for nucleophilic addition^19^. Seeking to develop a modular nucleophilic C(sp^3^)–H functionalization, we questioned whether photocatalytic ORPC could be combined with principles of HAT to achieve formal hydride abstraction from C(sp^3^)–H bonds. Given the versatility of carbocation intermediates, such a reaction platform could provide a general route to numerous desirable transformations such as C(sp^3^)–H halogenation, hydroxylation, and C–C bond formation by combining two abundant and structurally diverse feedstocks.

C(sp^3^)–H functionalization via HAT-ORPC has been proposed as a possible mechanism in several important studies^6,15,20–23^. For example, Chen and coworkers have proposed this mechanistic pathway in the context of C(sp^3^)–H hydroxylation and amidation with hypervalent iodine, and computational investigations from Stahl, Liu, and coworkers have supported a HAT-ORPC pathway for copper-catalyzed azidation and etherification reactions (Fig. 1C). While access to carbocation intermediates from C(sp^3^)–H bonds may also be accomplished electrochemically, contemporary methodologies are largely limited by the high overpotential required for reactivity^24,25^. Alternatively, recent contributions to radical-based C(sp^3^)–H functionalization with nucleophiles have centered on the use of a transition-metal catalyst to mediate radical capture and subsequent bond formation, rendering a nucleophile an electrophilic ligand in the presence of a stoichiometric oxidant. Stahl, Liu, and coworkers have demonstrated the utility of a copper/NFSI/nucleophile platform for radical-relay in a variety of C(sp^3^)–H functionalization methods (Fig. 1C)^15–17,26–30^. In addition, seminal work from Groves and coworkers has provided strategies for nucleophilic C(sp^3^)–H halogenation and azidation using a bioinspired Mn porphyrin catalyst (Fig. 1C)^31–34^. Zhang and coworkers have also developed a fluorination of C(sp^3^)–H bonds using a Cu^III^ fluoride complex generated in situ from fluoride^35^. While all highly enabling, the requirement for strong or super-stoichiometric oxidants in these methods can limit their application in synthesis and generality across diverse nucleophile coupling partners; functionality such as electron-rich arenes, alkenes, and thiols are susceptible to oxidation by oxidants such as iodosyl benzene and Selectfluor^TM^^36–38^. Moreover, the prior art in nucleophilic C(sp^3^)–H functionalization relies on electrophilic HAT agents, which are polarity-matched to select for hydridic C(sp^3^)–H bonds. The identification of mechanistically distinct strategies that permit mild conditions and enable distinct site- and chemoselectivity could advance the scope and practicality of C(sp^3^)–H functionalization methods with nucleophilic coupling partners in chemical synthesis.

Our initial investigations focused on C(sp^3^)–H fluorination, a valuable transformation in organic synthesis due to the unique chemical properties conferred by fluorine substitution^39–41^. In recent years, a number of electrophilic C(sp^3^)–H fluorination strategies have proven highly enabling^12,40^. However, few reports detailing C(sp^3^)–H fluorination with fluoride have been disclosed, due not only to the broad challenges posed by C(sp^3^)–H activation, but also the attenuated nucleophilicity of fluoride^22,31,35,42–44^. Despite these challenges, the development of nucleophilic C(sp^3^)–H fluorination methods is desirable given the low cost of fluoride sources and their application to radiofluorination for positron emission tomography (PET) imaging^40^.

Here we report a HAT-ORPC platform for C(sp^3^)–H functionalization using mild and commercially available *N*-acyloxyphthalimide—a methyl radical precursor—as the HAT reagent. The platform enables C(sp^3^)–H fluorination of secondary and tertiary benzylic and allylic substrates using Et_3_N•3HF. In addition, we demonstrate the versatility of the reaction to achieve C(sp^3^)–H chlorination, hydroxylation, etherification, thioetherification, azidation, and carbon–carbon bond formation.

## Results

### Reaction optimization

To evaluate the feasibility of the HAT-ORPC strategy for C(sp^3^)–H fluorination, we investigated the conversion of diphenylmethane to fluorodiphenylmethane (**2**) using a variety of phthalimide-derived HAT precursors (Table 1). We focused on *N*-acyloxyphthalimides and *N*-alkoxyphthalimides, as these redox-active species deliver a radical HAT agent via reductive fragmentation, leaving an oxidized photocatalyst available to execute ORPC; furthermore, these reagents are easy to prepare and tune, and are less oxidizing than the stoichiometric oxidants used in radical relay strategies^45^. Optimization of the HAT precursor focused on three design elements: (**1**) redox compatibility, (**2**) bond dissociation energy (BDE) of the radical generated upon fragmentation (favorable thermodynamics), and (**3**) nucleophilicity of the HAT byproduct (competitive carbocation functionalization). We were pleased to find that using Ir(*p*-F-ppy)_3_ as a photocatalyst, Et_3_N•3HF as a fluoride source, and HAT abstractor **3** (MeO–H BDE = 105 kcal/mol) in pivalonitrile afforded alkyl fluoride **2** in 45% yield (Table 1, entry **1**)^46^. In addition to desired fluoride **2**, we observed generation of the corresponding benzhydryl methyl ether in 7% yield, resulting from competitive trapping of the carbocation with methanol.

**Table 1:**
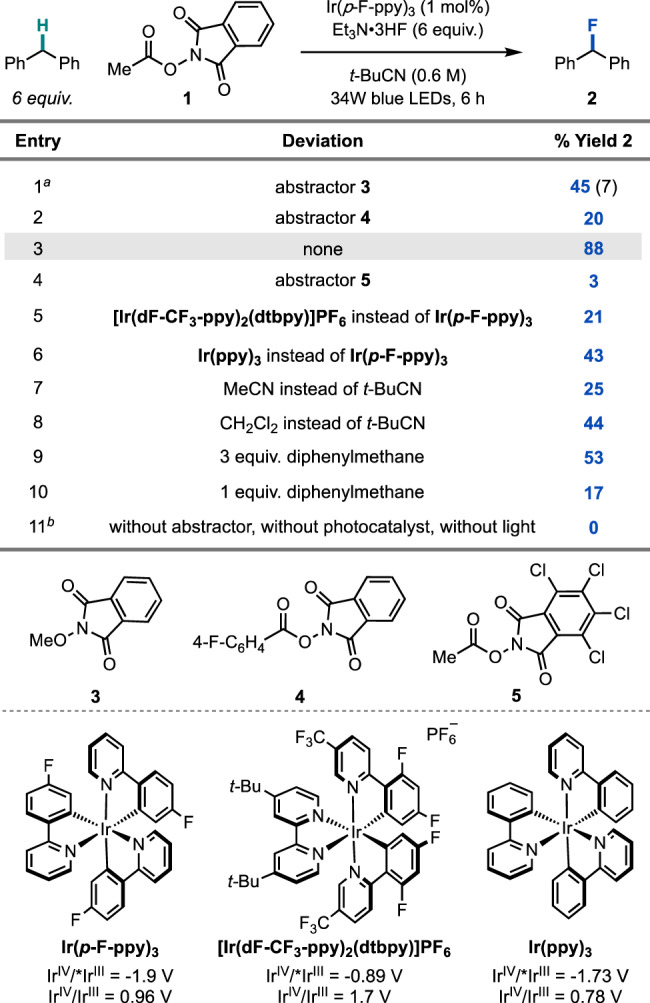
Reaction optimization.

Reactions performed on 0.15 mmol scale with 1-fluoronaphthalene added as an external standard (^19^F NMR yield). *t*-BuCN = pivalonitrile. All potentials given are versus a saturated calomel electrode (SCE) and taken from ref. ^52^. ^*a*^Parentheses indicate yield of the benzhydryl methyl ether product (^1^H NMR yield). ^*b*^Each control reaction was completed independently in the absence of key reaction components.

Moreover, analysis of the reaction mixture indicated poor conversion of **3**, possibly arising from inefficient single-electron reduction and fragmentation of the *N*-alkoxyphthalimide (*E*_1/2_^red^ ∼ −1.42 V vs. SCE)^47^.

These observations prompted us to evaluate *N*-acyloxyphthalimide **4** (*E*_1/2_^red^ ∼ −1.2–1.3 V vs. SCE), a benzoyloxy radical precursor^47^. Upon HAT, this radical generates benzoic acid, a less nucleophilic byproduct than methanol. However, **4** did not improve reaction yield (Table 1, entry **2**), likely due to competitive generation of the insufficiently reactive phthalimide radical upon SET and fragmentation (phthalimide N–H BDE = 89.1 kcal/mol vs. benzoic acid O–H BDE = 111 kcal/mol)^48^. Instead, we found that *N*-acyloxyphthalimide **1**—a methyl radical precursor—was the most effective HAT reagent, delivering the desired fluoride **2** in 88% yield (Table 1, entry **3**). Abstractor **1** is likely effective because there is a strong thermodynamic and entropic driving force associated with formation of methane (BDE = 105 kcal/mol), an inert, non-nucleophilic byproduct^46,49^. Notably, **1** is commercially available and can also be prepared on multi-decagram scale in one step from low-cost, readily available materials^50^. Tetrachlorophthalimide analogue **5** was also investigated, but the poor solubility of **5** led to trace conversion (Table 1, entry **4**)^51^. With **1**, Ir(*p*-F-ppy)_3_ was the optimal photocatalyst for this transformation, presumably because Ir(*p*-F-ppy)_3_ allows for both the reductive generation of methyl radical (*E*_1/2_ Ir^IV^/*Ir^III^ = −1.96 V vs. SCE for Ir(*p*-F-ppy)_3_ and *E*_1/2_^red^ = −1.24 V vs. SCE for **1**) and the oxidation of diphenylmethyl radical (*E*_1/2_ Ir^IV^/Ir^III^ = 0.96 V vs. SCE and *E*_1/2_^ox^ = 0.35 V vs. SCE for 2° benzylic)^47,52,53^. Use of either less reducing or less oxidizing photocatalysts resulted in diminished yields (Table 1, entries **5**–**6**). While highest yields were observed with 6 equivalents of the C(sp^3^)–H partner, 3 equivalents and 1 equivalent of the substrate could also be used, albeit with diminished reactivity (53% and 17% yield respectively) (Table 1, entry **9**–**10**). Finally, control reactions indicate that HAT reagent **1**, photocatalyst, and light are all necessary for reactivity (Table 1, entry **11**).

### Substrate scope

**Fig. 2:**
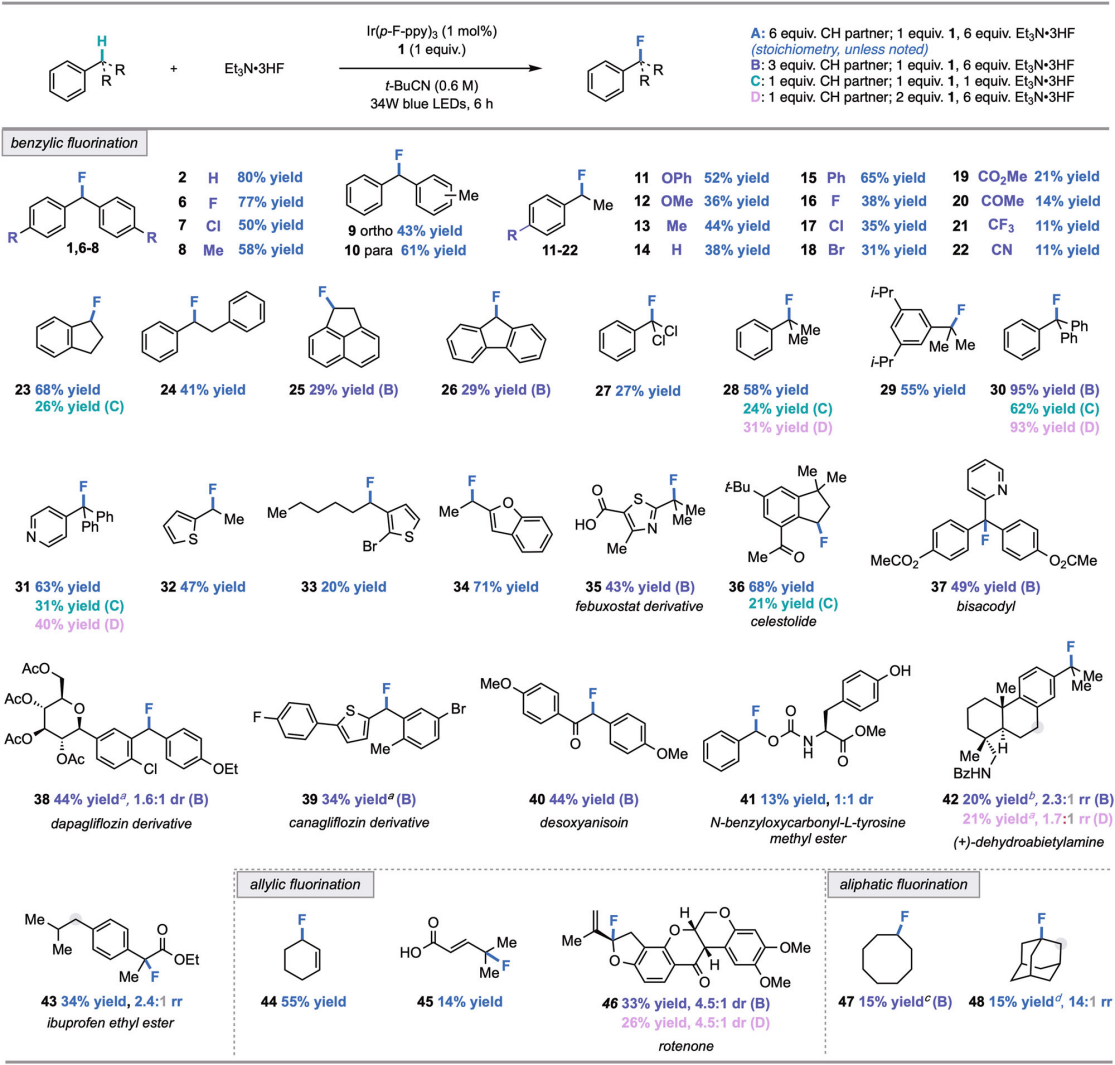
Scope of C(sp^3^)–H fluorination (0.25 mmol scale, ^19^F NMR yields). ^*a*^Reaction performed using Ir(*p*-CF_3_-ppy)_3_ as photocatalyst and benzene as solvent. ^*b*^Reaction performed using Ir(*p*-CF_3_-ppy)_3_ as photocatalyst and 1,2-difluorobenzene as solvent. ^*c*^Reaction performed with 20 mol % *n*-Bu_4_NPF_6_. ^*d*^Reaction performed using Ir(*p*-CF_3_-ppy)_3_ as photocatalyst, 1,2-difluorobenzene as solvent, and abstractor **3**.

With optimized conditions established, we set out to examine the scope of C(sp^3^)–H fluorination (Fig. 2). A broad range of functionality was tolerated, including halogen (**16**–**18**, **33**, **39**), ether (**11** and **12**), carboxylic acid (**35** and **45**), nitrile (**22**), and trifluoromethyl (**21**) substituents, as well as heterocycles (**31**–**35**, **37**, **39**), a protected amine (**42**), and a phenol (**41**). Electron-rich functionality, vulnerable to electrophilic reagents or stoichiometric oxidants, was also well tolerated (**11**, **38**, and **46**) (*vida infra*)^54,55^. Notably, tertiary benzylic C(sp^3^)–H partners underwent functionalization to generate fluorinated products often inaccessible via nucleophilic fluorination due to slow substitution and competitive elimination (**28, 29**, **30**, **31**, **35**, **37**, **42**, **and 43**)^56^. We also discovered that fluorination can be achieved with 1 equivalent each of C(sp^3^)–H coupling partner and Et_3_N•3HF (**23**, **28**, **30**, **31**, **36**, **42** and **46**). Of these examples, yields for tertiary C(sp^3^)–H coupling partners improved upon adjusting stoichiometry to a 2:1 ratio of HAT precursor **1**: substrate. We reason that excess **1** is advantageous in the case of tertiary substrates as the resulting product will not competitively consume methyl radical.

Through an exploration of late-stage derivatization, fluorination of a derivative of dapagliflozin—a medication for the treatment of type 2 diabetes—afforded **38** in 44% yield, demonstrating the compatibility of a complex, electron-rich C–glycoside with these conditions. Furthermore, given the significance of α-fluorocarbonyl motifs in medicinal chemistry, we also evaluated the fluorination of the immunosuppressant deoxyanisoin and a derivative of the anti-inflammatory ibuprofen, delivering **40** and **43** in 44% yield and 34% yield, respectively^39^. Interestingly, in the fluorination of both ibuprofen ethyl ester and the *N*-benzoyl derivative of anti-tumor agent (+)-dehydroabietylamine (**42**), site-selectivity for C(sp^3^)–H fluorination at tertiary rather than secondary sites was observed, a notable reversal in site-selectivity from prior studies demonstrating the functionalization of these targets^6,15,17,44^. Gratifyingly, the mild conditions of this methodology allowed the recovery of unreacted C(sp^3^)–H coupling partner unaltered from product mixtures.

Nucleophilic fluorination could also be extended to allylic C(sp^3^)–H coupling partners. Allylic fluorides are valuable motifs in medicinal chemistry and are useful building blocks in synthesis^57^. The development of allylic C(sp^3^)–H fluorination methods has proven challenging, as most electrophilic reagents and stoichiometric oxidants utilized in fluorination methodologies favor olefin oxidation over C(sp^3^)–H functionalization; alternatively, most sources of fluoride facilitate competitive elimination (See Supplementary Information for details)^35,43,58,59^. As an illustration of the mildness of a HAT-ORPC strategy, the fluorination of cyclohexene proceeded in 55% yield (**44**), a significant improvement to our prior efforts in the allylic C(sp^3^)–H fluorination of this substrate using a Pd/Cr cocatalyst system^43^. Furthermore, the fluorination of 4-methyl-2-pentenoic acid and the pesticide rotenone occurred in 14% and 33% yield, respectively (**45** and **46**). Finally, to explore the boundaries of reactivity with this HAT-ORPC approach, we examined unactivated C(sp^3^)–H scaffolds, as these substrates tend to possess higher BDEs and oxidation potentials in comparison to benzylic or allylic systems. Broadly, this substrate class demonstrated attenuated reactivity; for example, cyclooctane and adamantane underwent fluorination to deliver **47** and **48** in low yield.

In theory, synthetic methods that employ nucleophilic C(sp^3^)–H fluorination strategies can provide complementary functional group tolerance to their electrophilic counterparts. To demonstrate the synthetic opportunities afforded by this nucleophilic C(sp^3^)–H fluorination strategy that makes use of a mild oxidant (**1**), we performed a series of head-to-head comparisons with electrophilic fluorinating methods that use Selectfluor^TM^ or NFSI in order to examine the compatibility of electron-rich functionality (see Supplementary Information, Section VIII). We subjected three particularly electron-rich substrates from our scope studies—specifically, rotenone, a dapagliflozin derivative, and *p*-OPh ethylbenzene—to state of the art electrophilic fluorination conditions with Selectfluor^TM^, and observed little to no fluorination in all cases in addition to the generation of several degradation side products. Upon reaction with NFSI—a milder reagent than Selectfluor^TM^—we observed that *p*-OPh ethylbenzene was tolerated, affording product **11** in 76% yield. However, no fluorination was observed in the attempted syntheses of **46** and **38**. Further details on these experiments are provided in the Supplementary Information. Taken together, these studies demonstrate that this method offers complementarity to alternative strategies for C(sp^3^)–H fluorination with respect to scope and site-selectivity.

**Fig. 3:**
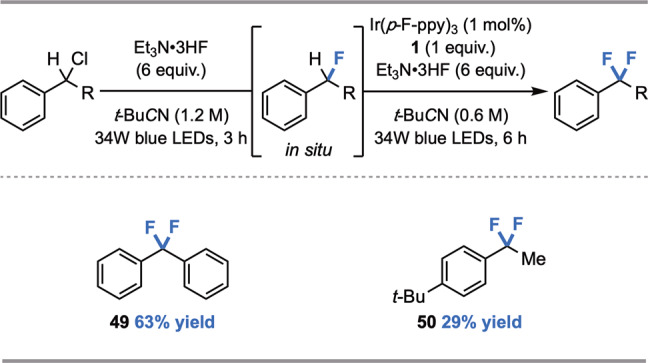
Scope of C(sp^3^)–H difluorination (0.25 mmol scale, ^19^F NMR yield). See Supplementary Information for reaction details.

Notably, difunctionalization is not observed to an appreciable extent in the fluorination of ArCH_2_R precursors, even though HAT with the monofluorinated product is favorable on account of weaker BDFEs and polarity matching (methyl radical is mildly nucleophilic). We hypothesize that monofluorination selectivity results from the relative stoichiometry of starting material and abstractor, which likely serves to mitigate unproductive side-reactivity involving methyl radical (See Supplementary Information, Section II, Part C). To explore this hypothesis, we envisioned that benzylic fluorides generated in situ from their monochlorinated precursors could deliver difluorinated products under optimized C(sp^3^)–H fluorination conditions. Difluorinated products **44** and **45** were obtained in 63% and 29% yield, respectively from the corresponding benzyl chlorides (Fig. 3). Furthermore, the results of this investigation suggest that HAT-ORPC from monofluorinated C(sp^3^)–H centers is less efficient than from the non-fluorinated C(sp^3^)–H starting materials (See Supplementary Information), likely arising from a less favorable radical oxidation step at an electronically deficient site. To our knowledge, this represents the first nucleophilic C(sp^3^)–H fluorination to achieve difluorinated motifs, units which have emerged as important lipophilic bioisosteres of hydroxyl and thiol functional groups in drug design^60^.

**Fig. 4:**
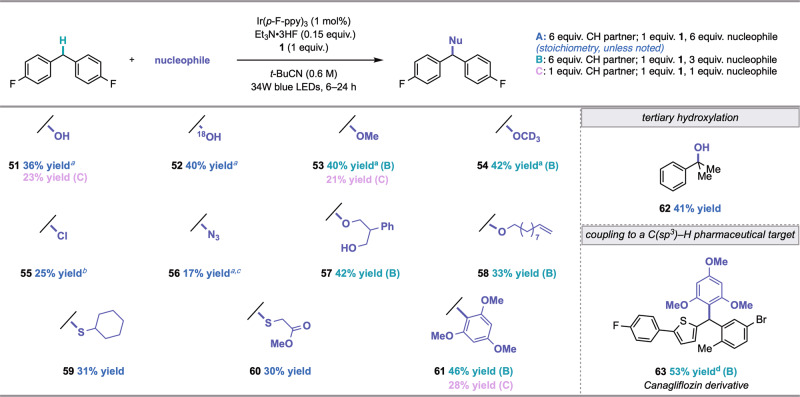
Scope of general nucleophilic C(sp^3^)–H functionalization (0.25 mmol, isolated yields). ^*a*^^19^F NMR yields. ^*b*^Reaction was performed without Et_3_N•3HF. ^*c*^Reaction was performed without Et_3_N•3HF and with 0.15 equiv. H_2_O. ^*d*^Reaction performed using Ir(*p*-CF_3_-ppy)_3_ as photocatalyst, benzene as solvent, and 3.0 equiv. C(sp^3^)–H coupling partner.

Next, we evaluated whether this strategy could serve as a platform for C(sp^3^)–H functionalization with other nucleophiles (Fig. 4). Indeed, we were pleased to find that only minor adjustments to the standard fluorination conditions were needed to accommodate nucleophiles other than Et_3_N•3HF (see Mechanistic Investigations for discussion on the role of Et_3_N•3HF, *vide infra*). Irradiation of 4,4′-difluorodiphenylmethane with 1 mol % Ir(*p*-F-ppy)_3_, 15 mol % Et_3_N•3HF, HAT precursor **1**, and 6 equivalents of water in pivalonitrile afforded benzhydryl alcohol **51** in 36% yield. Hydroxylation took place with no evidence of overoxidation to the ketone in the synthesis of both **51** and **52**, a common limitation of many C(sp^3^)–H oxidation methods^61^. These conditions were also amenable to the hydroxylation of a tertiary C(sp^3^)–H substrate (**62**). Furthermore, nucleophiles such as methanol and methanol*-d*_4_ afforded methyl ether products **53** and **54** in 40% and 42% yield, respectively. More complex oxygen-centered nucleophiles, including a 1,3-diol and dec-9-en-1-ol, were also compatible (**57** and **58**). Furthermore, we were pleased to accomplish the installation of a C(sp^3^)–Cl bond using HCl•Et_2_O as a nucleophile (**55**), and to discover that C(sp^3^)–N bond formation could be achieved through cross coupling with azidotrimethylsilane (**56**). The construction of medicinally valuable thioethers was also possible, using cyclohexanethiol (**59**) and methylthioglycolate (**60**) as sulfur-based nucleophiles. In particular, the implementation of sulfur nucleophiles highlights the mildness of reaction conditions, as thiol oxidation could otherwise interfere with C(sp^3^)–S bond formation under alternative C(sp^3^)–H functionalization approaches. Carbon–carbon bond formation via a mild, direct Friedel-Crafts alkylation was also accomplished in 41% yield from the coupling of 1,3,5-trimethoxybenzene and 4,4′-difluorodiphenylmethane (**61**). Friedel-Crafts reactions typically require pre-oxidized substrates—such as alkyl halides—and Lewis or Brønsted acid conditions that are often incompatible with the desired nucleophiles^62^. Gratifyingly, functionalization may also be achieved with 1 equivalent of C(sp^3^)–H coupling partner and 1 equivalent of nucleophile (**51**, **53**, and **61**). Finally, the late-stage derivatization of pharmaceutical targets was demonstrated in the Friedel-Crafts cross-coupling between the anti-diabetic drug canagliflozin precursor and 1,3,5-trimethoxybenzene to deliver **63** in 53% yield.

### Mechanistic studies

**Fig. 5:**
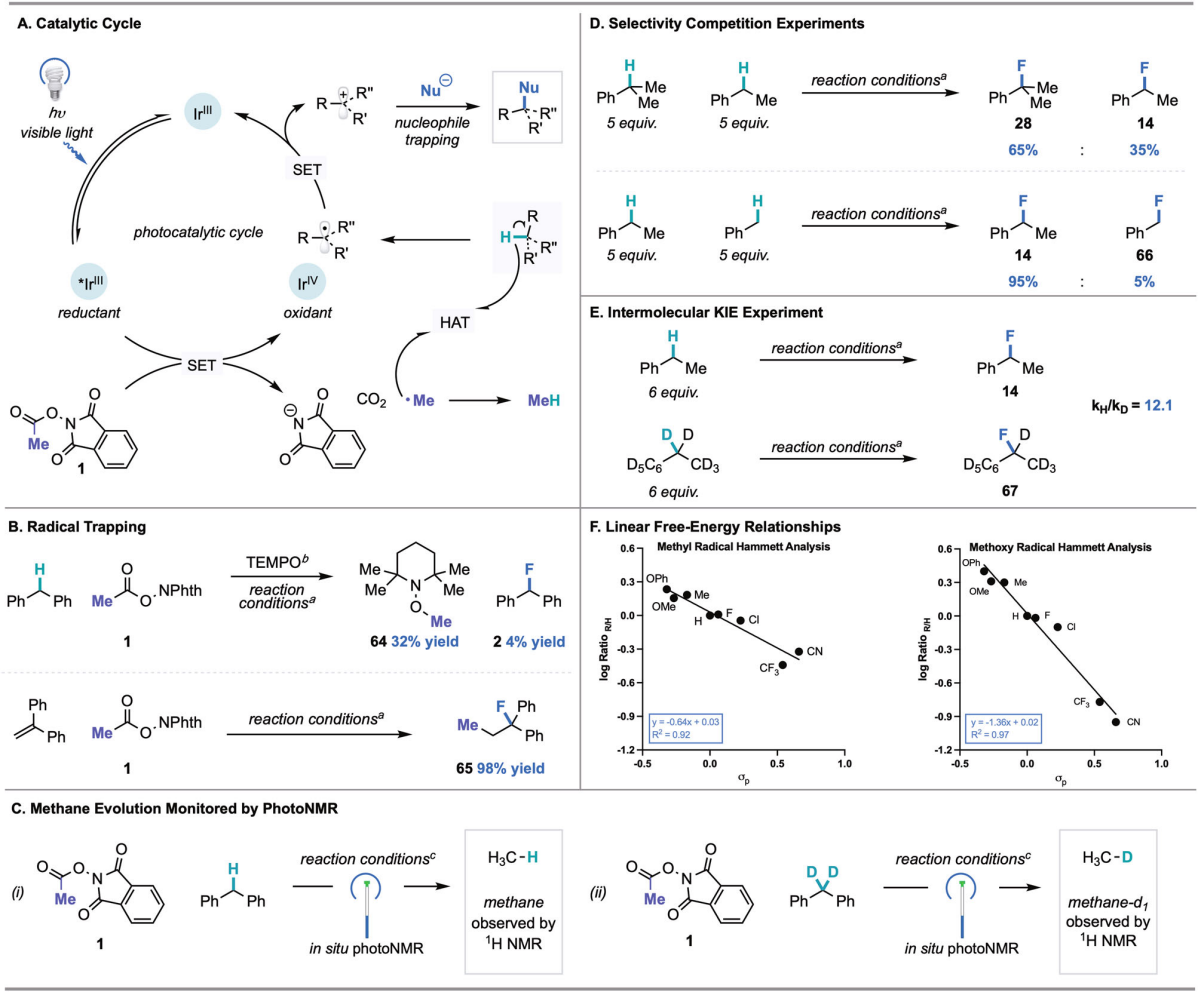
Mechanistic investigations of nucleophilic C(sp^3^)–H fluorination. **A** Proposed catalytic cycle. **B** Radical trapping experiments. **C** Monitoring of (i) methane and (ii) methane-*d*_*1*_ evolution by PhotoNMR. **D** Investigation of regioselectivity via competition experiments among 3°, 2° and 1 °C(sp^3^)–H coupling partners. **E** Investigation of kinetic isotope effect via parallel initial rates experiment with ethylbenzene and ethylbenzene-*d*_10_. **F** Hammett analysis performed with the methyl radical precursor (left) and the methoxy radical precursor (right). ^*a*^For reaction conditions see Fig. 2 (^19^F NMR yields). ^*b*^Reaction performed with 1.5 equiv. TEMPO (^1^H NMR yield). ^*c*^See Supplementary Information for details.

Having evaluated the scope of this transformation, we set out to interrogate its mechanism (Fig. 5). According to our prior studies^18^ and literature precedent^63^, we propose that visible light irradiation of the photocatalyst Ir(*p*-F-ppy)_3_ generates a long-lived excited state that serves as a single-electron reductant of **1**. Fragmentation of the resulting radical anion followed by extrusion of CO_2_ forms phthalimide anion and methyl radical. Since methyl radical is thermodynamically disfavored to undergo oxidation by Ir^IV^, it is instead available to facilitate HAT with the C(sp^3^)−H coupling partner to deliver a carbon-centered radical and methane as a byproduct (*E*_1/2_^ox^ ~ 2.5 V vs. SCE for methyl radical). ORPC between Ir^IV^ and the substrate radical generates a carbocation and turns over the photocatalyst. Subsequent nucleophilic trapping of the carbocation intermediate furnishes the desired product (Fig. 5A).

Consistent with the proposed first step of this mechanism, emission quenching experiments demonstrated that **1** is the only reaction component that quenches the excited state of the photocatalyst (See Supplementary Information). Our analysis also indicates that the rate of quenching is moderately enhanced in the presence of Et_3_N•3HF. This observation is consistent with the higher yields observed when Et_3_N•3HF is employed as a catalytic additive for the construction of C(sp^3^)–O, C(sp^3^)–S, and C(sp^3^)–C bonds. The presence of an acidic additive could aid reduction of **1** via proton-coupled electron transfer, as reported for related systems in the literature^64^. We have considered additional roles for Et_3_N•3HF on the basis of the improved yields observed with this nucleophile as compared with those obtained with other nucleophiles in Fig. 4. These roles include preventing back-electron transfer, aiding fragmentation of reduced **1**, and modulating the photophysics of the photocatalyst via hydrogen bonding. Experimental studies are ongoing to probe these possibilities.

Next, radical trapping experiments were conducted to evaluate the identity of key radical intermediates in the proposed mechanism. When the fluorination of diphenylmethane was conducted under standard conditions in the presence of 1.5 equivalents of TEMPO, we observed the methyl radical–TEMPO adduct (**64**) in 32% yield, accompanied by nearly complete suppression of fluorination (Fig. 5B). In addition, when 1,1-diphenylethylene was employed as a substrate under standard conditions, nearly quantitative 1,2-carbofluorination was observed, wherein methyl radical addition into the olefin terminus followed by radical oxidation and nucleophilic fluorination delivered product **65**. (Fig. 5B). This example of carbofluorination not only provides clear evidence for methyl radical formation, but also serves as a useful framework for sequential C(sp^3^)–C(sp^3^) and C(sp^3^)–F alkene difunctionalization. As further evidence, in situ NMR studies revealed the evolution of methane gas as the reaction proceeded. (Fig. 5C). Moreover, upon performing in situ NMR studies with diphenylmethane-*d*_2_, we observed the evolution of CDH_3_, indicating that methyl radical indeed facilitates HAT from the substrate (Fig. 5C). While acyloxy radicals generated under photocatalytic conditions have been shown to mediate HAT^48^, we did not observe the evolution of acetic acid in these studies.

To our knowledge, methyl radical guided HAT has not been previously explored for photocatalytic C(sp^3^)–H functionalization^65,66^. As such, we set out to understand the reactivity and selectivity effects inherent to the system. We conducted a series of competition experiments with cumene, ethylbenzene, and toluene under standard C(sp^3^)–H fluorination conditions (Fig. 5D). We found that HAT mediated by methyl radical and subsequent ORPC is preferential for 3°>2°>1° benzylic C(sp^3^)–H bonds. The data suggest that steric or polarity effects associated with HAT from a mildly nucleophilic methyl radical are minimal in these systems. Instead, the observed site-selectivity is consistent with the relative BDFEs and radical oxidation potential of the tertiary, secondary, and primary substrates.

To probe the independent roles of HAT and radical oxidation, we first conducted a kinetic isotope effect (KIE) study with ethylbenzene. A KIE of 12.1 was measured via parallel initial rate experiments using ethylbenzene and ethylbenzene-*d*_10_ (Fig. 5E). The magnitude of the KIE is consistent with prior studies of HAT involving methyl radical and suggests that HAT is the turnover-limiting step^67,68^. To probe the effect of substrate electronics on a HAT-ORPC mechanism, a Hammett analysis of the relative rate of benzylic fluorination across a series of *para*-substituted ethylbenzenes (determined by competition experiments, see Supplementary Information) was performed (Fig. 5F). Given the mild nucleophilicity of methyl radical, we might expect electron-deficient ethylbenzenes to undergo fluorination at a faster rate than electron-rich ethylbenzenes. However, the measured *ρ* value of −0.64 ± 0.07 (R^2^ = 0.92) indicates that electron-rich ethylbenzenes undergo C(sp^3^)–H fluorination more favorably than electron-deficient derivatives. This result suggests that radical oxidation—which would show a strong preference for more electron-rich substrates due to enhanced carbocation stabilization—influences the product distribution, perhaps as a result of being an irreversible step after turnover-limiting HAT. In this scenario, the competing electronic effects in the HAT and radical oxidation steps result in a moderate *ρ* value. By comparison, a *ρ* value of −1.36 was observed using electrophilic methoxy radical precursor **3**, consistent with the matched electronic effects in the two steps (Fig. 5F). In addition, analysis of selectivity outcomes with respect to computed C(sp^3^)–H BDFEs across the ethylbenzene series indicates no significant correlation between product selectivity and BDFE (Supplementary Fig. 39). These findings are most consistent with turnover-limiting HAT followed by an irreversible, product-determining radical oxidation. The observation that radical precursors **1** and **3** afford different *ρ* values provides further evidence that HAT, rather than radical oxidation (which occurs independent of the radical precursor) is the turnover-limiting step. Further studies are ongoing to probe additional mechanistic details.

**Fig. 6:**
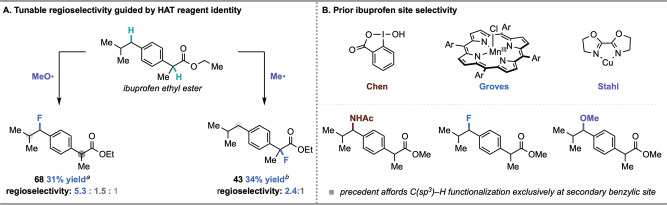
Investigations of site-selectivity with methoxy and methyl radical in the functionalization of ibuprofen ethyl ester. **A** Tunable selectivity for the C(sp^3^)–H functionalization of ibuprofen demonstrating favorable secondary benzylic fluorination with methoxy radical (left) and favorable tertiary benzylic fluorination with methyl radical (right). **B** Previous examples of site-selectivity in the C(sp^3^)–H functionalization of ibuprofen^6,15,44^. ^*a*^Reaction performed using abstractor **3** and standard reaction conditions described in Fig. 2. ^*b*^Reaction performed using abstractor **1** and standard reaction conditions described in Fig. 2.

Altogether, this work suggests that a HAT-ORPC strategy can provide a site-selective platform for C(sp^3^)–H functionalization. An advantage to this method is the utilization of phthalimide-derived species as redox-active HAT reagents; these reagents are not only readily available, but also are highly tunable. In this context, we questioned whether site-selectivity in the fluorination of ibuprofen ethyl ester—a complex substrate possessing various C(sp^3^)–H bonds—could be tuned on the basis of the radical species used in HAT (Fig. 6A). Under standard conditions with the methyl radical precursor **1**, the fluorination of ibuprofen ethyl ester favored C(sp^3^)–H functionalization at the tertiary benzylic site over the secondary benzylic site (**43**, 2.4:1 rr) (Fig. 6A). This site-selectivity is orthogonal to previously reported HAT-guided strategies (Fig. 6B)^6,15,44^ but consistent with our mechanistic studies that indicate a preference for tertiary C(sp^3^)–H functionalization according to BDFE and radical oxidation potential considerations (Fig. 5D). Furthermore, methyl radical is polarity matched to abstract a hydrogen atom proximal to an electron withdrawing group. By contrast, the prior art relies on electrophilic HAT mediators that are polarity mismatched to abstract a hydrogen atom proximal to an electron withdrawing group. As such, we hypothesized that employment of **3**, a precursor to the electrophilic methoxy radical, would afford distinct site-selectivity, favoring more electron-rich C(sp^3^)–H sites^69,70^. Indeed, we observed a reversal of site-selectivity in this case, wherein ibuprofen ethyl ester was fluorinated in 31% yield with a 5.3:1.5:1 rr favoring the secondary benzylic site (**68**). This example demonstrates the potential for this platform to engage readily available small molecule HAT reagents for tunable and predictable site-selective C(sp^3^)–H functionalization.

In conclusion, we have developed a photocatalytic method that employs widely available, low-cost nucleophiles and a readily accessible HAT precursor for C(sp^3^)–H fluorination, chlorination, etherification, thioetherification, azidation, and carbon–carbon bond formation. Mechanistic studies are consistent with methyl radical-mediated HAT and linear free-energy relationships suggest that radical oxidation influences site-selectivity. Furthermore, this approach was highly effective for the construction of multi-halogenated scaffolds and the late-stage functionalization of several bioactive molecules and pharmaceuticals with tunable regioselectivity.

## Methods

### General procedure for C(sp^3^)–H functionalization

To a 1-dram oven-dried vial, equipped with a Teflon stir bar, was added a Ir(*p*-F-ppy)_3_ (1.80 mg, 2.50 μmol, 1.00 mol %) and abstractor **1** (51.3 mg, 0.250 mmol, 1.00 equiv). The vial containing photocatalyst and abstractor **1** was then covered with a Kimwipe and pumped into a nitrogen-filled glovebox. To the reaction vial was added C(sp^3^)–H partner (1.50 mmol, 6.00 equiv), nucleophile (1.50 mmol, 6.00 equiv), and pivalonitrile (417 μL, 0.60 M). For reactions where triethylamine trihydrofluoride is not the nucleophile, triethylamine trihydrofluoride (6.1 μL, 0.04 mmol, 0.15 equiv.) was also added to the reaction mixture. The vial was capped, removed from the glovebox and sealed with electrical tape prior to irradiation. The reaction was stirred at 800 rpm for 6 h while illuminating with three 34 W blue LED lamps (Kessil KSH150B) and two cooling fans (Supplementary Fig. 1). The crude reaction mixture was passed through a short pad of silica, eluting with CDCl_3_, and analyzed by ^19^F NMR relative to 1-fluoronapthalene (32.3 μL, 0.250 mmol, 1.00 equiv) as an external standard.

## Supplementary information

Supplementary information file

## Data Availability

Materials and methods, experimental procedures, mechanistic studies, characterization data, spectral data, and xyz files (in accompanying zip drive) associated with computational data are available in the Supplementary Information.
