# Supplementary material for: A general strategy for C(sp3)–H functionalization with nucleophiles using methyl radical as a hydrogen atom abstractor
Source: Nat Commun. 2021 Nov 29;12:6950. doi: 10.1038/s41467-021-27165-z (PMC8630022; doi:10.1038/s41467-021-27165-z)
Supplement: Supplementary file 1 — Supplementary information file [file 41467_2021_27165_MOESM1_ESM.pdf]

A general strategy for C(sp^3)–H functionalization with nucleophiles using methyl radical as a hydrogen atom abstractor

Isabelle Nathalie-Marie Leibler,[1] Makeda A. Tekle-Smith,[1] and Abigail G. Doyle[1]

[1]Department of Chemistry, Princeton University, Princeton, New Jersey 08544, United States

Corresponding author email: agdoyle@princeton.edu

Supplementary Information

Table of Contents

Supplementary Methods 3

I. General Information 3

II. Selected Reaction Optimization and Control Experiments 5

A. Evaluation of Solvent. 6

B. Evaluation of Photocatalyst. 7

C. Evaluation of Fluoride Source. 8

D. Evaluation of C(sp^3)–H Partner Loading. 8

E. Evaluation of Et_3N•3HF Loading. 9

F. Photocatalyst Loading Experiments. 9

G. Concentration Screens. 10

H. Time Course. 10

I. Nucleophile Scope Optimization. 11

J. Control Studies. 12

III. Synthesis of Abstractors and Substrates 14

IV. Characterization of Fluorinated Products from C(sp^3)–H Partners 17

V. Characterization of Difluorination Scope 36

VI. Characterization of Nucleophile Addition Products 39

VII. Mechanistic Studies 47

A. Spectroscopic and Emission Quenching Experiments. 47

B. Light-Dark Cycle Experiment. 51

C. *In situ* NMR. 53

D. Methyl Radical Trapping Experiments. 56

E. Competition Experiments. 57

F. Kinetic Isotope Effect Studies. 58

G. Hammett Studies. 61

VIII. Electrophilic versus Nucleophilic Fluorination 66

IX. Calculation of Bond Dissociation Enthalpies 69

XI. NMR Spectra 72

Supplementary References 158

Supplementary Methods

I. General Information

Materials. Pivalonitrile was purchased from ACROS Organics™ (98%, anhydrous). Tris[5-fluoro-(2-pyridinyl-κ*N*)phenyl-κ*C*]iridium(III) (Ir(*p*-F-ppy)$_3$) was purchased from Strem. Triethylamine trihydrofluoride was purchased from Sigma-Aldrich.

Methods. Unless otherwise noted, reactions were performed with rigorous exclusion of air and moisture. Anhydrous pivalonitrile was passed through a plug of neutral alumina, degassed with nitrogen for 1h, and brought into a nitrogen-filled glovebox without further purification where it was stored over 4Å molecular sieves. Other solvents were distilled over calcium hydride or degassed prior to use unless otherwise noted. Triethylamine trihydrofluoride, in a PTFE plastic vial, was degassed under nitrogen for 1 h and brought into a nitrogen-filled glovebox without further purification. When handling triethylamine trihydrofluoride, it is recommended to have ready access to a tube of calcium gluconate in case of accidental exposure. Diphenylmethane was distilled over calcium hydride then degassed under nitrogen for 30 min and brought into a nitrogen-filled glovebox in a sealed vial. Reactions were monitored by thin-layer chromatography (TLC) on EMD Silica Gel 60 F254 plates, visualizing with UV-light (254 nm) fluorescence quenching. Organic solutions were concentrated under reduced pressure using a rotary evaporator (23 °C, <50 torr) unless otherwise noted (0 °C for more volatile compounds). Automated column chromatography was performed using silica gel cartridges on a Biotage Isolera 4 (40-53 μm, 60 Å). Preparative thin-layer chromatography was performed using 20 cm x 20 cm glass-backed silica preparative thin-layer chromatography plates (1000 μm layer thickness) from SiliCycle.

Instrumentation. Proton nuclear magnetic resonance (^{1}H NMR) spectra and carbon nuclear magnetic resonance (^{13}C NMR) spectra were recorded on a Bruker 500 AVANCE equipped with a cryoprobe (500 and 125 MHz, respectively). Chemical shifts for protons are reported in parts per million (ppm) downfield from tetramethylsilane and are referenced to residual protium in the NMR solvent ($CHCl_3$ = 7.26 ppm). Chemical shifts for carbon are reported in parts per million downfield from tetramethylsilane and are referenced to the carbon resonance of the solvent peak ($CDCl_3$ = 77.16 ppm). Fluorine nuclear magnetic resonance (^{19}F NMR) spectra were recorded on a Bruker 300 AVANCE (282 MHz) and are referenced to 1-fluoronaphthalene (-124.0 ppm). NMR data are represented as follows: chemical shift (δ ppm), multiplicity (s = singlet, d = doublet, t = triplet, q = quartet, p = pentet, hept = heptet, m = multiplet, br = broad), coupling constant (*J*) in Hertz (Hz), integration. All NMR spectra were taken at 25 °C. High-resolution mass spectra (HR/MS) were obtained on an Agilent 6220 LC/MS with an electrospray ionization time-of-flight (ESI-TOF) detector. Fourier-transform infrared spectroscopy (FT-IR) and Fourier-transform attenuated total reflection (FT-ATR) spectra were recorded on a Perkin-Elmer Spectrum 100 and are reported in terms of frequency of absorption (cm^{-1}). Gas chromatography (GC) was performed on an Agilent 7890A series instrument equipped with a split-mode capillary injection system and flame ionization detectors (FID). Liquid chromatography/mass spectrometry (LC/MS) data was

obtained on an Agilent 1260 Infinity instrument with a binary pump, a diode array detector, and an Agilent 6120 quadrupole detector. High-performance liquid chromatography (HPLC) was performed on an Agilent 1200 series instrument with a binary pump and a diode array detector with Chiralpak AS-H, Chiralpak AD-H, Chiralpak IC, and Chiralpak ID columns (25 cm x 0.46 cm).

Light Sources. Reactions were carried out using 34W Blue LED lamps (Kessil H150 LED Grow Lights) purchased from Kessil (λ_{max} = 450 nm flanked by a second peak at λ = 422 nm). Kessil lamps were placed approximately 2 cm away from 0.5 or 1.0-dram reaction vial. A fan was placed 6 inches above the Kessil lamp for cooling when one or two Kessil lamps were in use, and a second fan was placed in front when 3 Kessil lamps were used. A total of two 0.5-1.0 dram vials were placed in front of each lamp.

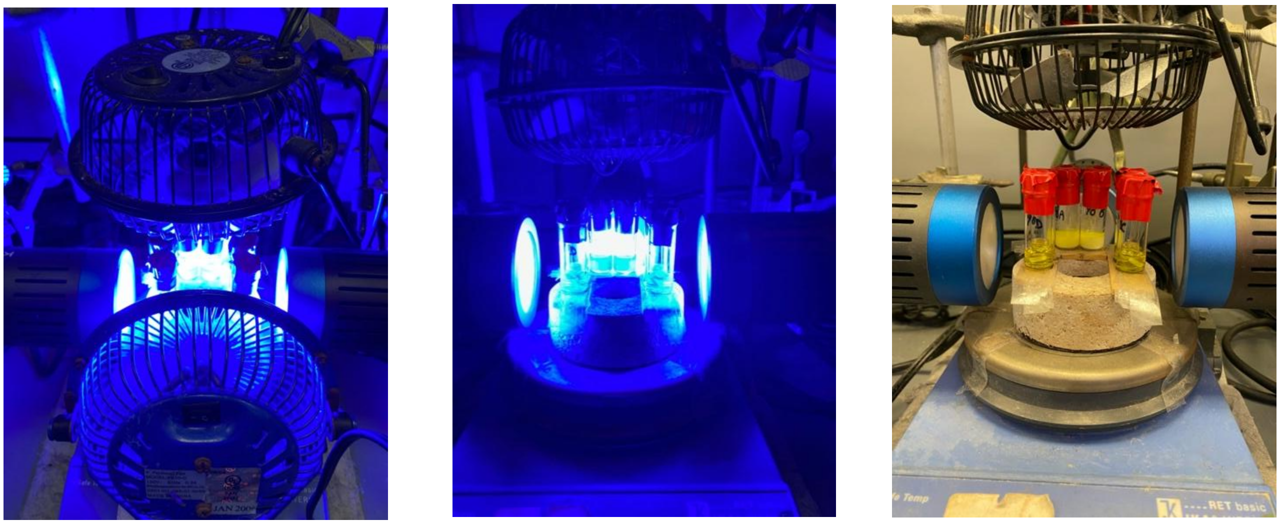

Supplementary Figure 1. Representative reaction light setups.

II. Selected Reaction Optimization and Control Experiments

General procedure A for reaction optimization:

Reagent handling: Ir(*p*-F-ppy)$_3$ was stored in a Drierite-containing desiccator and weighed out on the bench. Phthalimide abstractors **1**, **3**-**5** were prepared according to literature procedures,[1–3] dried under vacuum for 48 h, stored in a Drierite-containing desiccator, and weighed out on the bench. Triethylamine trihydrofluoride, in a PTFE plastic vial, was degassed under nitrogen for 1 h and brought into a nitrogen-filled glovebox without further purification. Anhydrous pivalonitrile was passed through a plug of neutral alumina, degassed under nitrogen for 1 h, and stored in the glovebox over 4Å molecular sieves. Diphenylmethane and 4,4'-diphenylmethane were distilled over calcium hydride then degassed under nitrogen for 30 min and brought into a nitrogen-filled glovebox in a sealed vial.

Reaction setup (0.150 mmol scale): To a 1-dram oven-dried vial, equipped with a Teflon stir bar, was added photocatalyst (1.5 μmol, 1.0 mol %) and an appropriate phthalimide-derived abstractor (0.150 mmol, 1.00 equiv). The vial containing photocatalyst and phthalimide abstractor was then covered with a Kimwipe and pumped into the glovebox. To the reaction vial was added diphenylmethane (150 μL, 0.900 mmol, 6.00 equiv), triethylamine trihydrofluoride (147 μL, 0.900 mmol, 6.00 equiv), and solvent (250 μL, 0.600 M). The vial was then capped, removed from the glovebox and sealed with electrical tape prior to irradiation. The reaction was stirred at 400 rpm for 1-12 h while illuminating with three 34W blue LED lamps (Kessil KSH150B) and two cooling fans (**Supplementary Figure 1**). The crude reaction mixture was passed through a short pad of silica, eluting with $CDCl_3$, and analyzed by ^{19}F NMR relative to 1-fluoronapthalene (25.8 μL, 0.200 mmol, 1.33 equiv) as an external standard.

A. Evaluation of Solvent.

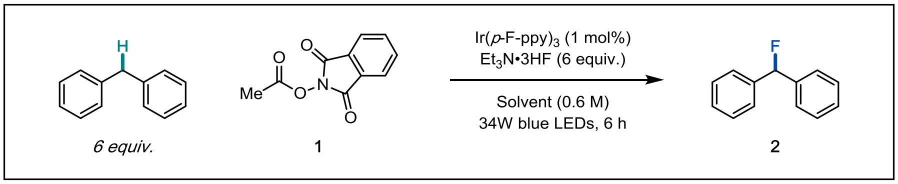

Entry	Deviation	Yield of 2 (%)
1	*t*-BuCN	88
2	MeCN	25
3	CH_2Cl_2	44
4	$CHCl_3$	40
5	1,2-difluorobenzene, Ir(CF_3-ppy)$_3$	60
6	Benzene, Ir(CF_3-ppy)$_3$	72

Supplementary Figure 2. Solvents screened according to general procedure A (0.15 mmol scale reaction). Yield of **2** determined via ^{19}F NMR by comparison to 1-fluoronaphthalene as an external standard.

The results reported in **Supplementary Figure 2** show the highest yielding solvent/photocatalyst combinations. In our examination of reaction solvent, we found pivalonitrile to be optimal for this transformation. Solvents possessing abstractable C–H bonds, such as acetonitrile or dichloromethane, resulted in competitive HAT from solvent and therefore fluorination in only moderate yield (**Supplementary Figure 2**, entries 2 and 3). Employing CD_3CN or CD_2Cl_2 restored reaction reactivity by minimizing competitive HAT. Fluorination was also suppressed in the presence of aromatic solvents, due to competitive arene functionalization by methyl radical (**Supplementary Figure 2**, entries 5 and 6). Interestingly, we do not observe Ritter-type products to any appreciable extent (yields < 3%). We reason this is due to the mild and anhydrous nature of the reaction conditions as more forcing or aqueous conditions are typically required to favor Ritter chemistry. Additionally, Ritter-type chemistry between pivalonitrile and C(sp^3)–H substrates is typically performed under more forcing conditions (e.g., elevated temperatures and acidic hydrolysis with 10 equiv. TFA).[4]

B. Evaluation of Photocatalyst.

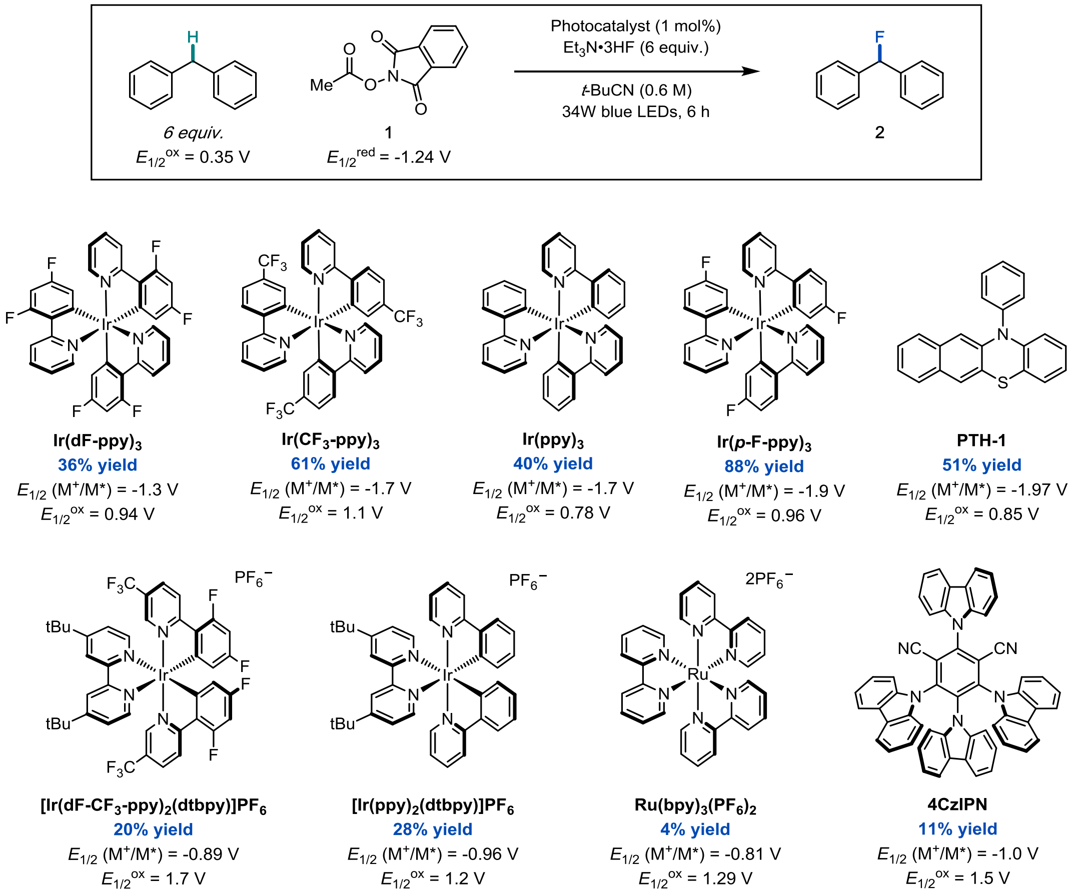

Supplementary Figure 3. Extended series of photocatalysts evaluated in reaction optimization. Photocatalysts screened according to general procedure A (0.15 mmol scale reaction). Yield of **2** determined via ^{19}F NMR by comparison to 1-fluoronaphthalene as an external standard.

C. Evaluation of Fluoride Source.

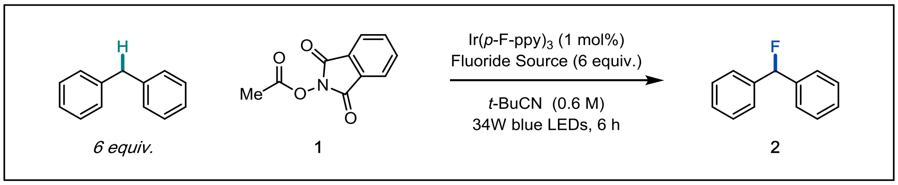

Entry	Fluoride Source	Conversion of 1	Yield of 2 (%)
1	$Et_3N•3HF$	100%	88
2	KF	64%	0
3	KF/HFIP	33%	0
4	KF/*t*-BuOH	19%	0
5	KF/NMP	82%	1
6	CsF	63%	0
7	$Et_4NF•H_2O$	32%	0
8	*n*-Bu_4NF	5%	0

Supplementary Figure 4. Fluoride sources screened according to general procedure A (0.15 mmol scale reaction). Yield of **2** determined via ^{19}F NMR by comparison to 1-fluoronaphthalene as an external standard. Conversion of **1** was determined via ^{1}H NMR by comparison to 1-fluoronaphthalene as an external standard.

D. Evaluation of C(sp^3)–H Partner Loading.

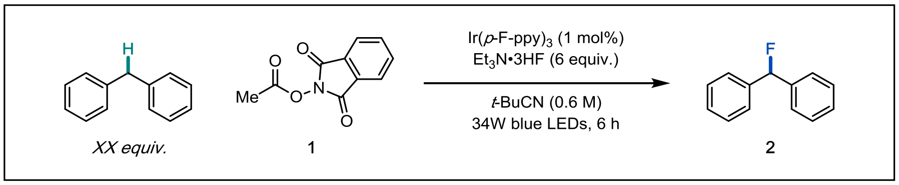

Entry	Diphenylmethane : 1 (equiv.)	Yield of 2 (%)
1	12 : 1	91
2	6 : 1	88
3	3 : 1	53
4	2 : 1	32
5	3 : 2	25
6	3 : 2.5	18
7	1 : 1	17
8	1 : 2	13

Supplementary Figure 5. Stoichiometry relationships between C(sp^3)–H coupling partner and HAT reagent evaluated in the optimization of C(sp^3)–H functionalization. Screening was performed according to general procedure A (0.15 mmol scale reaction). Yield of **2** determined via ^{19}F NMR by comparison to 1-fluoronaphthalene as an external standard.

E. Evaluation of $Et_3N•3HF$ Loading.

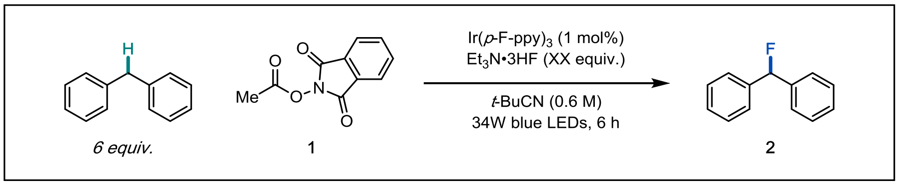

Entry	$Et_3N•3HF$ (equiv.)	Yield of 2 (%)
1	6	**88**
2	1	**55**
3	0.5	**37**
4	0.15	**11**

Supplementary Figure 6. Optimization of fluoride loading for C(sp^3)–H fluorination. Screening was performed according to general procedure A (0.15 mmol scale reaction). Yield of **2** determined via ^{19}F NMR by comparison to 1-fluoronaphthalene as an external standard.

F. Photocatalyst Loading Experiments.

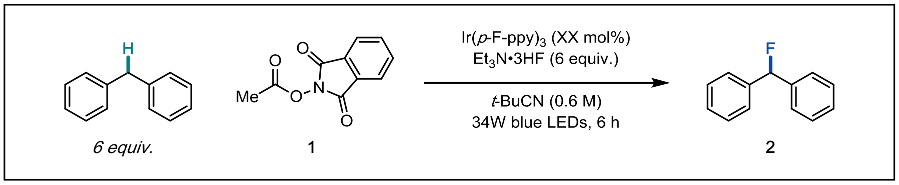

Entry	Photocatalyst Loading (mol%)	Turnovers	Yield of 2 (%)
1	1.0	88	**88**
2	0.5	154	**77**
3	0.25	292	**73**
4	0.125	440	**55**
5	0.0625	688	**43**
6	0.01	1100	**11**

Supplementary Figure 7. Photocatalyst loadings for Ir(*p*-F-ppy)$_3$ with corresponding photocatalyst turnover numbers. Screening was performed according to general procedure A (0.15 mmol scale reaction). Yield of **2** determined via ^{19}F NMR by comparison to 1-fluoronaphthalene as an external standard.

Note: For photocatalyst loading studies at 0.25 mol % and below, Ir(*p*-F-ppy)$_3$ was added as a stock solution in pivalonitrile to ensure accurate addition of very small photocatalyst quantities.

G. Concentration Screens.

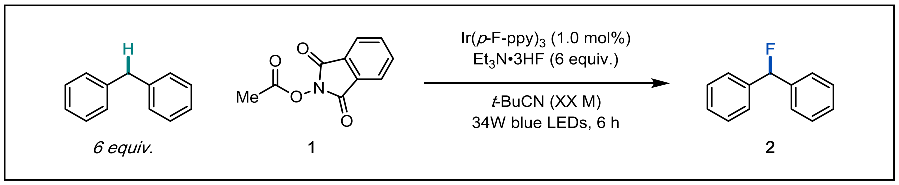

Entry	Concentration (M)	Yield of 2 (%)
1	0.1	49
2	0.2	53
3	0.3	55
4	0.4	59
5	0.6	88
6	0.8	51

Supplementary Figure 8. Concentration screening was performed according to general procedure A (0.15 mmol scale reaction). Yield of **2** determined via ^{19}F NMR by comparison to 1-fluoronaphthalene as an external standard.

H. Time Course.

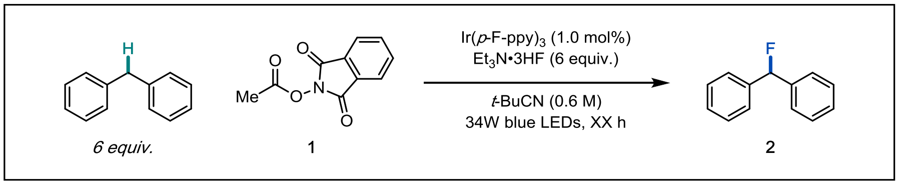

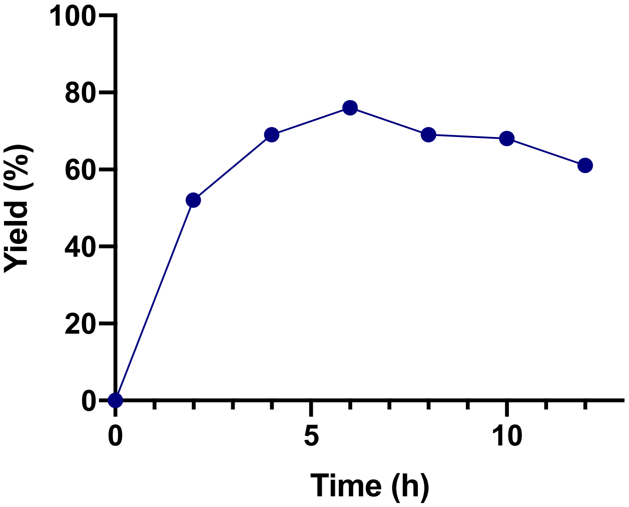

Supplementary Figure 9. Plotted reaction time course for C(sp^3)–H fluorination between 0-12 hours. Time course screening was performed according to general procedure A (0.15 mmol scale reaction). Yield of **2** determined via ^{19}F NMR by comparison to 1-fluoronaphthalene as an external standard.

The results of our time course evaluations suggest that optimal yield is observed at a 6-hour time point. Interestingly, we observed a decrease in yield following the 6-hour reaction time. We hypothesize that product activation and decomposition may be operative during these later time points, a phenomenon reported in prior literature regarding organofluorides under acidic conditions.[5,6] This hypothesis, however, applies specifically to activated C(sp^3)–H coupling partners, such as diphenylmethane.

I. Nucleophile Scope Optimization.

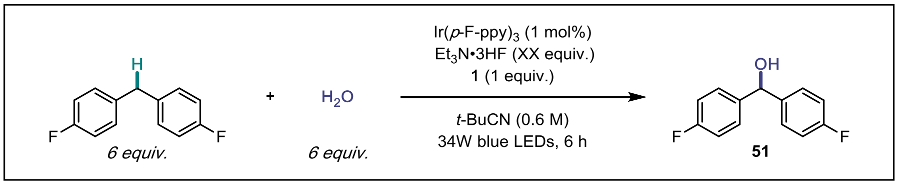

Entry	Equiv. $Et_3N•3HF$	Yield of 51 (%)	Yield of 6 (%)
1	0	18	0
2	0.05	31	0
3	0.10	29	0
4	0.15	31	0
5	0.30	25	3

Supplementary Figure 10. $Et_3N•3HF$ additive loading was screened according to general procedure A (0.15 mmol scale reaction). Yield of **51** was determined via ^{19}F NMR by comparison to 1-fluoronaphthalene as an external standard.

We considered the possibility that nucleophilic functionalization could proceed via nucleophilic substitution from an *in situ* generated benzylic fluoride, as reported for a related system in the literature.[7] However, in that instance Stahl and coworkers reported the need for an additive such as hexafluoroisopropanol or a strong Lewis acid such as boron trifluoride etherate to accomplish the sequential fluorination and substitution. For the work presented herein, hydroxylation (**Supplementary Figure 10**, entry 1), chlorination, and azidation proceed in the absence of $Et_3N•3HF$. Moreover, optimal yields are seen with sub-stoichiometric 0.05-0.15 amounts of $Et_3N•3HF$ (**Supplementary Figure 10**, entry 2-4). Employing higher than 0.15 equiv of $Et_3N•3HF$ led to competitive fluorination to yield product **6** (**Supplementary Figure 10**, entry 5). Furthermore, subjecting *in situ* generated fluorination product **6** to reaction conditions with water as a nucleophile produced a trace amount of hydroxylation (**51**) (**Supplementary Figure 11A**). However, upon addition of an acidic additive—such as $BF_3•Et_2O$ used by Stahl and coworkers—successful nucleophilic substitution was achieved with water (**Supplementary Figure 11B**) and trimethylacetamide (**Supplementary Figure 11C**). Altogether, these results suggest that functionalization does not proceed through nucleophilic substitution from an *in situ* generated benzylic fluoride to an appreciable extent.

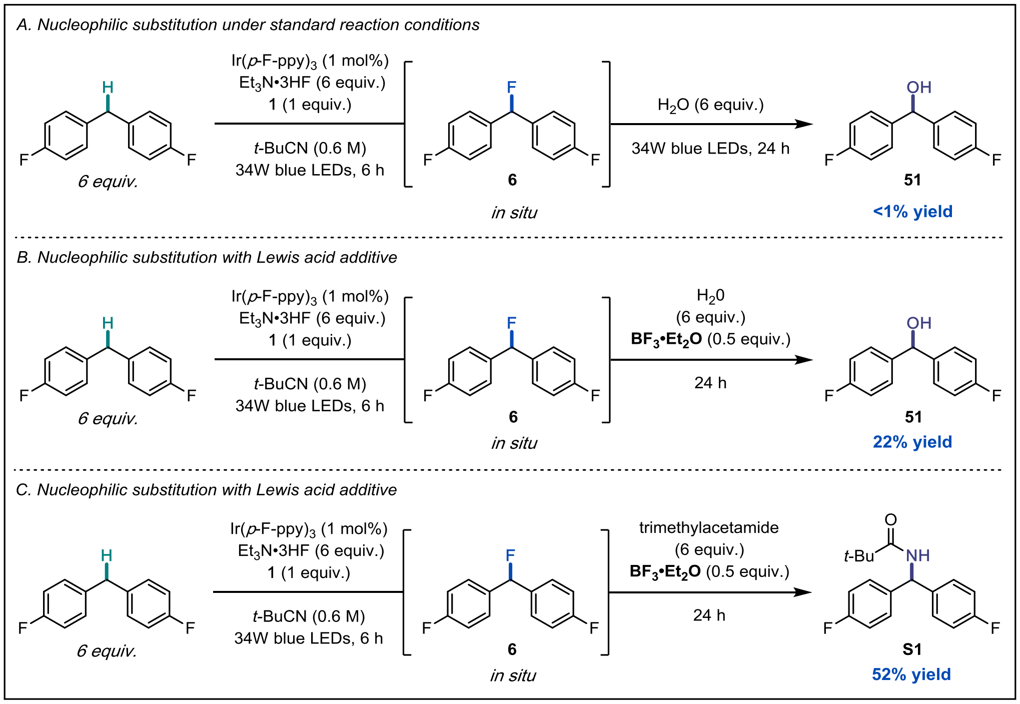

Supplementary Figure 11. Nucleophilic substitution studies were performed according to general procedure A (0.15 mmol scale reaction). Yield determined via ^{19}F NMR by comparison to 1-fluoronaphthalene as an external standard.

We hypothesize that reduced yields are observed for nucleophiles other than Et_3N•3HF as a result of secondary roles Et_3N•3HF plays in the reaction. We propose that Et_3N•3HF acts not only as a nucleophile, but also plays a role in productive electron transfer between photoexcited Ir(III) and phthalimide ester 1 and subsequent decarboxylative fragmentation over back electron transfer (See **Mechanistic Studies** for further discussion). Other nucleophiles capable of hydrogen bonding can also perform this role, however lower yields and poor conversion of **1** would suggest that the other nucleophiles surveyed are less effective in this capacity than Et_3N•3HF.

J. Control Studies.

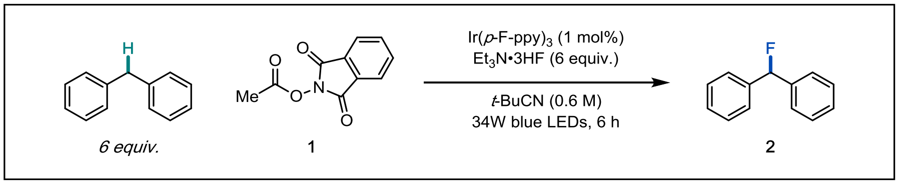

Entry	Deviation	Yield of 2 (%)
1	No deviation	88
2	No Et_3N•3HF	0
3[a]	No photocatalyst	0
4	No **1**	0
5[a]	No light	0

Supplementary Figure 12. Control studies were performed according to general procedure A (0.15 mmol scale reaction). Yield of **2** determined via ^{19}F NMR by comparison to 1-fluoronaphthalene as an external standard. [a] 0% conversion of **1** was observed.

III. Synthesis of Abstractors and Substrates

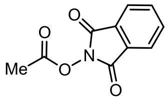

1,3-dioxoisoindolin-2-yl acetate (1): A 25 mL round-bottom flask, equipped with a stir bar, was charged with *N*-hydroxyphthalimide (2.45 g, 15.0 mmol, 1.00 equiv) and acetic anhydride (7.37 mL, 78.0 mmol, 5.20 equiv). The pale yellow suspension was stirred open to ambient atmosphere for 24 h, after which the resulting white suspension was filtered and washed with pentane (3 x 100 mL, chilled to 0 °C). Drying *in vacuo* afforded the title compound as a white powder (2.54 g, 82% yield). Characterization data are in agreement with reported literature values.[8]

^{1}H NMR (500 MHz, $CDCl_3$): δ 7.89 (dd, *J* = 5.5, 3.1 Hz, 2H), 7.79 (dd, *J* = 5.5, 3.1 Hz, 2H), 2.40 (s, 3H).

^{13}C NMR (126 MHz, $CDCl_3$): δ 166.7, 162.0, 134.9, 129.0, 124.2, 17.8.

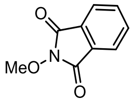

2-methoxyisoindoline-1,3-dione (3): A 100 mL round-bottom flask, equipped with a stir bar, was charged with *N*-hydroxyphthalimide (1.63 g, 10.0 mmol, 1.00 equiv) and sealed under nitrogen with a rubber septum. To the flask was then added dimethylformamide (45.0 mL, 0.211 M) and 1,8-diazabicyclo[5.4.0]undec-7-ene (1.67 g, 11.0 mmol, 1.10 equiv). The reaction solution appeared dark red/black in color. After stirring for five minutes, the flask was charged with iodomethane dropwise (745 µL, 12.0 mmol, 1.20 equiv) and the reaction mixture was stirred overnight under nitrogen at room temperature. The reaction mixture was then poured into a separation funnel containing 50 mL water and extracted with diethyl ether (3 x 50 mL). The combined organic layers were then concentrated *in vacuo* and the resulting residue was subjected to automated column chromatography (10-35% EtOAc/hexanes). Concentrating and drying in vacuo afforded the title compound as a white solid (1.04 g, 59% yield). Characterization data are in agreement with reported literature values.[1]

^{1}H NMR (500 MHz, $CDCl_3$): δ 7.85 (dd, *J* = 5.4, 3.1 Hz, 2H), 7.76 (dd, *J* = 5.4, 3.0 Hz, 2H), 4.07 (s, 3H).

^{13}C NMR (126 MHz, $CDCl_3$): δ 163.4, 134.7, 129.1, 123.7, 66.0.

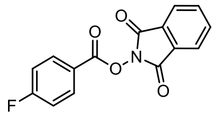

1,3-dioxoisoindolin-2-yl 4-fluorobenzoate (4): A 100 mL round-bottom flask, equipped with a stir bar, was charged with *N*-hydroxyphthalimide (3.26 g, 20.0 mmol, 1.00 equiv), 4-fluorobenzoic acid (3.36 g, 24.0 mmol, 1.20 equiv), and *N,N*-dimethylpyridin-4-amine (244 mg, 2.00 mmol, 0.010 equiv). The flask was then sealed under nitrogen with a rubber septum. The flask was then charged with dichloromethane (45.0 mL, 0.473 M) and *N,N*-diisopropylmethanediimine (2.78 g, 22.0 mmol, 1.10 equiv) was added dropwise. The resulting yellow reaction mixture was stirred overnight under nitrogen at room temperature. The reaction mixture was then concentrated *in vacuo*, and the resulting yellow residue was then dissolved in dichloromethane and subjected to automated column chromatography using a gradient of 5-25% EtOAc/hexanes. Concentrating and drying *in vacuo* afforded the title compound as a white powder (0.49 g, 9% yield). Characterization data are in agreement with reported literature values.[3]

^{1}H NMR (500 MHz, $CDCl_3$): δ 8.30 – 8.19 (m, 2H), 7.94 (dd, *J* = 5.5, 3.1 Hz, 2H), 7.83 (dd, *J* = 5.5, 3.1 Hz, 2H), 7.25 – 7.20 (m, 2H).

^{13}C NMR (126 MHz, $CDCl_3$): δ 168.0, 166.0, 162.1 (d, *J* = 18.1 Hz), 135.0, 133.6 (d, *J* = 9.8 Hz), 129.1, 124.2, 121.7 (d, *J* = 3.1 Hz), 116.5 (d, *J* = 22.4 Hz).

^{19}F NMR (282 MHz, $CDCl_3$): δ -101.4 – -101.5 (m, 1F).

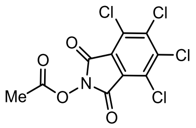

4,5,6,7-tetrachloro-1,3-dioxoisoindolin-2-yl acetate (5): A 20.0 mL scintillation vial, equipped with a stir bar, was charged with 4,5,6,7-tetrachloro-2-hydroxyisoindoline-1,3-dione (2.26 g, 7.50 mmol, 1.00 equiv) and acetic anhydride (3.69 mL, 39.0 mmol, 5.20 equiv). The suspension was stirred open to ambient atmosphere for 24 h, after which the resulting white suspension was filtered and washed with pentane (3 x 100 mL, chilled to 0 °C). Drying *in vacuo* afforded the title compound as a white powder (1.80 g, 70% yield).

^{1}H NMR (500 MHz, $CDCl_3$): δ 2.41 (s, 3H).

^{13}C NMR (126 MHz, $CDCl_3$): δ 166.3, 157.6, 141.2, 130.7, 124.8, 17.7.

FTIR (ATR, cm^{-1}): 3201, 3075, 1771, 1704, 1365, 1315, 1082, 913, 744, 711, 648, 606, 585, 468.

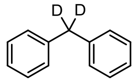

diphenylmethane-*d*$_2$: A flame-dried vial was charged with anhydrous $AlCl_3$ (472 mg, 3.54 mmol, 1.77 equiv) and lithium aluminum deuteride (84.0 mg, 2.00 mmol, 1.00 equiv) under nitrogen atmosphere. The mixture was carefully suspended in dry ether (8 mL). Benzophenone (364 mg, 2.00 mmol, 1.00 equiv) was carefully added as solid (violent reaction) to the suspension. The mixture was stirred for 1 h at room temperature, diluted with ether (20 mL) and quenched by the addition of aqueous HCl (1 M). The phases where separated, and the aqueous phase was extracted with ether (3 x 10 mL). The organic phase was dried over sodium sulfate, filtered, and concentrated. The resulting crude oil was purified by silica gel column chromatography, eluting with 100% pentane, to obtain the title compound as a white solid (252 mg, 74% yield). Characterization data are in agreement with reported literature values.[9]

^{1}H NMR (500 MHz, CDCl$_3$): δ 7.29 (dd, *J* = 8.6, 6.7 Hz, 4H), 7.21 (m, 6H).

^{13}C NMR (126 MHz, CDCl$_3$): δ 141.2, 129.0, 128.6, 126.2.

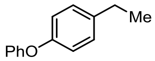

1-Ethyl-4-phenoxybenzene (S2) was prepared according to literature procedure.[10] To an oven-dried round bottom flask was added phenol (2.80 g, 30.0 mmol, 1.50 equiv), cesium carbonate (14.0 g, 40.0 mmol, 2.00 equiv), copper (I) iodide (381 mg, 2.00 mmol, 10 mol %), and *N*, *N*- dimethylglycine, HCl salt (837 mg, 6.00 mmol, 30 mol %). The flask was then charged with 4-bromoethylbenzene (2.70 mL, 20.0 mmol, 1.00 equiv) and dioxane (34.6 mL, 0.5 M). The reaction was stirred at 90 °C for 72 hours. The reaction mixture was then cooled to room temperature and diluted with ethyl acetate (20 mL) and water (50 mL). The aqueous layer was extracted with ethyl acetate (3 x 20 mL). The combined organic layers were washed (2 x 30 mL) with brine and dried over sodium sulfate, filtered, and concentrated *in vacuo* to give a dark orange liquid. This liquid was then subjected to automated column chromatography using a gradient of 0-1% EtOAc/hexanes to obtain **S2** as a clear oil (3.0 g, 76% yield). Characterization data are in agreement with reported literature values.[10]

^{1}H NMR (500 MHz, CDCl$_3$): δ 7.50 (m, 2H), 7.36 (dd, *J* = 9.0, 2.1 Hz, 2H), 7.30 – 7.08 (m, 5H), 2.84 (qd, *J* = 7.5, 1.6 Hz, 2H), 1.48 – 1.44 (m, 3H).

^{13}C NMR (126 MHz, CDCl$_3$): δ 157.9, 155.0, 139.4, 129.8, 129.1, 122.9, 119.2, 118.5, 28.3, 15.9.

IV. Characterization of Fluorinated Products from C(sp^3)–H Partners

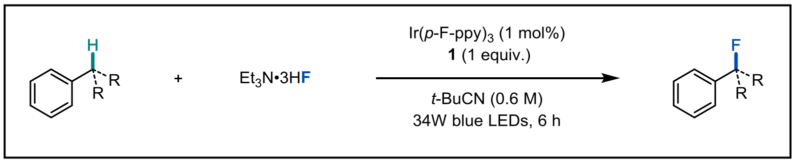

General procedure B for fluorination C(sp^3)–H partners:

Reagent handling: Ir(*p*-F-ppy)$_3$ was stored in a Drierite-containing desiccator and weighed out on the bench. Phthalimide abstractor **1** was prepared according to literature procedure,[8] dried under vacuum for 48 h, stored in a Drierite-containing desiccator, and weighed out on the bench. Triethylamine trihydrofluoride, in a PTFE plastic vial, was degassed under nitrogen for 1 h and brought into the glovebox without further purification. Anhydrous pivalonitrile was passed through a plug a neutral alumina, degassed under nitrogen for 1 h, and stored in the glovebox over 4Å molecular sieves. Diphenylmethane, 4,4′-difluorodiphenylmethane, cumene, and 1,3,5-triisopropylbenzene were distilled over calcium hydride then degassed under nitrogen for 30 min and brought into the glovebox in sealed vials. Cumene and 1,3,5-triisopropylbenzene were used directly after purification as oxidation of the starting materials can occur over time even while stored in a nitrogen-filled glovebox. *N*-(((1R,4aS)-7-isopropyl-1,4a-dimethyl-1,2,3,4,4a,9,10,10a-octahydrophenanthren-1-yl)methyl)benzamide was prepared according to literature procedure.[11]

Reaction setup (0.250 mmol scale): To a 1-dram oven-dried vial, equipped with a Teflon stir bar, was added a Ir(*p*-F-ppy)$_3$ (1.80 mg, 2.50 μmol, 1.00 mol %) and phthalimide-derived abstractor **1** (51.3 mg, 0.250 mmol, 1.00 equiv). The vial containing photocatalyst and phthalimide abstractor was then covered with a Kimwipe and pumped into a nitrogen-filled glovebox. To the reaction vial was added C(sp^3)–H partner (1.50 mmol, 6.00 equiv), triethylamine trihydrofluoride (245 μL, 1.50 mmol, 6.00 equiv), and pivalonitrile (417 μL, 0.60 M). The vial was capped, removed from the glovebox and sealed with electrical tape prior to irradiation. The reaction was stirred at 800 rpm for 6 h while illuminating with three 34W blue LED lamps (Kessil KSH150B) and two cooling fans (**Supplementary Figure 1**). The crude reaction mixture was passed through a short pad of silica, eluting with $CDCl_3$, and analyzed by ^{19}F NMR relative to 1-fluoronapthalene (32.3 μL, 0.250 mmol, 1.00 equiv) as an external standard.

Notes on purification: Some benzylic fluoride products were found to be unstable towards standard purification conditions, leading to hydrolysis or elimination products. These compounds were characterized by ^{19}F NMR and ^{1}H NMR of the crude reaction mixtures. Additionally, fluorination of lighter, more volatile C(sp^3)–H partners resulted in the formation of volatile products that were either fully or partially lost in purification.[12–14] In these cases, the products were isolated by preparatory thin-layer chromatography eluting with 100% pentane. Spectral data were obtained without concentrating the material, often resulting in spectra still containing trace amounts of pivalonitrile.

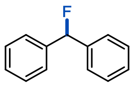

Fluorodiphenylmethane (2) was prepared from diphenylmethane (251 µL, 1.50 mmol, 6.00 equiv) according to general procedure B. The title compound was purified via preparatory thin-layer chromatography, eluting with 100% petroleum ether (80% NMR yield, average of two runs; Run 1: 80% yield, Run 2: 79% yield). Characterization data is consistent with reported literature values.[15]

^{1}H NMR (500 MHz, $CDCl_3$): δ 7.31 – 7.25 (m, 10H), 6.40 (d, *J* = 47.4 Hz, 1H).

^{13}C NMR (126 MHz, $CDCl_3$): δ 139.8 (d, *J* = 21.7 Hz), 128.5, 128.4 (d, *J* = 2.2 Hz), 126.6 (d, *J* = 6.3 Hz), 94.5 (d, *J* = 172.6 Hz).

^{19}F NMR (282 MHz, $CDCl_3$): δ -166.8 (d, *J* = 47.5 Hz, 1F).

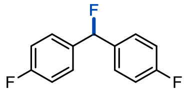

4,4'-(fluoromethylene)bis(fluorobenzene) (6) was prepared from 4,4′-difluorodiphenylmethane (268 µL, 1.50 mmol, 6.00 equiv) according to general procedure B. The title compound was purified via preparatory thin-layer chromatography, eluting with 100% petroleum ether (77% NMR yield, average of two runs; Run 1: 75% yield, Run 2: 78% yield). The title compound was isolated and characterized with hydrolysis product **51,** which was formed under isolation conditions. Characterization data is consistent with reported literature values.[16]

^{1}H NMR (500 MHz, $CDCl_3$): δ 7.38 – 7.28 (m, 4H), 7.16 – 7.00 (m, 4H), 6.46 (d, *J* = 47.4 Hz, 1H).

^{13}C NMR (126 MHz, $CDCl_3$): δ 162.9 (dd, *J* = 247.5, 2.4 Hz), 135.6 (dd, *J* = 22.2, 3.2 Hz), 128.6 (dd, *J* = 8.3, 6.1 Hz), 115.6 (dd, *J* = 21.6, 14.3 Hz), 93.4 (d, *J* = 173.3 Hz).

^{19}F NMR (282 MHz, $CDCl_3$): δ -113.2 (m, 2F), -163.3 (m, 1F).

FTIR (ATR, cm^{-1}): 3085, 3061, 3025, 2961, 2926, 2848, 1676, 1508, 1444, 1405, 1271, 1221, 914, 774, 757, 701, 610, 471.

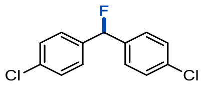

4,4'-(fluoromethylene)bis(chlorobenzene) (7) was prepared from 4,4′-dichlorodiphenylmethane (356 mg, 1.50 mmol, 6.00 equiv) according to general procedure B. The title compound was purified via preparatory thin-layer chromatography, eluting with 100% petroleum ether (50% NMR yield, average of two runs; Run 1: 52% yield, Run 2: 48% yield). Characterization data is consistent with reported literature values.[17]

^{1}H NMR (500 MHz, $CDCl_3$): δ 7.40 – 7.34 (m, 4H), 7.26 (m, 4H), 6.43 (d, *J* = 47.0 Hz, 1H).

^{13}C NMR (126 MHz, $CDCl_3$): δ 137.8 (d, *J* = 22.1 Hz), 134.6 (d, *J* = 2.6 Hz), 128.8, 127.9 (d, *J* = 6.2 Hz), 93.1 (d, *J* = 174.3 Hz).

^{19}F NMR (282 MHz, $CDCl_3$): δ -166.6 (d, *J* = 47.0 Hz, 1F).

FTIR (ATR, cm^{-1}): 2961, 2944, 2926, 2828, 1672, 1497, 1444, 1407, 1273, 1026, 738, 702, 609.

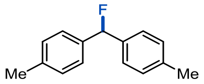

4,4'-(fluoromethylene)bis(methylbenzene) (8) was prepared from 4,4′-dimethyldiphenylmethane (301 µL, 1.50 mmol, 6.00 equiv) according to general procedure B. After reacting for 6 h, the reaction mixture was passed through a short plug of silica gel. The yield of the title compound was determined by ^{19}F NMR analysis of the crude reaction (58% NMR yield, average of two runs; Run 1: 61% yield, Run 2: 55% yield). Characterization data is consistent with reported literature values.[18]

^{19}F NMR (282 MHz, $CDCl_3$): δ -164.6 (d, *J* = 47.5 Hz, 1F).

FTIR (ATR, cm^{-1}): 3050, 2980, 1646, 1512, 1476, 1401, 1369, 1303, 1242, 1210, 1164, 1039, 912, 795, 727, 642, 569, 476.

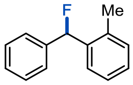

1-(fluoro(phenyl)methyl)-2-methylbenzene (9) was prepared from 2-benzyltoluene (274 µL, 1.50 mmol, 6.00 equiv) according to general procedure B. After reacting for 6 h, the reaction mixture was passed through a short plug of silica gel. The yield of the title compound was determined by ^{19}F NMR analysis of the crude reaction (43% NMR

yield, average of two runs; Run 1: 41% yield, Run 2: 45% yield). Characterization data is consistent with reported literature values.[19]

^{19}F NMR (282 MHz, $CDCl_3$): δ -166.9 (d, *J* = 47.1 Hz, 1F).

FTIR (ATR, cm^{-1}): 3061, 3024, 2920, 2854, 1695, 1601, 1493, 1451, 1400, 1379, 1269, 1106, 1073, 1049, 1029, 913, 765, 742, 725, 696, 615, 461, 440.

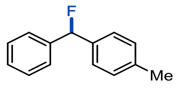

1-(fluoro(phenyl)methyl)-4-methylbenzene (10) was prepared from 1-methyl-4-(phenylmethyl)benzene (276 µL, 1.50 mmol, 6.00 equiv) according to general procedure B. After reacting for 6 h, the reaction mixture was passed through a short plug of silica gel. The yield of the title compound was determined by ^{19}F NMR analysis of the crude reaction (61% NMR yield, average of two runs; Run 1: 61% yield, Run 2: 60 % yield).

^{19}F NMR (282 MHz, $CDCl_3$): δ -165.6 (d, *J* = 47.6 Hz, 1F).

FTIR (ATR, cm^{-1}): 3060, 3024, 2919, 2854, 1658, 1604, 1513, 1493, 1452, 1377, 1276, 1180, 1107, 1073, 1029, 831, 788, 767, 721, 696, 595, 481.

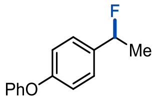

(1-fluoroethyl)-4-phenoxybenzene (11) was prepared from 1-ethyl-4-phenoxybenzene (287 µL, 1.50 mmol, 6.00 equiv) according to general procedure B. After reacting for 6 h, the reaction mixture was passed through a short plug of silica gel. The yield of the title compound was determined by ^{19}F NMR analysis of the crude reaction (52% NMR yield, average of two runs; Run 1: 55% yield, Run 2: 49% yield).

^{19}F NMR (282 MHz, $CDCl_3$): δ -163.6 (dq, *J* = 47.2, 23.5 Hz, 1F).

FTIR (ATR, cm^{-1}): 3030, 2963, 2929, 2871, 1589, 1504, 1487, 1455, 1231, 1165, 1108, 1071, 1015, 869, 834, 754, 690, 544, 505.

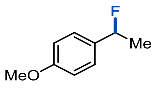

1-(1-fluoroethyl)-4-methoxybenzene (12) was prepared from *p*-ethylanisole (213 μL, 1.50 mmol, 6.00 equiv) according to general procedure B. After reacting for 6 h, the reaction mixture was passed through a short plug of silica gel. The yield of the title compound was determined by ^{19}F NMR analysis of the crude reaction (36% NMR yield, average of two runs; Run 1: 36% yield, Run 2: 36% yield). Characterization data is consistent with reported literature values.[20]

^{19}F NMR (282 MHz, $CDCl_3$): δ -161.4 (dq, *J* = 47.2, 23.5 Hz, 1F).

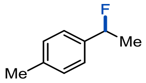

1-(1-fluoroethyl)-4-methylbenzene (13) was prepared from 4-ethyltoluene (209 mL, 1.50 mmol, 6.00 equiv) according to general procedure B. The title compound was isolated via preparatory thin-layer chromatography, eluting with 100% petroleum ether (44% NMR yield of **13**, 4% NMR yield of 1-ethyl-4-(fluoromethyl)benzene **S3,** average of two runs; Run 1: 43% yield, Run 2: 45% yield). Product **13** was isolated with **S3**. Characterization data is consistent with reported literature values.[20]

^{1}H NMR (500 MHz, $CDCl_3$): δ 7.25 – 7.15 (m, 4H), 5.60 (dq, *J* = 47.7, 6.4 Hz, 1H), 2.36 (s, 3H), 1.63 (dd, *J* = 23.8, 6.4 Hz, 3H).

^{13}C NMR (126 MHz, $CDCl_3$): δ 138.6 (d, *J* = 19.6 Hz), 138.2 (d, *J* = 2.0 Hz), 129.3, 125.5 (d, *J* = 6.6 Hz), 91.1 (d, *J* = 166.4 Hz), 23.0 (d, *J* = 25.5 Hz), 21.3.

^{19}F NMR (282 MHz, $CDCl_3$): δ -164.9 (dq, *J* = 47.5, 23.6 Hz, 1F).

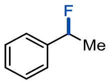

1-fluoroethylbenzene (14) was prepared from ethylbenzene (184 μL, 1.50 mmol, 6.00 equiv) according to general procedure B. The title compound was purified via preparatory thin-layer chromatography, eluting with 100% petroleum ether (38% NMR yield, average of two runs; Run 1: 35% yield, Run 2: 41% yield). Characterization data is consistent with reported literature values.[21]

^{1}H NMR (500 MHz, $CDCl_3$): δ 7.40 – 7.29 (m, 5H), 5.63 (dq, *J* = 47.6, 6.5 Hz, 1H), 1.65 (dd, *J* = 23.9, 6.4 Hz, 3H).

^{13}C NMR (126 MHz, CDCl$_3$): δ 141.6 (d, *J* = 19.5 Hz), 128.6, 128.4 (d, *J* = 2.1 Hz), 125.4 (d, *J* = 6.7 Hz), 91.1 (d, *J* = 167.3 Hz), 23.1 (d, *J* = 25.3 Hz).

^{19}F NMR (282 MHz, CDCl$_3$): δ -167.0 (dq, *J* = 47.8, 24.0 Hz, 1F).

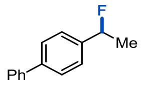

4-(1-fluoroethyl)-1,1'-biphenyl (15) was prepared from 4-ethylbiphenyl (273 mg, 1.5 mmol, 6.00 equiv) according to general procedure B. The title compound was isolated via preparatory thin-layer chromatography, eluting with 100% petroleum ether (65% NMR yield, average of two runs; Run 1: 60% yield, Run 2: 69% yield). Characterization data is consistent with reported literature values.[22–24]

^{1}H NMR (500 MHz, CDCl$_3$): δ 7.62 – 7.58 (m, 4H), 7.47 – 7.33 (m, 5H), 5.68 (dq, *J* = 47.7, 6.5 Hz, 1H), 1.69 (dd, *J* = 23.8, 6.4 Hz, 3H).

^{13}C NMR (126 MHz, CDCl$_3$): δ 141.4, 140.8, 140.5 (d, *J* = 19.8 Hz), 129.0, 127.6, 127.4, 127.3, 125.9 (d, *J* = 6.5 Hz), 91.0 (d, *J* = 167.4 Hz), 23.0 (d, *J* = 25.4 Hz).

^{19}F NMR (282 MHz, CDCl$_3$): δ -166.6 (dq, *J* = 47.7, 23.9 Hz, 1F).

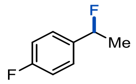

1-fluoro-4-(1-fluoroethyl)benzene (16) was prepared from 1-ethyl-4-fluorobenzene (186 µL, 1.50 mmol, 6.00 equiv) according to general procedure B. The title compound was purified via preparatory thin-layer chromatography, eluting with 100% petroleum ether (38% NMR yield, average of two runs; Run 1: 40% yield, Run 2: 36% yield). Characterization data is consistent with reported literature values.[22]

^{1}H NMR (500 MHz, CDCl$_3$): δ 7.37 – 7.30 (m, 2H), 7.06 (t, *J* = 8.5 Hz, 2H), 5.61 (dq, *J* = 47.5, 6.4 Hz, 1H), 1.63 (ddd, *J* = 23.8, 6.5, 1.2 Hz, 3H).

^{13}C NMR (126 MHz, CDCl$_3$): δ 162.7 (dd, *J* = 246.4, 2.4 Hz), 137.3 (dd, *J* = 20.0, 3.1 Hz), 127.3 (dd, *J* = 8.3, 6.4 Hz), 115.5 (d, *J* = 21.5 Hz), 90.5 (d, *J* = 167.4 Hz), 23.0 (d, *J* = 25.5 Hz).

^{19}F NMR (282 MHz, CDCl$_3$): δ -114.0 (m, 1F), -164.4 – -165.5 (m, 1F).

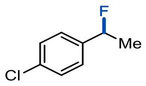

1-chloro-4-(1-fluoroethyl)benzene (17) was prepared from 1-chloro-4-ethylbenzene (202 µL, 1.50 mmol, 6.00 equiv) according to general procedure B. The title compound was purified via preparatory thin-layer chromatography, eluting with 100% petroleum ether (35% NMR yield, average of two runs; Run 1: 39% yield, Run 2: 30% yield). Characterization data is consistent with reported literature values.[22]

^{1}H NMR (500 MHz, $CDCl_3$): δ 7.36 – 7.30 (m, 4H), 5.60 (dq, *J* = 47.5, 6.4 Hz, 1H), 1.62 (dd, *J* = 23.9, 6.4 Hz, 3H).

^{13}C NMR (126 MHz, $CDCl_3$): δ 140.1 (d, *J* = 19.9 Hz), 134.1 (d, *J* = 2.4 Hz), 128.8, 126.8 (d, *J* = 6.7 Hz), 90.4 (d, *J* = 168.3 Hz), 23.0 (d, *J* = 25.2 Hz).

^{19}F NMR (282 MHz, $CDCl_3$): δ -167.5 (dq, *J* = 47.8, 23.8 Hz, 1F).

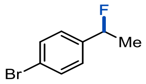

1-bromo-4-(1-fluoroethyl)benzene (18) was prepared from 1-bromo-4-ethylbenzene (207 µL, 1.50 mmol, 6.00 equiv) according to general procedure B. The title compound was purified via preparatory thin-layer chromatography, eluting with 100% petroleum ether (31% NMR yield, average of two runs; Run 1: 31% yield, Run 2: 30% yield). Characterization data is consistent with reported literature values.[25]

^{1}H NMR (500 MHz, $CDCl_3$): δ 7.50 (d, *J* = 8.1 Hz, 2H), 7.23 (d, *J* = 8.0 Hz, 2H), 5.58 (dq, *J* = 47.4, 6.4 Hz, 1H), 1.62 (dd, *J* = 23.9, 6.4 Hz, 3H).

^{13}C NMR (126 MHz, $CDCl_3$): δ 140.6 (d, *J* = 20.0 Hz), 131.8, 127.1 (d, *J* = 6.8 Hz), 122.2 (d, *J* = 2.5 Hz), 90.4 (d, *J* = 168.6 Hz), 23.0 (d, *J* = 25.1 Hz).

^{19}F NMR (282 MHz, $CDCl_3$): δ -168.1 (dq, *J* = 47.6, 23.8 Hz, 1F).

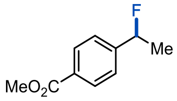

methyl-4-(1-fluoroethyl)benzoate (19) was prepared from 4-ethyl-benzoic acid methyl ester (237 µL, 1.50 mmol, 6.00 equiv) according to general procedure B. The title compound was purified via preparatory thin-layer chromatography, eluting with 100% petroleum ether (21% NMR yield, average of two runs; Run 1: 22% yield, Run 2: 19% yield). Characterization data is consistent with reported literature values.[6]

^{1}H NMR (500 MHz, CDCl$_3$): δ 8.08 – 8.02 (m, 2H), 7.44 – 7.39 (m, 2H), 5.68 (dq, *J* = 47.5, 6.4 Hz, 1H), 3.92 (s, 3H), 1.65 (dd, *J* = 24.0, 6.5 Hz, 3H).

^{13}C NMR (126 MHz, CDCl$_3$): δ 166.8, 146.4, 129.8, 126.0, 124.9 (d, *J* = 7.4 Hz), 90.4 (d, *J* = 169.5 Hz), 52.2, 23.0 (d, *J* = 24.8 Hz).

^{19}F NMR (282 MHz, CDCl$_3$): δ -171.3 (dq, *J* = 47.8, 23.9 Hz, 1F).

FTIR (ATR, cm^{-1}): 2978, 2878, 1720, 1611, 1461, 1435, 1370, 1277, 1179, 1108, 1020, 855, 734, 703.

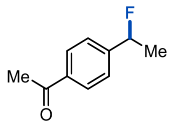

1-(4-(1-fluoroethyl)phenyl)ethanone (20) was prepared from 4′-ethylacetophenone (224 mL, 1.50 mmol, 6.00 equiv) according to general procedure B. The title compound was isolated via preparatory thin-layer chromatography, eluting with 50% diethyl ether/petroleum ether (14% NMR yield, average of two runs; Run 1: 14% yield, Run 2: 14% yield). Characterization data is consistent with reported literature values.[22]

^{1}H NMR (500 MHz, CDCl$_3$): δ 7.98 (d, *J* = 8.3 Hz, 2H), 7.44 (d, *J* = 7.8 Hz, 2H), 5.68 (dd, *J* = 47.4, 6.4 Hz, 1H), 2.61 (s, 3H), 1.65 (dd, *J* = 24.0, 6.5 Hz, 3H).

^{13}C NMR (126 MHz, CDCl$_3$): δ 197.8, 146.8 (d, *J* = 19.6 Hz), 137.0 (d, *J* = 1.7 Hz), 128.8, 125.2 (d, *J* = 7.4 Hz), 90.5 (d, *J* = 169.8 Hz), 26.8, 23.2 (d, *J* = 24.7 Hz).

^{19}F NMR (282 MHz, CDCl$_3$): δ -171.3 (dq, *J* = 48.1, 24.0 Hz, 1F).

FTIR (ATR, cm^{-1}): 2980, 2878, 1653, 913, 856, 744, 689, 671, 635.

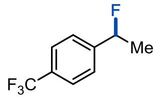

1-(1-fluoroethyl)-4-(trifluoromethyl)benzene (21) was prepared from 1-ethyl-4-(trifluoromethyl)benzene (261 mg, 1.50 mmol, 6.00 equiv) according to general procedure B. The title compound was purified via preparatory thin-layer chromatography, eluting with 100% petroleum ether (11% NMR yield, average of two runs; Run 1: 11% yield, Run 2: 10% yield). Characterization data is consistent with reported literature values.[24]

^{19}F NMR (282 MHz, CDCl$_3$): δ -62.6 (d, *J* = 1.1 Hz, 3F), -171.2 (dq, *J* = 47.7, 23.8 Hz, 1F).

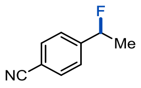

4-(1-fluoroethyl)benzonitrile (22) was prepared from 4-ethylbenzonitrile (206 μL, 1.50 mmol, 6.00 equiv) according to general procedure B. The title compound was purified via preparatory thin-layer chromatography, eluting with 100% petroleum ether (11% NMR yield, average of two runs; Run 1: 11% yield, Run 2: 11% yield). Characterization data is consistent with reported literature values.[22]

^{19}F NMR (282 MHz, $CDCl_3$): δ -172.7 (dq, *J* = 47.9, 23.9 Hz, 1F).

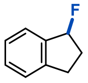

1-fluoroindane (23) was prepared from indane (184 μL, 1.50 mmol, 6.00 equiv) according to general procedure B. After reacting for 6 h, the reaction mixture was passed through a short plug of silica gel. The yield of the title compound was determined by ^{19}F NMR analysis of the crude reaction (68% NMR yield, average of two runs; Run 1: 69% yield, Run 2: 66% yield). Characterization data is consistent with reported literature values.[7]

^{19}F NMR (282 MHz, $CDCl_3$): δ -159.4 – -160.5 (m, 1F).

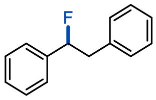

(1-fluoroethane-1,2-diyl)dibenzene (24) was prepared from bibenzyl (273 mg, 1.50 mmol, 6.00 equiv) according to general procedure B. The title compound was isolated via preparatory thin-layer chromatography, eluting with 100% petroleum ether (41% NMR yield, average of two runs; Run 1: 41% yield, Run 2: 41% yield). Characterization data is consistent with reported literature values.[25,26]

^{1}H NMR (500 MHz, $CDCl_3$): δ 7.39 – 7.27 (m, 7H), 7.25 – 7.16 (m, 3H), 5.62 (ddd, *J* = 47.4, 8.0, 4.9 Hz, 1H), 3.34 – 3.03 (m, 2H).

^{13}C NMR (126 MHz, $CDCl_3$): δ 139.9 (d, *J* = 19.9 Hz), 136.8 (d, *J* = 4.0 Hz), 129.7, 128.5, 128.5, 126.8, 125.8 (d, *J* = 6.7 Hz), 95.0 (d, *J* = 174.0 Hz), 44.1 (d, *J* = 24.4 Hz). Carbon shifts at 128.5 overlap in the reported spectrum.

^{19}F NMR (282 MHz, $CDCl_3$): δ -173.2 (ddd, *J* = 46.6, 28.4, 17.6 Hz, 1F).

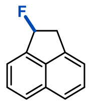

Flouroacenapthene (25) was prepared from acenapthene (116 mg, 0.75 mmol, 3.00 equiv) according to general procedure B. After reacting for 6 h, the reaction mixture was passed through a short plug of silica gel. The yield of the title compound was determined by ^{19}F NMR analysis of the crude reaction (29% NMR yield, average of two runs; Run 1: 29% yield, Run 2: 29% yield). Characterization data is consistent with reported literature values.[27]

^{19}F NMR (282 MHz, $CDCl_3$): δ -159.4 (ddd, *J* = 53.5, 29.8, 20.9 Hz, 1F).

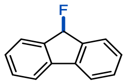

9-fluoro-9*H*-fluorene (26) was prepared from fluorene (125 mg, 0.75 mmol, 3.00 equiv) according to general procedure B. The title compound was isolated via preparatory thin-layer chromatography, eluting with 100% petroleum ether (29% NMR yield, average of two runs; Run 1: 29% yield, Run 2: 29% yield). Characterization data is consistent with reported literature values.[28,29]

^{1}H NMR (500 MHz, $CDCl_3$): δ 7.64 (m, 4H), 7.43 (t, *J* = 7.5 Hz, 2H), 7.32 (t, *J* = 7.5 Hz, 2H), 6.31 (d, *J* = 54.3 Hz, 1H).

^{13}C NMR (126 MHz, $CDCl_3$): δ 141.0, 141.0, 130.4 (d, *J* = 2.5 Hz), 128.2 (d, *J* = 2.2 Hz), 126.0 (d, *J* = 1.3 Hz), 120.3, 92.7 (d, *J* = 178.4 Hz). Carbon shifts at 141.0 overlap in the reported spectrum.

^{19}F NMR (282 MHz, $CDCl_3$): δ -187.0 (d, *J* = 54.3 Hz, 1F).

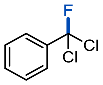

(dichlorofluoromethyl)benzene (27) was prepared from benzal chloride (193 μL, 1.50 mmol, 6.00 equiv) according to general procedure B. After reacting for 6 h, the reaction mixture was passed through a short plug of silica gel. The yield of the title compound was determined by ^{19}F NMR analysis of the crude reaction (27% NMR yield, average of two runs; Run 1: 27% yield, Run 2: 26% yield). Characterization data is consistent with reported literature values.[30]

^{19}F NMR (282 MHz, $CDCl_3$): δ -52.8 (s, 1F).

FTIR (ATR, cm^{-1}): 2980, 1477, 1459, 1370, 1242, 1210, 911, 729, 700.

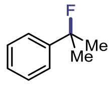

α,α-dimethylbenzyl fluoride (28) was prepared from cumene (209 μL, 1.50 mmol, 6.00 equiv) according to general procedure B. The title compound was purified via preparatory thin-layer chromatography, eluting with 100% petroleum ether (58% NMR yield, average of two runs; Run 1: 59% yield, Run 2: 57% yield). Characterization data is consistent with reported literature values.[31]

^{1}H NMR (500 MHz, CDCl$_3$): δ 7.41 – 7.31 (m, 4H), 7.30 – 7.27 (m, 1H), 1.67 (d, *J* = 22.2 Hz, 6H).

^{13}C NMR (126 MHz, CDCl$_3$): δ 145.9, 128.3, 127.2 (d, *J* = 1.5 Hz), 123.8 (d, *J* = 8.9 Hz), 95.7 (d, *J* = 168.8 Hz), 29.3 (d, *J* = 25.8 Hz).

^{19}F NMR (282 MHz, CDCl$_3$): δ -137.4 (hept, *J* = 21.8 Hz, 1F).

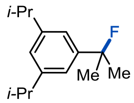

1-(2-fluoropropan-2-yl)-3,5-diisopropylbenzene (29) was prepared from 1,3,5-triisopropyl benzene (306 mg, 1.50 mmol, 6.00 equiv) according to general procedure B. The title compound was isolated via preparatory thin-layer chromatography, eluting with 100% petroleum ether (55% NMR yield, average of two runs; Run 1: 58% yield, Run 2: 51% yield).

^{1}H NMR (500 MHz, CDCl$_3$): δ 7.08 (d, *J* = 1.8 Hz, 2H), 7.03 (d, *J* = 1.7 Hz, 1H), 2.92 (hept, *J* = 7.0 Hz, 2H), 1.71 (d, *J* = 22.1 Hz, 6H), 1.28 (d, *J* = 6.7 Hz, 12H).

^{13}C NMR (126 MHz, CDCl$_3$): δ 148.9, 145.8 (d, *J* = 21.4 Hz), 123.7 (d, *J* = 1.6 Hz), 119.6 (d, *J* = 8.6 Hz), 96.2 (d, *J* = 168.4 Hz), 34.5, 29.6 (d, *J* = 25.8 Hz), 24.2.

^{19}F NMR (282 MHz, CDCl$_3$): δ -136.1 (hept, *J* = 22.3 Hz, 1F).

FTIR (ATR, cm^{-1}): 2925, 2852, 2007, 913, 743, 636.

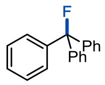

Fluorotriphenylmethane (30) was prepared from triphenylmethane (183 mg, 0.750 mmol, 3.00 equiv) according to general procedure B. After reacting for 6 h, the reaction mixture was passed through a short plug of silica gel. The yield of the title compound was determined by ^{19}F NMR analysis of the crude reaction (95% NMR yield, average of two runs; Run 1: 93% yield, Run 2: 97% yield). Characterization data is consistent with reported literature values.[29,32,33]

^{19}F NMR (282 MHz, $CDCl_3$): δ -125.9 (s, 1F).

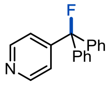

4-(fluorodiphenylmethyl)pyridine (31) was prepared from diphenyl-4-pyridylmethane (368 mg, 1.50 mmol, 6.00 equiv) according to general procedure B. After reacting for 6 h, the reaction mixture was passed through a short plug of silica gel. The yield of the title compound was determined by ^{19}F NMR analysis of the crude reaction (63% NMR yield, average of two runs; Run 1: 61% yield, Run 2: 64% yield).

^{19}F NMR (282 MHz, $CDCl_3$): δ -130.2 (s, 1F).

FTIR (ATR, cm^{-1}): 3059, 3026, 2977, 1724, 1591, 1557, 1494, 1448, 1413, 1370, 1301, 1070, 1031, 993, 732, 698, 639, 604.

HRMS: (ESI-TOF) calculated for $C_{18}H_{15}FN^{+}$ ($[M+H]^{+}$): 264.1183, found 264.1183.

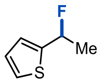

1-fluoro-ethylthiophene (32) was prepared from 2-ethylthiophene (170 μL, 1.50 mmol, 6.00 equiv) according to general procedure B. After reacting for 6 h, the reaction mixture was passed through a short plug of silica gel. The yield of the title compound was determined by ^{19}F NMR analysis of the crude reaction (47% NMR yield, average of two runs; Run 1: 47% yield, Run 2: 46% yield).

^{19}F NMR (282 MHz, $CDCl_3$): δ -148.5 – -149.5 (m, 1F).

FTIR (ATR, cm^{-1}): 2967, 2928, 2872, 2853, 1723, 1452, 1374, 1307, 1234, 913, 850, 804, 743, 694.

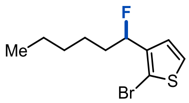

2-bromo-3-(1-fluorohexyl)thiophene (33) was prepared from 3-hexyl-2-bromothiophene (299 µL, 1.50 mmol, 6.00 equiv) according to general procedure B. The title compound was isolated via preparatory thin-layer chromatography (eluting with 5% EtOAc in hexane) to produce a clear oil (20% NMR yield, average of two runs; Run 1: 19% yield, Run 2: 20% yield). Characterization data is consistent with reported literature values.[25]

^{1}H NMR (500 MHz, $CDCl_3$): δ 7.28 (d, *J* = 5.7 Hz, 1H), 7.00 (d, *J* = 5.7 Hz, 1H), 5.52 (ddd, *J* = 47.2, 8.0, 5.3 Hz, 1H), 2.05 – 1.92 (m, 1H), 1.78 (m, 1H), 1.50 – 1.43 (m, 1H), 1.36 – 1.28 (m, 5H), 0.92 – 0.86 (m, 3H).

^{13}C NMR (126 MHz, $CDCl_3$): δ 140.3 (d, *J* = 23.0 Hz), 126.6, 125.8 (d, *J* = 3.2 Hz), 110.7 (d, *J* = 8.5 Hz), 89.9 (d, *J* = 168.4 Hz), 35.9 (d, *J* = 23.8 Hz), 31.6, 24.7 (d, *J* = 4.3 Hz), 22.6, 14.1.

^{19}F NMR (282 MHz, $CDCl_3$): δ -172.2 (ddd, *J* = 47.0, 27.6, 15.7 Hz, 1F).

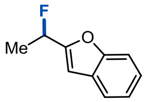

2-(1-fluoroethyl)benzofuran (34) was prepared from 2-ethylbenzofuran (219 mg, 1.50 mmol, 6.00 equiv) according to general procedure B. The title compound was isolated via preparatory thin-layer chromatography (eluting with 5% EtOAc in hexane) to produce a clear oil (71% NMR yield, average of two runs; Run 1: 78% yield, Run 2: 71% yield). The title compound was isolated and characterized with hydrolysis product**,** which was formed under isolation conditions.

^{1}H NMR (500 MHz, $CDCl_3$): δ 7.58 (dd, *J* = 7.7, 1.2 Hz, 1H), 7.52 – 7.48 (m, 1H), 7.34 – 7.29 (m, 1H), 7.26 – 7.21 (m, 1H), 6.77 (d, *J* = 3.8 Hz, 1H), 5.73 (dq, *J* = 48.3, 6.6 Hz, 1H), 1.80 (dd, *J* = 22.9, 6.6 Hz, 3H).

^{13}C NMR (126 MHz, $CDCl_3$): δ 160.2, 155.3 (d, *J* = 20.5 Hz), 127.6 (d, *J* = 2.1 Hz), 125.0 (d, *J* = 1.5 Hz), 123.0 (d, *J* = 0.7 Hz), 121.5 (d, *J* = 1.6 Hz), 111.5, 104.7 (d, *J* = 6.0 Hz), 84.0 (d, *J* = 165.4 Hz), 19.1 (d, *J* = 24.6 Hz).

^{19}F NMR (282 MHz, $CDCl_3$): δ -165.8 – -166.2 (m, 1F).

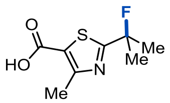

2-(2-fluoropropan-2-yl)-4-methylthiazole-5-carboxylic acid (febuxostat derivative, 35) was prepared from 2-isopropyl-4-methyl-1,3-thiazole-5-carboxylic acid (139 mg, 0.750 mmol, 3.00 equiv) according to general procedure B. After reacting for 6 h, the reaction mixture was passed through a short plug of silica gel. The yield of the title compound was determined by ^{19}F NMR analysis of the crude reaction (43% NMR yield, average of two runs; Run 1: 41% yield, Run 2: 47% yield).

^{19}F NMR (282 MHz, $CDCl_3$): δ -130.5 – -131.2 (m, 1F).

FTIR (ATR, cm^{-1}): 2979, 1691, 1535, 1472, 1386, 1316, 1242, 1096, 1039, 1019, 911, 726, 518, 476, 447.

HRMS: (ESI-TOF) calculated for $C_8H_{11}FNO_2S^+$ ([M+H]$^+$): 204.0489, found 204. 0494.

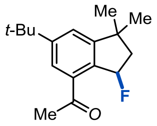

1-(6-(*tert*-butyl)-3-fluoro-1,1-dimethyl-2,3-dihydro-1H-inden-4-yl)ethan-1-one (celestolide derivative, 36) was prepared from celestolide (367 mg, 1.50 mmol, 6.00 equiv) according to general procedure B. The title compound was isolated via preparatory thin-layer chromatography, eluting with 5% EtOAc in hexane (68% NMR yield, average of two runs; Run 1: 65% yield, Run 2: 70% yield). Characterization data is consistent with reported literature values.[25]

^{1}H NMR (500 MHz, $CDCl_3$): δ 7.76 (d, *J* = 1.8 Hz, 1H), 7.42 (t, *J* = 1.5 Hz, 1H), 6.44 (ddd, *J* = 54.0, 6.1, 1.4 Hz, 1H), 2.66 (s, 3H), 2.30 (ddd, *J* = 23.3, 14.7, 1.4 Hz, 1H), 2.19 (ddd, *J* = 34.1, 14.7, 6.1 Hz, 1H), 1.37 (m, 12H), 1.34 (s, 3H).

^{13}C NMR (126 MHz, $CDCl_3$): δ 199.8, 156.0 (d, *J* = 4.7 Hz), 154.3 (d, *J* = 3.7 Hz), 135.2 (d, *J* = 16.4 Hz), 135.0, 125.7 (d, *J* = 2.7 Hz), 123.7 (d, *J* = 2.6 Hz), 93.9 (d, *J* = 172.4 Hz), 48.5 (d, *J* = 22.3 Hz), 42.9 (d, *J* = 1.7 Hz), 35.3, 31.7 (d, *J* = 2.1 Hz), 31.5, 29.2 (d, *J* = 4.7 Hz), 28.8 (d, *J* = 1.3 Hz).

^{19}F NMR (282 MHz, $CDCl_3$): δ -158.6 (ddd, *J* = 54.7, 33.7, 23.7 Hz, 1F).

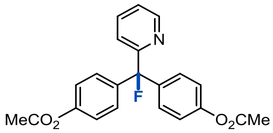

(fluoro(pyridin-2-yl)methylene)bis(4,1-phenylene) diacetate (bisacodyl derivative, 37) was prepared from bisacodyl (271 mg, 0.75 mmol, 3.00 equiv) according to general procedure B. After reacting for 6 h, the reaction mixture was passed through a short plug of silica gel. The yield of the title compound was determined by ^{19}F NMR analysis of the crude reaction (49% NMR yield, average of two runs; Run 1: 52% yield, Run 2: 45% yield).

^{19}F NMR (282 MHz, $CDCl_3$): δ -133.8 (s, 1F).

FTIR (ATR, cm^{-1}): 2980, 1755, 1588, 1504, 1477, 1462, 1370, 1201, 1166, 1034, 1018, 911, 727, 645, 476.

HRMS: (ESI-TOF) calculated for $C_{22}H_{19}FNO_4{}^+$ ($[M+H]^+$): 380.1292, found 380.1292.

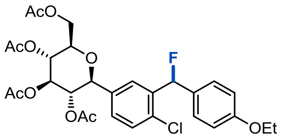

(2*R*,3*R*,4*R*,5*S*,6*S*)-2-(acetoxymethyl)-6-(4-chloro-3-((4-ethoxyphenyl)fluoromethyl)phenyl)tetrahydro-2*H*-pyran-3,4,5-triyl triacetate (Dapagliflozin derivative, 38) was prepared from (2*R*,3*R*,4*R*,5*S*,6*S*)-2-(acetoxymethyl)-6-(4-chloro-3-(4-ethoxybenzyl)phenyl)tetrahydro-2*H*-pyran-3,4,5-triyl triacetate (130 mg, 0.225 mmol, 3.00 equiv) according to general procedure B with the following deviations. The reaction was performed using Ir(*p*-CF_3-ppy)$_3$ as photocatalyst and benzene as solvent. After reacting for 6 h, the reaction mixture was passed through a short plug of Florisil. The yield of the title compound was determined by ^{19}F NMR analysis of the crude reaction (44% NMR yield, 1.6:1 dr). Characterization data is consistent with reported literature values.[34]

^{19}F NMR (376 MHz, $CDCl_3$): major diastereomer δ -163.7 (d, J = 47.7 Hz, 1F), minor diastereomer δ -165.7 (d, J = 47.8 Hz, 1F).

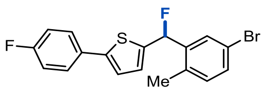

2-((5-bromo-2-methylphenyl)fluoromethyl)-5-(4-fluorophenyl)thiophene (39) was prepared from 2-(5-bromo-2-methylbenzyl)-5-(4-fluorophenyl)thiophene (163 mg, 0.45 mmol, 3.00 equiv) according to general procedure B with the following deviations. The

reaction was performed using Ir(*p*-CF_3-ppy)$_3$ as photocatalyst and benzene as solvent. After reacting for 6 h, the yield of the title compound was determined by ^{19}F NMR analysis of the crude reaction (34% NMR yield).

^{19}F NMR (376 MHz, $CDCl_3$): δ -113.5 – -113.6 (m, 1F), -154.9 (d, *J* = 48.0 Hz, 1F).

HRMS: (ESI-TOF) calculated for $C_{18}H_{14}BrF_2S^+$ ($[M+H]^+$): 378.9962, found 378.9959.

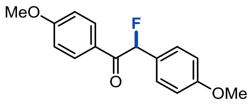

2-fluoro-1,2-bis(4-methoxyphenyl)ethan-1-one (40) was prepared from desoxyanisoin (192 mg, 0.750 mmol, 3.00 equiv) according to general procedure B. After reacting for 6 h, the reaction mixture was passed through a short plug of silica gel. The title compound was purified via preparatory thin-layer chromatography (eluting with 20% ethyl acetate in hexane) to produce a white solid (44% NMR yield). Characterization data is consistent with reported literature values.[35]

^{1}H NMR (500 MHz, $CDCl_3$): δ 7.92 (d, *J* = 9.0 Hz, 2H), 7.40 (dd, *J* = 8.7, 1.9 Hz, 2H), 6.89 (t, *J* = 9.3 Hz, 4H), 6.45 (d, *J* = 49.0 Hz, 1H), 3.84 (s, 3H), 3.79 (s, 3H).

^{13}C NMR (126 MHz, $CDCl_3$): δ 192.8 (d, *J* = 21.4 Hz), 164.0, 160.7 (d, *J* = 2.7 Hz), 131.5, 129.5 (d, *J* = 4.7 Hz), 127.1, 126.8 (d, *J* = 20.6 Hz), 114.6 (d, *J* = 1.5 Hz), 114.0, 93.4 (d, *J* = 184.3 Hz), 55.6, 55.5.

^{19}F NMR (376 MHz, $CDCl_3$): δ -171.2 (d, *J* = 48.9 Hz, 1F).

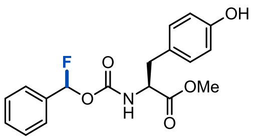

methyl ((fluoro(phenyl)methoxy)carbonyl)-L-tyrosinate (41) was prepared from *N*-benzyloxycarbonyl-L-tyrosine methyl ester (296 mg, 0.90 mmol, 6.00 equiv) according to general procedure B. After reacting for 6 h, the reaction mixture was passed through a short plug of silica gel. The yield of the title compound was determined by ^{19}F NMR analysis of the crude reaction (13% NMR yield, 1:1 dr).

^{19}F NMR (376 MHz, $CDCl_3$): δ -120.3 (d, *J* = 49.1 Hz, 1F), -120.5 (d, *J* = 48.6 Hz, 1F).

FTIR (ATR, cm^{-1}): 3333, 2980, 1720, 1615, 1516, 1477, 1460, 1370, 1210, 1175, 1058, 909, 727, 647.

HRMS: (ESI-TOF) calculated for $C_{18}H_{19}FNO_5^+$ ($[M+H]^+$): 348.1241, found 348.1236.

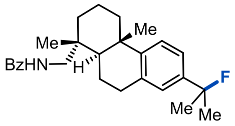

N-(((1R,4aS,10aR)-7-(2-fluoropropan-2-yl)-1,4a-dimethyl-1,2,3,4,4a,9,10,10a-octahydrophenanthren-1-yl)methyl)benzamide (42) was prepared from N-(((1R,4aS)-7-isopropyl-1,4a-dimethyl-1,2,3,4,4a,9,10,10a-octahydrophenanthren-1-yl)methyl)benzamide (175 mg, 0.45 mmol, 3.00 equiv) according to general procedure B with the following deviations. The reaction was performed using Ir(*p*-CF_3-ppy)$_3$ as photocatalyst and 1,2-difluorobenzene as solvent. After reacting for 6 h, the reaction mixture was passed through a short plug of silica gel. The yield of the title compound was determined by ^{19}F NMR analysis of the crude reaction (20% NMR yield, 2.3:1 rr).

^{19}F NMR (376 MHz, $CDCl_3$): δ -135.8 (hept, *J* = 21.9 Hz, 1F).

FTIR (ATR, cm^{-1}): 3359, 2980, 1731, 1466, 1374, 1246, 1095, 1045, 905.

HRMS: (ESI-TOF) calculated for $C_{27}H_{35}FNO^+$ ($[M+H]^+$): 408.2697, found 408.2695.

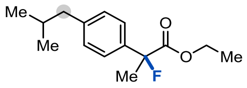

Ethyl 2-fluoro-2-(4-isobutylphenyl)propanoate (ibuprofen ethyl ester, 43) was prepared from ibuprofen ethyl ester (176 mg, 0.75 mmol, 3.00 equiv) according to general procedure B. The title compound was isolated via preparatory thin-layer chromatography (eluting with 10% EtOAc in hexane) to produce a clear oil (34% NMR yield, 2.4:1 rr, average of two runs; Run 1: 34% yield, Run 2: 33% yield). Minor regioisomer **62** characterization data is consistent with reported literature values.[25]

^{19}F NMR (282 MHz, $CDCl_3$): δ -150.2 (q, *J* = 22.1 Hz, 1F).

FTIR (ATR, cm^{-1}): 2934, 2853, 1758, 1651, 1456, 1425, 1241, 913, 818, 744, 664.

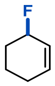

3-fluorocyclohex-1-ene (44) was prepared from cyclohexene (152 μL, 1.50 mmol, 6.00 equiv) according to general procedure B. After reacting for 6 h, the reaction mixture was passed through a short plug of silica gel. The yield of the title compound was determined by ^{19}F NMR analysis of the crude reaction (55% NMR yield, average of two

runs; Run 1: 56% yield, Run 2: 53% yield). Characterization data is consistent with reported literature values.[36]

^{19}F NMR (282 MHz, $CDCl_3$): δ -164.8 – -165.8 (m, 1F).

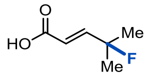

(E)-4-fluoro-4-methylpent-2-enoic acid (45) was prepared from 4-methyl-pent-2-enoic acid (171 mg, 1.50 mmol, 6.00 equiv) according to general procedure B. After reacting for 6 h, the reaction mixture was passed through a short plug of silica gel. The yield of the title compound was determined by ^{19}F NMR analysis of the crude reaction (14% NMR yield). Characterization data is consistent with reported literature values.[37]

^{19}F NMR (282 MHz, $CDCl_3$): δ -142.2 – -142.6 (m, 1F).

FTIR (ATR, cm^{-1}): 3076 (b), 2964, 2872, 1792, 1693, 1651, 1476, 1416, 1369, 1302, 1280, 1212, 1163, 985, 965, 867, 721, 699, 663, 476.

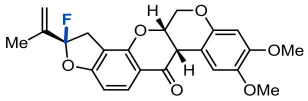

Rotenone derivative (46) was prepared from rotenone (296 mg, 0.75 mmol, 3.00 equiv) according to general procedure B. After reacting for 6 h, the reaction mixture was passed through a short plug of silica gel. The yield of the title compound was determined by ^{19}F NMR analysis of the crude reaction (33% NMR yield, 4.5:1 dr, average of two runs; Run 1: 33% yield, Run 2: 33% yield).

^{19}F NMR (282 MHz, $CDCl_3$): major diastereomer δ -132.2 (t, J = 6.1 Hz, 1F).

FTIR (ATR, cm^{-1}): 3483, 3305, 2978, 2950, 1729, 1679, 1607, 1510, 1457, 1436, 1346, 1298, 1215, 1177, 1038, 910, 815, 730, 712, 680, 644, 476.

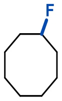

Fluorocyclooctane (47) was prepared from cyclooctane (101 μL, 0.750 mmol, 3.00 equiv) according to general procedure B with the following deviations. The reaction was performed with a 20 mol % n-Bu_4NPF_6 additive. After reacting for 6 h, the reaction mixture was passed through a short plug of silica gel. The yield of the title compound was determined by ^{19}F NMR analysis of the crude reaction (15% NMR yield, average of

two runs; Run 1: 17% yield, Run 2: 13% yield). Characterization data is consistent with reported literature values.[20]

^{19}F NMR (282 MHz, $CDCl_3$): δ -159.3 (m, 1F).

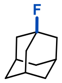

1-fluoroadamantane (48) was prepared from adamantane (204 mg, 1.50 mmol, 6.00 equiv) according to general procedure B with the following deviations. The reaction was performed using Ir(*p*-CF_3-ppy)$_3$ as photocatalyst, 1,2-difluorobenzene as solvent, and abstractor **3**. After reacting for 6 h, the reaction mixture was passed through a short plug of silica gel. The yield of the title compound was determined by ^{19}F NMR analysis of the crude reaction (15% NMR yield, 14:1 rr, average of two runs; Run 1: 16% yield, Run 2: 14% yield). Characterization data is consistent with reported literature values.[17]

^{1}H NMR (500 MHz, $CDCl_3$): δ 2.26 (s, 3H), 1.81 – 2.03 (m, 6H), 1.67 (s, 6H).

^{13}C NMR (126 MHz, $CDCl_3$): δ 92.6 (d, J = 183.4 Hz), 42.9 (d, J = 17.1 Hz), 36.0 (d, J = 2.1 Hz), 31.7 (d, J = 9.6 Hz).

^{19}F NMR (282 MHz, $CDCl_3$): δ -138.3 – -138.6 (m, 1F).

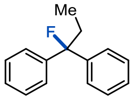

(1-fluoropropane-1,1-diyl)dibenzene (65) was prepared from 1,1-diphenylethylene (265 μL, 1.50 mmol, 6.00 equiv) according to general procedure B. After reacting for 6 h, the reaction mixture was passed through a short plug of silica gel. The yield of the title compound was determined by ^{19}F NMR analysis of the crude reaction (98% NMR yield). Characterization data is consistent with reported literature values.[38]

^{19}F NMR (282 MHz, $CDCl_3$): δ -152.4 (t, J = 24.3 Hz, 1F).

V. Characterization of Difluorination Scope

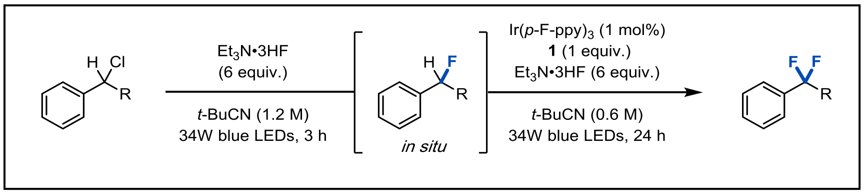

General procedure C for difluorination scope:

Reagent handling: Ir(*p*-F-ppy)$_3$ was stored in a Drierite-containing desiccator and weighed out on the bench. Phthalimide abstractor **1** was prepared according to literature procedure,[8] dried under vacuum for 48 h, stored in a Drierite-containing desiccator, and weighed out on the bench. Triethylamine trihydrofluoride, in a PTFE plastic vial, was degassed under nitrogen for 1 h and brought into the glovebox without further purification. Anhydrous pivalonitrile was passed through a plug a neutral alumina, degassed under nitrogen for 1 h, and stored in the glovebox over 4Å molecular sieves.

Reaction setup (0.250 mmol scale):
In situ generation of fluorinated C(sp^3)–H partner. To a 1-dram oven-dried vial, equipped with a Teflon stir bar, was added the chlorinated C(sp^3)–H partner (1.50 mmol, 6.00 equiv), triethylamine trihydrogen fluoride (245 μL, 1.50 mmol, 6.00 equiv), and pivalonitrile (208 μL) in a nitrogen-filled glovebox. The vial was capped, removed from the glovebox and sealed with electrical tape prior to irradiation. The reaction was stirred at 400 rpm for 3 h while illuminating with three 34W blue LED lamps (Kessil KSH150B) and two cooling fans (**Supplementary Figure 1**). After 3 h the vial was pumped back into the glovebox for further use. Note: fluorination does occur without illumination, albeit at a significantly slower rate, likely due to the absence of heat provided by the lamps.

Fluorination of monofluorinated C(sp^3) partner. To a separate 1-dram oven-dried vial, equipped with a Teflon stir bar, was added Ir(*p*-F-ppy)$_3$ (1.8 mg, 2.5 μmol, 1.0 mol %) and phthalimide-derived abstractor **1** (51.3 mg, 0.25 mmol, 1.00 equiv). The vial containing photocatalyst and phthalimide abstractor **1** was then covered with a Kimwipe and pumped into a nitrogen-filled glovebox. To the reaction vial was added the solution of *in situ* generated monofluorinated C(sp^3)–H partner (1.50 mmol, 6.00 equiv), rinsing with pivalonitrile (209 μL). Triethylamine trihydrofluoride (245 μL, 1.50 mmol, 6.00 equiv) was then added to the mixture. The vial was capped, removed from the glovebox and sealed with electrical tape prior to irradiation. The reaction was stirred at 800 rpm for 24 h while illuminating with three 34W blue LED lamps (Kessil KSH150B) and two cooling fans (**Supplementary Figure 1**). The crude reaction mixture was passed through a short pad of silica, eluting with $CDCl_3$, and analyzed by ^{19}F NMR relative to 1-fluoronapthalene (32.3 μL, 0.250 mmol, 1.00 equiv) as an external standard.

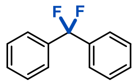

Difluorodiphenylmethane (49) was prepared from chlorodiphenylmethane (304 mg, 1.50 mmol, 6.00 equiv) according to general procedure C. The title compound was purified via preparatory thin-layer chromatography (eluting with 100% petroleum ether) to produce a clear oil (63% NMR yield, average of two runs; Run 1: 62% yield, Run 2: 63% yield). Characterization data is consistent with reported literature values.[39]

^{1}H NMR (500 MHz, $CDCl_3$): δ 7.55 – 7.46 (m, 4H), 7.47 – 7.37 (m, 6H).

^{13}C NMR (126 MHz, $CDCl_3$): δ 137.8 (t, *J* = 28.3 Hz), 130.0 (t, *J* = 1.9 Hz), 128.5, 125.9 (t, *J* = 5.6 Hz), 120.8 (t, *J* = 241.7 Hz).

^{19}F NMR (282 MHz, $CDCl_3$): δ -88.8 (s, 2F).

FTIR (ATR, cm^{-1}): 2923, 1452, 1272, 1222, 1053, 1026, 955, 769, 696, 647.

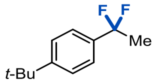

1-(*tert*-butyl)-4-(1,1-difluoroethyl)benzene (50) was prepared from 1-(*tert*-butyl)-4-(1-chloroethyl)benzene (295 mg, 1.50 mmol, 6.00 equiv) according to general procedure C. The title compound was purified via preparatory thin-layer chromatography (eluting with 100% petroleum ether) to produce a clear oil (29% NMR yield, average of two runs; Run 1: 30% yield, Run 2: 28% yield).

^{1}H NMR (500 MHz, $CDCl_3$): δ 7.44 (s, 4H), 1.92 (t, *J* = 18.1 Hz, 3H), 1.33 (s, 9H).

^{13}C NMR (126 MHz, $CDCl_3$): δ 135.4, 125.5, 124.5 (t, *J* = 5.9 Hz), 123.7 (t, *J* = 8.0 Hz), 122.1, 34.9, 31.4, 26.0 (t, *J* = 30.1 Hz). Carbon shift at 122.1 is a triplet however the baseline obscures the splitting pattern in the reported spectrum.

^{19}F NMR (282 MHz, $CDCl_3$): δ -86.8 (q, *J* = 18.2 Hz, 2F).

FTIR (ATR, cm^{-1}): 3470, 3071, 2979, 2949, 1638, 1475, 1444, 1397, 1365, 1303, 1172, 1071, 1035, 877, 785, 720, 644, 473, 447.

Monofluorination and difluorination comparison.

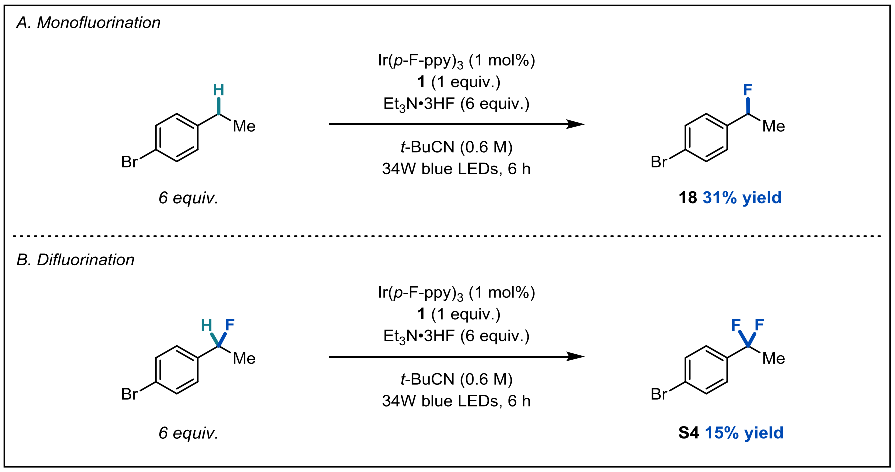

Supplementary Figure 13. A. C(sp^3)–H fluorination of 1-bromo-4-ethylbenzene. B. C(sp^3)–H fluorination of 1-bromo-4-(1-fluoroethyl)benzene.

Discussion. The fluorination of 1-bromo-4-ethylbenzene and the fluorination 1-bromo-4-(1-fluoroethyl)benzene were examined. C(sp^3)–H fluorination to produce difluorinated **S4** was found to be a less efficient reaction than the functionalization of 1-bromo-4-ethylbenzene to produce **18** (**Supplementary Figure 13**). Although methyl radical hydrogen atom transfer should be more favorable for C(sp^3)–H partners with a geminal fluorine, the oxidation potential of the resulting radical is hypothesized to be higher that its non-fluorinated counterpart. This is also consistent with the results in section IID (**Supplementary Figure 5**) wherein reactions with 1:1 stoichiometry of diphenylmethane to abstractor **1**, monofluorinated product **2** is observed in 17% yield and difluorinated product **44** is observed in 2% yield. Diminished yields of **2** are consistent with product inhibition, however increased yield of the difluorinated product **44** are not observed suggesting oxidation of the fluorinated product radical is less favorable.

VI. Characterization of Nucleophile Addition Products

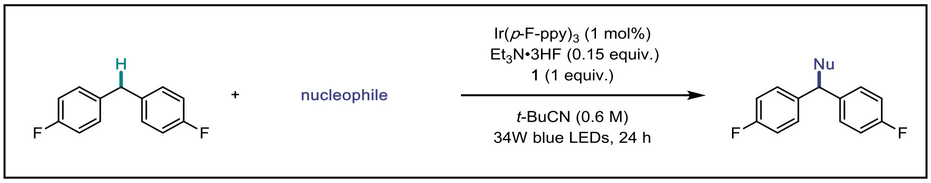

General procedure D for nucleophile scope:

Reagent handling: Ir(*p*-F-ppy)$_3$ was stored in a Drierite-containing desiccator and weighed out on the bench. Phthalimide abstractor **1** was prepared according to literature procedure,[8] dried under vacuum for 48 h, stored in a Drierite-containing desiccator, and weighed out on the bench. Triethylamine trihydrofluoride, in a PTFE plastic vial, was degassed under nitrogen for 1 h and brought into the glovebox without further purification. Anhydrous pivalonitrile was passed through a plug a neutral alumina, degassed under nitrogen for 1 h, and stored in the glovebox over 4Å molecular sieves. 4,4′-difluorodiphenylmethane was distilled over calcium hydride then degassed under nitrogen for 30 min and brought into the glovebox in a sealed vial. Tetrabutylammonium chloride was dried under vacuum for 12 h and brought into the glovebox in a sealed vial.

Reaction setup (0.250 mmol scale): To a 1-dram oven-dried vial, equipped with a Teflon stir bar, was added a Ir(*p*-F-ppy)$_3$ (1.8 mg, 2.5 μmol, 1.0 mol %) and phthalimide-derived abstractor **1** (51.3 mg, 0.25 mmol, 1.00 equiv). The vial containing photocatalyst and abstractor **1** was then covered with a Kimwipe and pumped into a nitrogen-filled glovebox. To the reaction vial was added 4,4′-difluorodiphenylmethane (268 μL, 1.50 mmol, 6.00 equiv), nucleophile (1.50 mmol, 6.00 equiv), triethylamine trihydrofluoride (6.1 μL, 0.04 mmol, 0.15 equiv), and pivalonitrile (417 μL, 0.60 M). The vial was capped, removed from the glovebox and sealed with electrical tape prior to irradiation. The reaction was stirred at 800 rpm for 24 h while illuminating with three 34W blue LED lamps (Kessil KSH150B) and two cooling fans (**Supplementary Figure 1**). The crude reaction mixture was passed through a short pad of silica, eluting with $CDCl_3$, and analyzed by ^{19}F NMR relative to 1-fluoronapthalene (32.3 μL, 0.250 mmol, 1.00 equiv) as an external standard.

Notes on purification: Functionalization with lighter nucleophile coupling partners resulted in the formation of volatile products that were either fully or partially lost in purification. In these cases, ^{19}F NMR yields were used with 1-fluoronaphthalene as an external standard.

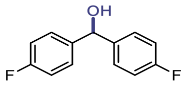

4,4'-difluorobenzhydryl alcohol (51) was prepared from water (27.0 µL, 1.50 mmol, 6.00 equiv) according to general procedure D. The title compound was isolated via preparatory thin-layer chromatography, eluting with 30% ethyl acetate in hexanes (36% NMR yield, average of two runs; Run 1: 37% yield, Run 2: 36% yield). Characterization data is consistent with reported literature values.[40,41]

^{1}H NMR (500 MHz, $CDCl_3$): δ 7.34 – 7.31 (m, 4H), 7.04 – 6.01 (m, 4H), 5.83 (d, *J* = 3.0 Hz, 1H).

^{13}C NMR (126 MHz, $CDCl_3$): δ 162.4 (d, *J* = 246.2 Hz), 139.6 (d, *J* = 3.1 Hz), 128.3 (d, *J* = 8.1 Hz), 115.6 (d, *J* = 21.5 Hz), 75.1.

^{19}F NMR (282 MHz, $CDCl_3$): δ -114.8 – -114.9 (m, 2F).

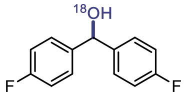

4,4'-difluorobenzhydryl alcohol-^{18}O (52) was prepared from $H_2{}^{18}O$ (27.1 µL, 1.50 mmol, 6.00 equiv) according to general procedure D. The title compound was isolated via preparatory thin-layer chromatography, eluting with 1% methanol in dichloromethane (40% NMR yield, average of two runs; Run 1: 39% yield, Run 2: 40% yield). HRMS with ESI was unable to identify an intact mass.

^{1}H NMR (500 MHz, $CDCl_3$): δ 7.33 (dd, *J* = 8.6, 5.3 Hz, 4H), 7.03 (t, *J* = 8.7 Hz, 4H), 5.83 (d, *J* = 3.1 Hz, 1H), 2.19 (d, *J* = 3.4 Hz, 1H).

^{13}C NMR (126 MHz, $CDCl_3$): δ 162.4 (d, *J* = 246.1 Hz), 139.6 (d, *J* = 3.1 Hz), 128.3 (d, *J* = 8.1 Hz), 115.6 (d, *J* = 21.5 Hz), 75.1.

^{19}F NMR (282 MHz, $CDCl_3$): δ -114.8 – -114.9 (m, 2F).

FTIR (ATR, cm^{-1}): 2958, 2922, 2851, 913, 743.

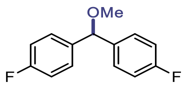

4,4'-(methoxymethylene)bis(fluorobenzene) (53) was prepared from methanol (60.7 µL, 1.50 mmol, 6.00 equiv) according to general procedure D. The title compound was

isolated via preparatory thin-layer chromatography, eluting with 10% ethyl acetate in hexanes (40% NMR yield, average of two runs; Run 1: 39% yield, Run 2: 49% yield). Characterization data is consistent with reported literature values.[41]

^{1}H NMR (500 MHz, $CDCl_3$): δ 7.29 – 7.27 (m, 4H), 7.03 – 7.00 (m, 4H), 5.20 (s, 1H), 3.35 (s, 3H).

^{13}C NMR (126 MHz, $CDCl_3$): δ 162.3 (d, *J* = 245.9 Hz), 137.8 (d, *J* = 3.1 Hz), 128.7 (d, *J* = 8.1 Hz), 115.5 (d, *J* = 21.4 Hz), 84.1, 57.1.

^{19}F NMR (282 MHz, $CDCl_3$): δ -114.9 – -115.0 (m, 2F).

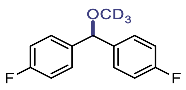

4,4'-((methoxy-*d*$_3$)methylene)bis(fluorobenzene) (54) was prepared from methanol-*d*$_4$ (60.9 µL, 1.50 mmol, 6.00 equiv) according to general procedure D. The title compound was isolated via preparatory thin-layer chromatography, eluting with 10% ethyl acetate in hexanes (42% NMR yield, average of two runs; Run 1: 44% yield, Run 2: 40% yield). HRMS with ESI was unable to identify an intact mass.

^{1}H NMR (500 MHz, $CDCl_3$): δ 7.29 – 7.26 (m, 4H), 7.01 (t, *J* = 8.7 Hz, 4H), 5.20 (s, 1H).

^{13}C NMR (126 MHz, $CDCl_3$): δ 162.3 (d, *J* = 245.9 Hz), 137.8 (d, *J* = 2.9 Hz), 128.7 (d, *J* = 8.1 Hz), 115.5 (d, *J* = 21.4 Hz), 84.0.

^{19}F NMR (282 MHz, $CDCl_3$): δ -114.94 – -115.04 (m, 2F).

^{2}H NMR (77 MHz, $CDCl_3$): δ 3.35 (s, 3D).

FTIR (ATR, cm^{-1}): 2958, 2925, 2854, 1603, 1509, 1464, 1226, 1154, 1120, 913, 830, 743.

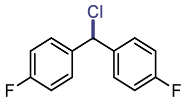

4,4'-(chloromethylene)bis(fluorobenzene) (55) could be prepared according to either of the following procedures.

Procedure 1. **55** was prepared from HCl•Et_2O (2.00 M) (250 µL, 0.500 mmol, 2.00 equiv) and without Et_3N•3HF, according to general procedure D. The title compound was purified via preparatory thin-layer chromatography, eluting with 100% petroleum

ether (25% NMR yield, average of two runs; Run 1: 25% yield, Run 2: 24% yield). Characterization data is consistent with reported literature values.[42]

Procedure 2. **55** was prepared from tetrabutylammonium chloride (69.5 mg, 0.250 mmol, 1.00 equiv) according to general procedure D with the following deviations. Water (0.72 µL, 0.04 mmol, 0.15 equiv) was added as a co-additive. The title compound was purified via preparatory thin-layer chromatography, eluting with 100% petroleum ether (22% NMR yield, average of two runs; Run 1: 25% yield, Run 2: 19% yield). Characterization data is consistent with reported literature values.[42]

Note: The reaction yield is dependent on the amount of water present and therefore on the purity and dryness of the tetrabutylammonium chloride.

^{1}H NMR (500 MHz, $CDCl_3$): δ 7.39 – 7.33 (m, 4H), 7.07 – 7.01 (m, 4H), 6.11 (s, 1H).

^{13}C NMR (126 MHz, $CDCl_3$): δ 162.5 (d, *J* = 247.8 Hz), 136.9 (d, *J* = 3.3 Hz), 129.6 (d, *J* = 8.3 Hz), 115.7 (d, *J* = 21.7 Hz), 62.9.

^{19}F NMR (282 MHz, $CDCl_3$): δ -113.6 (m, 2F).

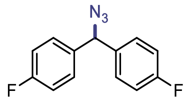

4,4'-(azidomethylene)bis(fluorobenzene) (56) was prepared from azidotrimethylsilane (199 µL, 1.50 mmol, 6.00 equiv) and using water as an additive instead of Et_3N•3HF (0.72 µL, 0.04 mmol, 0.15 equiv), according to general procedure D. The title compound was purified via preparatory thin-layer chromatography (eluting with 5% ethyl acetate in pentane) to produce a clear oil (17% NMR yield, average of two runs; Run 1: 17% yield, Run 2: 17% yield). Characterization data is consistent with reported literature values.[43]

^{1}H NMR (500 MHz, $CDCl_3$): δ 7.33 – 7.20 (m, 4H), 7.05 (t, *J* = 8.6 Hz, 4H), 5.68 (s, 1H).

^{13}C NMR (126 MHz, $CDCl_3$): δ 162.6 (d, *J* = 247.5 Hz), 135.4 (d, *J* = 2.9 Hz), 129.2 (d, *J* = 8.3 Hz), 115.9 (d, *J* = 21.7 Hz), 67.2.

^{19}F NMR (282 MHz, $CDCl_3$): δ -113.7 (m, 2F).

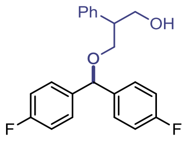

3-(bis(4-fluorophenyl)methoxy)-2-phenylpropan-1-ol (57) was prepared from 2-phenyl-1,3-propanediol (114 mg, 0.750 mmol, 3.00 equiv) according to general procedure D. The title compound was isolated via preparatory thin-layer chromatography, eluting with 100% dichloromethane, to produce a clear oil (37.4 mg, 42% yield). HRMS with ESI was unable to identify an intact mass.

^{1}H NMR (500 MHz, $CDCl_3$): δ 7.33 – 7.29 (m, 2H), 7.25 – 7.19 (m, 7H), 7.03 – 6.97 (m, 4H), 5.32 (s, 1H), 4.05 – 3.88 (m, 2H), 3.77 – 3.69 (m, 2H), 3.26 – 3.21 (m, 1H), 2.08 (t, *J* = 6.4 Hz, 1H).

^{13}C NMR (126 MHz, $CDCl_3$): δ 162.7 (d, *J* = 246.0 Hz), 139.6, 137.6 (dd, *J* = 12.1, 3.2 Hz), 128.8, 128.6 (d, *J* = 8.1 Hz), 128.2, 127.3, 115.6 (dd, *J* = 21.5, 7.0 Hz), 83.1, 72.0, 66.1, 48.2.

^{19}F NMR (282 MHz, $CDCl_3$): δ -114.6 – -114.7 (m, 2F).

FTIR (ATR, cm^{-1}): 3389, 3061, 3030, 2966, 2917, 1602, 1505, 1453, 1220, 1182, 1154, 1077, 1030, 1014, 824, 699, 553.

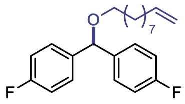

4,4'-((dec-9-en-1-yloxy)methylene)bis(fluorobenzene) (58) was prepared from dec-9-en-1-ol (268 µL, 1.50 mmol, 6.00 equiv) according to general procedure D. The title compound was isolated via preparatory thin-layer chromatography, concentrating the reaction mixture *in vacuo* prior to purification (eluting with 100% petroleum ether) to produce a white solid (29 mg, 33% yield). HRMS with ESI was unable to identify an intact mass.

^{1}H NMR (500 MHz, $CDCl_3$): δ 7.30 – 7.24 (m, 4H), 7.05 – 6.97 (m, 4H), 5.81 (ddt, *J* = 16.9, 10.2, 6.7 Hz, 1H), 5.28 (s, 1H), 5.03 – 4.90 (m, 2H), 3.40 (t, *J* = 6.5 Hz, 2H), 2.08 – 1.99 (m, 2H), 1.67 – 1.58 (m, 2H), 1.36 (m, 4H), 1.28 (m, 6H).

^{13}C NMR (126 MHz, $CDCl_3$): δ 162.2 (d, *J* = 245.6 Hz), 139.4, 138.4 (d, *J* = 3.2 Hz), 128.6 (d, *J* = 8.0 Hz), 115.4 (d, *J* = 21.4 Hz), 114.3, 82.3, 69.4, 33.9, 29.9, 29.6, 29.5, 29.2, 29.0, 26.4.

^{19}F NMR (282 MHz, $CDCl_3$): δ -115.3 (tt, *J* = 8.5, 5.3 Hz, 2F).

FTIR (ATR, cm^{-1}): 2926, 2855, 1662, 1601, 1507, 1233, 1159, 1095, 1014, 824, 769, 568, 518.

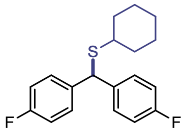

(bis(4-fluorophenyl)methyl)(cyclohexyl)sulfane (59) was prepared from cyclohexanethiol (184 μL, 1.50 mmol, 6.00 equiv) according to general procedure D. The reaction was conducted for 6 h. The title compound was isolated via preparatory thin-layer chromatography, concentrating the reaction mixture *in vacuo* prior to purification (eluting with 10% dichloromethane in hexane) to produce a white solid (25 mg, 31% yield). Characterization data is consistent with reported literature values.[44]

^{1}H NMR (500 MHz, $CDCl_3$): δ 7.39 – 7.33 (m, 4H), 7.02 – 6.96 (m, 4H), 5.20 (s, 1H), 2.43 (m, 1H), 1.91 – 1.82 (m, 2H), 1.77 – 1.64 (m, 2H), 1.40 – 1.12 (m, 6H).

^{13}C NMR (126 MHz, $CDCl_3$): δ 161.9 (d, *J* = 246.0 Hz), 137.6 (d, *J* = 3.1 Hz), 129.9 (d, *J* = 8.0 Hz), 115.5 (d, *J* = 21.5 Hz), 51.0, 43.8, 33.3, 26.0, 25.9.

^{19}F NMR (282 MHz, $CDCl_3$): δ -115.5 (m, 2F).

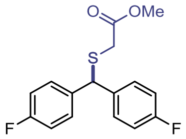

methyl 2-((bis(4-fluorophenyl)methyl)thio)acetate (60) was prepared from methyl thioglycolate (134 mL, 1.50 mmol, 6.00 equiv) according to general procedure D. The title compound was isolated via preparatory thin-layer chromatography (eluting with 10% ethyl acetate in hexanes) to produce a clear oil (23.3 mg, 30% yield). HRMS with ESI was unable to identify an intact mass.

^{1}H NMR (500 MHz, $CDCl_3$): δ 7.39 – 7.36 (m, *J* =, 4H), 7.03 – 7.00 (m, 4H), 5.38 (s, 1H), 3.69 (s, 3H), 3.07 (s, 2H).

^{13}C NMR (126 MHz, $CDCl_3$): δ 170.59, 162.2 (d, *J* = 246.9 Hz), 136.0 (d, *J* = 3.3 Hz), 130.1 (d, *J* = 8.1 Hz), 115.7 (d, *J* = 21.5 Hz), 52.7, 52.6, 33.5.

^{19}F NMR (282 MHz, $CDCl_3$): δ -114.6 – -114.7 (m, 2F).

FTIR (ATR, cm^{-1}): 2926, 1732, 1650, 1604, 1507, 1454, 1227, 1158, 905, 837, 728, 650.

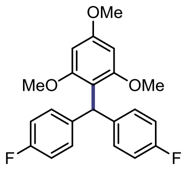

2-Bis(4-fluorophenyl)methyl-1,3,5-trimethoxybenzene (61) was prepared from 1,3,5-trimethoxybenzene (126 mg, 0.75 mmol, 3.00 equiv) according to general procedure D. The title compound was isolated via preparatory thin-layer chromatography, eluting with 10% ethyl acetate in hexanes to produce a yellow oil (50.2 mg, 41% isolated yield). Characterization data is consistent with reported literature values.[45]

^{1}H NMR (500 MHz, $CDCl_3$): δ 7.12 (m, 4H), 6.90 (t, *J* = 8.8 Hz, 4H), 6.14 (s, 2H), 5.97 (s, 1H), 3.80 (s, 3H), 3.60 (s, 6H).

^{13}C NMR (126 MHz, $CDCl_3$): δ 161.2 (d, *J* = 222.1 Hz), 160.2, 159.0, 139.7 (d, *J* = 3.3 Hz), 130.5 (d, *J* = 7.7 Hz), 114.4 (d, *J* = 21.0 Hz), 113.2, 91.7, 55.8, 55.4, 43.8.

^{19}F NMR (282 MHz, $CDCl_3$): δ -118.4 – -118.5 (m, 2F).

FTIR (ATR, cm^{-1}): 2999, 2937, 2838, 1590, 1504, 1455, 1417, 1220, 1203, 1148, 1113, 1060, 818, 537.

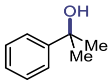

2-phenylpropan-2-ol (62) was prepared from cumene (209 μL, 1.50 mmol, 6.00 equiv) and water (27.0 μL, 1.50 mmol, 6.00 equiv), according to general procedure D. The title compound was isolated via preparatory thin-layer chromatography, concentrating the reaction mixture *in vacuo* prior to purification (eluting with 1% methanol in dichloromethane) to produce a clear oil (15.7 mg, 41% yield). Characterization data is consistent with reported literature values.[46]

^{1}H NMR (500 MHz, $CDCl_3$): δ 7.53 – 7.48 (m, 2H), 7.35 (t, *J* = 7.7 Hz, 2H), 7.24 (t, *J* = 7.2 Hz, 1H), 1.59 (s, 6H).

^{13}C NMR (126 MHz, $CDCl_3$): δ 149.2, 128.4, 126.9, 124.5, 72.7, 31.9.

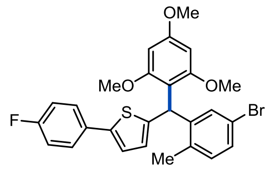

2-((5-bromo-2-methylphenyl)(2,4,6-trimethoxyphenyl)methyl)-5-(4-fluorophenyl)thiophene (63) was prepared from 2-(5-Bromo-2-methylbenzyl)-5-(4-fluorophenyl)thiophene (163 mg, 0.45 mmol, 3.00 equiv) and 1,3,5-trimethoxybenzene (75.6 mg, 0.75 mmol, 3.00 equiv) according to general procedure D. After reacting for 24 h, the reaction mixture was passed through a short plug of silica gel. The title compound was purified via preparatory thin-layer chromatography (eluting with 25% ethyl acetate in hexane) to produce a white solid (41.6 mg, 53% yield).

^{1}H NMR (500 MHz, $CDCl_3$): δ 7.54 (d, *J* = 2.2 Hz, 1H), 7.52 – 7.47 (m, 2H), 7.21 (dd, *J* = 8.1, 2.2 Hz, 1H), 7.05 (d, *J* = 3.6 Hz, 1H), 7.04 – 6.98 (m, 2H), 6.94 (dd, *J* = 8.1, 0.7 Hz, 1H), 6.66 (dd, *J* = 3.7, 1.2 Hz, 1H), 6.15 (s, 2H), 6.13 (s, 1H), 3.81 (s, 3H), 3.66 (s, 6H), 2.14 (s, 3H).

^{13}C NMR (126 MHz, $CDCl_3$): δ 162.1 (d, *J* = 246.3 Hz), 160.7, 159.1, 147.0, 144.4, 141.2, 135.3, 133.1, 131.5, 129.0, 127.2 (d, *J* = 7.8 Hz), 126.9, 122.5, 119.0, 115.7 (d, *J* = 21.7 Hz), 111.7, 91.7, 55.9, 55.4, 38.9, 19.1.

^{19}F NMR (376 MHz, $CDCl_3$): δ -115.6 (m, 1F).

FTIR (ATR, cm^{-1}): 2936, 2837, 1603, 1589, 1507, 1457, 1417, 1223, 1204, 1154, 1115, 809, 732.

HRMS: (ESI-TOF) calculated for $C_{27}H_{25}BrFO_3S^+$ ($[M+H]^+$): 527.0686, found 527.0688.

VII. Mechanistic Studies

A. Spectroscopic and Emission Quenching Experiments.

Absorption and emission experiments were conducted in line with our previous publication on the arylation of ethereal C–H bonds.[47] An excitation wavelength of 415 nm (ε = 4.3 × 10^3 M^{-1} cm^{-1}) and an emission wavelength of 508 nm were used for monitoring quenching of the iridium photocatalyst. All reagents were prepared in stock solutions inside a nitrogen filled glove box. Reagents were diluted in pivalonitrile (2 mL) and sealed in a screw-top 1.0 cm quartz cuvette. A blank composed of pivalonitrile was used in absorbance measurements. To each cuvette was added stock solutions of Ir(*p*-F-ppy)$_3$ (2.62 mM in pivalonitrile, amount dispensed: 45 µL, 5.9 × 10^{-5} M after dilution) and quencher, followed by pivalonitrile to dilute to a volume of 2 mL. Absorption spectra were collected on an Agilent Technologies Cary 60 UV-Vis Spectrophotometer. Emission quenching data were collected on an Agilent Cary Eclipse Fluorescence Spectrophotometer with excitation and emissions slit widths of 2.5 nm and 20 nm. Note: Error bars are not observable on all points as they are smaller than the size of the points represented.

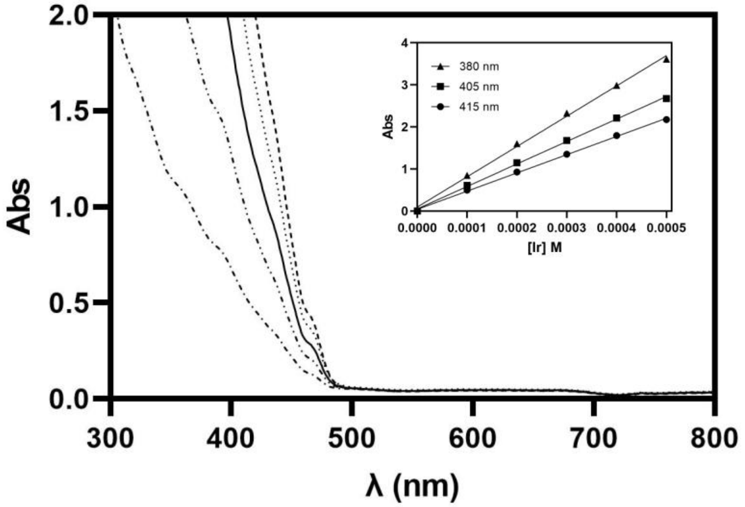

Supplementary Figure 14. Electronic absorption spectra of Ir(*p*-F-ppy)$_3$ in pivalonitrile at various concentrations around those employed in quenching experiments: dot dashed 1.0×10^{-4} M, dot dot dashed 2×10^{-4} M, solid 3×10^{-4} M, dotted 4×10^{-4} M, dashed 5×10^{-4} M. In the inset calibration curves at various wavelengths: Ir(*p*-F-ppy)$_3$ at 380 nm (ε = 7.2 × 10^4 M^{-1} cm^{-1}), at 405 nm (ε = 5.3 × 10^3 M^{-1} cm^{-1}), at 415 nm (excitation wavelength used in quenching experiments; ε = 4.3 × 10^3 M^{-1} cm^{-1}).

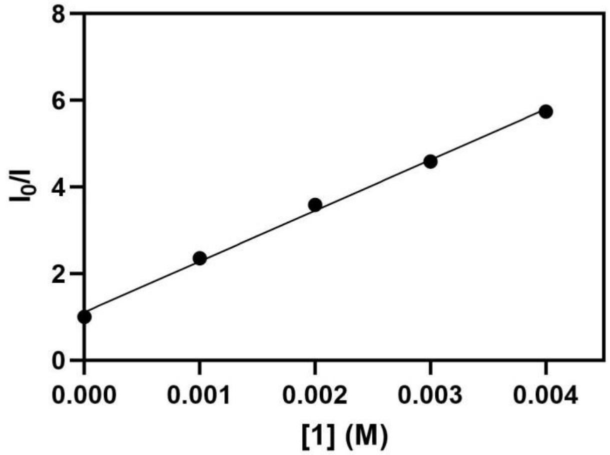

[1] (M)	Trial 1 (I)	Trial 2 (I)	Trial 3 (I)
0	665.40	681.04	602.27
0.001	278.05	278.61	268.76
0.002	188.90	185.74	168.04
0.003	144.91	148.75	131.06
0.004	116.49	116.72	105.86

slope	y-intercept	R^2
1172	1.113	0.996

Supplementary Figure 15. Plot and data of Ir(*p*-F-ppy)$_3$ emission quenching by phthalimide ester **1** in pivalonitrile.

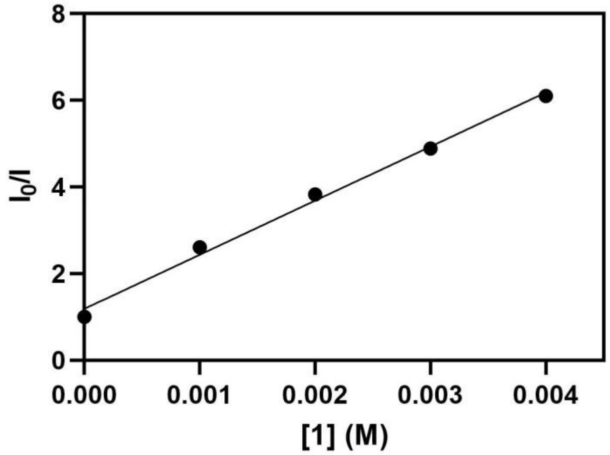

[1] (M)	[Et_3N•3HF] (M)	Trial 1 (I)	Trial 2 (I)	Trial 3 (I)
0	0.024	756.63	716.91	755.65
0.001	0.024	278.79	281.28	293.74
0.002	0.024	194.69	190.04	196.87
0.003	0.024	151.20	150.62	154.25
0.004	0.024	120.87	118.70	125.61

slope	y-intercept	R^2
1249	1.190	0.994

Supplementary Figure 16. Plot and data of Ir(*p*-F-ppy)$_3$ emission quenching by varied concentrations of phthalimide ester **1** and constant Et_3N•3HF in pivalonitrile.

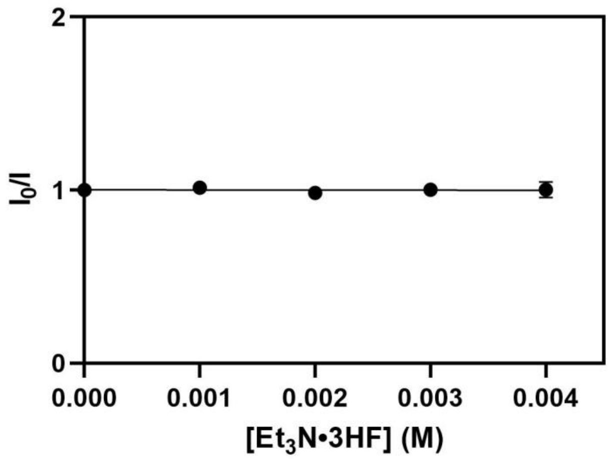

$[Et_3N•3HF]$ (M)	Trial 1 (I)	Trial 2 (I)
0	608.18	639.72
0.001	607.42	629.17
0.002	619.78	642.49
0.003	623.63	659.75
0.004	626.35	462.50
slope	y-intercept	R^2
-0.660	1.002	0.002

Supplementary Figure 17. Plot and data of Ir(*p*-F-ppy)$_3$ emission quenching by $Et_3N•3HF$ in pivalonitrile.

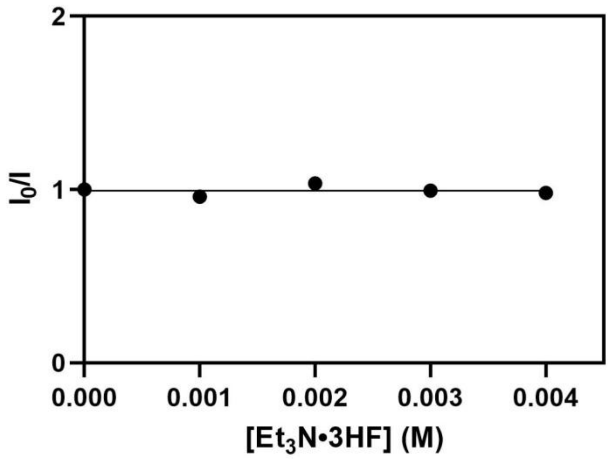

$[Et_3N•3HF]$ (M)	[1] (M)	Trial 1 (I)	Trial 2 (I)
0	0.01	95.76	91.28
0.001	0.01	97.83	97.25
0.002	0.01	91.89	88.55
0.003	0.01	95.03	93.18
0.004	0.01	95.99	94.57
slope	y-intercept		R^2
-0.224	0.995		0.000

Supplementary Figure 18. Plot and data of Ir(*p*-F-ppy)$_3$ emission quenching by constant phthalimide ester **1** and varied concentrations of $Et_3N•3HF$ in pivalonitrile.

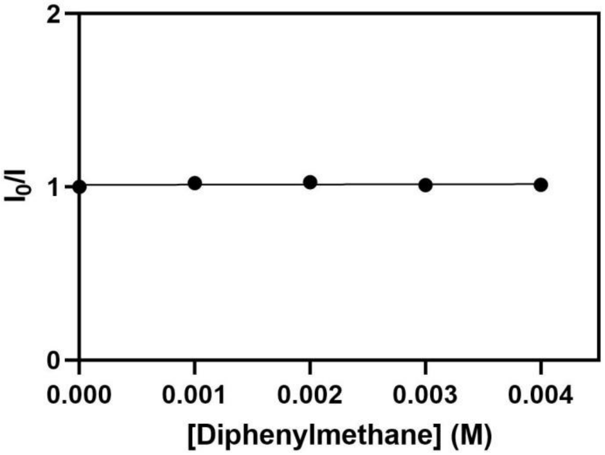

[diphenylmethane] (M)	Trial 1 (I)	Trial 2 (I)
0	749.16	704.02
0.001	720.11	699.82
0.002	729.36	685.17
0.003	728.19	709.34
0.004	736.79	698.94
slope	y-intercept	R^2
1.152	1.012	0.012

Supplementary Figure 19. Plot and data of Ir(*p*-F-ppy)$_3$ emission quenching by diphenylmethane in pivalonitrile.

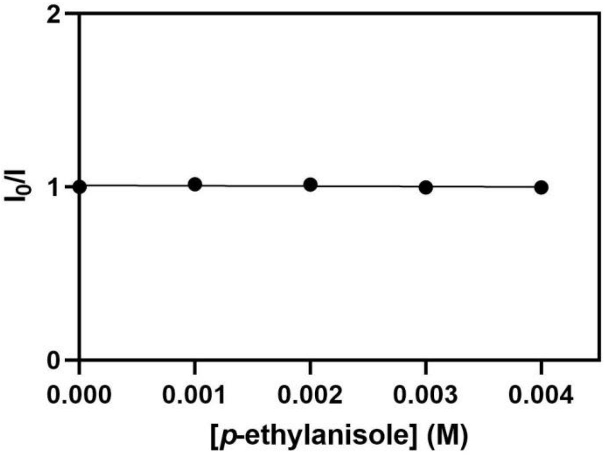

[*p*-ethylanisole] (M)	Trial 1 (I)	Trial 2 (I)
0	687.29	714.30
0.001	677.05	703.62
0.002	674.49	706.90
0.003	692.77	711.91
0.004	688.72	716.25
slope	y-intercept	R^2
-2.222	1.009	0.130

Supplementary Figure 20. Plot and data of Ir(*p*-F-ppy)$_3$ emission quenching by *p*-ethylanisole in pivalonitrile.

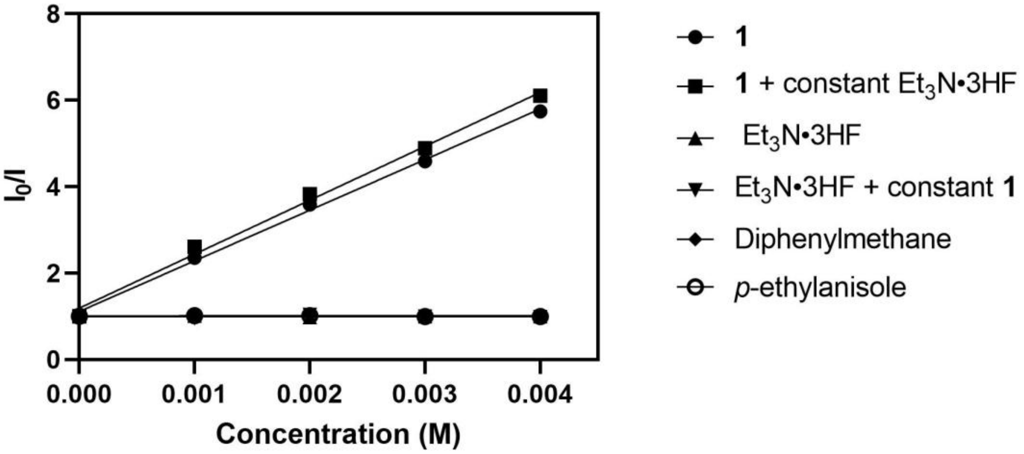

Supplementary Figure 21. Compiled data of Ir(*p*-F-ppy)$_3$ emission quenching by various quenchers in pivalonitrile.

Discussion. Based on the quenching experiments, phthalimide ester **1** interacts with the photocatalyst preferentially. Phthalimide esters are oxidative quenchers; therefore, the observed interaction is indicative of oxidative quenching of the photocatalyst, providing further mechanistic support for the proposed strategy. The Stern-Volmer quenching rate constant is 5.7×10^8 M^{-1} s^{-1} (based on a literature lifetime for Ir(*p*-F-ppy)$_3$, 2040 ns).[48] A small increase in quenching rate (6.1×10^8 M^{-1} s^{-1}) was observed in the presence of Et_3N•3HF. Previous literature reports have shown that hydrogen bond donors can facilitate reduction of related compounds via proton-coupled electron transfer (PCET).[49] However, given the small difference in Stern-Volmer quenching rate constants, the role of Et_3N•3HF is more likely to prevent back-electron transfer and aid fragmentation of the reduced phthalimide ester **1** or modulate the photophysics of the photocatalyst via hydrogen bonding. Experimental studies are ongoing to probe these possibilities.

B. Light-Dark Cycle Experiment.

Procedure (0.150 mmol scale): To a 1-dram oven-dried vial, equipped with a Teflon stir bar, was added Ir(*p*-F-ppy)$_3$ (1.1 mg, 1.5 µmol, 1.0 mol %) and phthalimide abstractor **1** (30.8 mg, 0.150 mmol, 1.00 equiv). The vial containing photocatalyst and phthalimide abstractor **1** was then covered with a Kimwipe and pumped into a nitrogen-filled glovebox. To the vial containing photocatalyst and phthalimide abstractor was added diphenylmethane (150 µL, 0.900 mmol, 6.00 equiv), triethylamine trihydrofluoride (147 µL, 0.900 mmol, 6.00 equiv), and pivalonitrile (250 µL, 0.600 M). The vials were capped, removed from the glovebox and sealed with electrical tape prior to irradiation. A series of 5 reactions were set up. The reactions were stirred at 400 rpm for the indicated time intervals while illuminating with three 34W blue LED lamps (Kessil KSH150B) and two cooling fans (**Supplementary Figure 1**). The crude reaction mixture was passed

through a short pad of silica, eluting with $CDCl_3$, and analyzed by 19F NMR relative to 1-fluoronapthalene (19.4 µL, 0.150 mmol, 1.00 equiv) as an external standard.

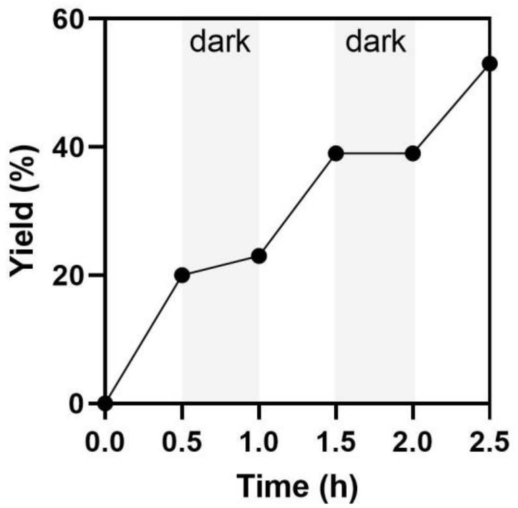

Supplementary Figure 22. The light-dark cycle experiment. Yields are reported as an average of three runs. Although light-dark experiments cannot rule out chain propagation,[50–52] the light-dark study did not show that significant propagation was occurring during dark intervals.

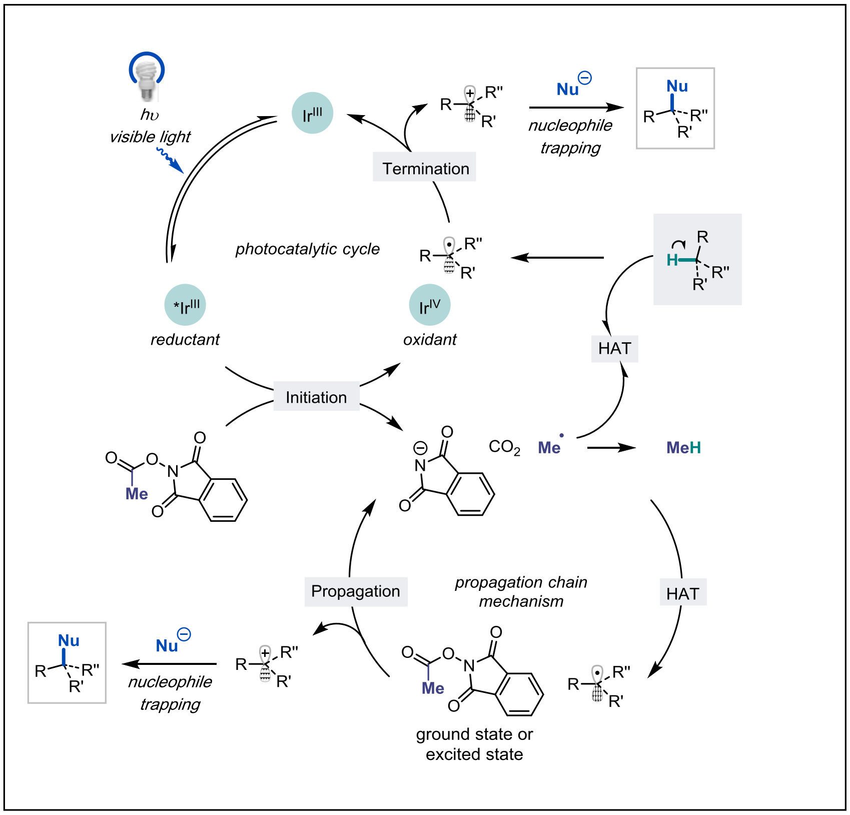

Supplementary Figure 23. Alternative chain propagation mechanism.

Discussion. Previous studies examining Ir photocatalyst/phthalimide ester reaction systems have shown that at any time during the reaction course there likely exists only a small amount of excited phthalimide ester.[31] Therefore a chain mechanism propagated by excited state phthalimide ester is unlikely. A previously reported quantum yield (0.37) of an analogous reaction system lends further support for a chain mechanism being unlikely or inefficient.[31] A light-dark cycling experiment was conducted to examine if significant reactivity was observed without constant irradiation. This experiment only showed a small increase in product yield during the first light off interval, which was within experimental error.

A ground state chain propagation mechanism has also been considered. The literature reduction potential for *N*-hydroxyphthalimide ester **1** is reported to be $E_{1/2}^{red}$ ~ -1.24 V vs. SCE in DMF. The literature oxidation potentials for C(sp^3)–H coupling partner radicals are reported to be ~ 0.73 V – 0.09 V vs SCE.[53] Therefore, reduction of the redox active ester by the radical species is thermodynamically disfavored.

C. *In situ* NMR.

Reaction setup (0.150 mmol scale): To a 1-dram oven-dried vial, equipped with a Teflon stir bar, was added Ir(*p*-F-ppy)$_3$ (1.1 mg, 1.5 µmol, 1.0 mol %) and phthalimide abstractor **1** (30.8 mg, 0.150 mmol, 1.00 equiv). The vial containing photocatalyst and phthalimide abstractor was then covered with a Kimwipe and pumped into a nitrogen-filled glovebox. To the vial containing photocatalyst and phthalimide abstractor was added diphenylmethane (150 µL, 0.900 mmol, 6.00 equiv), triethylamine trihydrofluoride (147 µL, 0.900 mmol, 6.00 equiv), 1-fluoronapthalene (19.4 µL, 0.150 mmol, 1.00 equiv) and CD_3CN (250 µL, 0.60 M). The reaction mixture was transferred to J. Young NMR tube. The NMR tube was capped, removed from the glovebox and irradiated. The reaction was allowed to proceed for 1h while illuminating with one 34W blue LED lamp (Kessil KSH150B) and one cooling fan. The crude reaction mixture was analyzed by ^{1}H NMR relative to 1-fluoronapthalene as an internal standard. The reaction was allowed to proceed for an additional 2h while illuminating with one 34W blue LED lamp (Kessil KSH150B) and one cooling fan. The crude reaction mixture was directly analyzed by ^{1}H NMR relative to 1-fluoronapthalene as an internal standard. Methane was observed in increasing amounts at 1 h (**Supplementary Figure 24**) and 3 h (**Supplementary Figure 25**) time intervals.

PhotoNMR. PhotoNMR reactions were set up as described above and then transferred to a modified J. Young NMR tube equipped with an inner cell for an optic cable insert (part number: NE-379-5-Br). For each spectrum, 16 scans were acquired for a total data collection time of 1 minute 7 seconds per data point. The last spectrum of each sample represents an extended data collection of 500 scans. Receiver gain optimization is done for the first spectrum only and is bypassed in the remaining time points. Two dummy scans are used as a countdown for turning on the light source at time = 0. As soon as the second dummy scan is finished, the LED is turned on manually. Shimming is done manually for each sample to achieve optimal peak shape prior to data collection. The samples were irradiated with 445nm LED fiber optic cable.

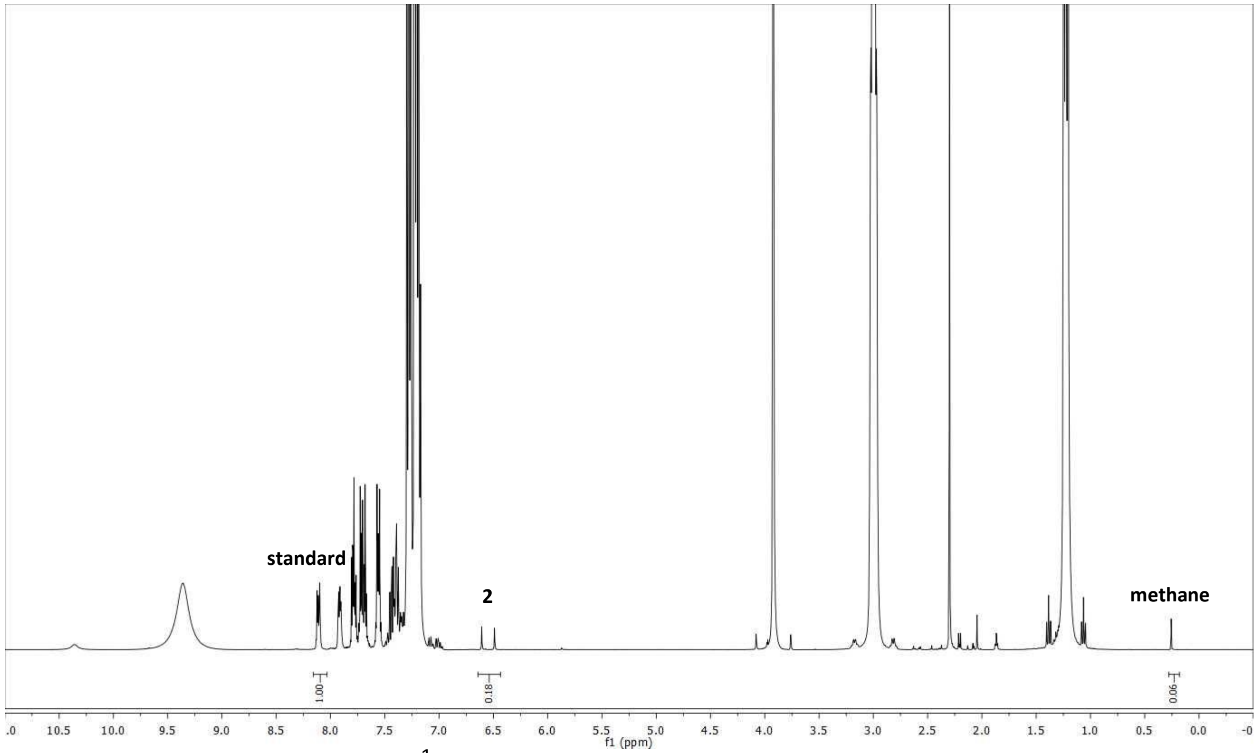

Supplementary Figure 24. ^{1}H NMR (CD_3CN) of the fluorination of diphenylmethane after 1h.

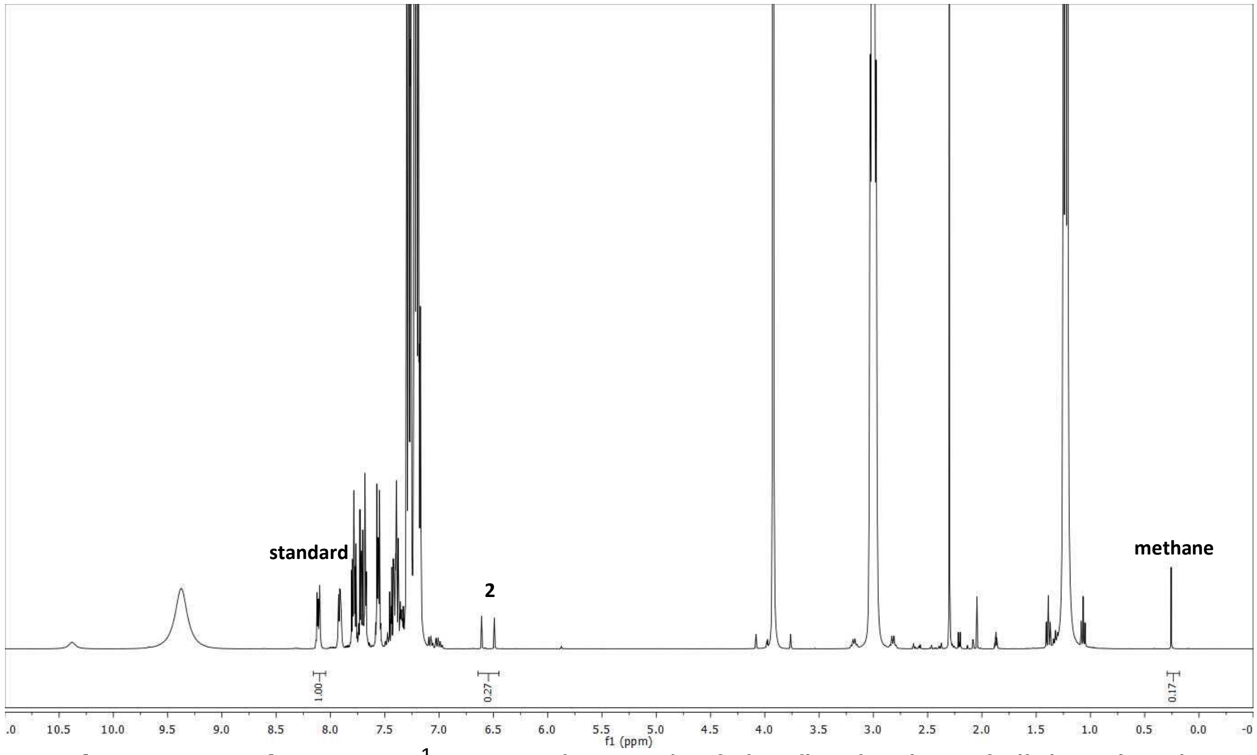

Supplementary Figure 25. ^{1}H NMR (CD_3CN) of the fluorination of diphenylmethane after 3h.

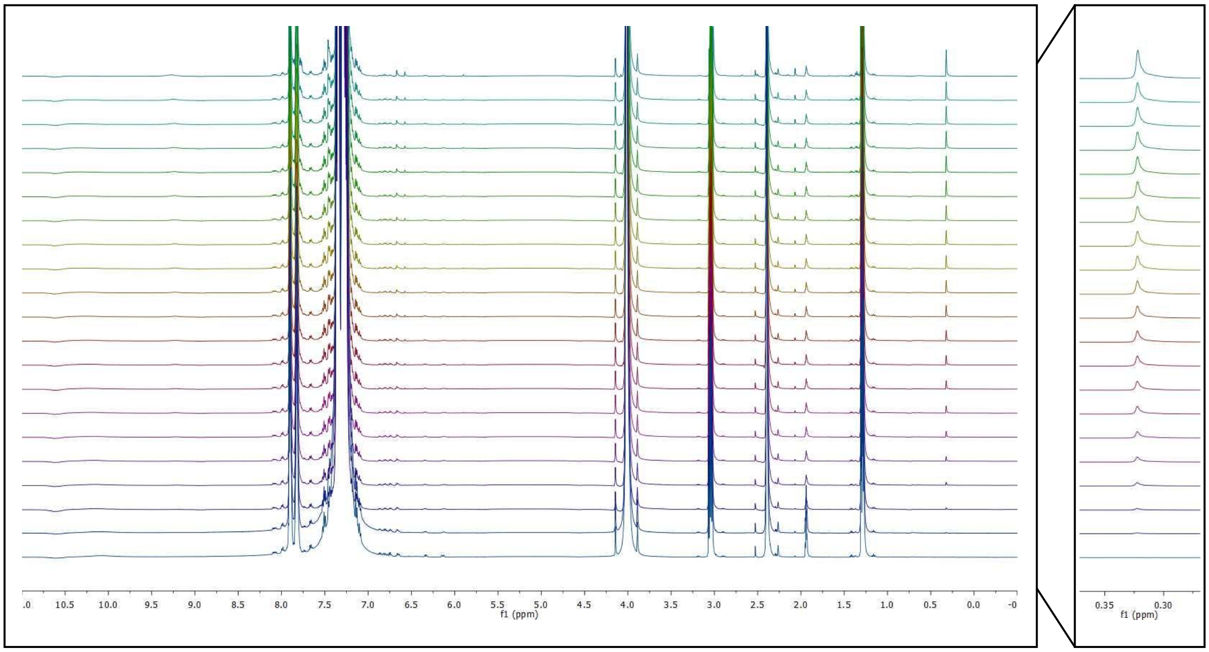
.0 10.5 10.0 9.5 9.0 8.5 8.0 7.5 7.0 6.5 6.0 5.5 5.0 4.5 4.0 3.5 3.0 2.5 2.0 1.5 1.0 0.5 0.0 -0
f1 (ppm)
0.35 0.30
f1 (ppm)

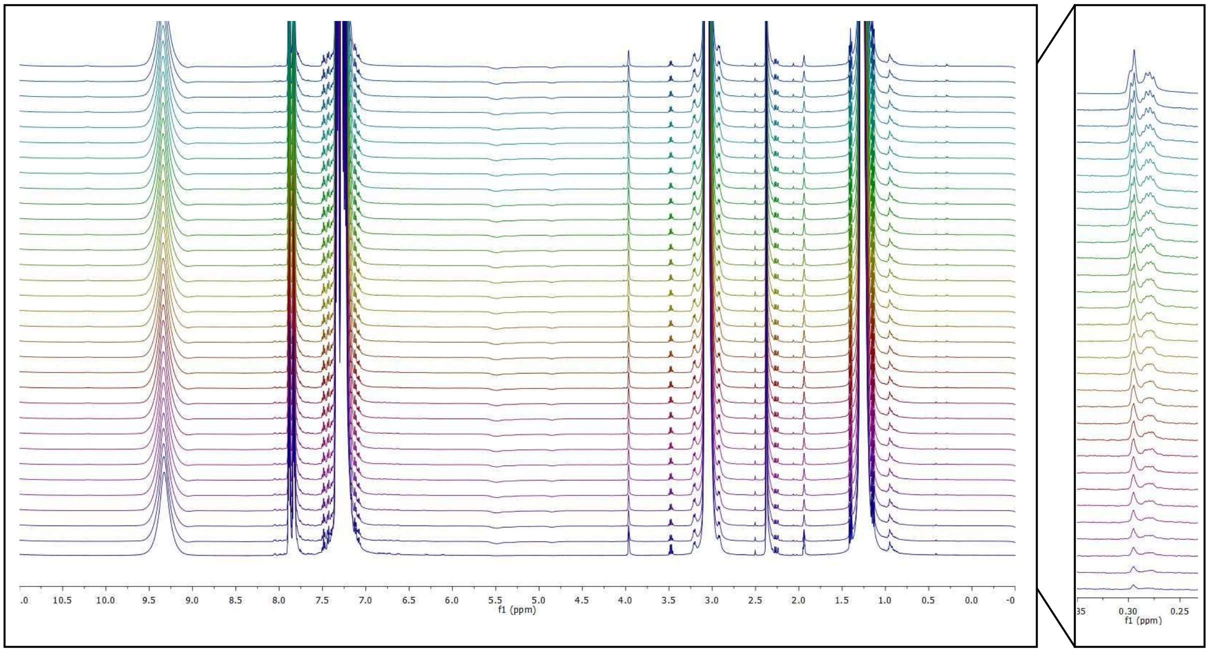
.0 10.5 10.0 9.5 9.0 8.5 8.0 7.5 7.0 6.5 6.0 5.5 5.0 4.5 4.0 3.5 3.0 2.5 2.0 1.5 1.0 0.5 0.0 -0
f1 (ppm)
35 0.30 0.25
f1 (ppm)

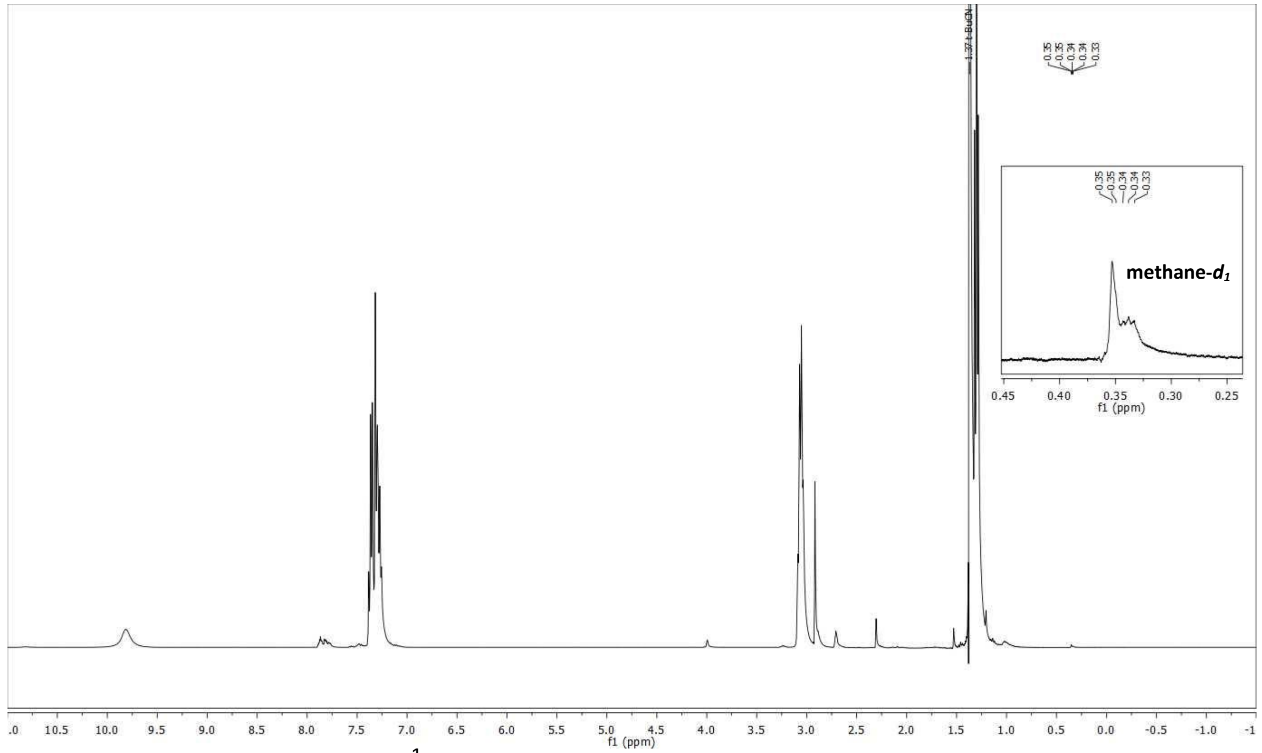

Supplementary Figure 28. ^{1}H NMR (*t*-BuCN) of the fluorination of diphenylmethane-d_2 after 1h.

Reaction progress monitoring and photoNMR studies revealed the evolution of methane gas during the fluorination of diphenylmethane under standard reaction conditions. Moreover, upon performing *in situ* NMR studies with diphenylmethane-d_2, we observed the evolution of CDH_3, indicating that methyl radical indeed facilitates HAT from the substrate (**Supplementary Figure 27** and **28**). This result was only observed when diphenylmethane-d_2 was present and has been illustrated in deuterated (**Supplementary Figure 27**) and non-deuterated solvent (**Supplementary Figure 28**). Methane is also observed in these studies, which is a result of the presence of a small amount of diphenylmethane (3.99 ppm) in the diphenylmethane-d_2 starting material. While acyloxy radicals generated under photocatalytic conditions have been shown to mediate HAT,[54] we did not observe the evolution of acetic acid in these studies, thereby contradicting this species as a HAT mediator in this reaction.

D. Methyl Radical Trapping Experiments.

Radical Trapping with TEMPO. To a 1-dram oven-dried vial equipped with a Teflon stir bar was added Ir(*p*-F-ppy)$_3$ (1.1 mg, 1.5 μmol, 1.0 mol %), 1,3-dioxoisoindolin-2-yl acetate **1** (31.0 mg, 0.150 mmol, 1.00 equiv), and (2,2,6,6-Tetramethylpiperidin-1-yl)oxyl (TEMPO, 35.0 mg, 0.225 mmol, 1.50 equiv) The vial containing photocatalyst, **1**, and TEMPO was then covered with a Kimwipe and pumped into a nitrogen-filled glovebox. To the vial containing photocatalyst and **1** was added triethylamine trihydrofluoride (147 μL, 0.900 mmol, 6.00 equiv), diphenylmethane (150 μL, 0.900 mmol, 6.00 equiv), and pivalonitrile (250 μL, 0.60 M). The vial was then capped, removed from the glovebox and sealed with electrical tape prior to irradiation. The reaction was stirred at 500 rpm

for 6 h while illuminating with three 34W blue LED lamps (Kessil KSH150B) and two cooling fans (**Supplementary Figure 1**). The crude reaction mixture was passed through a short pad of silica, eluting with $CDCl_3$, and analyzed by ^1H NMR and ^{19}F NMR relative to 1-fluoronapthalene (25.8 µL, 0.200 mmol, 1.33 equiv) as an external standard. Spectral data is consistent with literature reports.[55,56]

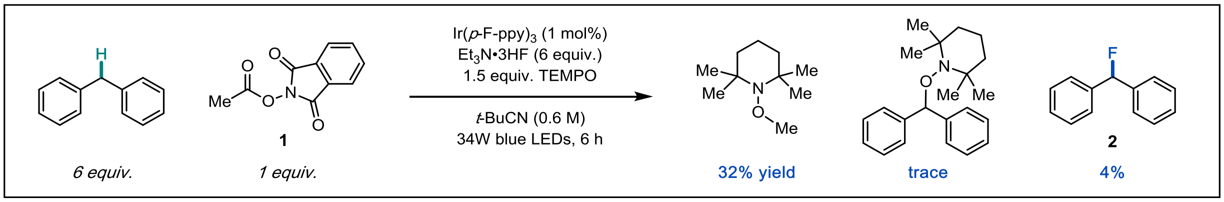

Supplementary Figure 29. TEMPO trapping as a probe for radical intermediacy.

Radical Trapping with 1,1-diphenylethylene. To a 1-dram oven-dried vial equipped with a Teflon stir bar was added Ir(*p*-F-ppy)$_3$ (1.1 mg, 1.5 µmol, 1.0 mol %) and 1,3-dioxoisoindolin-2-yl acetate **1** (31.0 mg, 0.150 mmol, 1.00 equiv). The vial containing photocatalyst and **1** was then covered with a Kimwipe and pumped into a nitrogen-filled glovebox. To the vial containing photocatalyst and **1** was added triethylamine trihydrofluoride (147 µL, 0.900 mmol, 6.00 equiv), 1,1-diphenylethylene (159 µL, 0.900 mmol, 6.00 equiv), and pivalonitrile (250 µL, 0.60 M). The vial was then capped, removed from the glovebox and sealed with electrical tape prior to irradiation. The reaction was stirred at 500 rpm for 6 h while illuminating with three 34W blue LED lamps (Kessil KSH150B) and two cooling fans (**Supplementary Figure 1**). The crude reaction mixture was passed through a short pad of silica, eluting with $CDCl_3$, and analyzed by ^1H NMR and ^{19}F NMR relative to 1-fluoronapthalene (25.8 µL, 0.200 mmol, 1.33 equiv) as an external standard.

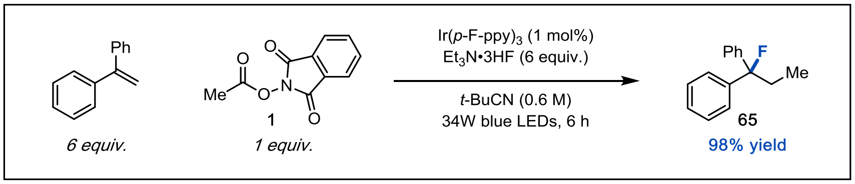

Supplementary Figure 30. Radical trapping with 1,1-diphenylethylene as a probe for methyl radical intermediacy.

E. Competition Experiments.

To a 1-dram oven-dried vial equipped with a Teflon stir bar was added Ir(*p*-F-ppy)$_3$ (1.1 mg, 1.5 µmol, 1.0 mol %) and 1,3-dioxoisoindolin-2-yl acetate **1** (31.0 mg, 0.150 mmol, 1.00 equiv). The vial containing photocatalyst and **1** was then covered with a Kimwipe and pumped into a nitrogen-filled glovebox. To the vial containing photocatalyst and **1** was added triethylamine trihydrofluoride (147 µL, 0.900 mmol, 6.00 equiv) and 5.00 equiv (0.750 mmol) of either cumene and ethylbenzene (tertiary vs. secondary) or ethylbenzene and toluene (secondary vs. primary). Lastly, the vial was charged with pivalonitrile (250 µL, 0.60 M). The vial was then capped, removed from the glovebox and sealed with electrical tape prior to irradiation. The reaction was stirred at 500 rpm

for 6 h while illuminating with three 34W blue LED lamps (Kessil KSH150B) and two cooling fans (**Supplementary Figure 1**). The crude reaction mixture was passed through a short pad of silica, eluting with $CDCl_3$, and analyzed by ^{19}F NMR relative to 1-fluoronapthalene (19.4 μL, 0.15 mmol) as an external standard.

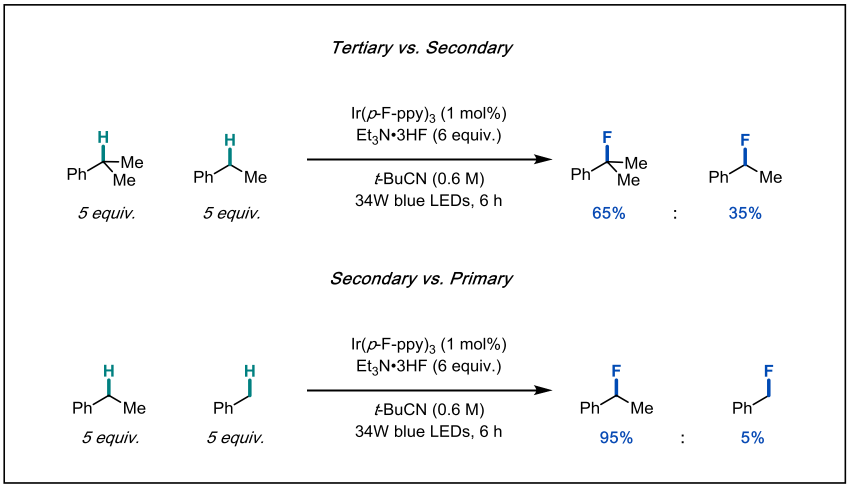

Supplementary Figure 31. Reaction design for site-selectivity competition experiments among tertiary, secondary, and primary C(sp^3)–H coupling partners.

F. Kinetic Isotope Effect Studies.

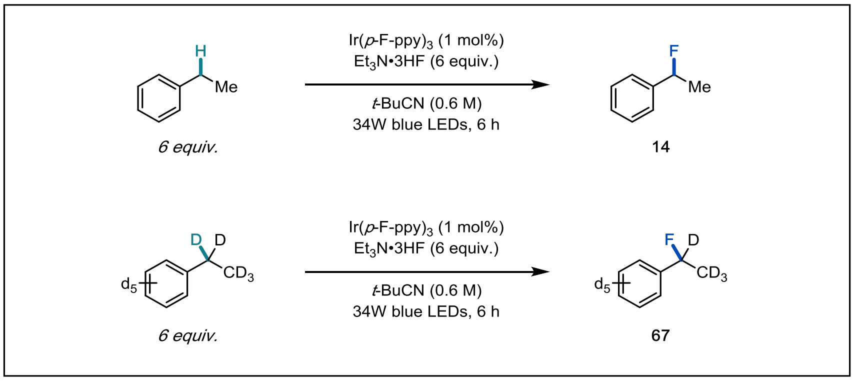

Supplementary Figure 32. Parallel initial rates experiments using ethylbenzene and ethylbenzene-d_{10}.

Initial Rate KIE. To a 2-dram oven-dried vial equipped with a Teflon stir bar was added Ir(*p*-F-ppy)$_3$ (3.2 mg, 4.5 μmol, 1.0 mol %) and 1,3-dioxoisoindolin-2-yl acetate **1** (92.3 mg, 0.450 mmol, 1.00 equiv). The vial was then charged with 2-fluorobiphenyl (15.5 mg, 0.200 mmol) as internal standard. The vial containing photocatalyst, **1**, and 2-fluorobiphenyl was then covered with a Kimwipe and pumped into a nitrogen-filled glovebox. To the vial containing photocatalyst and **1** was added triethylamine trihydrofluoride (440 μL, 2.70 mmol, 6.00 equiv) and 6.00 equiv (2.70 mmol) of either ethylbenzene (331 μL) or ethylbenzene-d_{10} (334 μL). Lastly, the vial was charged with pivalonitrile (1.5 mL, 0.30 M). The vial was then capped, removed from the glovebox and sealed with electrical tape prior to irradiation. The reaction was stirred at 500 rpm

over the series of initial rates timepoints while illuminating with three 34W blue LED lamps (Kessil KSH150B) and two cooling fans (**Supplementary Figure 1**). Time points were acquired every four minutes by quickly removing the vial from the light setup, obtaining a 100 μL aliquot under nitrogen, and then placing the vial back on the light setup. The crude aliquot was passed through a short pad of silica, eluting with $CDCl_3$, and analyzed by ^{19}F NMR relative to 2-fluorobiphenyl as an internal standard.

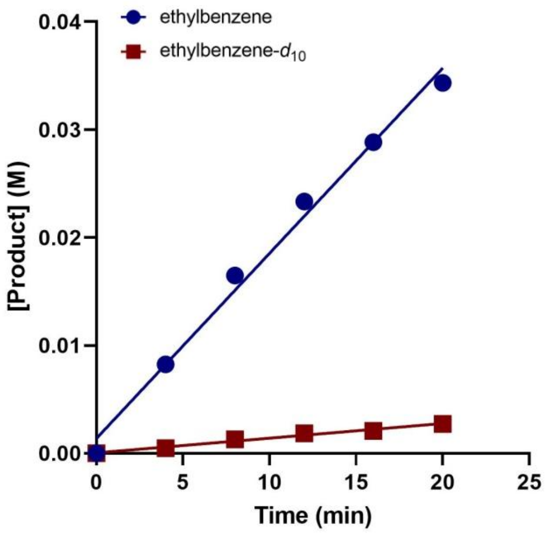

Time (min)	Yield 14 (%, ^{19}F-NMR)	[14] (M)	Yield 61 (%, ^{19}F-NMR)	[61] (M)
0	0	0	0	0
4	2.8	8.2×10^{-3}	0.16	4.8×10^{-4}
8	5.5	1.6×10^{-2}	0.43	1.3×10^{-3}
12	7.8	2.3×10^{-2}	0.62	1.8×10^{-3}
16	9.6	2.9×10^{-2}	0.70	2.1×10^{-3}
20	11.4	3.4×10^{-2}	0.91	2.7×10^{-3}

Parameters	14	61
slope	1.7×10^{-3}	1.4 x 10-4
+/-	8.2×10^{-5}	8.9×10^{-6}
R^2	0.991	0.983
k(A)/*k*(B)	12.1	

Supplementary Figure 33. Trial 1 of initial rate KIE. Plot, data, and linear regression parameters for the product yield versus time in trial 1 for each isotopologue.

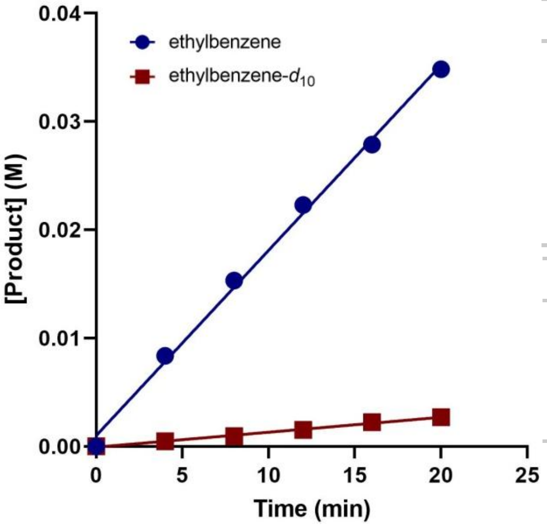

Time (min)	Yield 14 (%, ^{19}F-NMR)	[14] (M)	Yield 61 (%, ^{19}F-NMR)	[61] (M)
0	0	0	0	0
4	2.8	8.4×10^{-3}	0.16	4.7×10^{-4}
8	5.1	1.5×10^{-2}	0.32	9.5×10^{-4}
12	7.4	2.2×10^{-2}	0.51	1.5×10^{-3}
16	9.3	2.8×10^{-2}	0.75	2.2×10^{-3}
20	11.6	3.5×10^{-2}	0.90	2.7×10^{-3}

Parameters	14	61
slope	1.7×10^{-3}	1.4×10^{-4}
+/-	4.9×10^{-5}	4.8×10^{-6}
R^2	0.997	0.995
k(A)/*k*(B)	12.1	

Supplementary Figure 34. Trial 2 of initial rate KIE. Plot, data, and linear regression parameters for the product yield versus time in trial 2 for each isotopologue.

Competition Studies. To a 1-dram oven-dried vial equipped with a Teflon stir bar was added Ir(*p*-F-ppy)$_3$ (1.1 mg, 1.5 μmol, 1.0 mol %) and 1,3-dioxoisoindolin-2-yl acetate **1** (31.0 mg, 0.150 mmol, 1.00 equiv). The vial containing photocatalyst and **1** was then covered with a Kimwipe and pumped into a nitrogen-filled glovebox. To the vial containing photocatalyst and **1** was added triethylamine trihydrofluoride (147 μL, 0.900 mmol, 6.00 equiv) and 5.00 equiv (0.750 mmol) each of C(sp^3)–H partner and C(sp^3)–D partner. Lastly, the vial was charged with pivalonitrile (250 μL, 0.60 M). The vial was then capped, removed from the glovebox and sealed with electrical tape prior to irradiation. The reaction was stirred at 500 rpm for 6 h while illuminating with three 34W blue LED lamps (Kessil KSH150B) and two cooling fans (**Supplementary Figure 1**). The crude reaction mixture was passed through a short pad of silica, eluting with $CDCl_3$, and analyzed by ^{19}F NMR relative to 1-fluoronapthalene (19.4 μL, 0.150 mmol, 1.00 equiv) as an external standard.

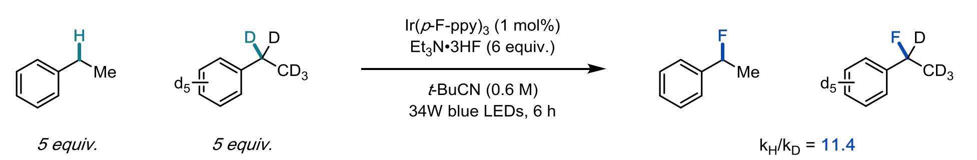

Supplementary Figure 35. Kinetic isotope effect competition studies using ethylbenzene and ethylbenzene-d_{10}. **Run 1**: 25% NMR yield **14**, 2% NMR yield **67**, KIE = 12.5. **Run 2**: 31% NMR yield **14**, 3% NMR yield **67**, KIE = 10.3

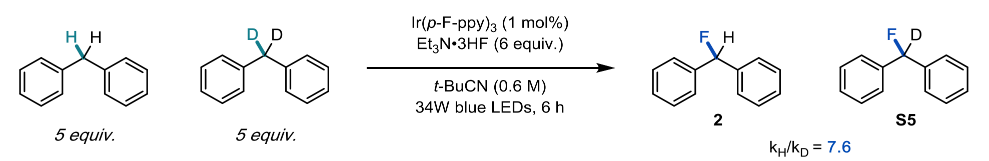

Supplementary Figure 36. Kinetic isotope effect competition studies using diphenylmethane and diphenylmethane-d_2. **Run 1**: 61% NMR yield **2**, 8% NMR yield **S5**, KIE = 7.6. **Run 2**: 77% NMR yield **2**, 10% NMR yield **S5**, KIE = 7.7

The kinetic isotope effect (KIE) studies conducted via initial rates and as intermolecular competition experiments show consistent and large KIEs. The magnitudes of the recorded KIEs are above the classical limit, indicating that light atom tunneling may play a significant role in this mechanism. However, upon further investigation of mechanisms of HAT with methyl radical, we discovered that large KIE values (>7) are expected for methyl radical due to the geometric and vibrational characteristics of the methyl radical intermediate.[57,58] Given that we see a substantial KIE in parallel reactions with ethylbenzene isotopologues (**Supplementary Figure 32**), we would expect the hydrogen atom transfer step to be turnover-limiting in our proposed mechanism. However, if the resulting carbon-centered radical, generated upon HAT with C(sp^3)–H coupling partner, is in equilibrium prior to a turnover-limiting radical oxidation step a KIE could also be observed. The magnitudes of the KIEs observed across two different substrates suggests that this equilibrium HAT mechanism is unlikely.

G. Hammett Studies.

To a 1-dram oven-dried vial equipped with a Teflon stir bar was added Ir(*p*-F-ppy)$_3$ (1.1 mg, 1.5 µmol, 1.0 mol %) and 1,3-dioxoisoindolin-2-yl acetate **1** (31.0 mg, 0.150 mmol, 1.00 equiv). The vial was then covered with a Kimwipe and pumped into a nitrogen-filled glovebox. To the vial containing photocatalyst and **1** was added triethylamine trihydrogenfluoride (147 µL, 0.900 mmol, 6.00 equiv), ethylbenzene (91.9 µL, 0.750 mmol, 5.00 equiv), and the *para*-substituted ethylbenzene (0.750 mmol, 5.00 equiv). Lastly, the vial was charged with pivalonitrile (250 µL, 0.60 M). The vial was then capped, removed from the glovebox and sealed with electrical tape prior to irradiation. The reaction was stirred at 500 rpm for 6 h while illuminating with three 34W blue LED lamps (Kessil KSH150B) and two cooling fans (**Supplementary Figure 1**). The crude reaction mixture was passed through a short pad of silica, eluting with $CDCl_3$, and analyzed by ^{19}F NMR relative to 1-fluoronapthalene (25.8 µL, 0.20 mmol, 1.33 equiv) as an external standard.

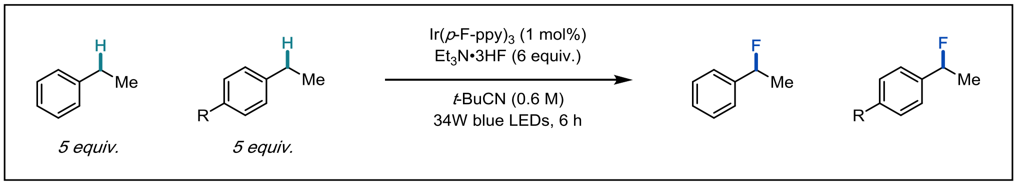

Supplementary Figure 37. Hammett analysis by competition between ethylbenzene (*para*-H) and various ethylbenzene derivatives (*para*-R).

Hammett studies were conducted as competition experiments for C(sp^3)–H fluorination between ethylbenzene and a series of *para*-substituted ethylbenzene derivatives (R = CN, CF_3, Cl, F, Me, OMe, OPh). Competition experiments for each substrate were performed in triplicate. The relative rates of the reactions were determined from the product ratios between secondary benzylic fluorinated products. The logarithm of the selectivity was plotted against both σ_p and σ_p^+ Hammett constants (**Supplementary Figure 31 and 33**).

As discussed in the manuscript, we observed that the fluorination of electron-rich substrates is favored over electron-deficient substrates, lending to a negative ρ value of -0.64. This result is consistent with a mechanism wherein radical oxidation is involved in the product-determining step. Furthermore, we posit that a moderate ρ value is observed as a result of competing electronic effects, wherein HAT with the mildly nucleophilic methyl radical favors electron-poor substituents (polarity matching), while radical oxidation favors electron-rich substrates (carbocation stability). Overall, the sign of the ρ value suggests that radical oxidation dominates selectivity with respect to electronic effects. We do not suggest that HAT via methyl radical is reversible; if this were the case, we would not observe a large primary KIE in the independent rate measurements. Likewise, if oxidative radical-polar crossover were turnover limiting, we would expect a larger negative ρ value—consistent with carbocation formation—and a small, secondary KIE. Instead, we are proposing that radical oxidation occurs after the turnover limiting step and is irreversible. In this case, the competition experiments in the

Hammett analysis could be influenced by the relative rates of radical oxidation even though radical oxidation contributes only minimally to the overall rate of reaction. A similar scenario is seen for KIE studies in catalytic reactions wherein C-H cleavage occurs after the turnover limiting step, but is irreversible (see Hartwig *ACIE* **2012**, *51*, 3066). It is possible that competing pathways from the substrate radical exist with lower energy barriers prior to radical oxidation. In this case, then the magnitude of the radical oxidation energy barrier relative to any side processes would be a dominant factor in terms of product determining selectivity.

In this scenario, we expect a Hammett analysis to reflect the sensitivity of both HAT and radical oxidation to electronic effects. In the case of the electronically varied *para*-substituted ethylbenzene series presented in this work, we would expect a small electronic bias favoring electron-deficient substrates for HAT with methyl radical. However, radical oxidation is expected to be more sensitive to electronic effects, strongly favoring electron-rich substrates due to enhanced carbocation stability. Combined, radical oxidation is expected to dominate the product distribution. Indeed, in Hammett analysis with methyl radical, a negative ρ value with a small magnitude is observed (-0.64), consistent with this analysis.

By contrast, we would expect an electrophilic methoxy radical to show greater preference for abstracting hydrogen atoms from electron-rich substrates, as this radical is polarity matched for electron-rich substrates over electron-deficient substrates. In addition to preferential reactivity for electron-rich substrates in the HAT event, radical oxidation in this case should also demonstrate favorable selectivity for electron-rich substrates as a result of carbocation stability. Thus, in Hammett analysis with the methoxy radical, we observe an additive selectivity effect, wherein both HAT and radical oxidation are both aligned to favorably select for more electron-rich substrates, and an enhanced ρ value of -1.36 is observed (**Supplementary Figure 36**). The observation that these two abstractors afford different ρ values is additional evidence that HAT, not radical oxidation (which is independent of the abstractor) is turnover limiting.

As part of our Hammett analysis, we also examined a competition between ethylbenzene and methyl 4-ethylbenzoate (*para*-CO_2Me). However, we removed the data point attributed to this substrate from the final Hammett plot. We hypothesize that this particular substrate may experience a greater resonance contribution from the ester substituent than captured by Hammett or Hammett-Brown constants, and therefore demonstrates selectivity higher than expected in competition with ethylbenzene.

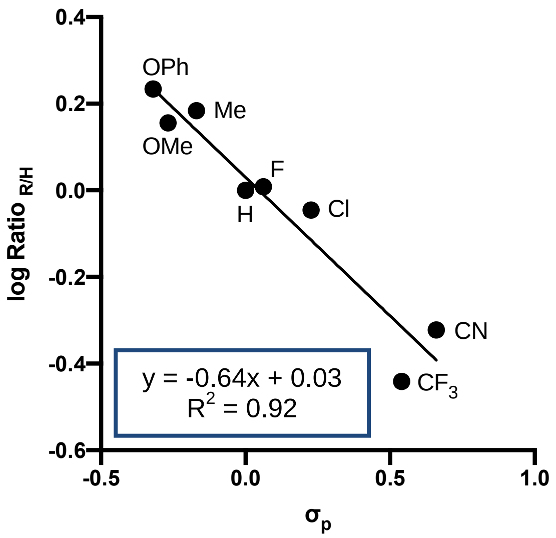

Supplementary Figure 38. Hammett correlation with abstractor **1** using Hammett constants.[59,60]

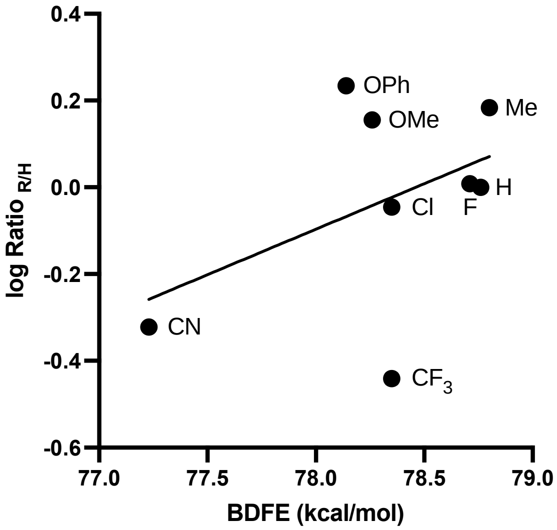

Supplementary Figure 39. Hammett analysis and correlation of selectivity with computed BDFE for a series of ethylbenzene derivatives.[56]

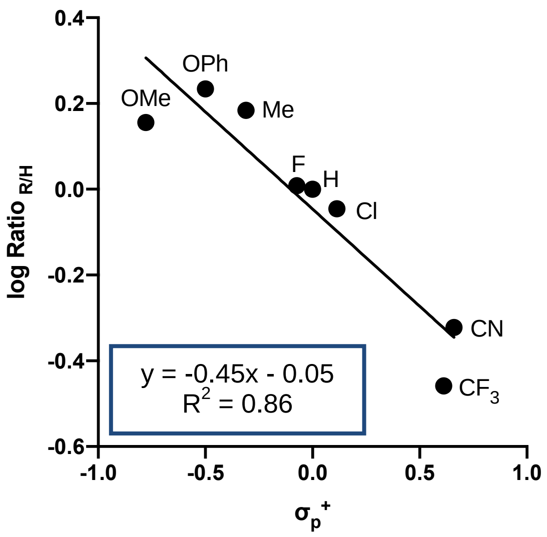

Supplementary Figure 40. Hammett correlation with abstractor **1** using Hammett-Brown constants. Hammett-Brown constant for -OPh is the reported calculated value.[59,60]

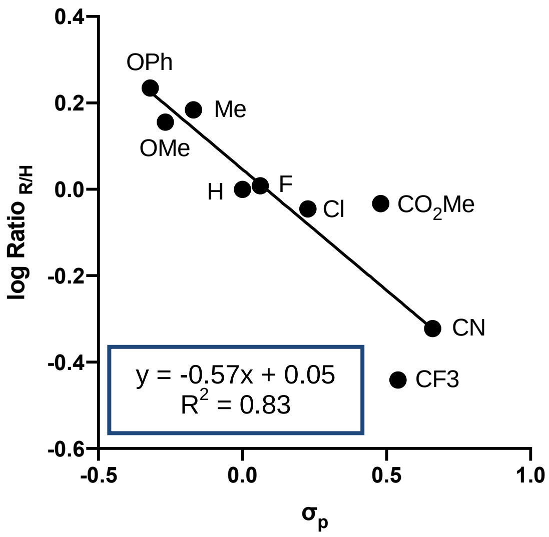

Supplementary Figure 41. Hammett correlation with abstractor **1**, including *para*-CO_2Me ethylbenzene data point, using Hammett constants.[59,60]

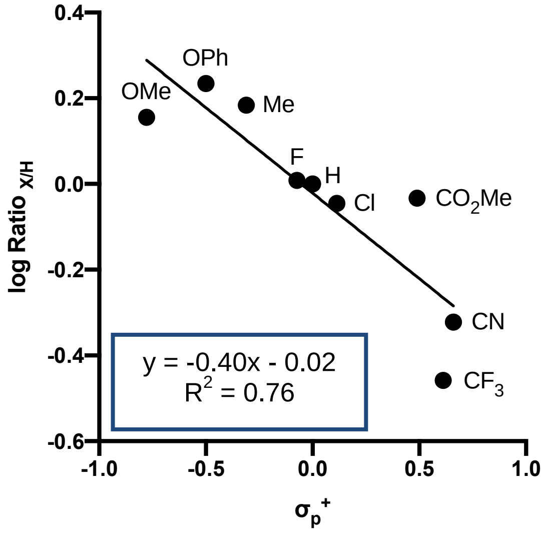

Supplementary Figure 42. Hammett correlation with abstractor **1**, including *para*-CO_2Me ethylbenzene data point, using Hammett-Brown constants.[59,60]

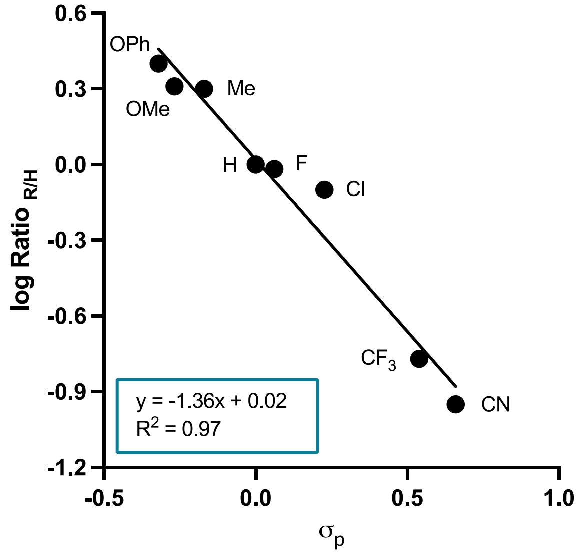

Supplementary Figure 43. Hammett correlation with abstractor **3** using Hammett constants.[59,60]

VIII. Electrophilic versus Nucleophilic Fluorination

General procedure for electrophilic fluorination comparisons:

Reaction setup (0.150 mmol scale): To a 1-dram oven-dried vial, equipped with a Teflon stir bar, was added 9-fluorenone (1.40 mg, 7.50 μmol, 5.00 mol %) and Selectfluor™ or NFSI (0.300 mmol, 2.00 equiv). The vial and its contents were then covered with a Kimwipe and pumped into a nitrogen-filled glovebox. To the reaction vial was then added C(sp^3)–H partner (0.150 mmol, 1.00 equiv) and acetonitrile (1.9 mL, 0.08 M). The vial was capped, removed from the glovebox and sealed with electrical tape prior to irradiation. The reaction was stirred at 800 rpm for 6 h while illuminating with three 34W blue LED lamps (Kessil KSH150B) and two cooling fans (**Supplementary Figure 1**). The crude reaction mixture was passed through a short pad of silica, eluting with $CDCl_3$, and analyzed by ^{19}F NMR relative to 1-fluoronapthalene (19.4 μL, 0.150 mmol, 1.00 equiv) as an external standard.

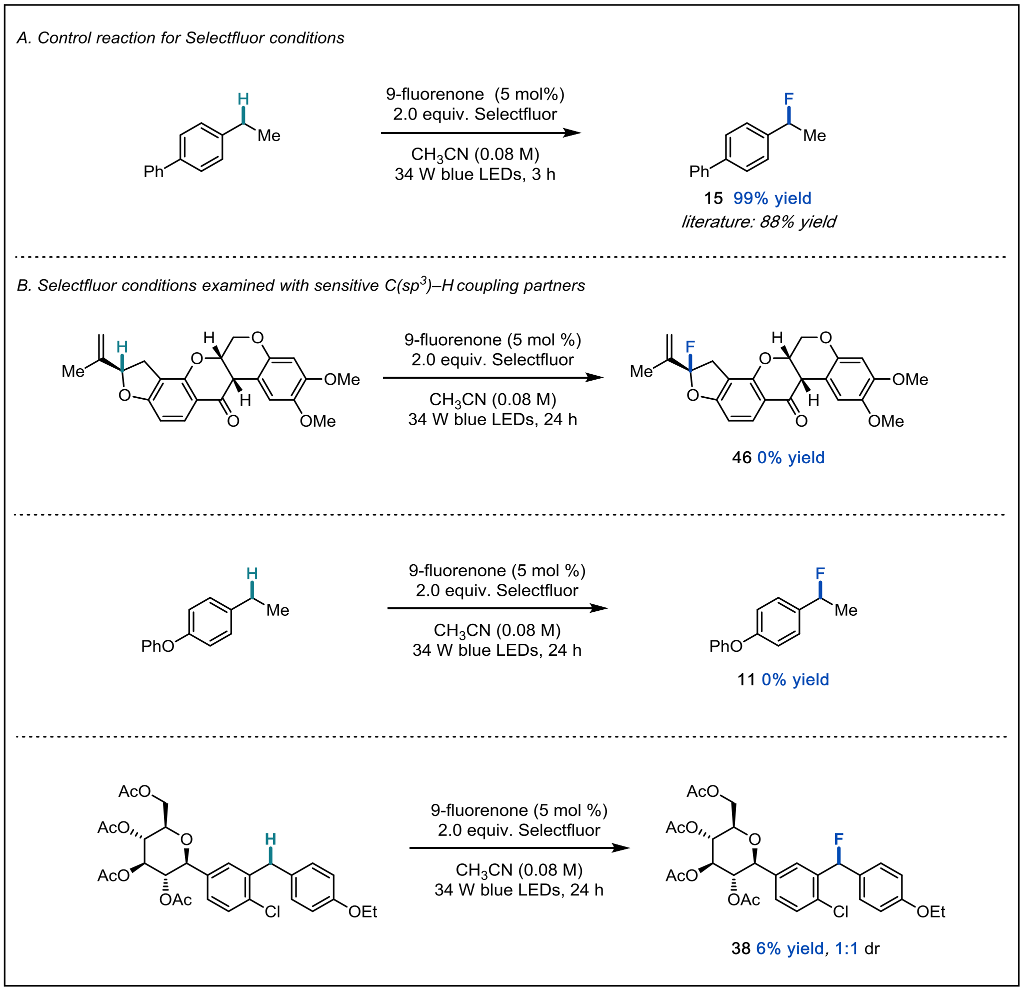

Supplementary Figure 44. C(sp^3)–H fluorination with Selectfluor.

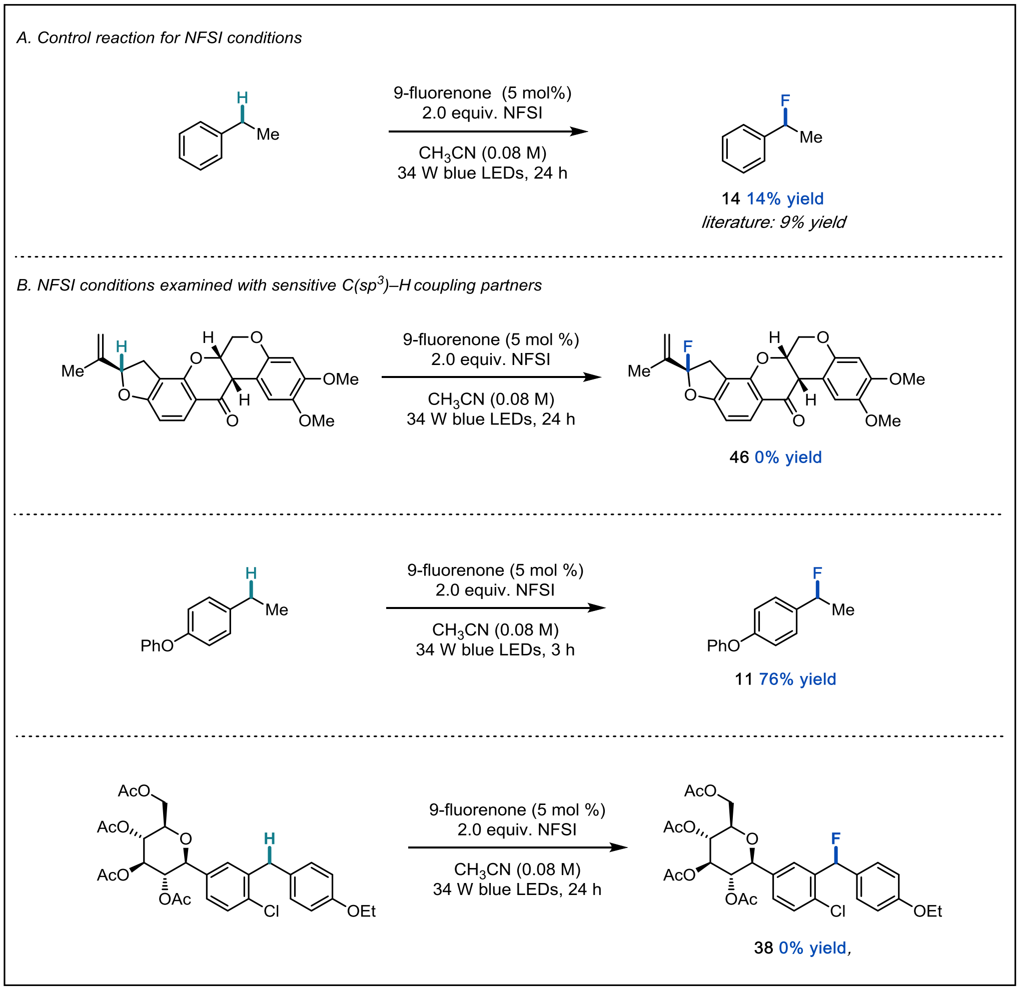

Supplementary Figure 45. C(sp^3)–H fluorination with NFSI.

Head-to-head comparisons for select substrates using electrophilic C(sp^3)–H fluorination conditions were performed to demonstrate the complementarities and opportunities afforded by pursuing a nucleophilic approach. We performed case studies with rotenone, a dapagliflozin derivative, and *p*-OPh ethylbenzene using state-of-the-art electrophilic C(sp^3)–H fluorination conditions reported by Chen and coworkers.[61] These substrates contain electron-rich functionality and therefore, are expected to be susceptible to degradation by electrophilic fluorinating reagents. Indeed, upon subjecting these substrates to reaction conditions wherein Selectfluor is the fluorinating reagent—we observed little to no fluorination and the generation of several degradation side products (**Supplementary Figure 44**). Upon subjecting these substrates to reaction conditions with NFSI, a milder reagent than Selectfluor, we observed that *p*-OPh ethylbenzene is now tolerated in 76% yield. However, no fluorination and the generation of several degradation side products is observed in the attempted syntheses of **46** and **38** (**Supplementary Figure 45**). By comparison, under the standard nucleophilic fluorinating conditions reported in **Figure 2**, the fluorination of rotenone

proceeds to deliver **46** in 33% yield, the fluorination of *p*-OPh ethylbenzene delivers **11** in 52% yield, and the fluorination of the dapagliflozin derivative delivers **38** in 44% yield.

IX. Calculation of Bond Dissociation Enthalpies

C(sp^3)–H Bond Strength Calculations. Calculations were performed on Gaussian 16 Revision A.03 software suite[62] using the M06-2X/def2-TZVP level of theory with the SMD (acetonitrile) solvation model. All frequency calculations gave no imaginary frequencies. Cartesian coordinates for solution phase optimized geometries are contained as .xyz files in the accompanying zip file. BDFEs were computed according to: John, P. C. St., Guan, Y., Kim, Y., Kim, S. & Paton, R. S. Prediction of organic homolytic bond dissociation enthalpies at near chemical accuracy with sub-second computational cost. *Nat. Commun.* **11**, 2328 (2020). In our experience, different levels of theory give slightly different ordering of the compounds by BDFE. This is due to energetic differences that are small relative to the error associated with DFT. However, all levels of theory evaluated gave similarly poor correlations and do not affect the conclusions drawn.

Entry	Molecule	Electronic Energies	Enthalpies	Free Energies
1	H•	-0.497749	-0.495389	-0.508404
2	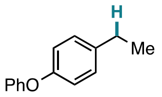	-617.118418	-616.861818	-616.917249
3	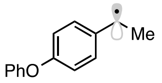	-616.470960	-616.228507	-616.279209
4	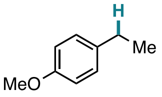	-425.378801	-425.177708	-425.223962
5	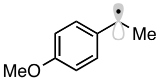	-424.731459	-424.544429	-424.590835
6	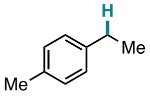	-350.162656	-349.967792	-350.013435
7	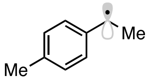	-349.515128	-349.334288	-349.379448
8	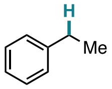	-310.852116	-310.686414,	-310.726953
9	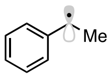	-310.204235	-310.052587	-310.093033

Supplementary Table 1. Thermodynamic data for substituted ethylbenzenes reported in Hartrees.

Entry	Molecule	Electronic Energies	Enthalpies	Free Energies
10	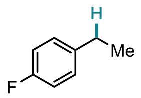	-410.105661	-409.947425	-409.989978
11	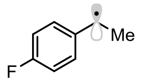	-409.457586	-409.313407	-409.356139
12	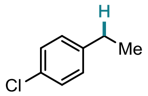	-770.457535	-770.300200	-770.344020
13	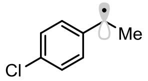	-769.810034	-769.666655	-769.710759
14	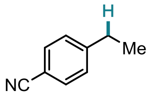	-403.107876	-402.941611	-402.986445
15	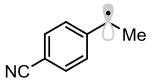	-402.462060	-402.309589	-402.354969
16	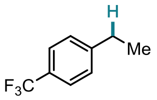	-647.946378	-647.772277	-647.822754
17	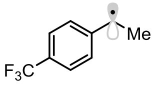	-647.299027	-647.138841	-647.189490

Supplementary Table 2. Thermodynamic data for substituted ethylbenzenes reported in Hartrees (continued).

Entry	Molecule	Electronic Energies	Enthalpies	Free Energies
1	H•	-0.497749	-0.495389	-0.508404
2	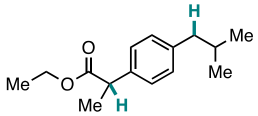	-656.671015	-656.368337	-656.430474
3	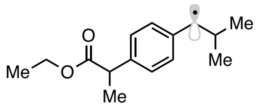	-656.024256	-655.735581	-655.798074
4	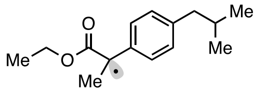	-656.028420	-655.739061	-655.801413

Supplementary Table 3. Thermodynamic data for ibuprofen and derived radicals reported in Hartrees.

Entry	Molecule	Electronic Energies	Enthalpies	Free Energies
1	**H•**	**-0.497749**	**-0.495389**	**-0.508404**
2	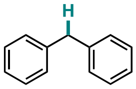	**-502.589062**	**-502.366795**	**-502.415752**
3	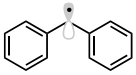	**-501.947445**	**-501.738389**	**-501.786415**
4	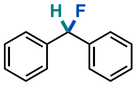	**-601.841784**	**-601.626389**	**-601.677943**
5	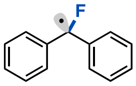	**-601.201325**	**-600.999152**	**-601.049075**

Supplementary Table 4. Thermodynamic data for diphenylmethane and fluorodiphenylmethane and their derived radicals reported in Hartrees.

Entry	Molecule	BDFE (kcal/mol)	Entry	Molecule	BDFE (kcal/mol)
1	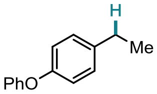	78.14	7	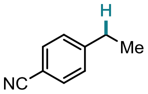	77.23
2	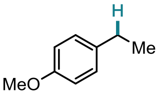	78.26	8	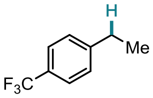	78.35
3	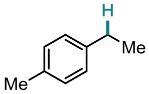	78.80	9	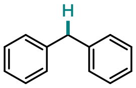	75.89
4	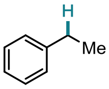	78.76	10	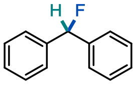	75.59
5	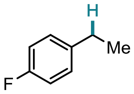	78.71	11	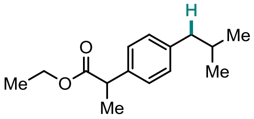	77.81
6	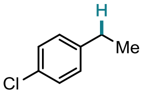	78.35	12	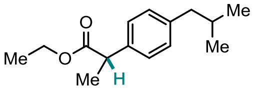	75.71

Supplementary Table 5. Calculated bond dissociation free energies.

XI. NMR Spectra
Spectra of Abstractors and Substrates

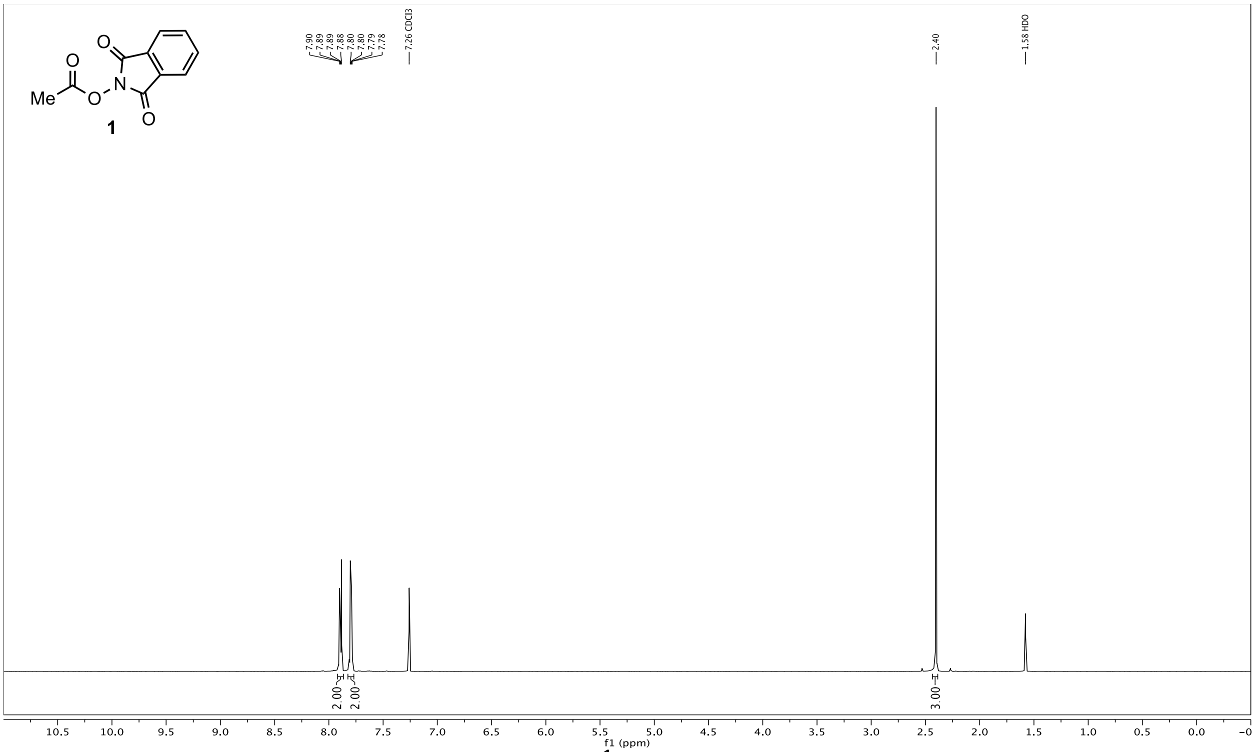

Supplementary Figure 46. ^{1}H NMR (500 MHz, $CDCl_3$) of **1**.

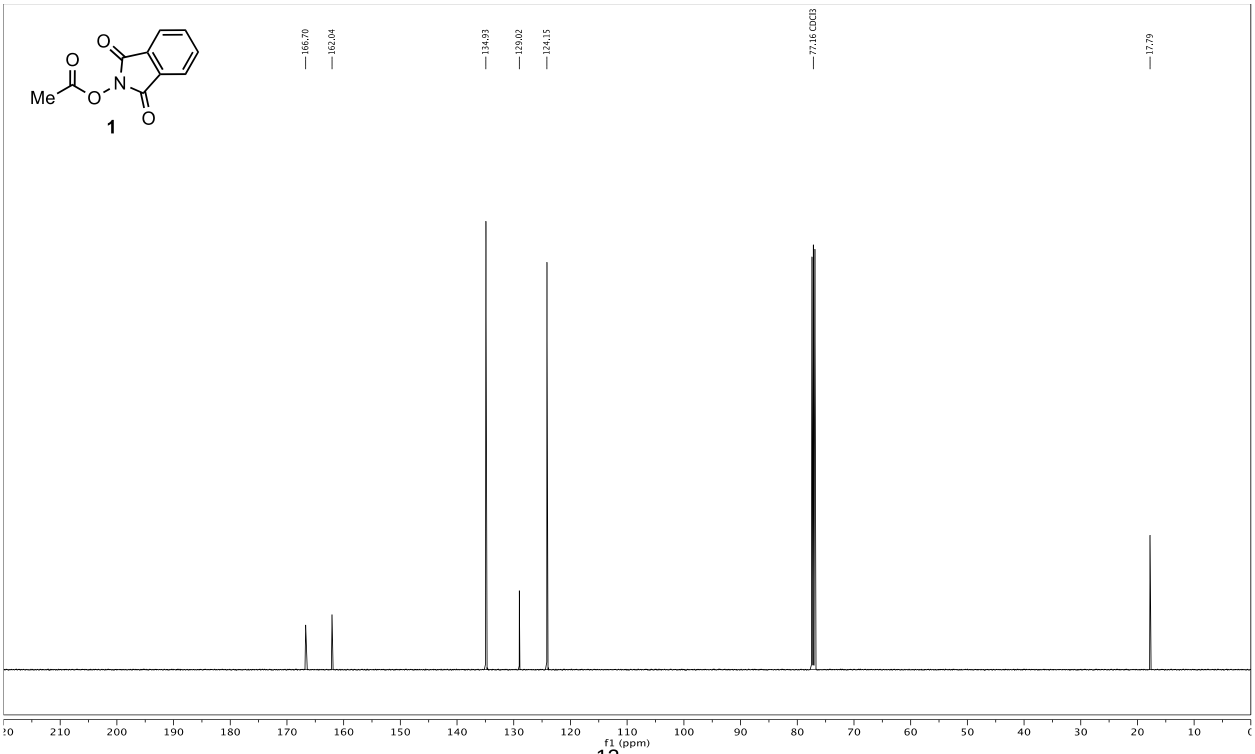

Supplementary Figure 47. ^{13}C NMR (126 MHz, $CDCl_3$) of **1**.

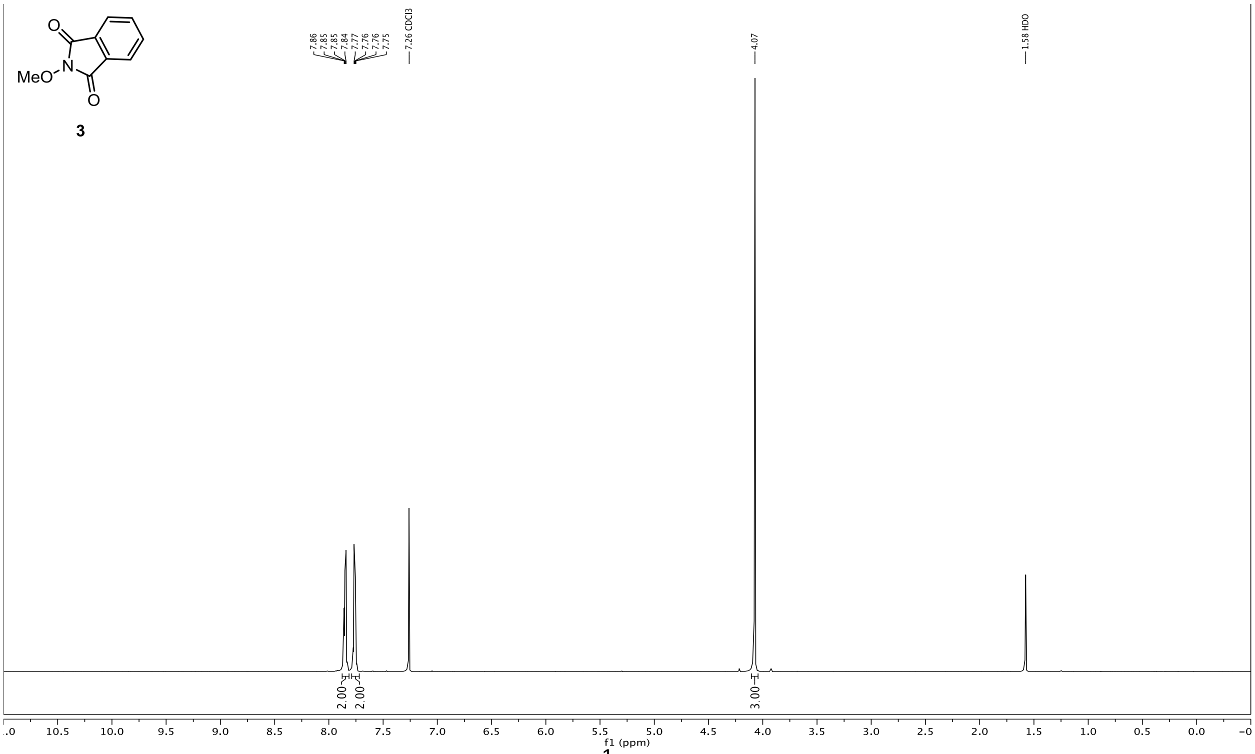

Supplementary Figure 48. ^{1}H NMR (500 MHz, $CDCl_3$) of **3**.

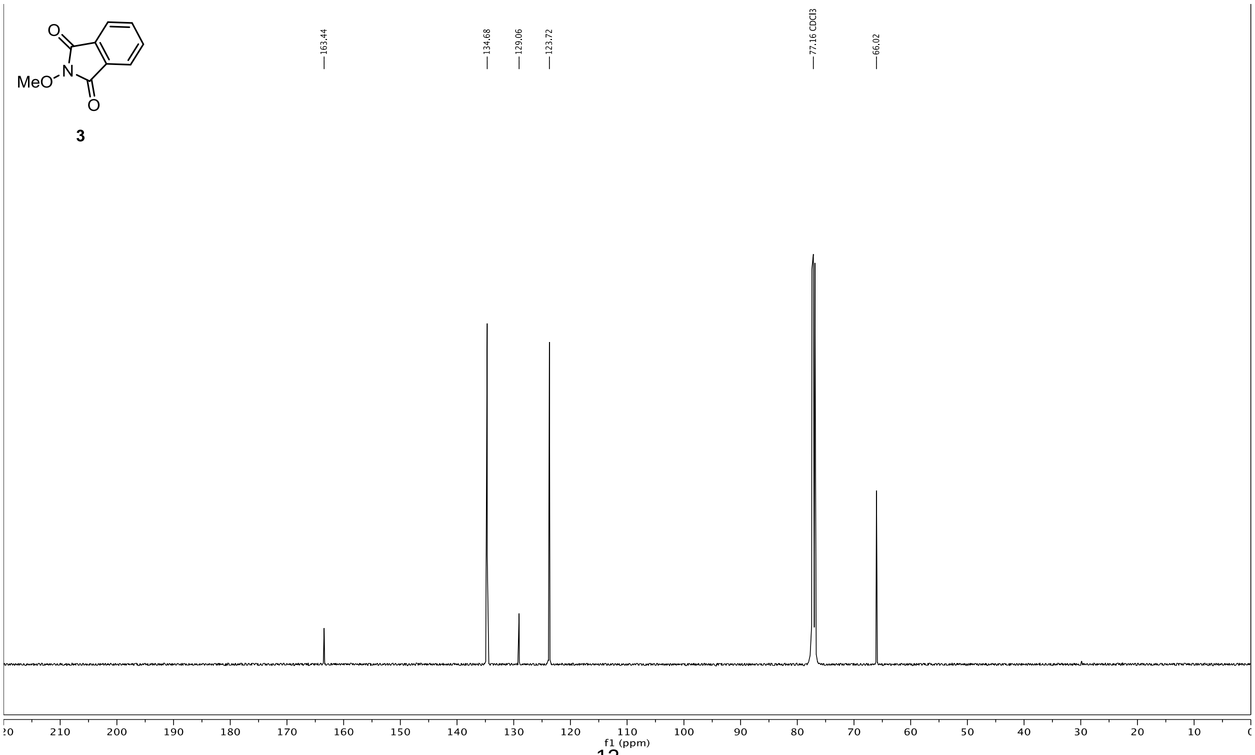

Supplementary Figure 49. ^{13}C NMR (126 MHz, $CDCl_3$) of **3**.

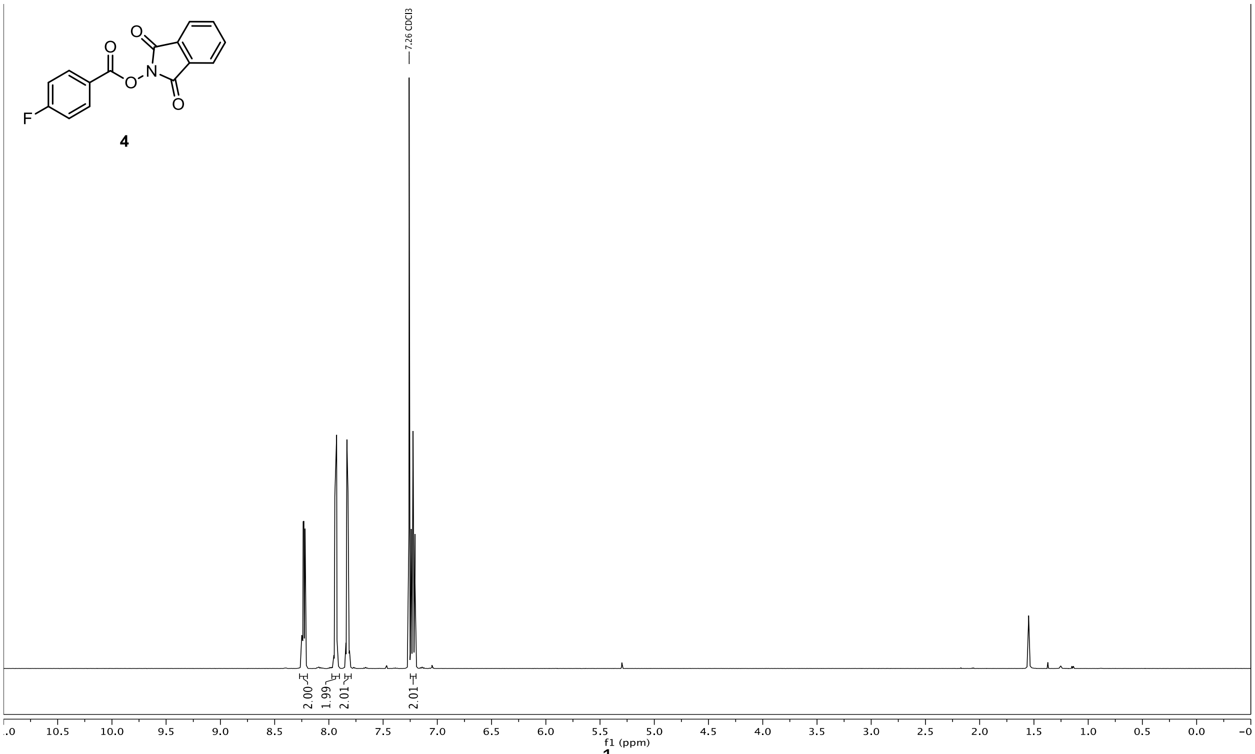

Supplementary Figure 50. ^{1}H NMR (500 MHz, $CDCl_3$) of **4**.

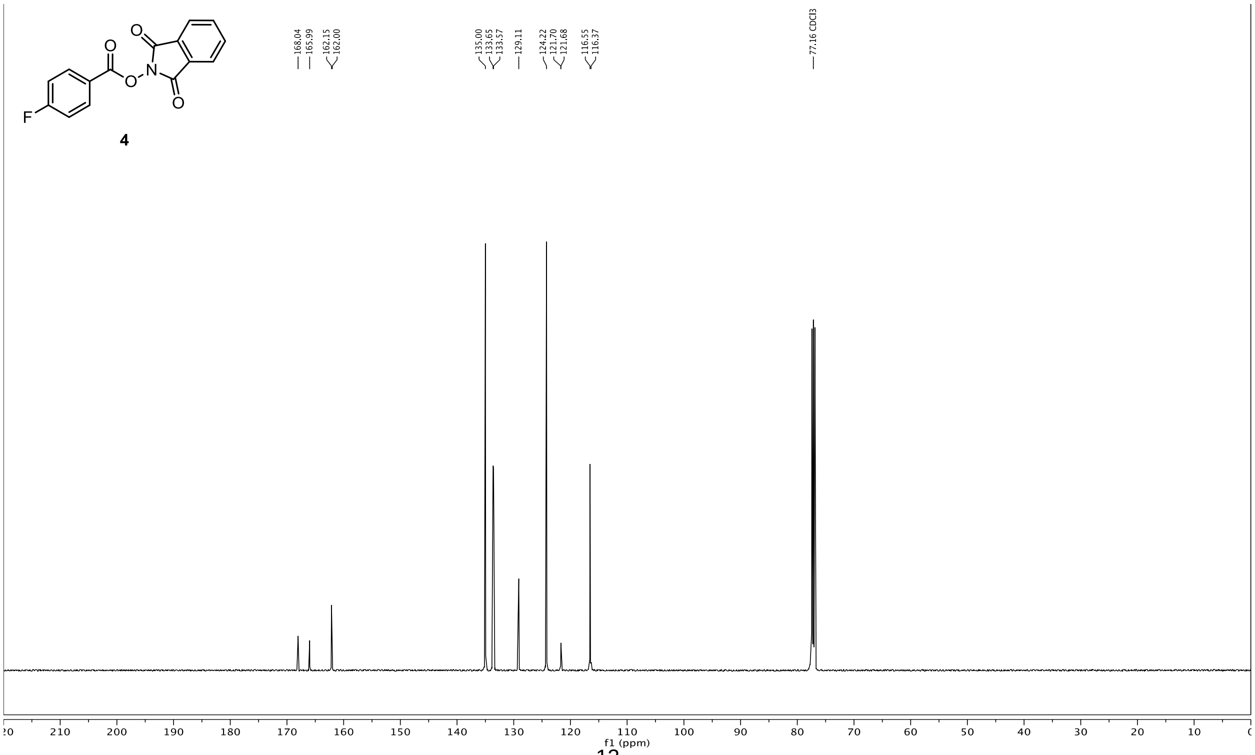

Supplementary Figure 51. ^{13}C NMR (126 MHz, $CDCl_3$) of **4**.

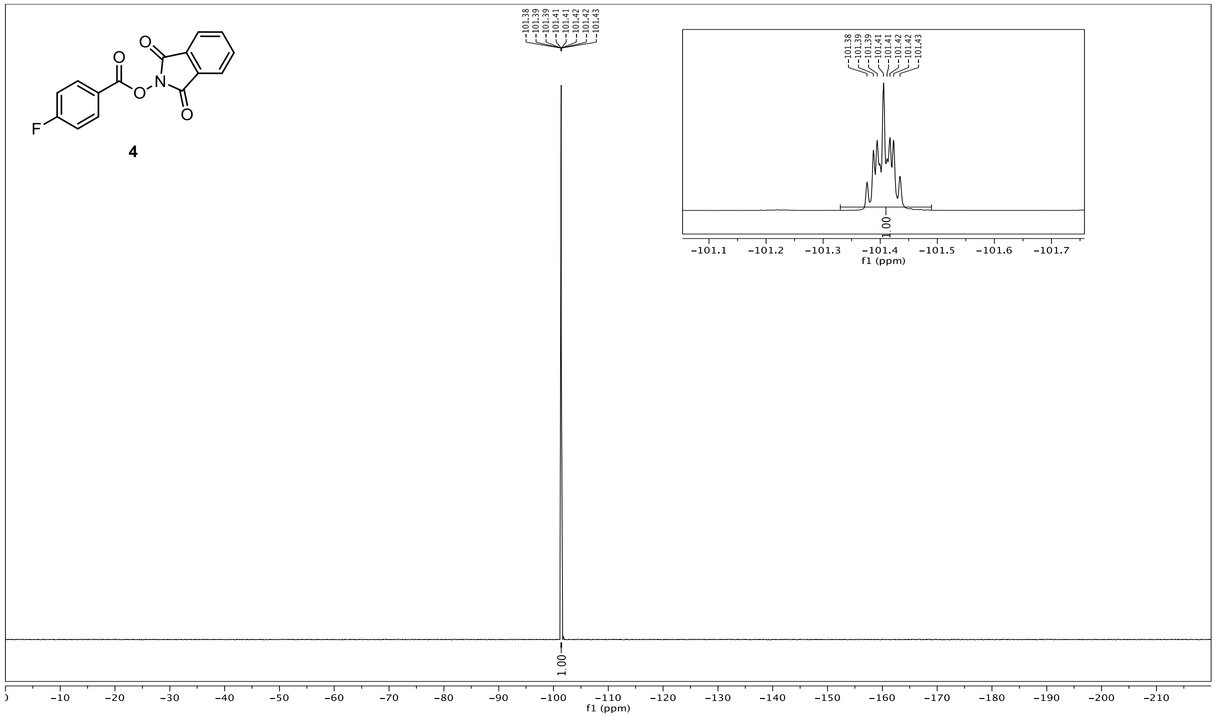

Supplementary Figure 52. ^{19}F NMR (282 MHz, $CDCl_3$) of **4**.

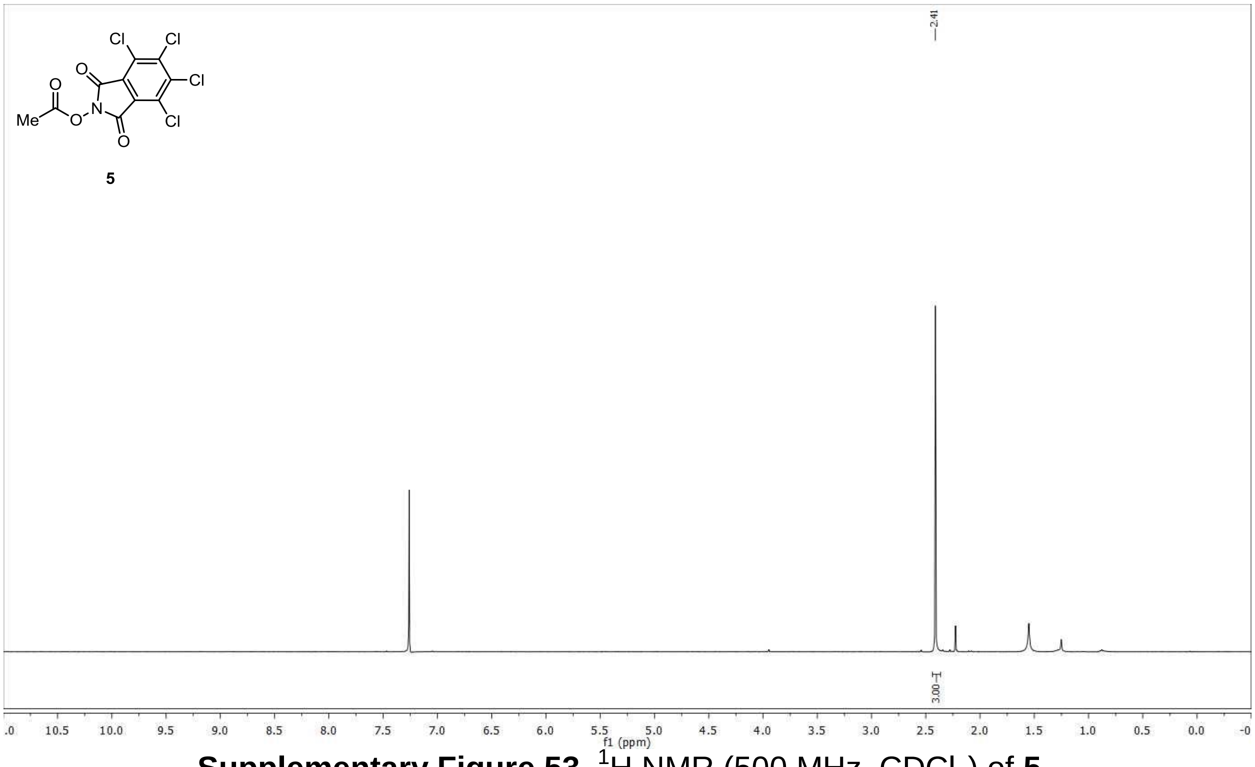

Supplementary Figure 53. ^{1}H NMR (500 MHz, $CDCl_3$) of **5**.

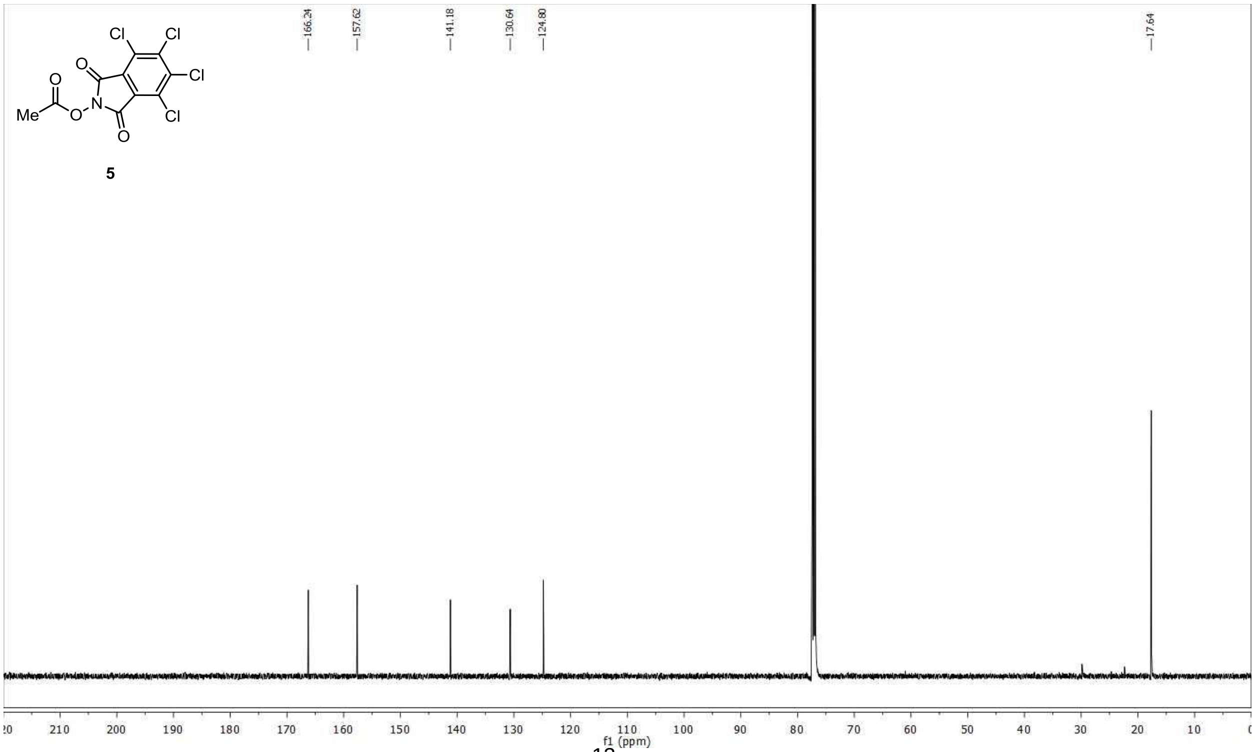

Supplementary Figure 54. ^{13}C NMR (126 MHz, $CDCl_3$) of **5**.

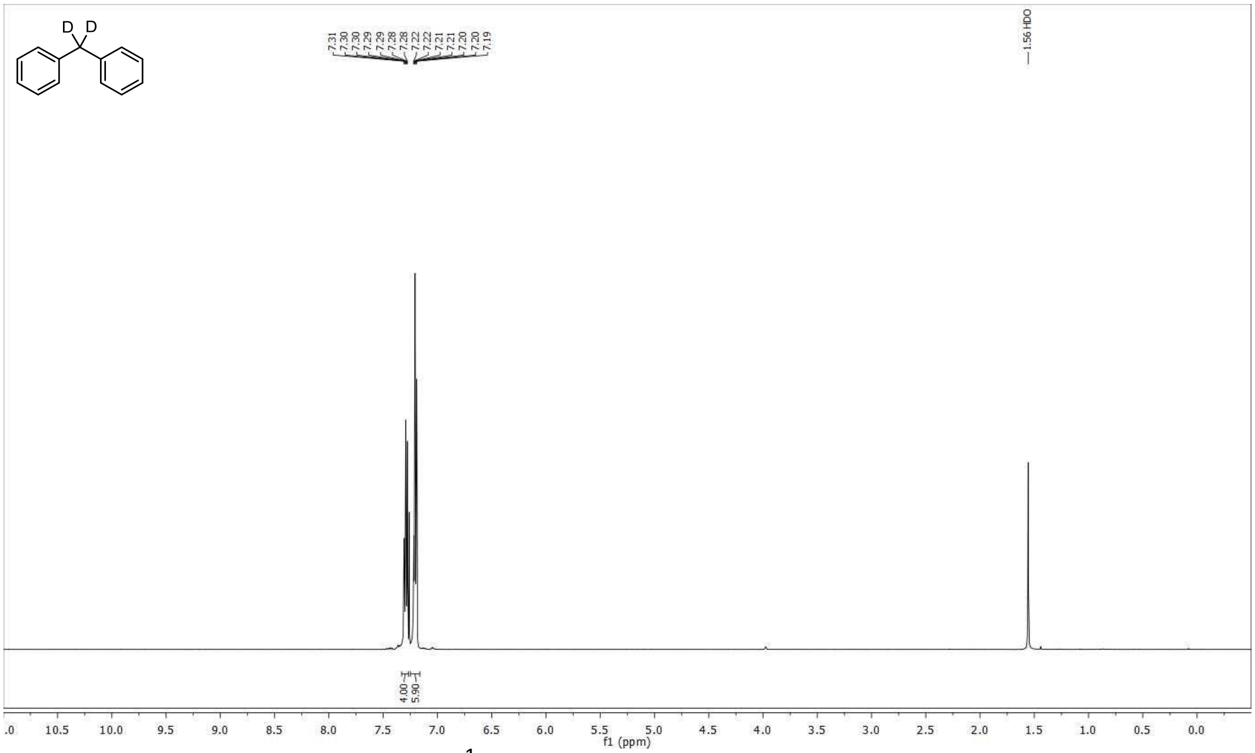

Supplementary Figure 55. ^{1}H NMR (500 MHz, $CDCl_3$) of diphenylmethane-d_2.

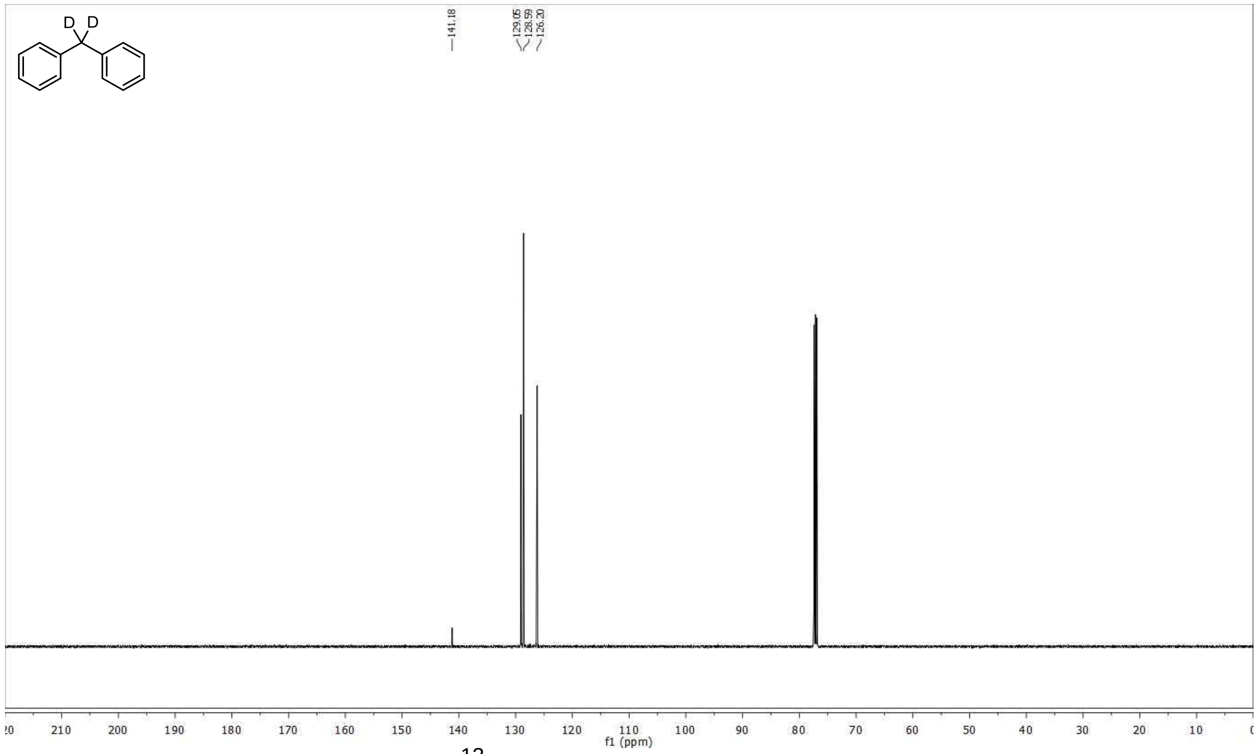

Supplementary Figure 56. ^{13}C NMR (126 MHz, $CDCl_3$) of diphenylmethane-d_2.

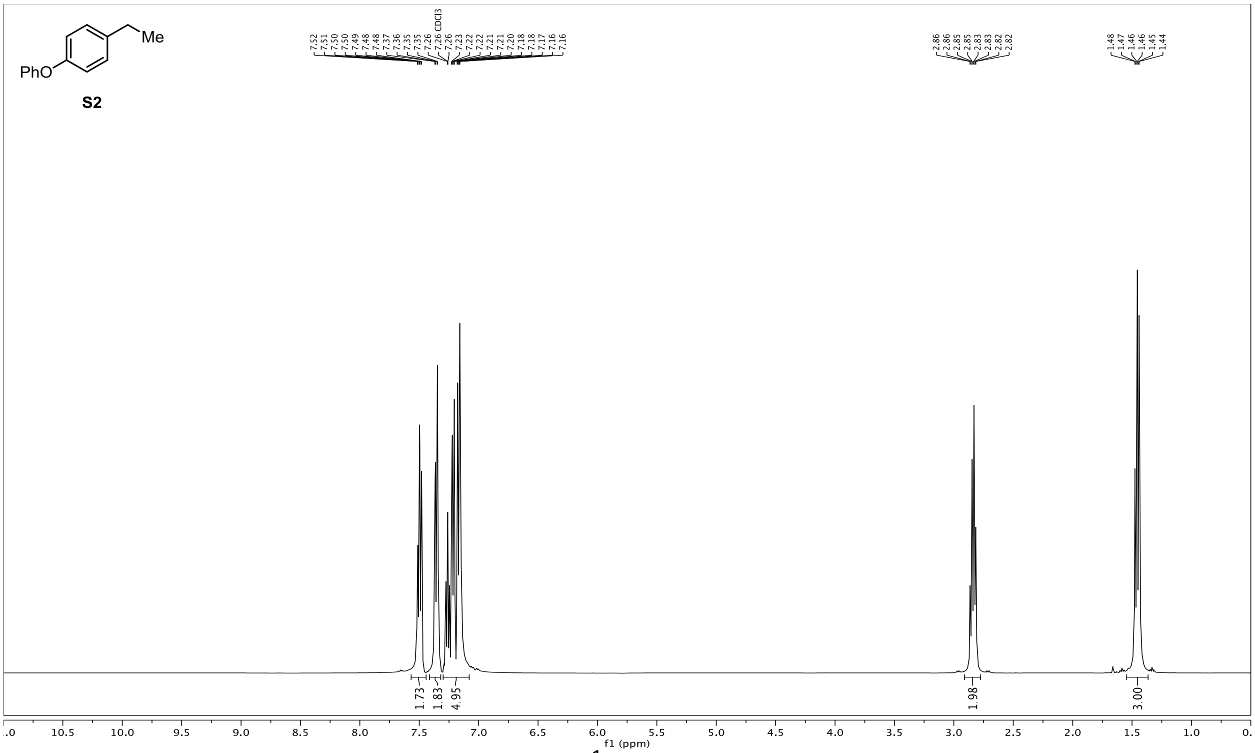

Supplementary Figure 57. ^{1}H NMR (500 MHz, $CDCl_3$) of **S2**.

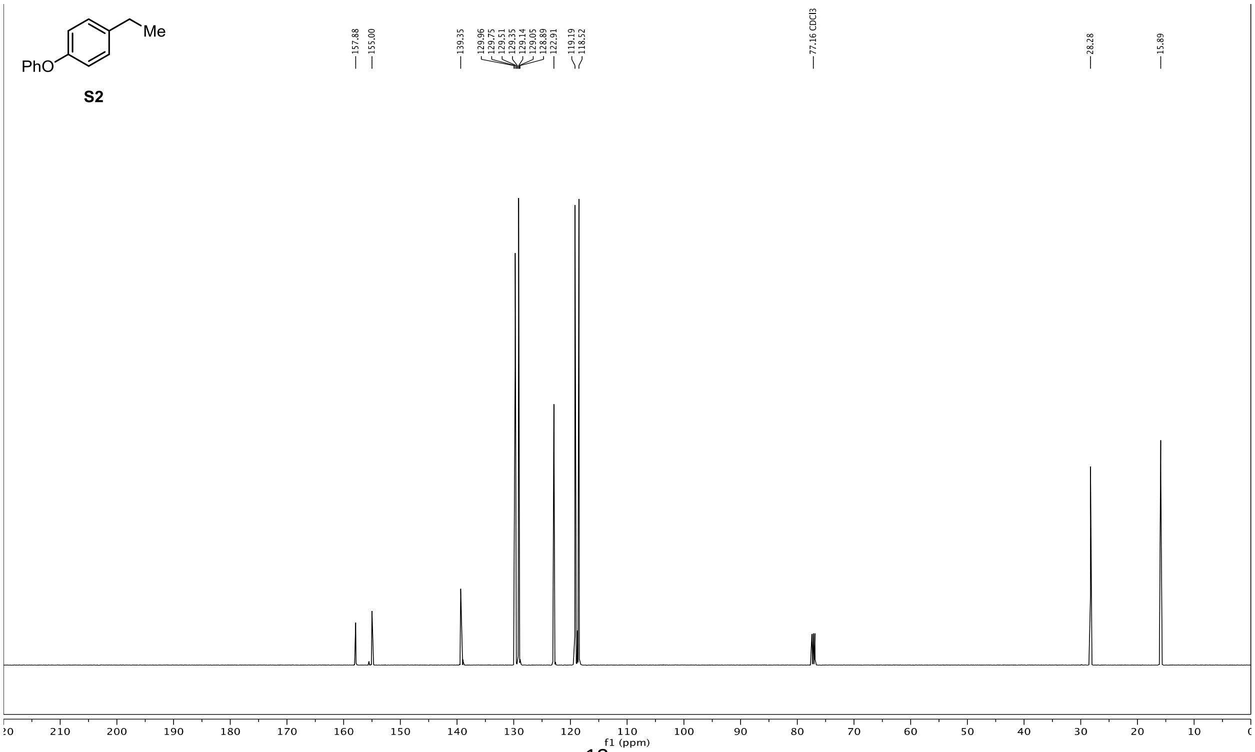

Supplementary Figure 58. ^{13}C NMR (126 MHz, $CDCl_3$) of **S2**.

Spectra of Fluorinated Products

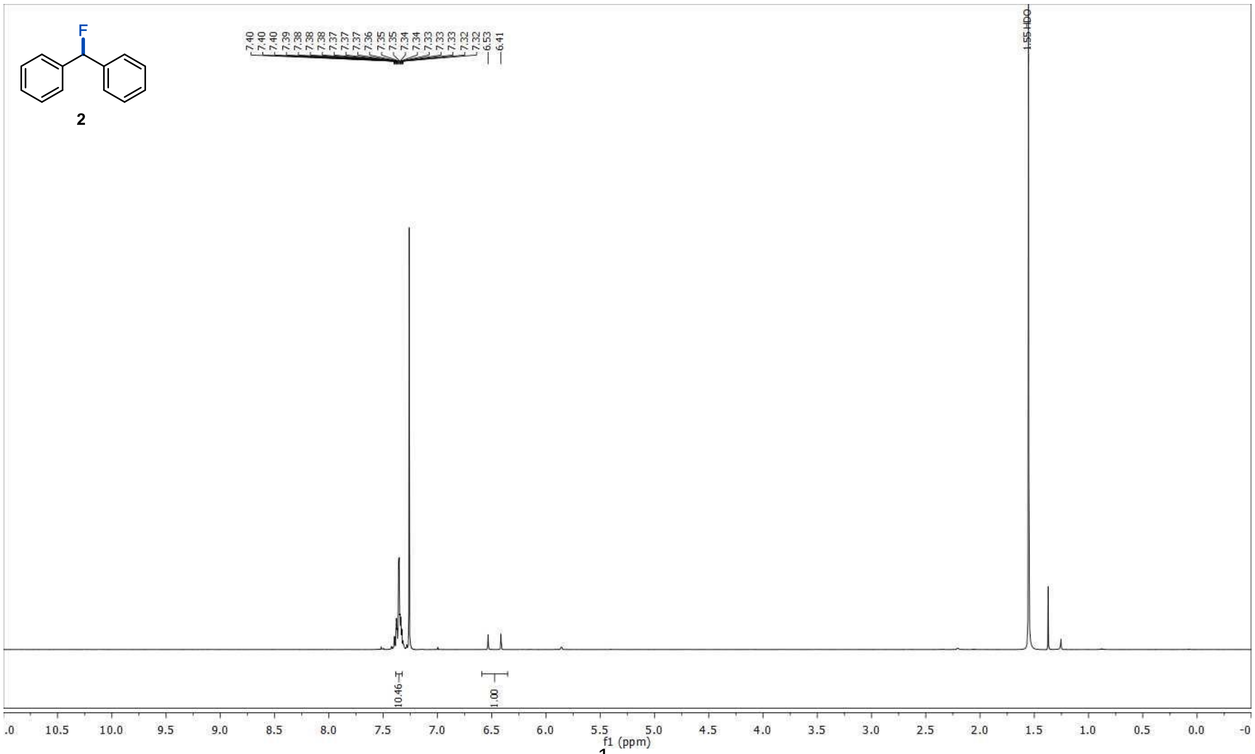

Supplementary Figure 59. ^{1}H NMR (500 MHz, $CDCl_3$) of **2**.

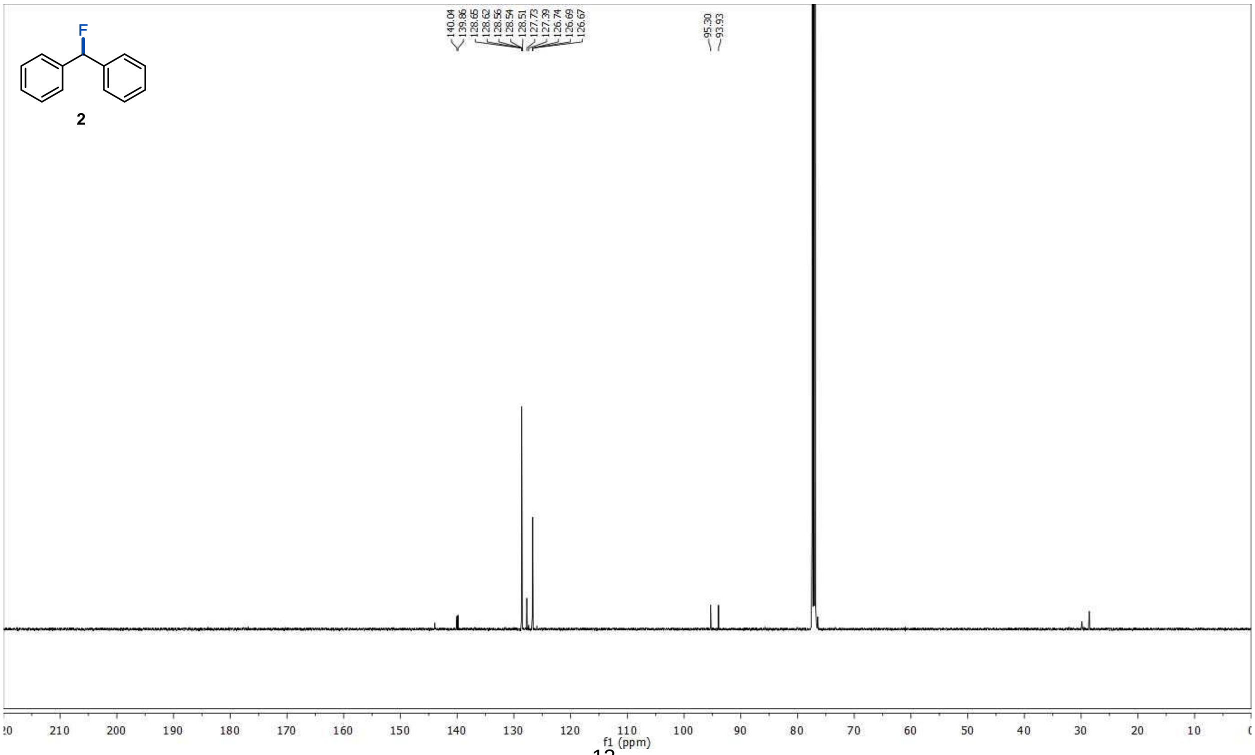

Supplementary Figure 60. ^{13}C NMR (126 MHz, $CDCl_3$) of **2**.

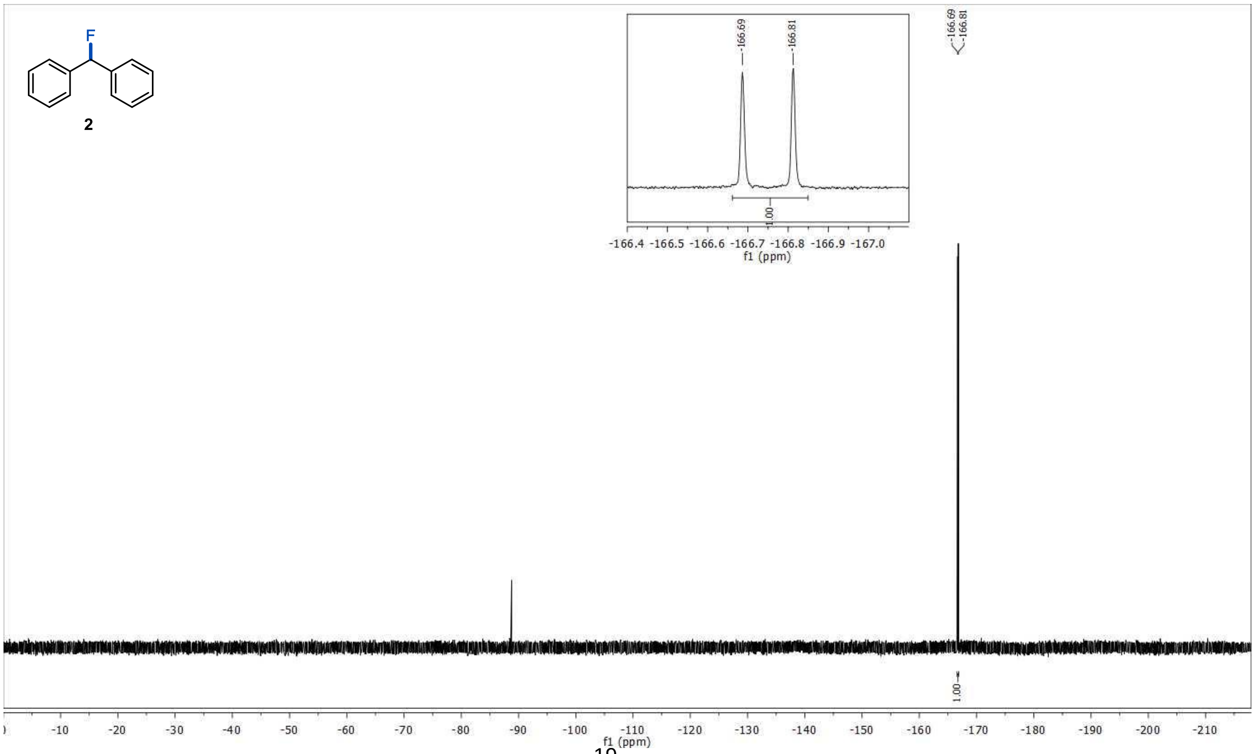

Supplementary Figure 61. ^{19}F NMR (282 MHz, $CDCl_3$) of **2**.

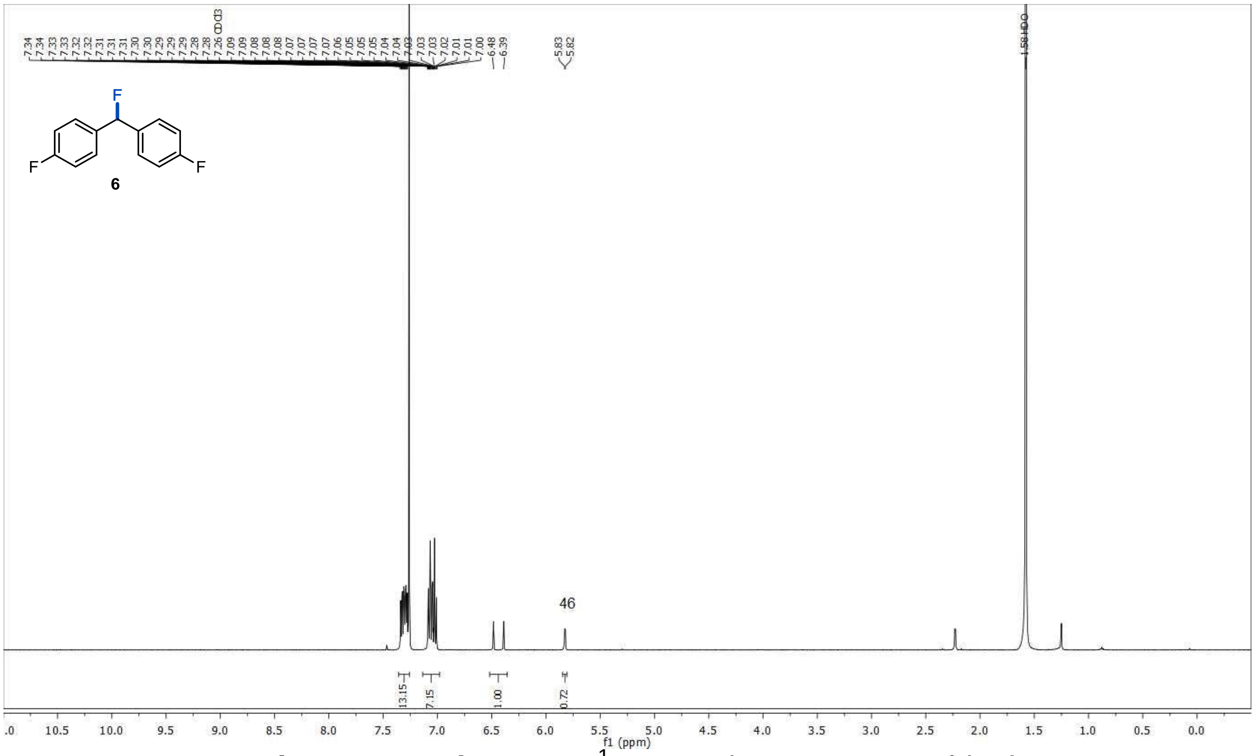

Supplementary Figure 62. ^{1}H NMR (500 MHz, $CDCl_3$) of **6**.

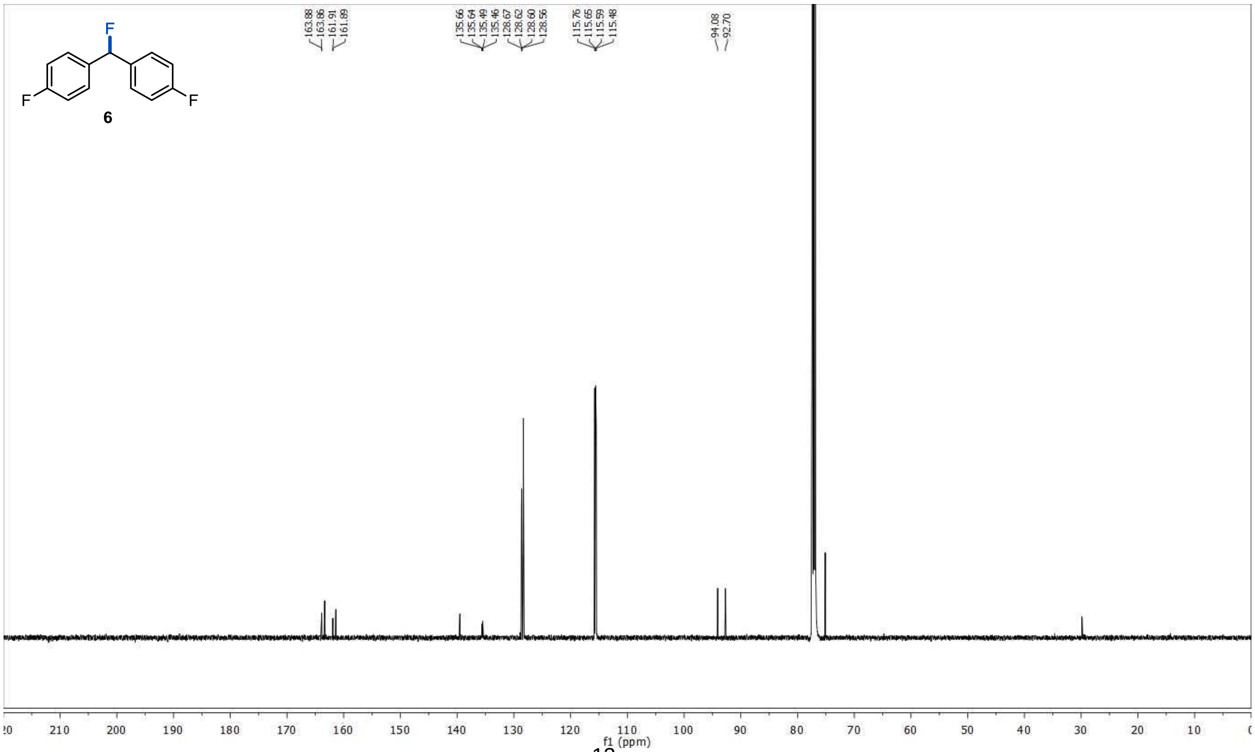

Supplementary Figure 63. ^{13}C NMR (126 MHz, $CDCl_3$) of **6**.

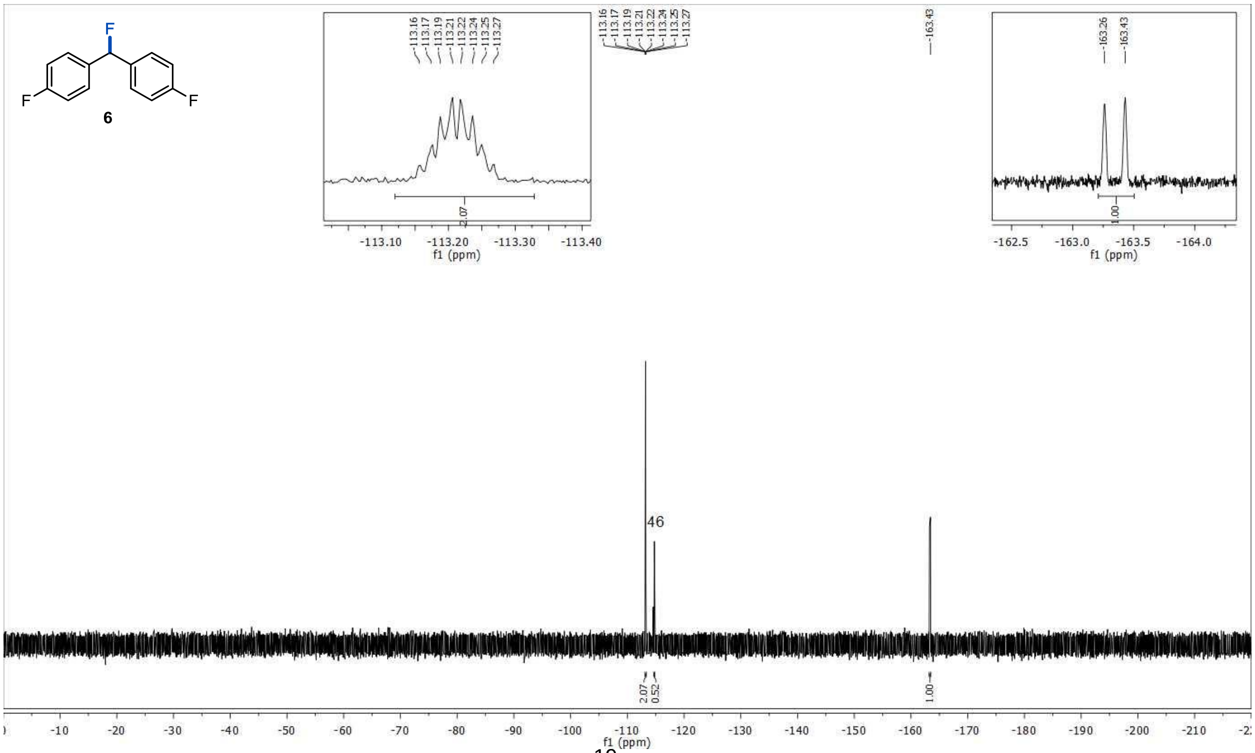

Supplementary Figure 64. ^{19}F NMR (282 MHz, $CDCl_3$) of **6**.

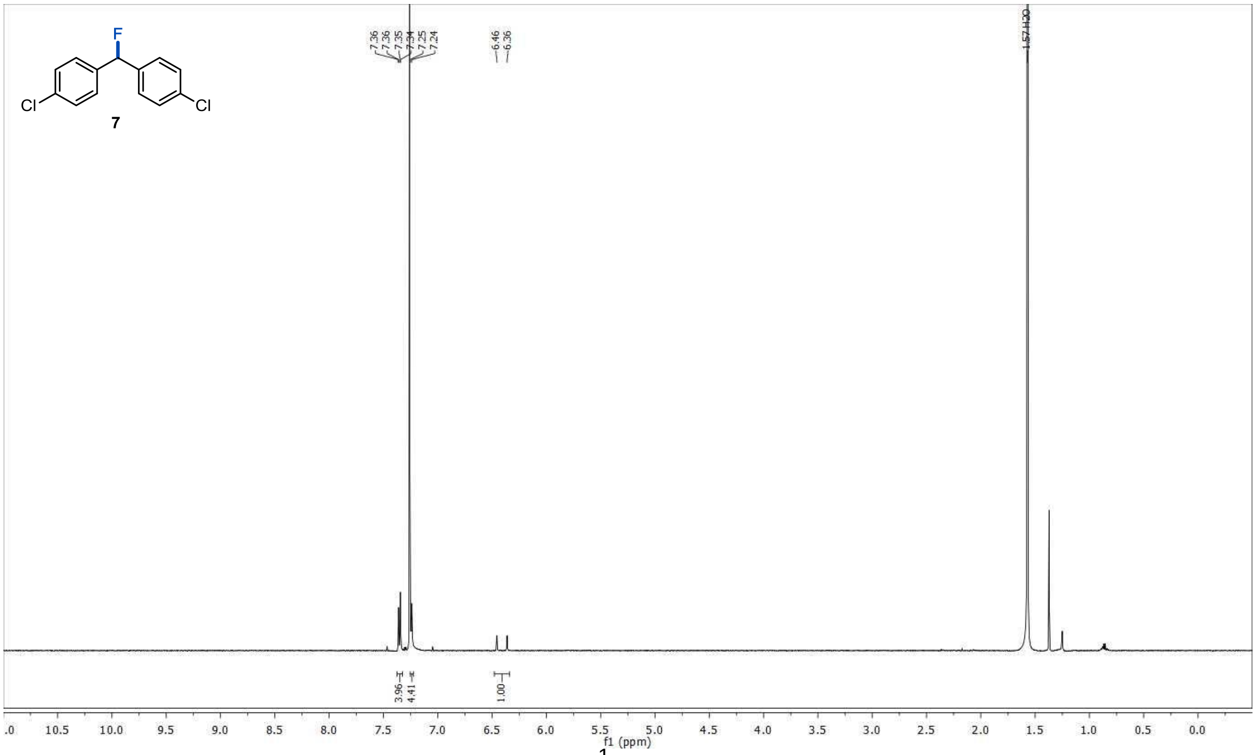

Supplementary Figure 65. ^{1}H NMR (500 MHz, $CDCl_3$) of **7**.

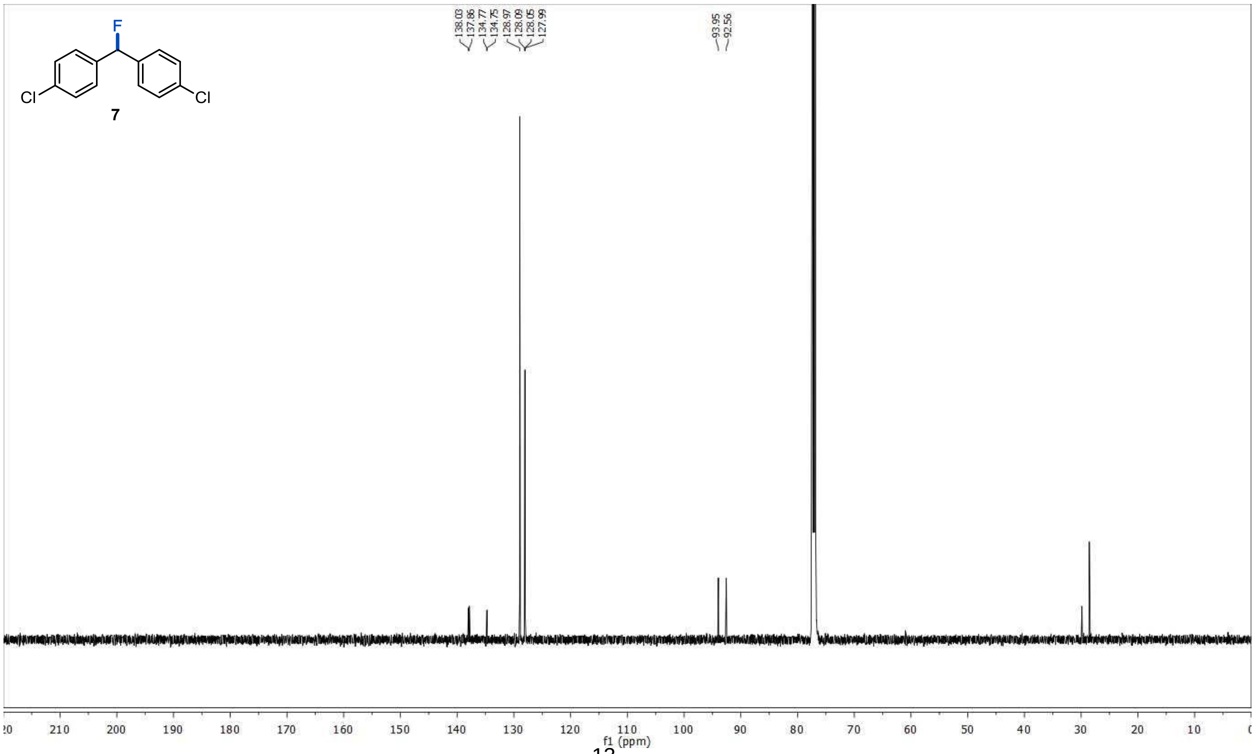

Supplementary Figure 66. ^{13}C NMR (126 MHz, $CDCl_3$) of **7**.

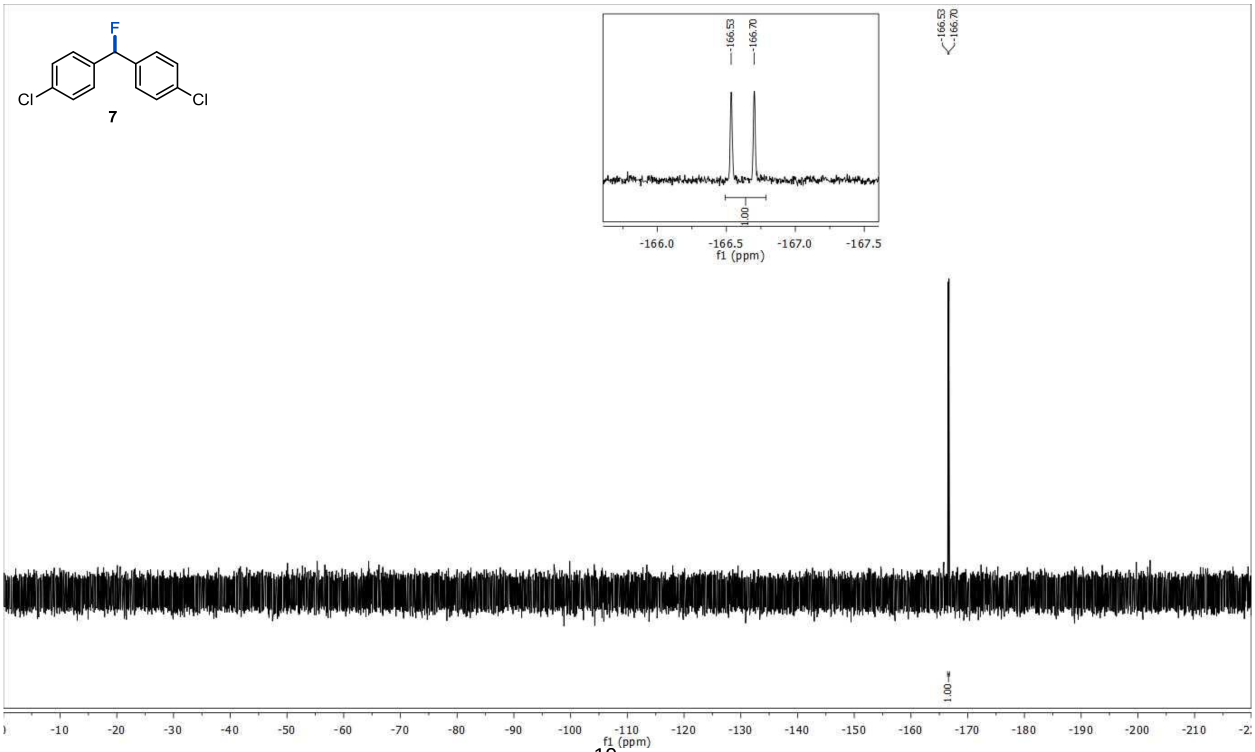

Supplementary Figure 67. ^{19}F NMR (282 MHz, $CDCl_3$) of **7**.

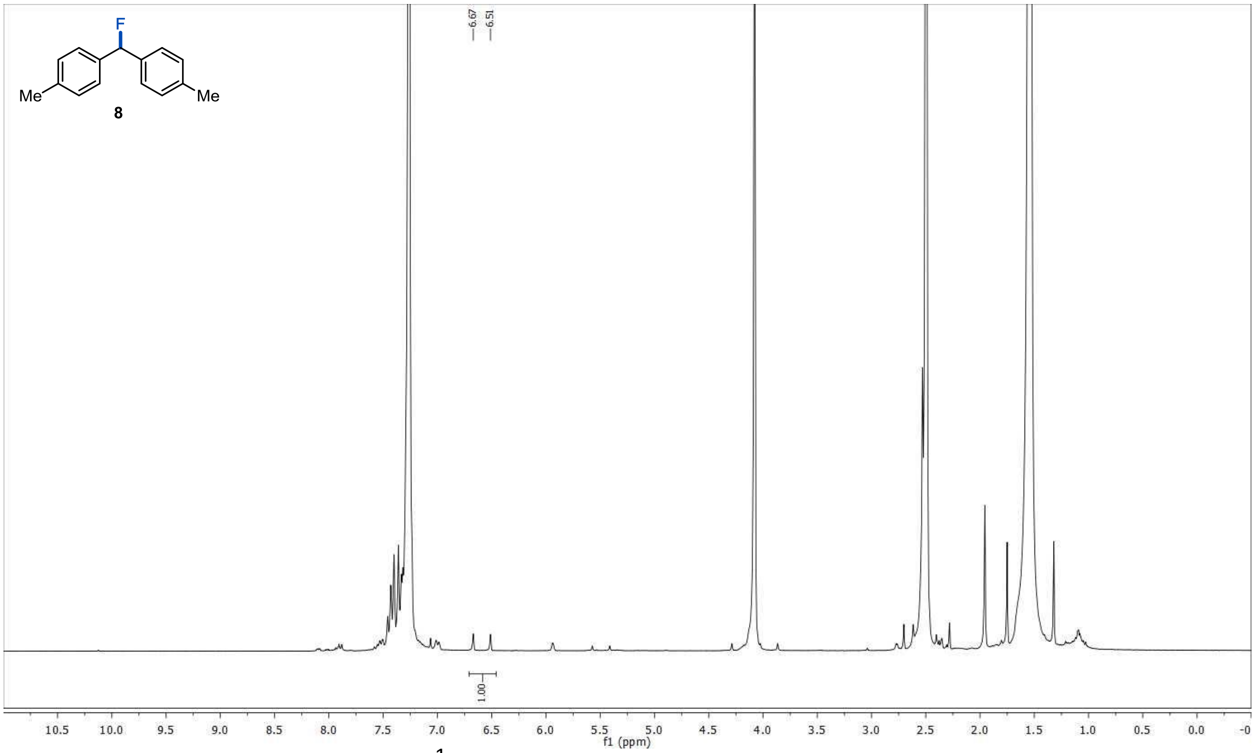

Supplementary Figure 68. ^{1}H NMR (500 MHz, $CDCl_3$) of **8** crude reaction mixture.

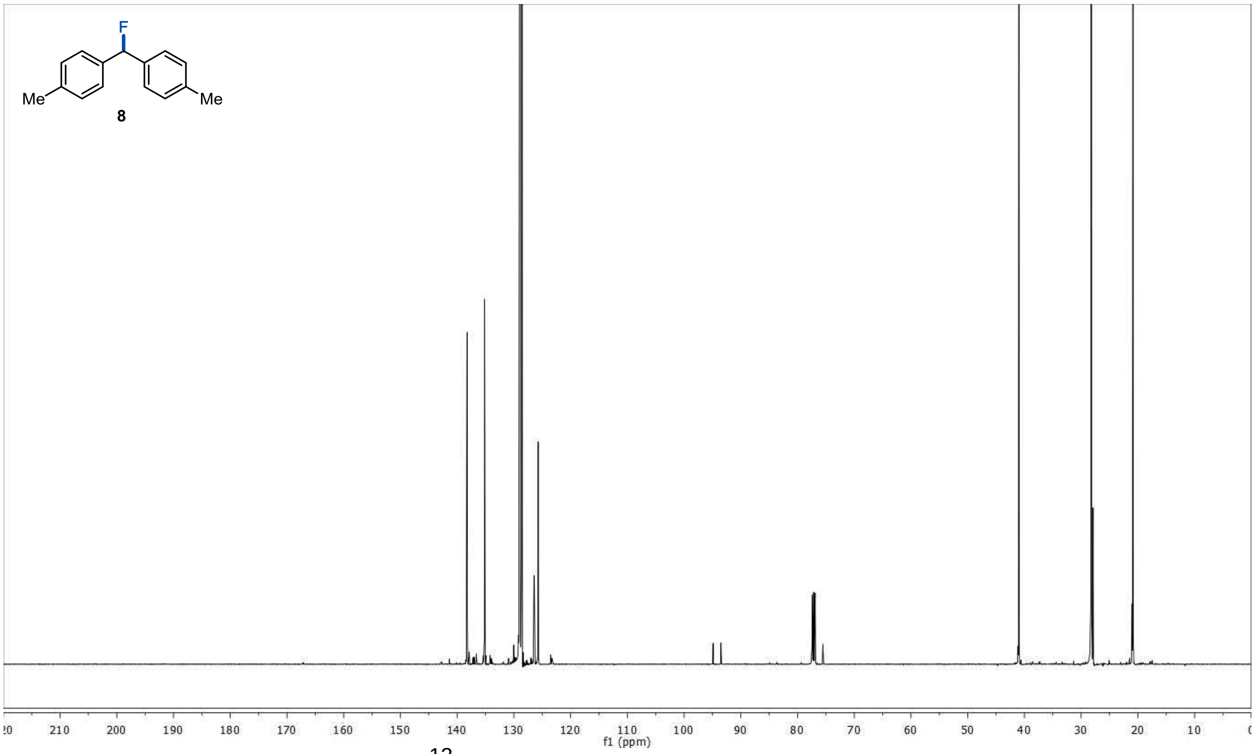

Supplementary Figure 69. ^{13}C NMR (126 MHz, $CDCl_3$) of **8** crude reaction mixture.

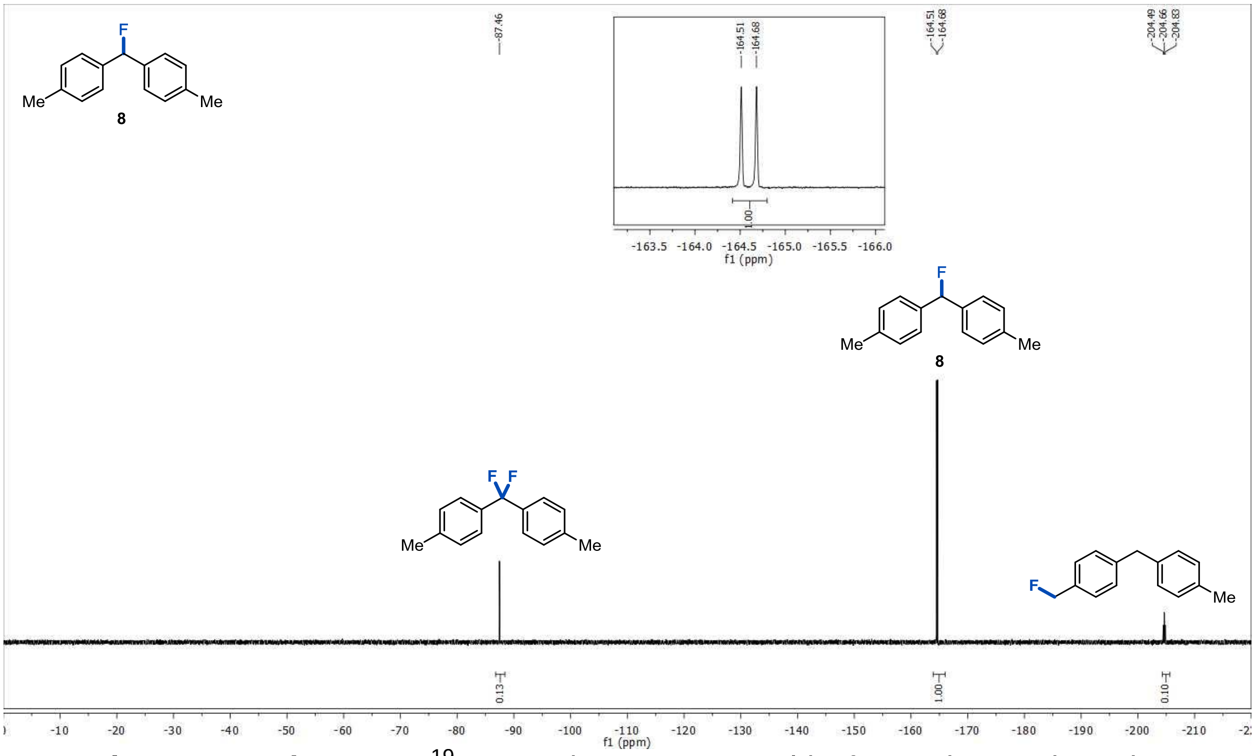

Supplementary Figure 70. ^{19}F NMR (282 MHz, $CDCl_3$) of **8** crude reaction mixture.

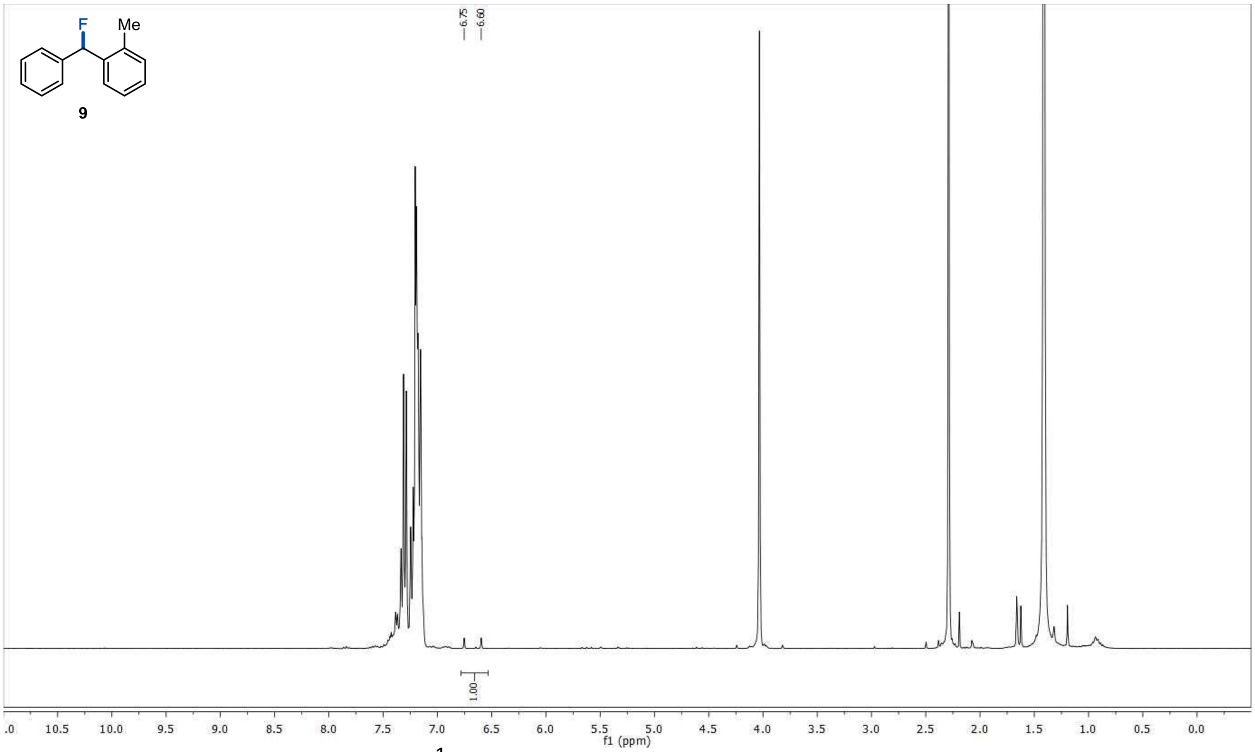

Supplementary Figure 71. ^{1}H NMR (500 MHz, $CDCl_3$) of **9** crude reaction mixture.

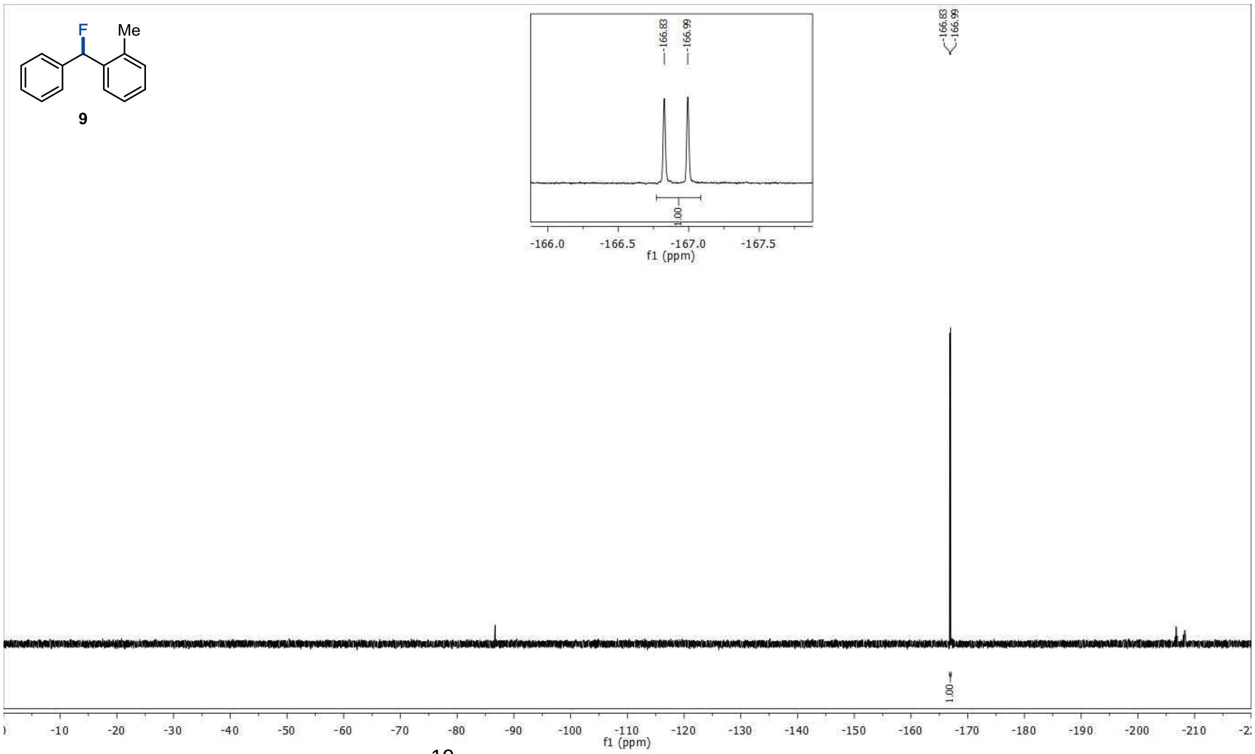

Supplementary Figure 72. ^{19}F NMR (282 MHz, $CDCl_3$) of **9** crude reaction mixture.

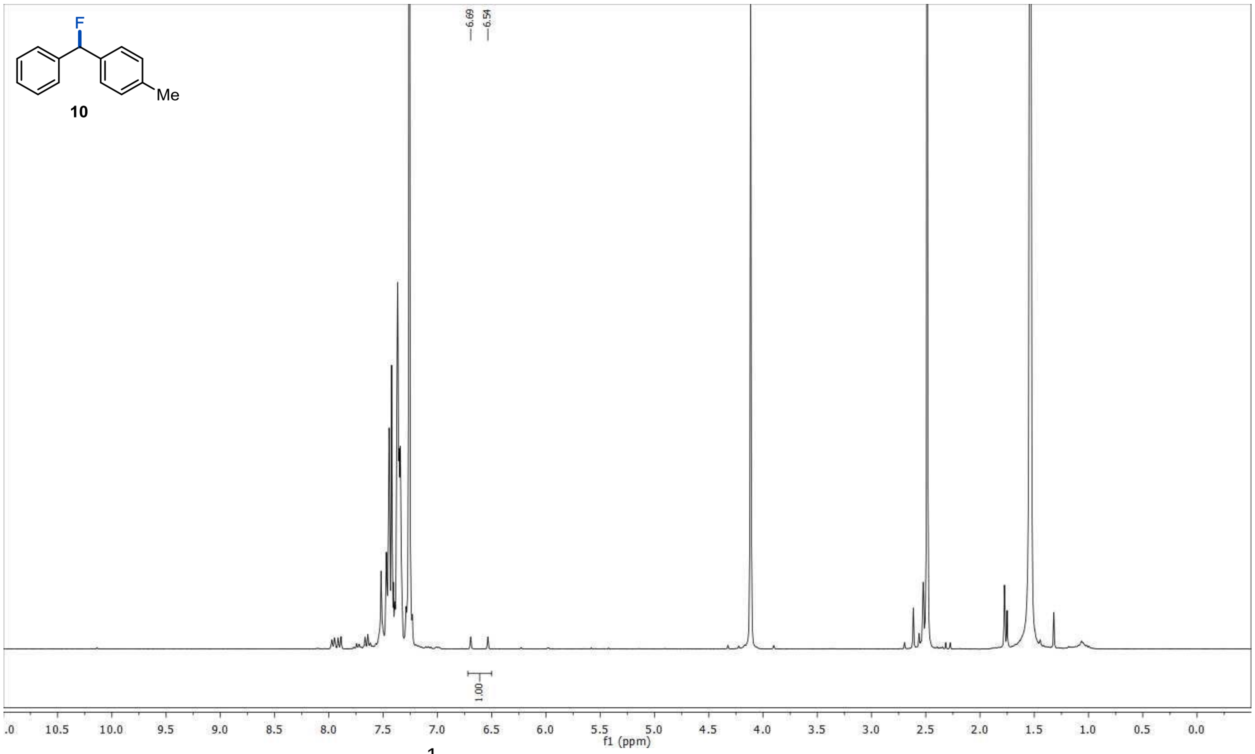

Supplementary Figure 73. ^{1}H NMR (500 MHz, $CDCl_3$) of **10** crude reaction mixture.

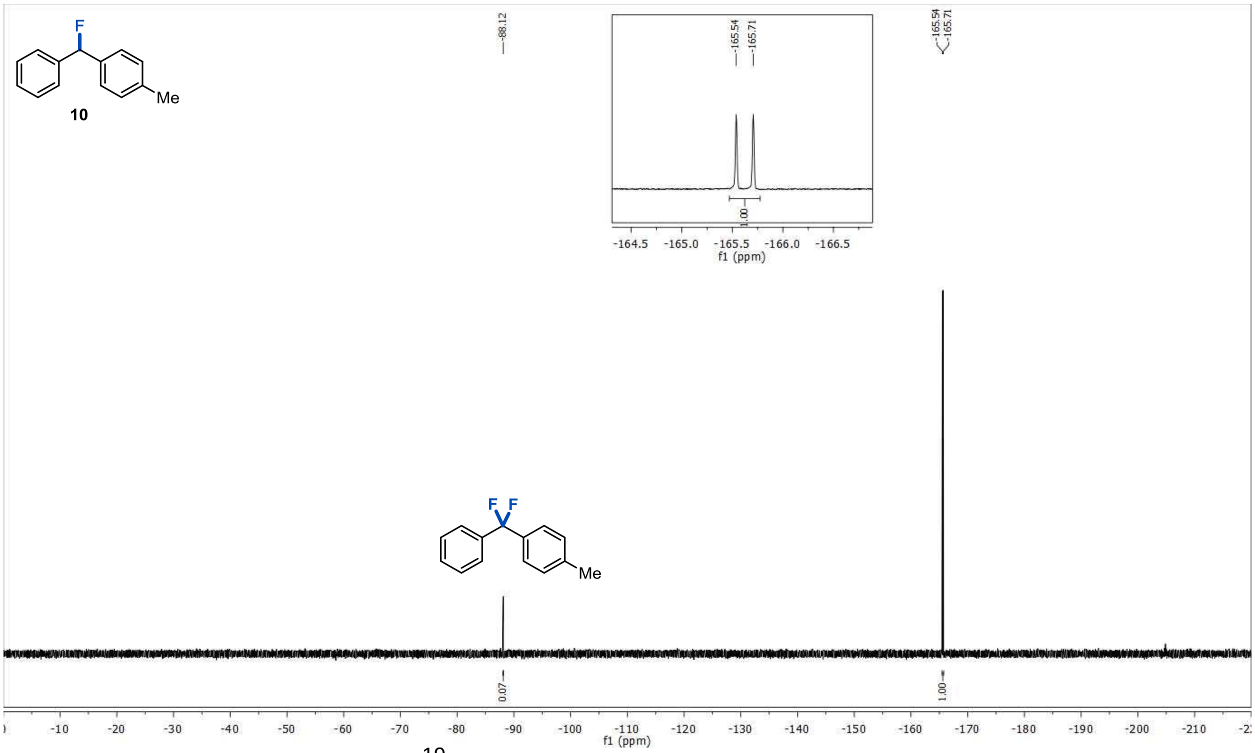

Supplementary Figure 74. ^{19}F NMR (282 MHz, $CDCl_3$) of **10** crude reaction mixture.

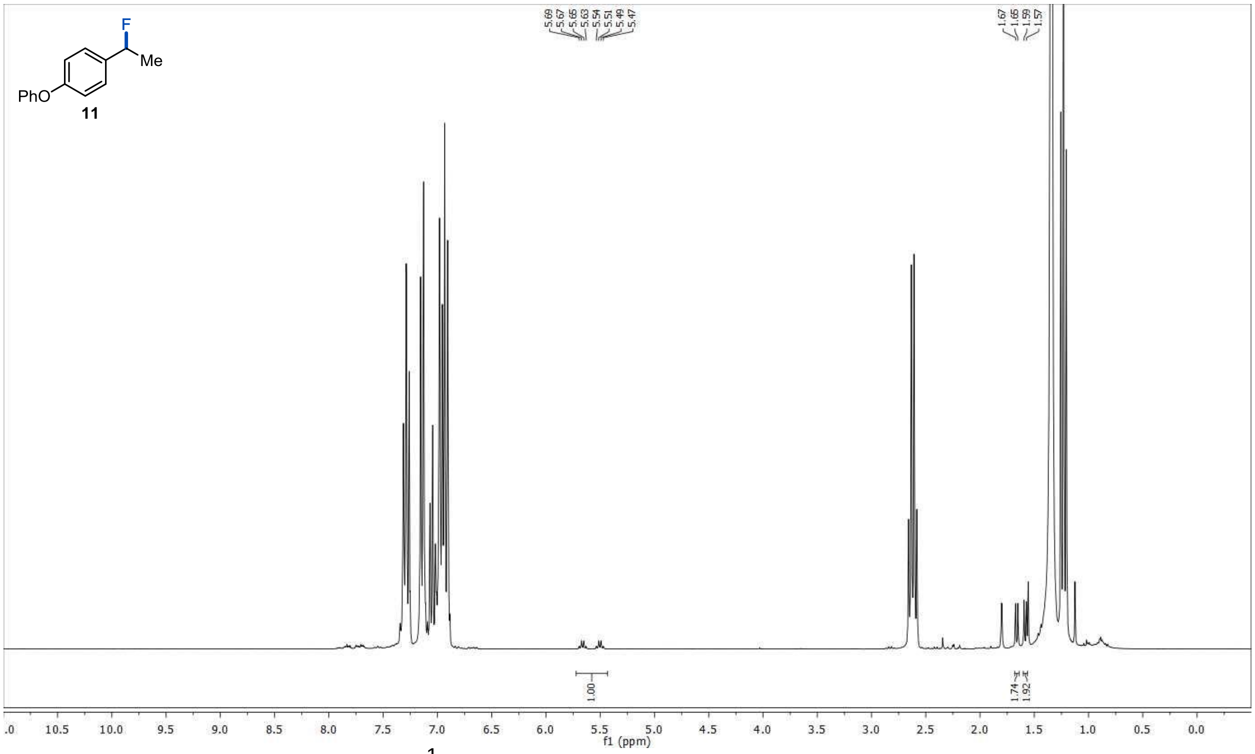

Supplementary Figure 75. ^{1}H NMR (500 MHz, $CDCl_3$) of **11** crude reaction mixture.

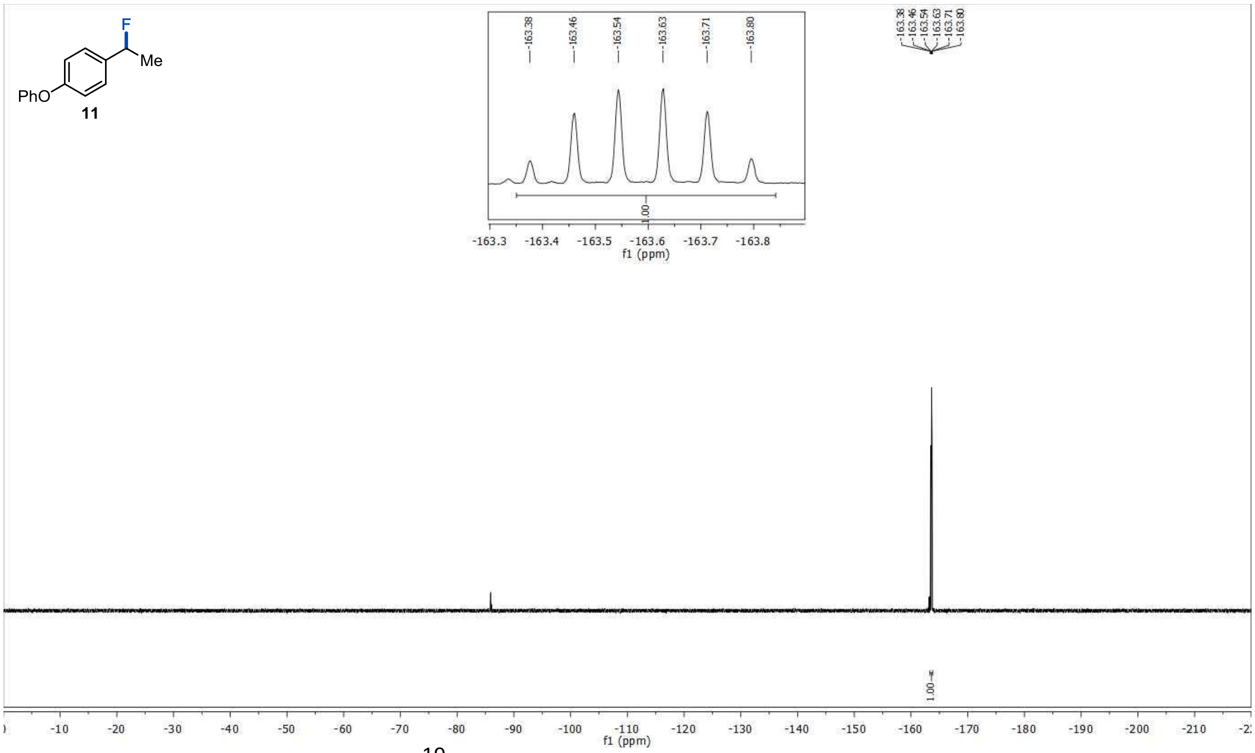

Supplementary Figure 76. ^{19}F NMR (282 MHz, $CDCl_3$) of **11** crude reaction mixture.

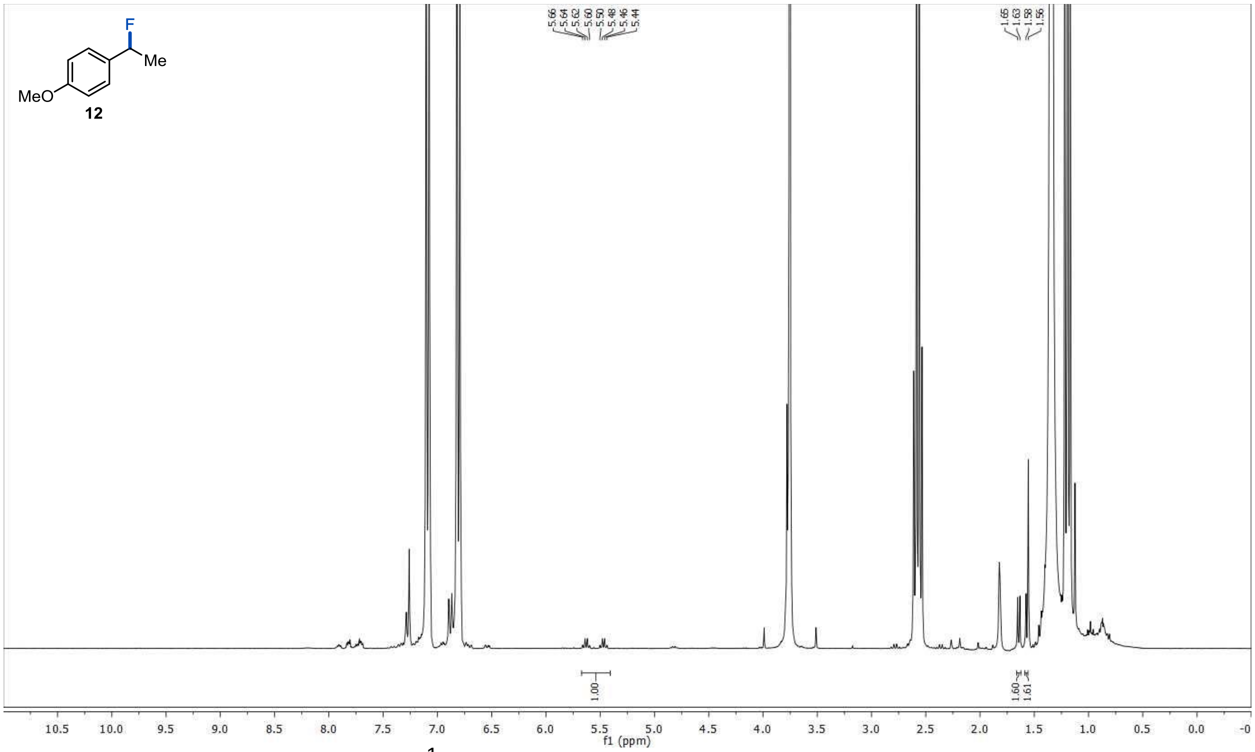

Supplementary Figure 77. ^{1}H NMR (500 MHz, $CDCl_3$) of **12** crude reaction mixture.

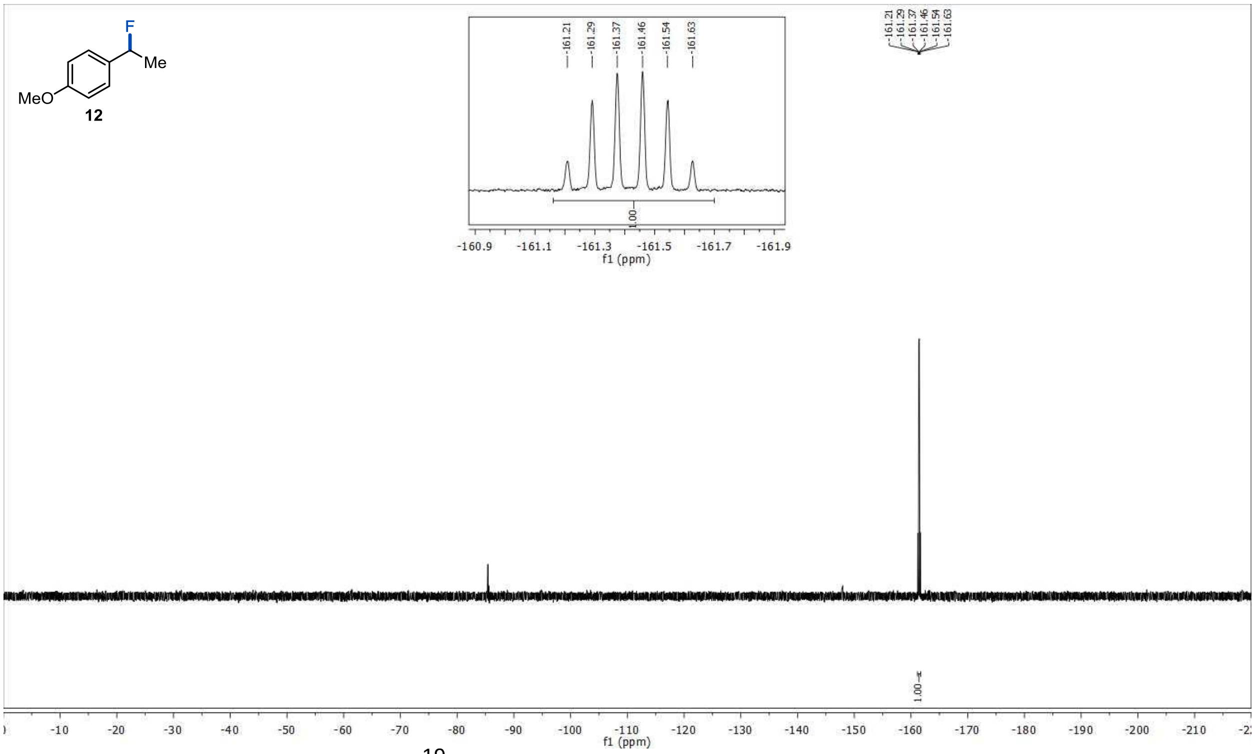

Supplementary Figure 78. ^{19}F NMR (282 MHz, $CDCl_3$) of **12** crude reaction mixture.

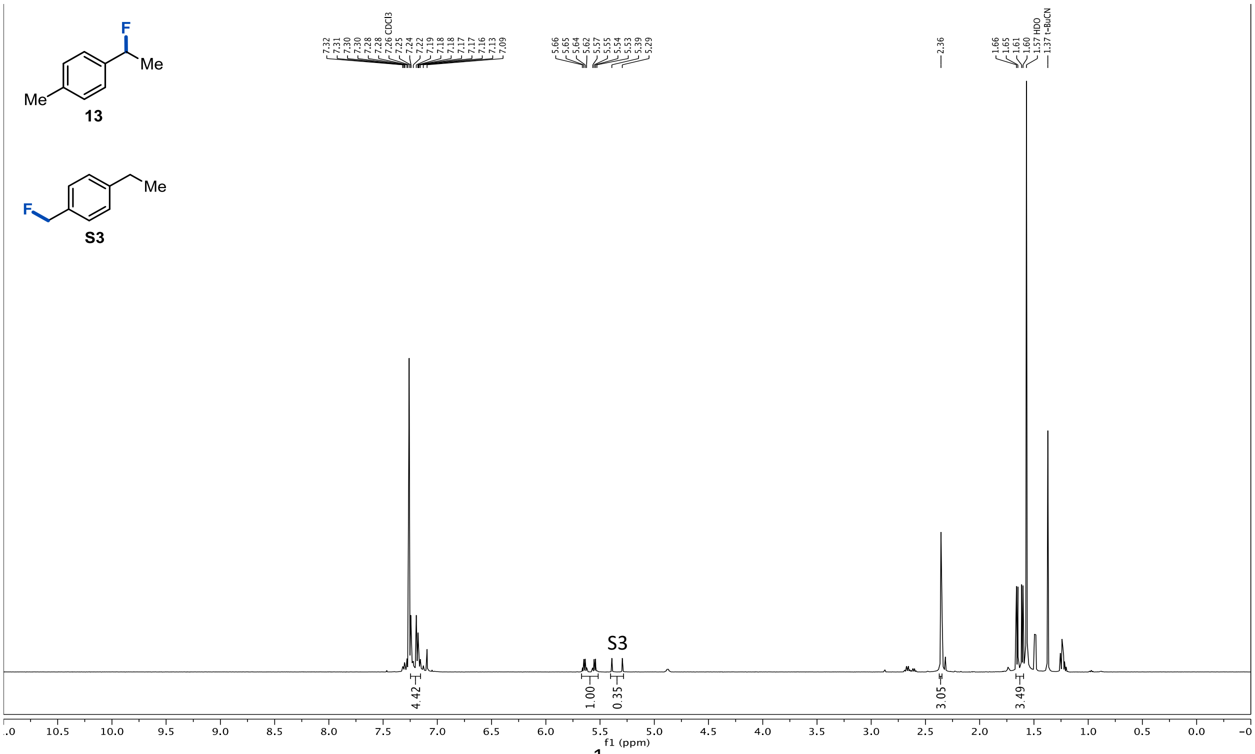

Supplementary Figure 79. ^{1}H NMR (500 MHz, $CDCl_3$) of **13**.

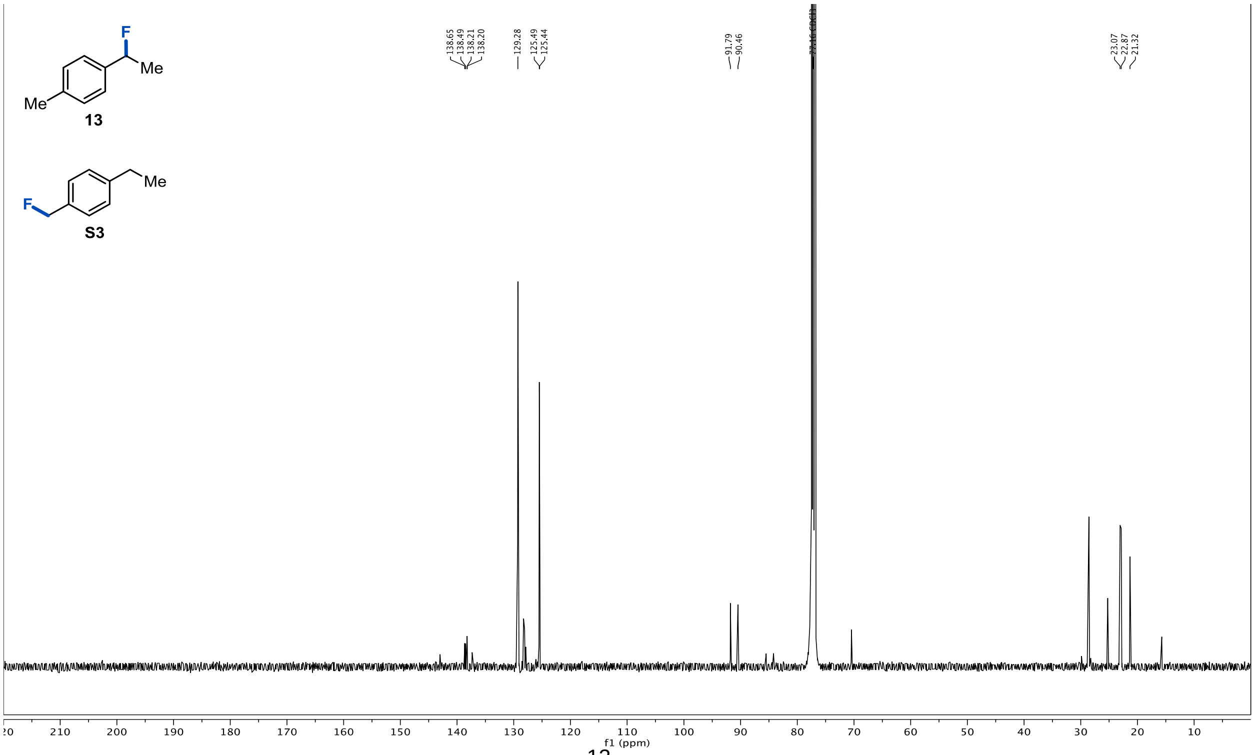

Supplementary Figure 80. ^{13}C NMR (126 MHz, $CDCl_3$) of **13**.

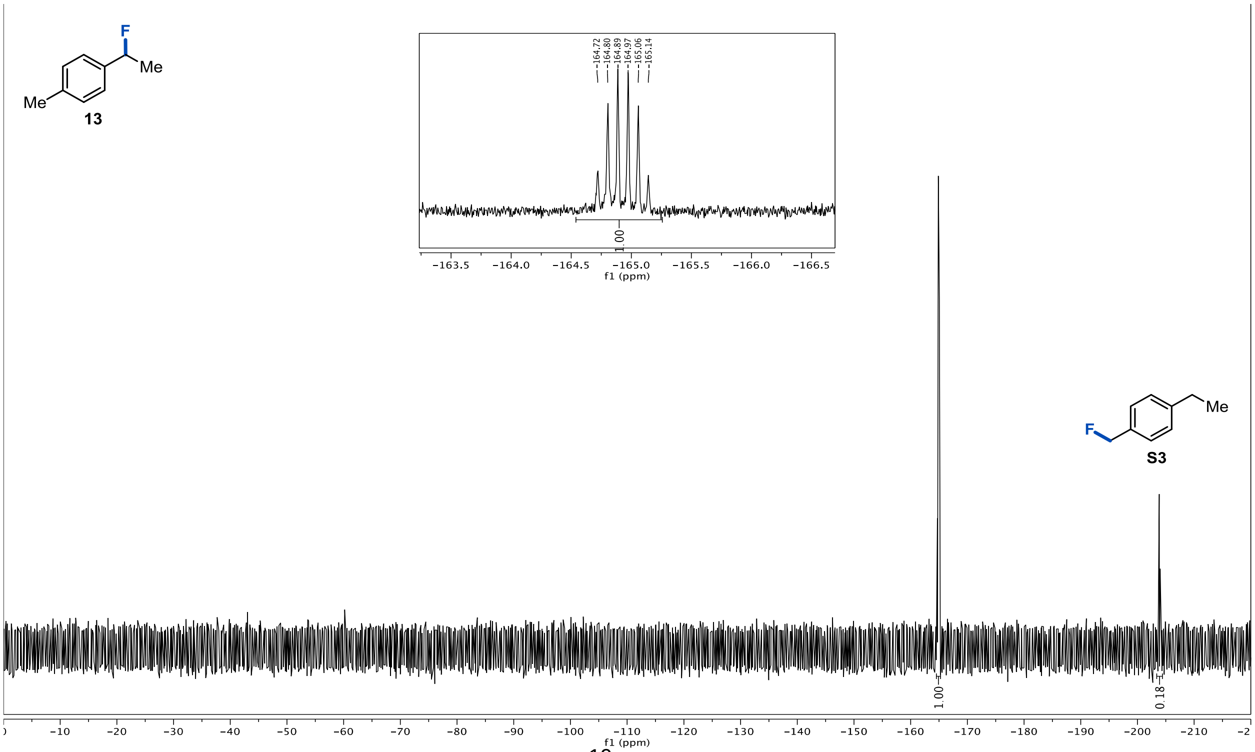

Supplementary Figure 81. ^{19}F NMR (282 MHz, $CDCl_3$) of **13**.

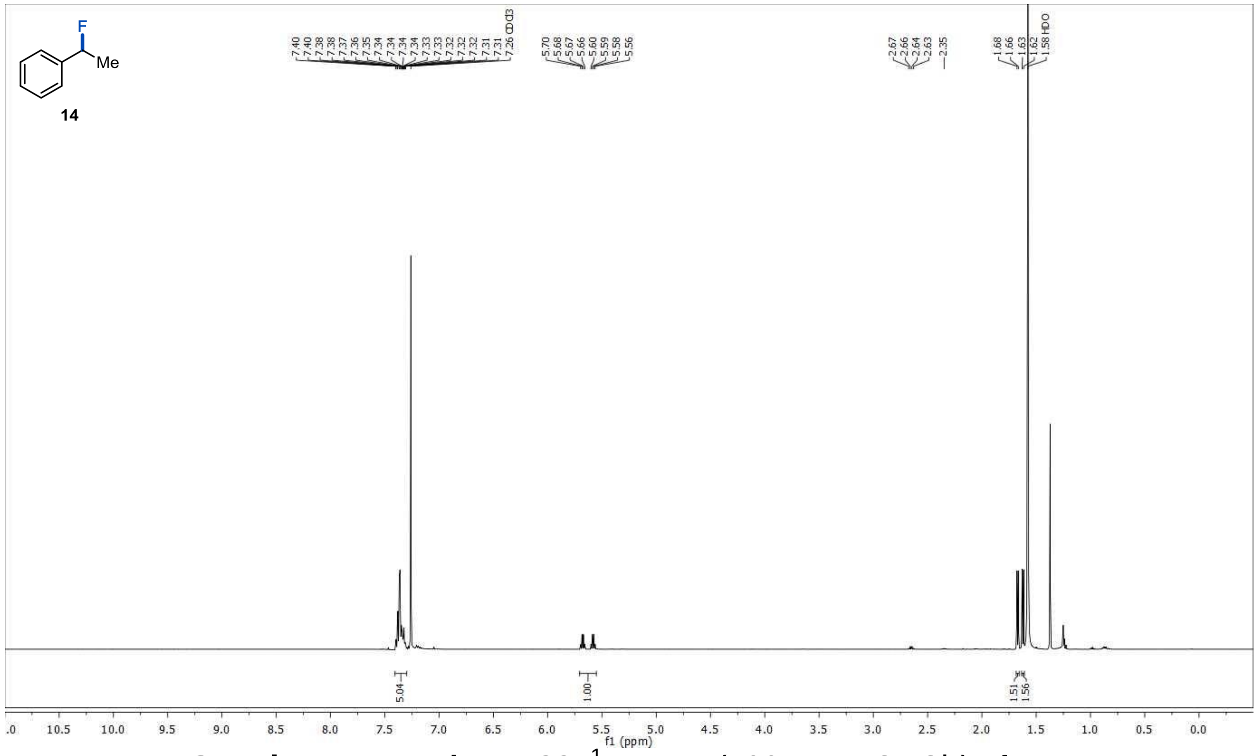

Supplementary Figure 82. ^{1}H NMR (500 MHz, $CDCl_3$) of **14**.

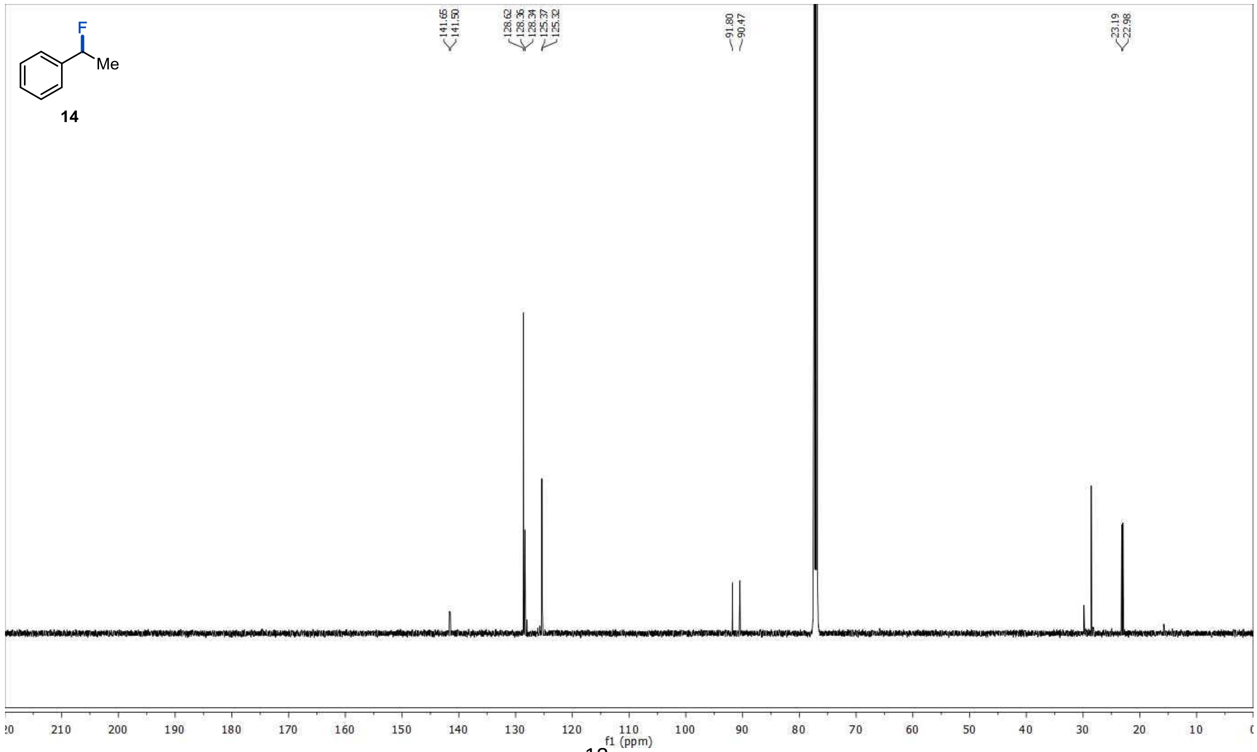

Supplementary Figure 83. ^{13}C NMR (126 MHz, $CDCl_3$) of **14**.

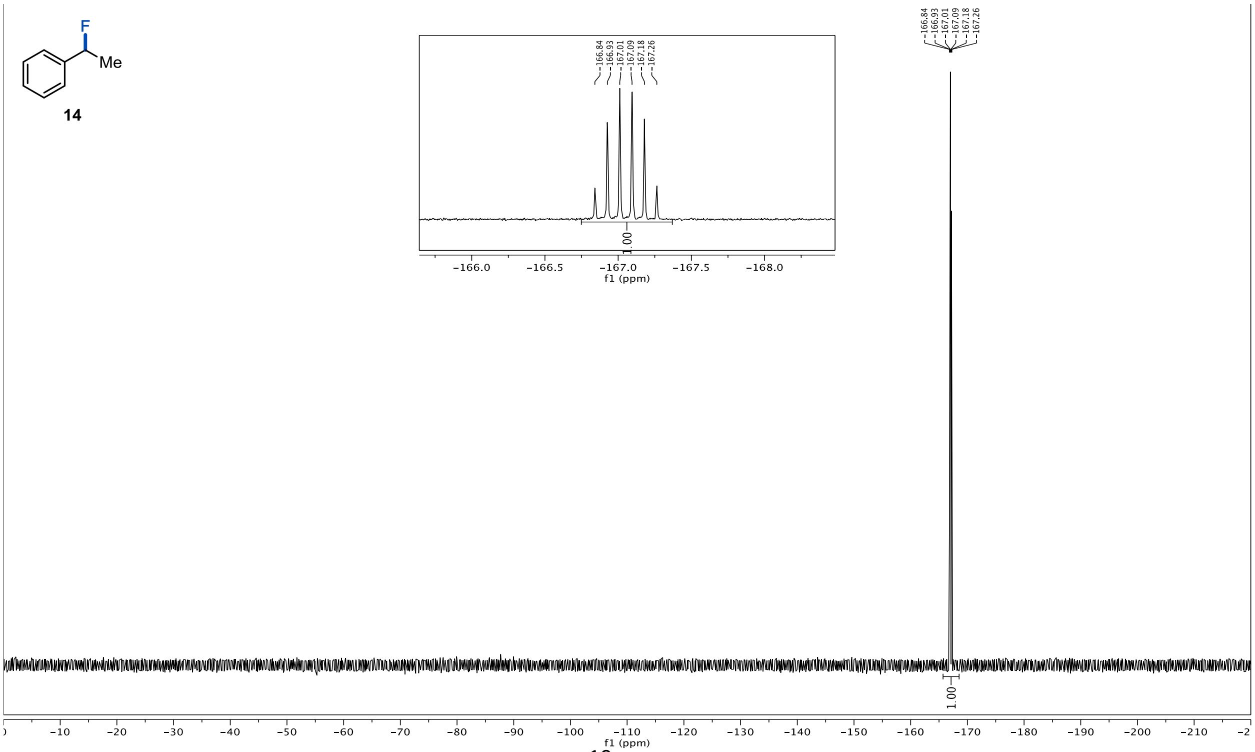

Supplementary Figure 84. ^{19}F NMR (282 MHz, $CDCl_3$) of **14**.

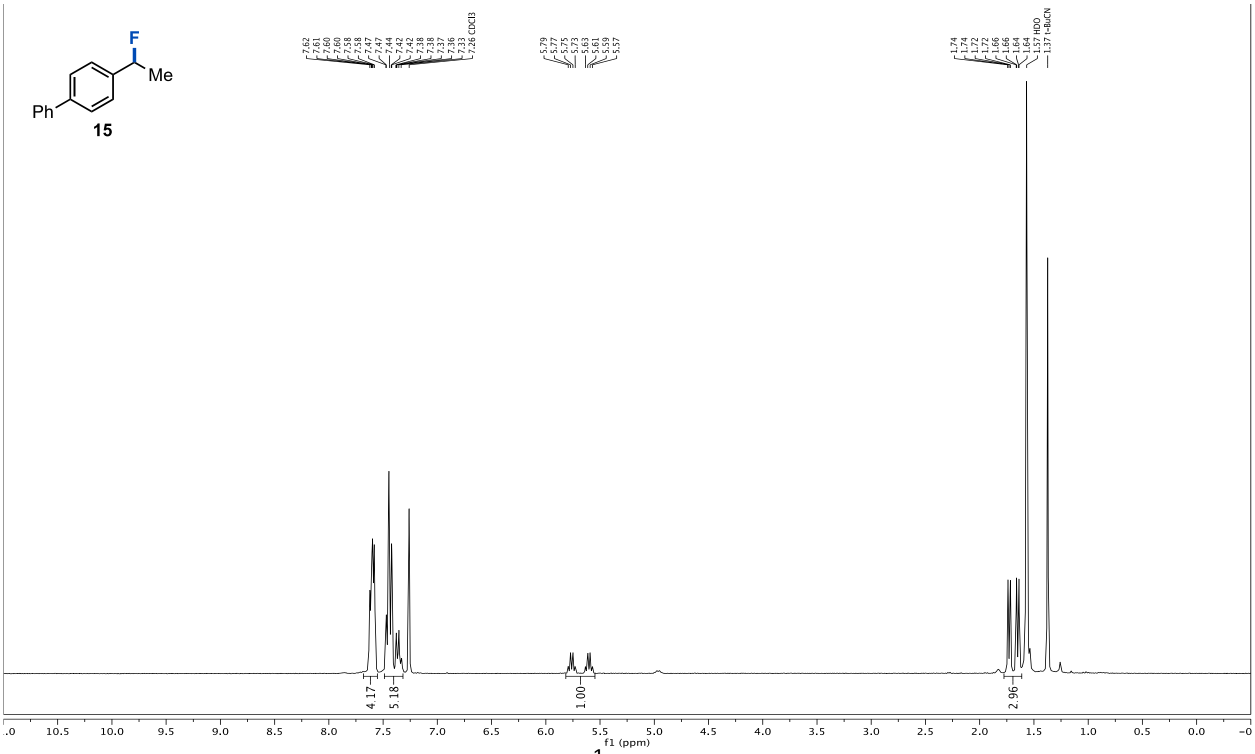

Supplementary Figure 85. ^{1}H NMR (500 MHz, $CDCl_3$) of **15**.

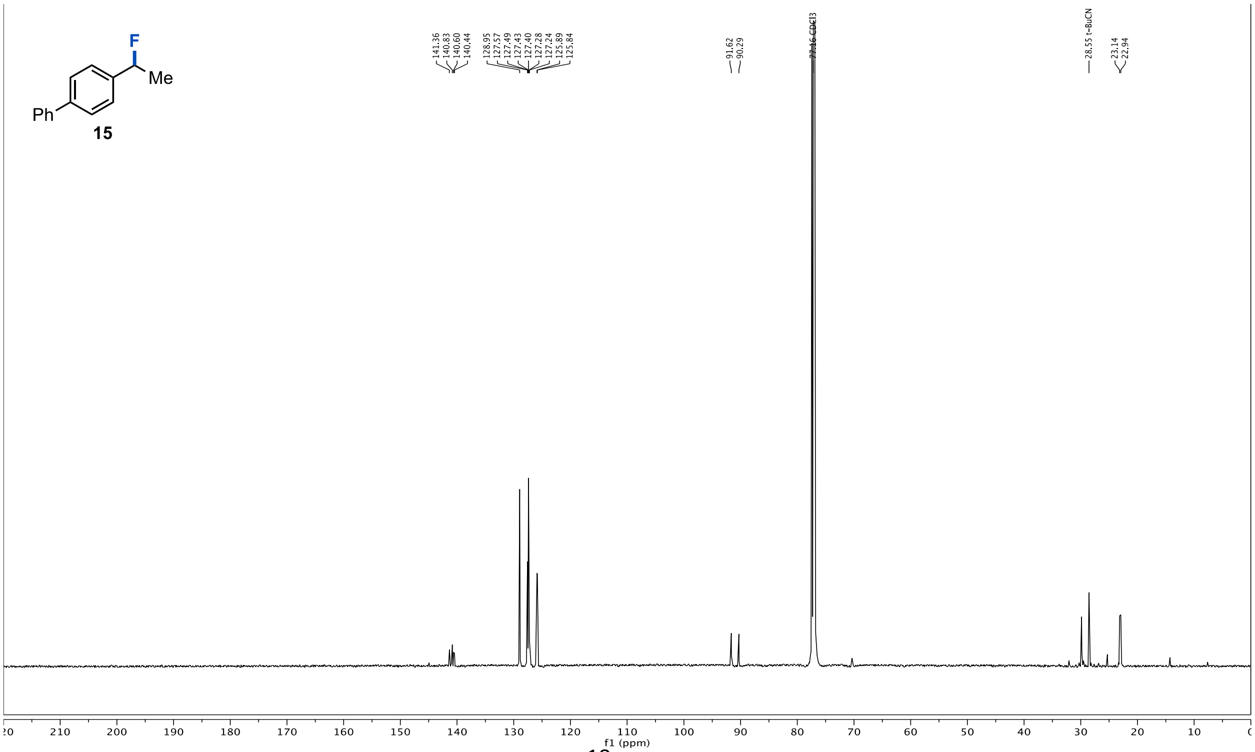

Supplementary Figure 86. ^{13}C NMR (126 MHz, $CDCl_3$) of **15**.

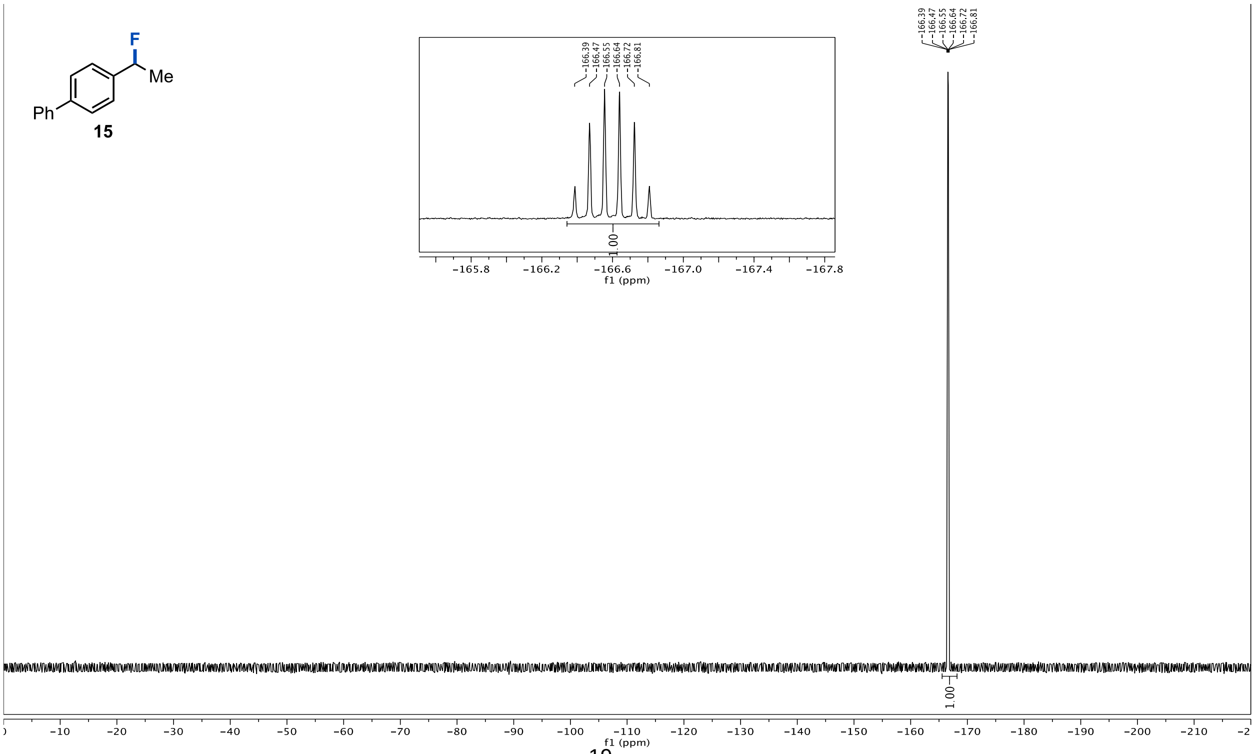

Supplementary Figure 87. ^{19}F NMR (282 MHz, $CDCl_3$) of **15**.

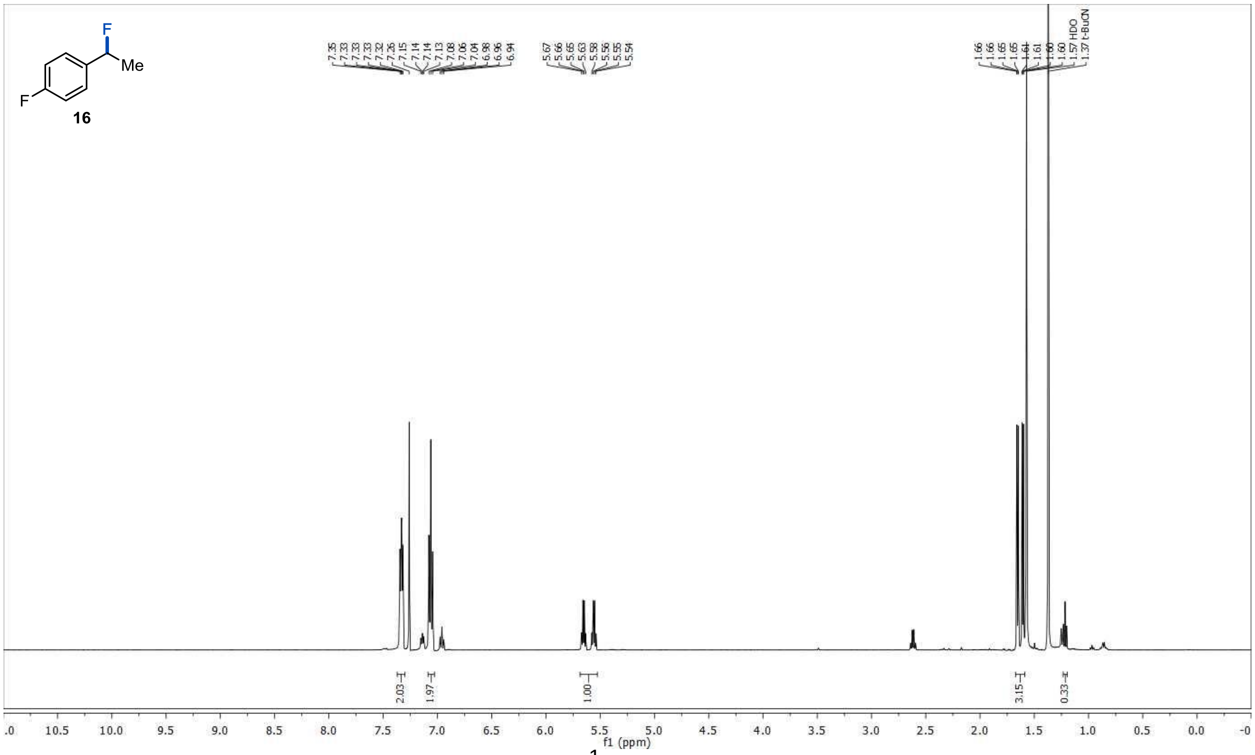

Supplementary Figure 88. ^{1}H NMR (500 MHz, $CDCl_3$) of **16**.

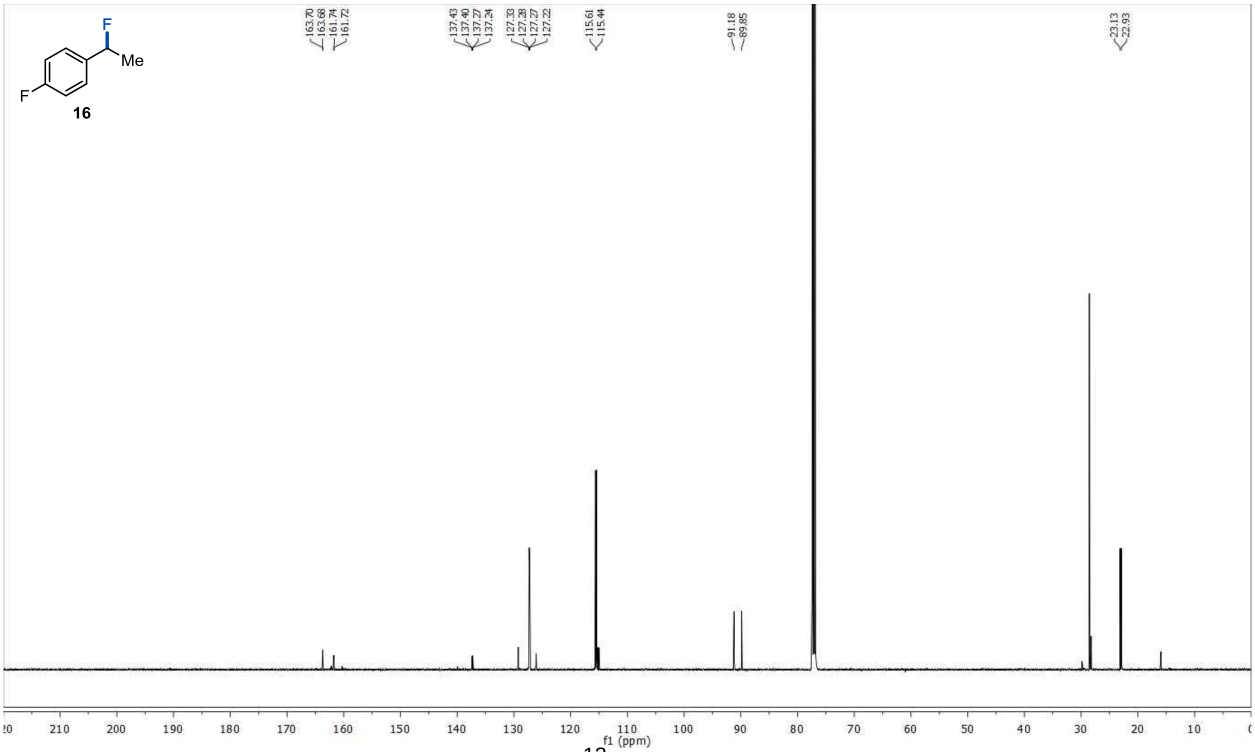

Supplementary Figure 89. ^{13}C NMR (126 MHz, $CDCl_3$) of **16**.

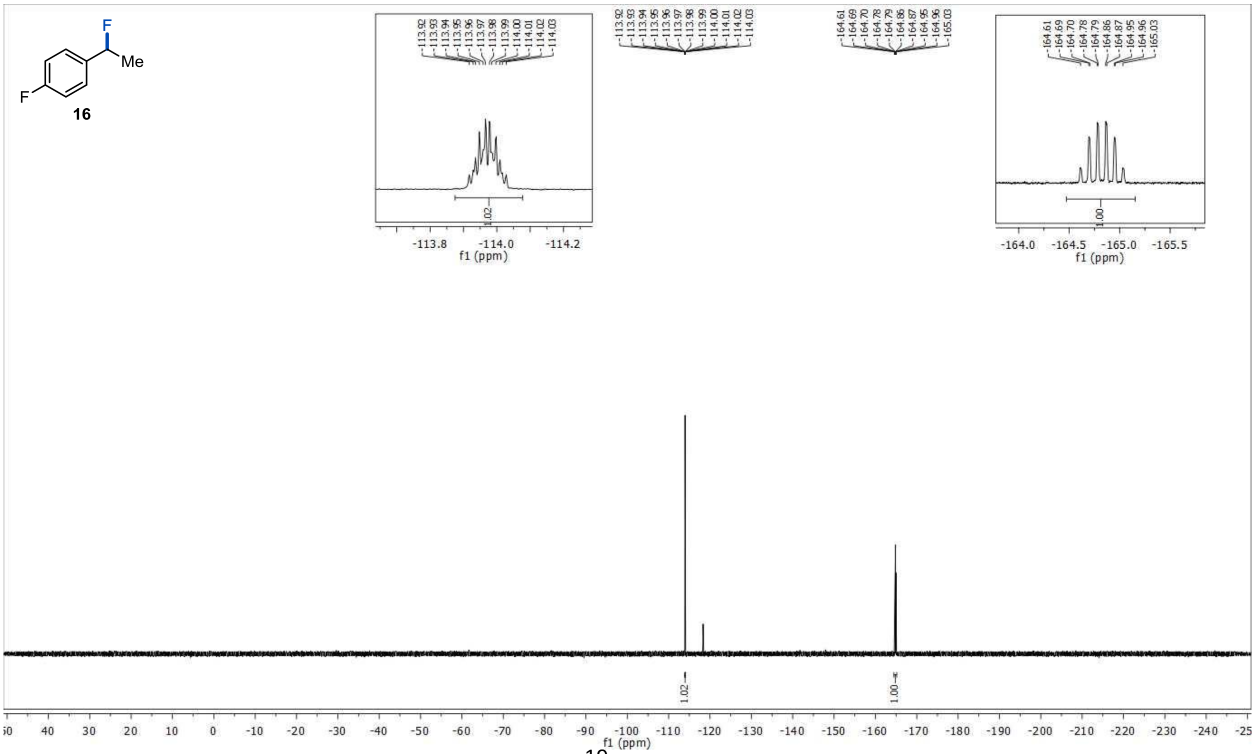

Supplementary Figure 90. ^{19}F NMR (282 MHz, $CDCl_3$) of **16**.

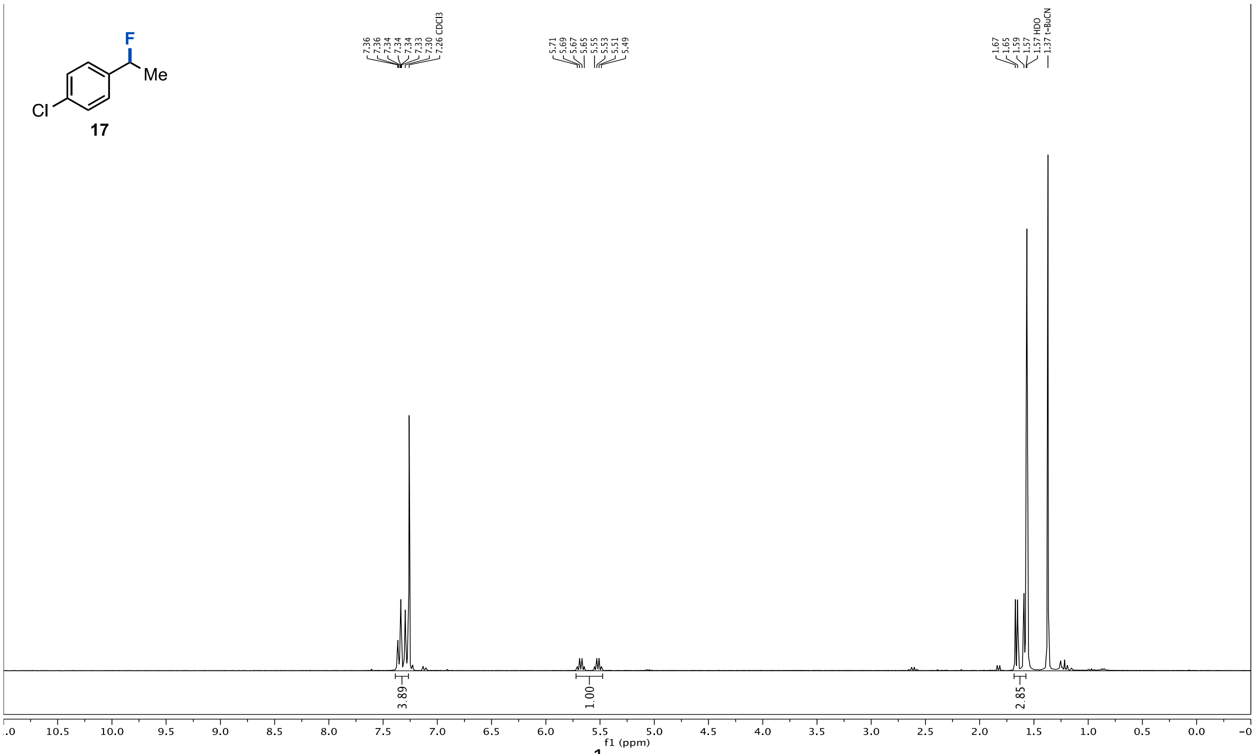

Supplementary Figure 91. ^{1}H NMR (500 MHz, $CDCl_3$) of **17**.

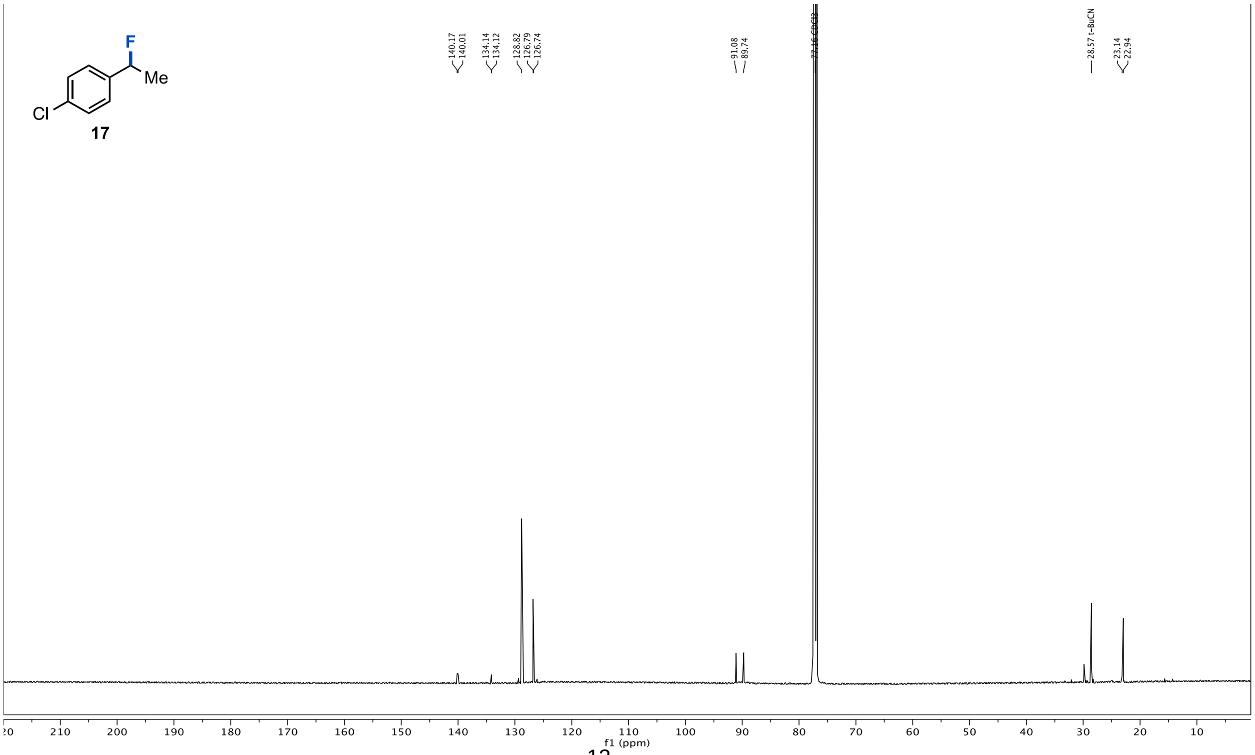

Supplementary Figure 92. ^{13}C NMR (126 MHz, $CDCl_3$) of **17**.

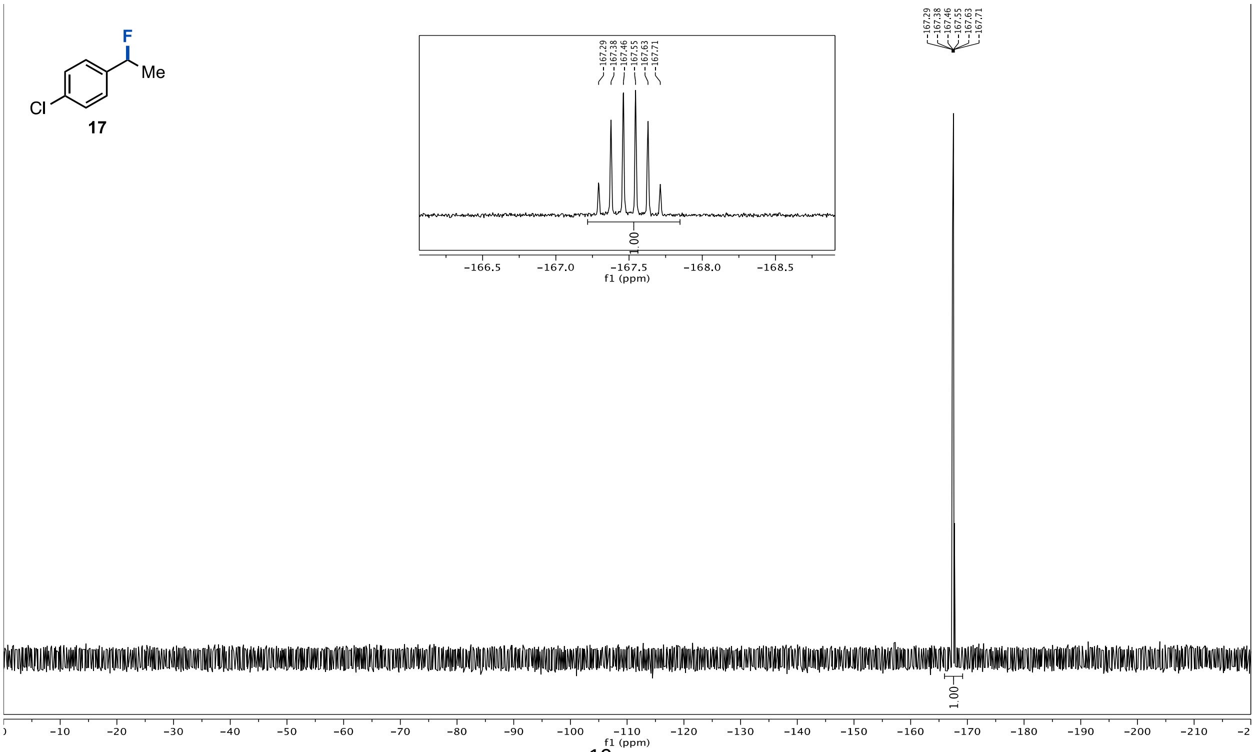

Supplementary Figure 93. ^{19}F NMR (282 MHz, $CDCl_3$) of **17**.

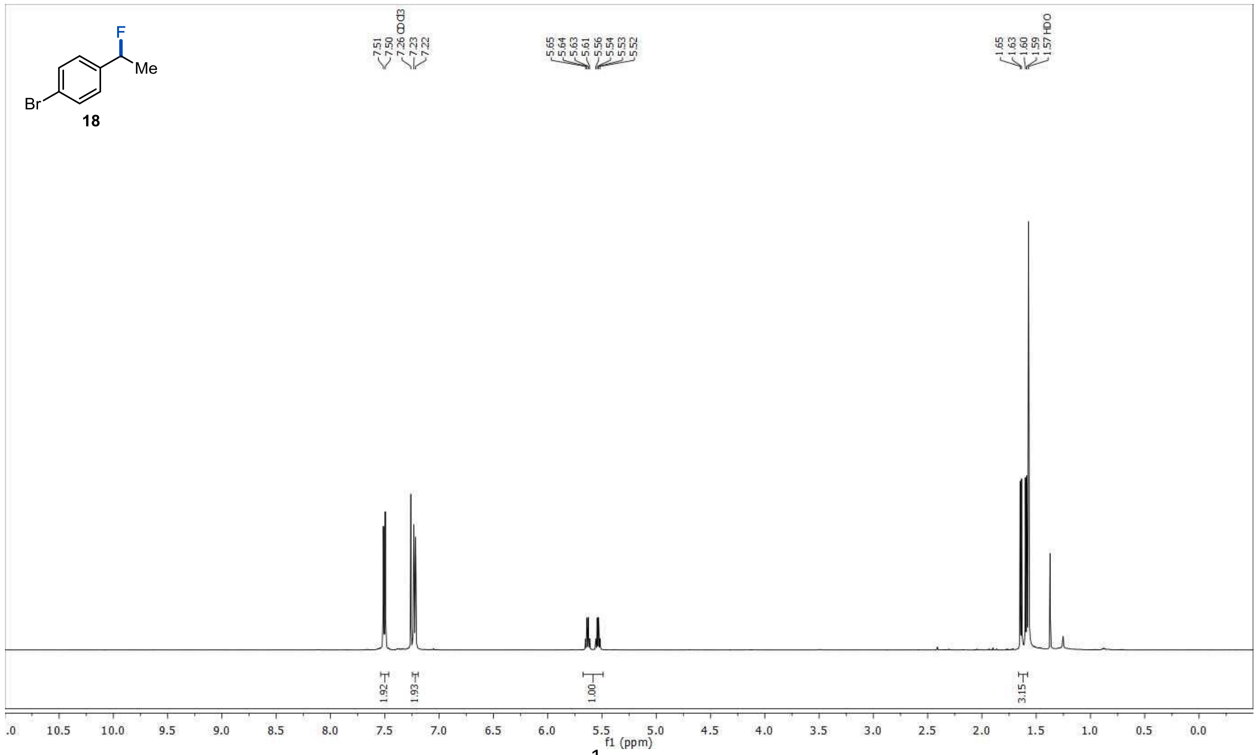

Supplementary Figure 94. ^{1}H NMR (500 MHz, $CDCl_3$) of **18**.

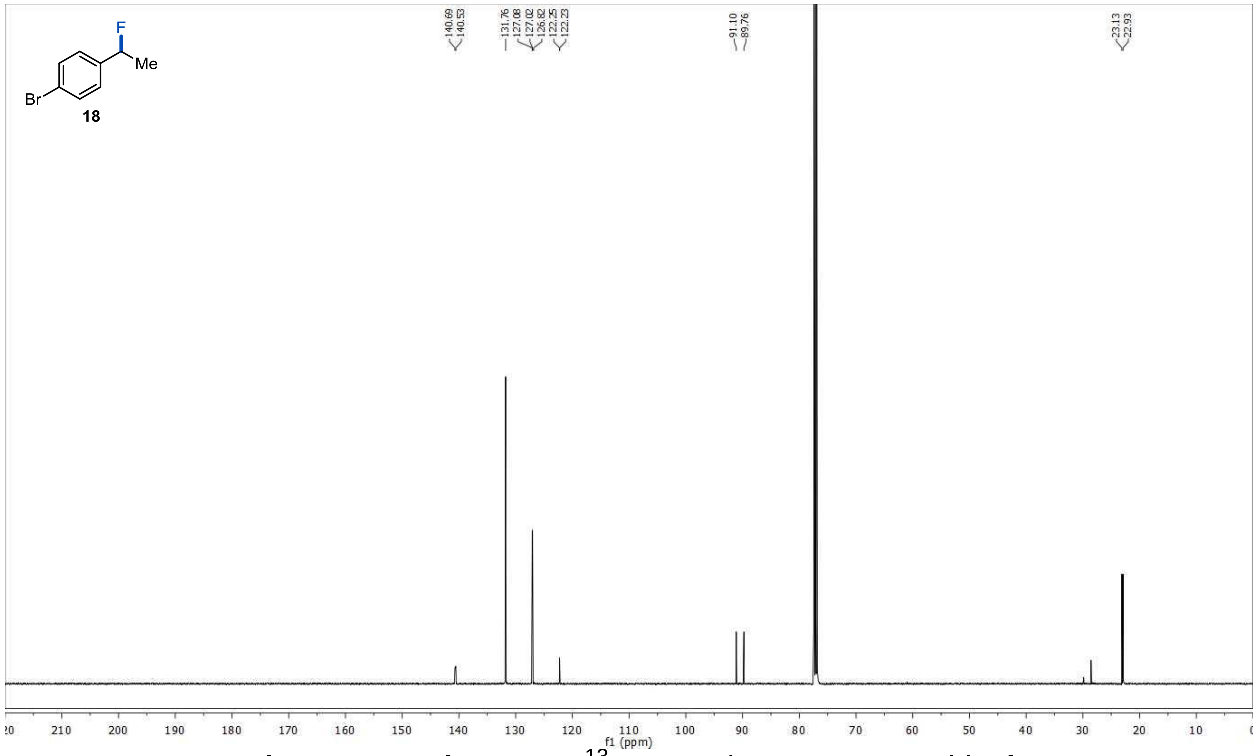

Supplementary Figure 95. ^{13}C NMR (126 MHz, $CDCl_3$) of **18**.

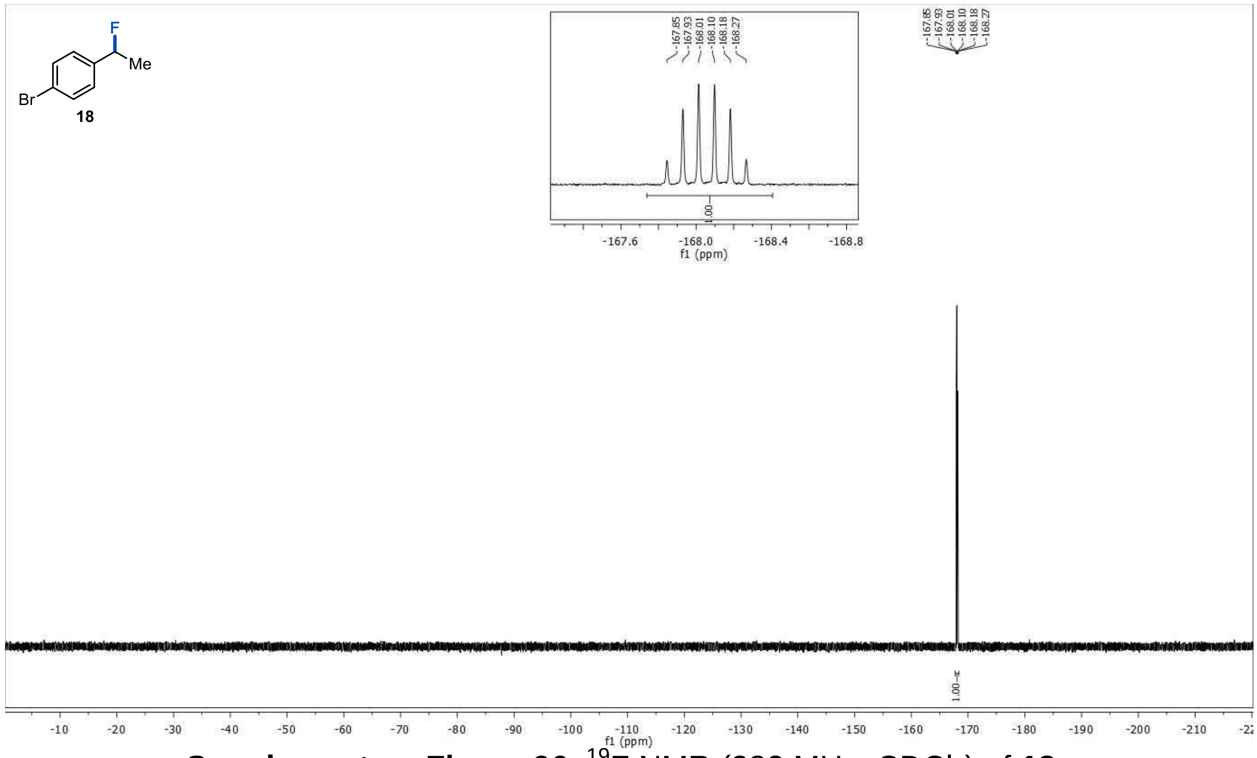

Supplementary Figure 96. ^{19}F NMR (282 MHz, $CDCl_3$) of **18**.

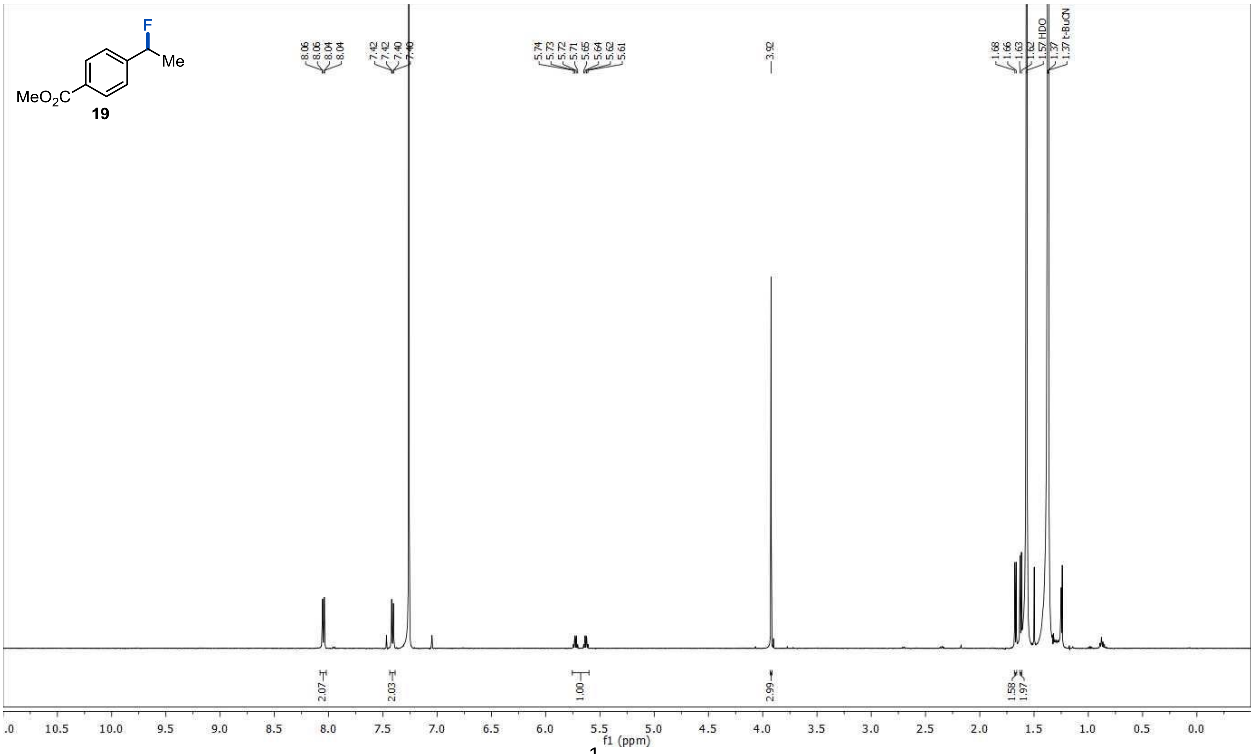

Supplementary Figure 97. ^{1}H NMR (500 MHz, $CDCl_3$) of **19**.

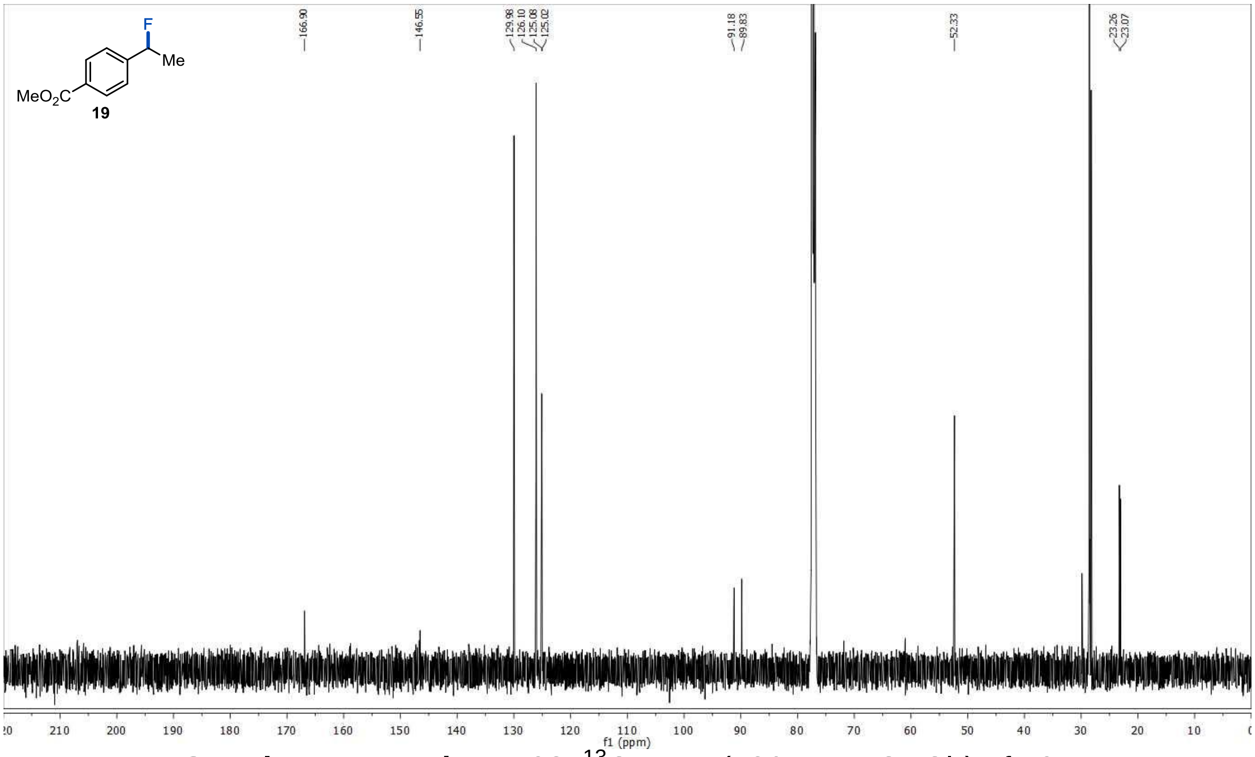

Supplementary Figure 98. ^{13}C NMR (126 MHz, $CDCl_3$) of **19**.

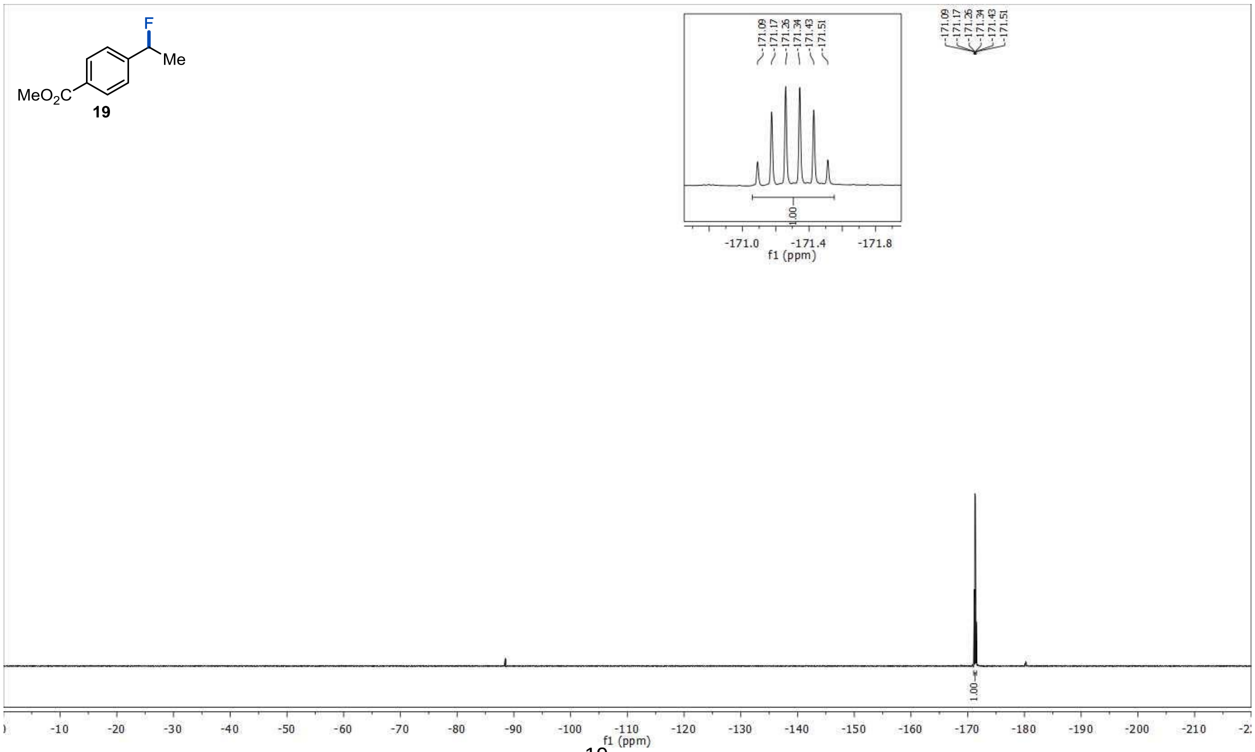

Supplementary Figure 99. ^{19}F NMR (282 MHz, $CDCl_3$) of **19**.

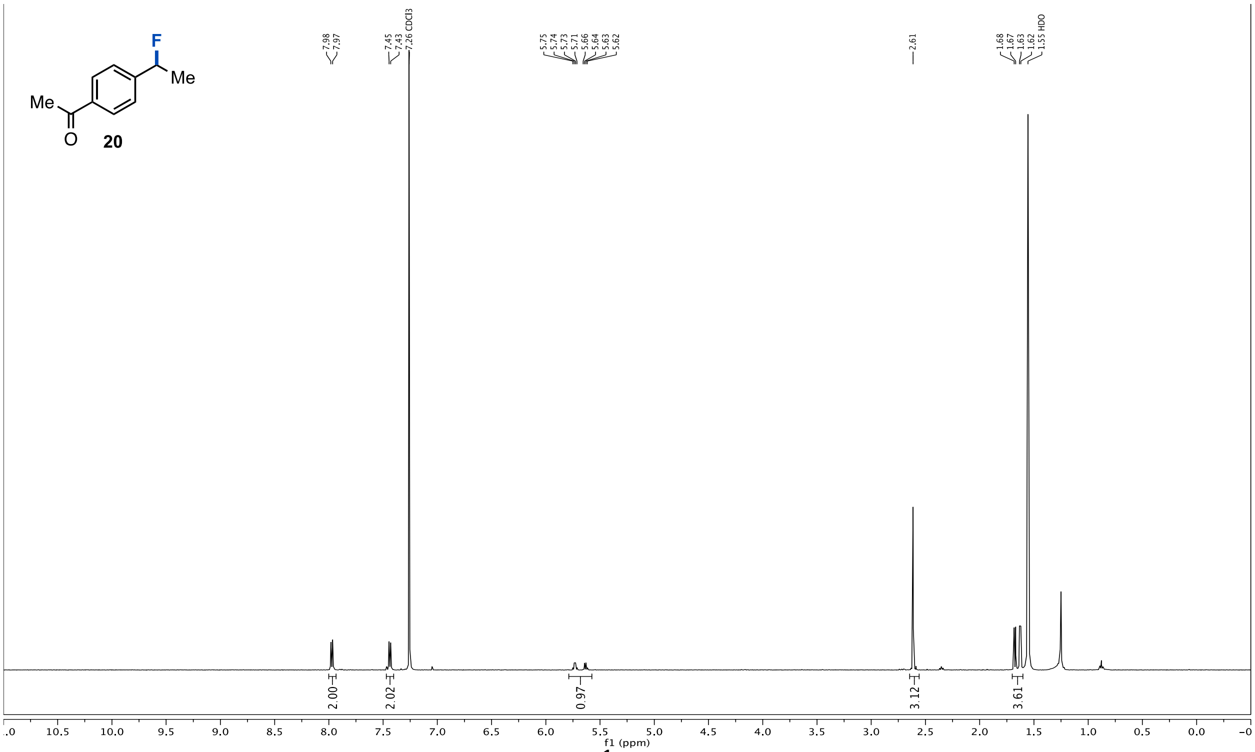

Supplementary Figure 100. ^{1}H NMR (500 MHz, $CDCl_3$) of **20**.

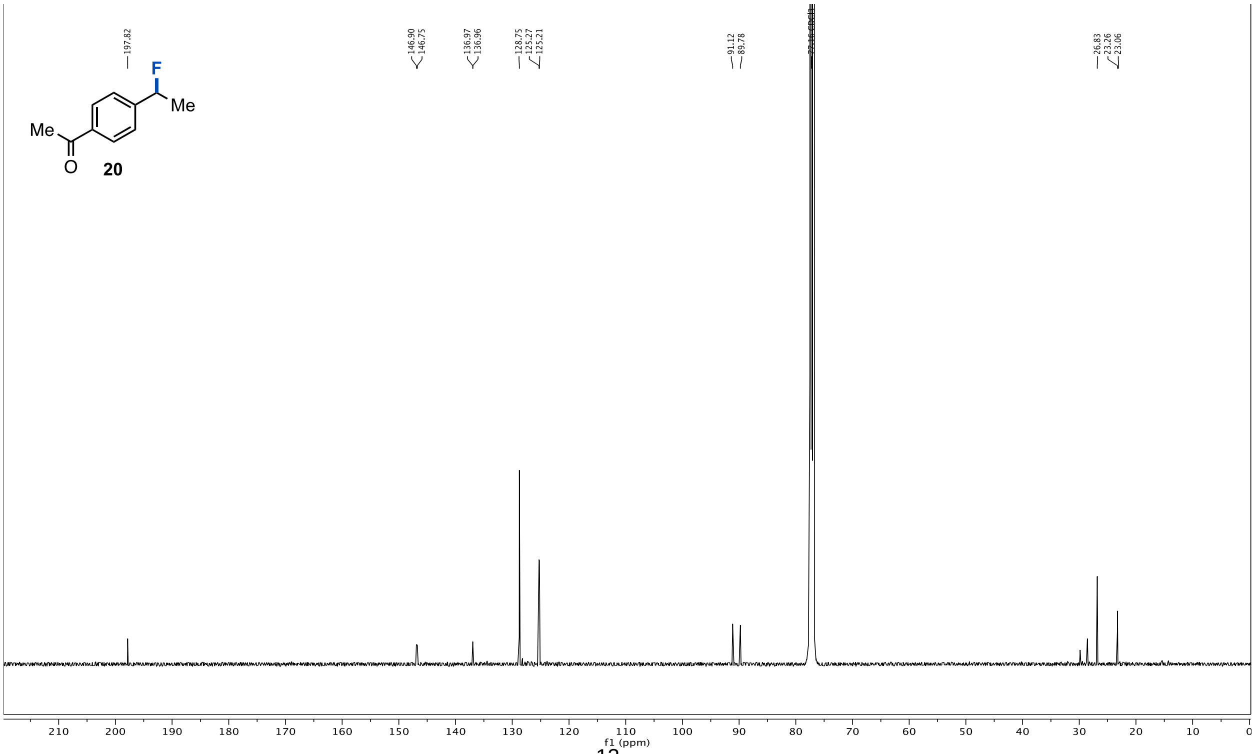

Supplementary Figure 101. ^{13}C NMR (126 MHz, $CDCl_3$) of **20**.

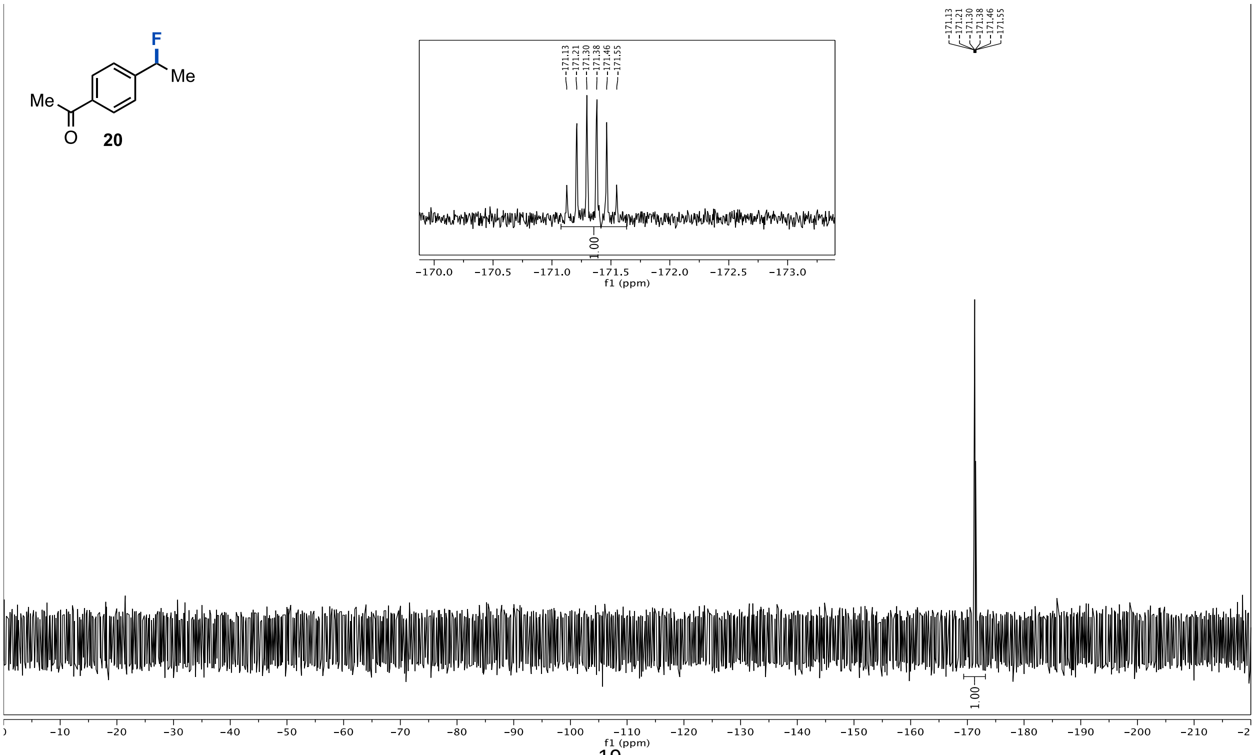

Supplementary Figure 102. ^{19}F NMR (282 MHz, $CDCl_3$) of **20**.

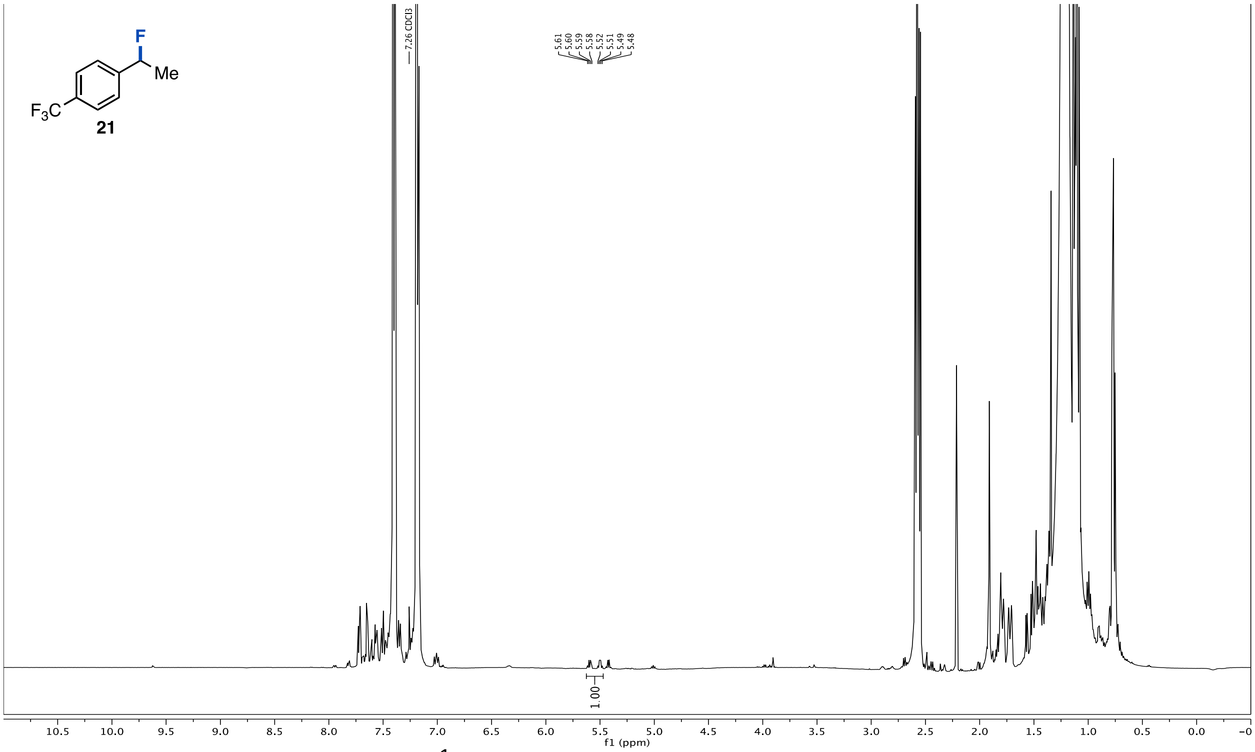

Supplementary Figure 103. ^{1}H NMR (500 MHz, $CDCl_3$) of **21** crude reaction mixture.

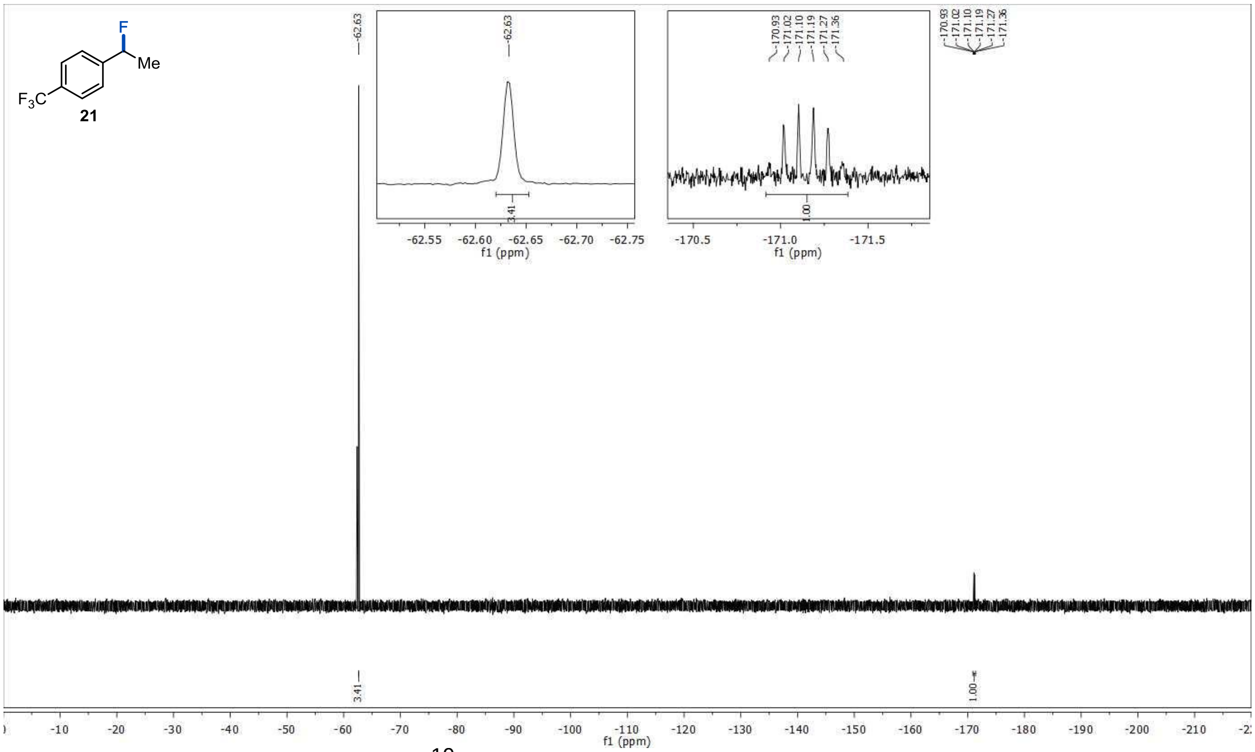

Supplementary Figure 104. ^{19}F NMR (282 MHz, $CDCl_3$) of **21** crude reaction mixture.

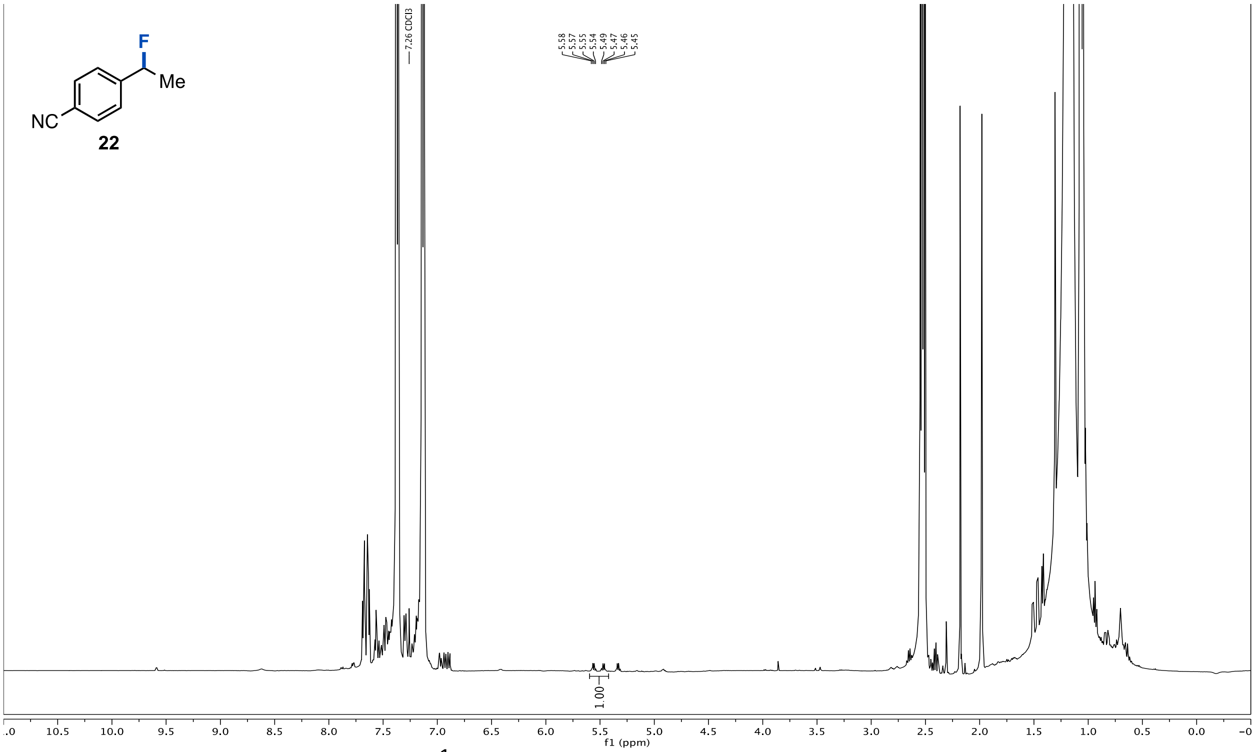

Supplementary Figure 105. ^{1}H NMR (500 MHz, $CDCl_3$) of **22** crude reaction mixture.

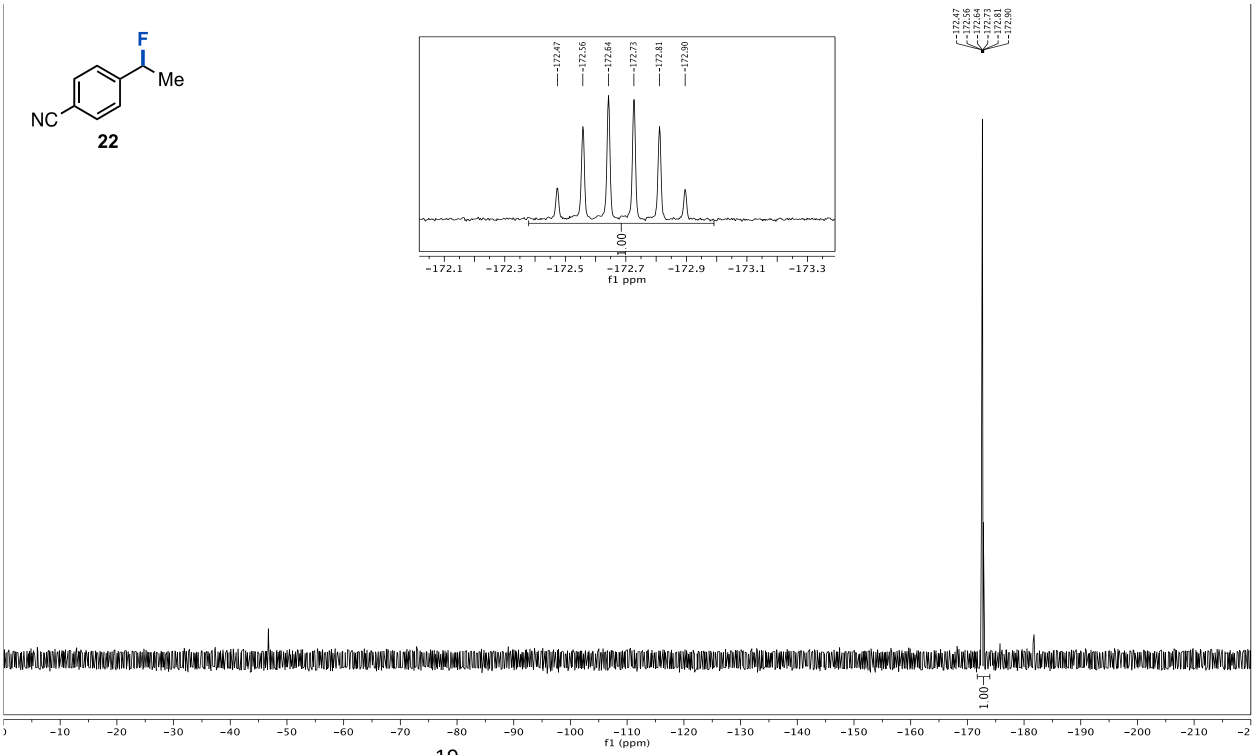

Supplementary Figure 106. ^{19}F NMR (282 MHz, $CDCl_3$) of **22** crude reaction mixture.

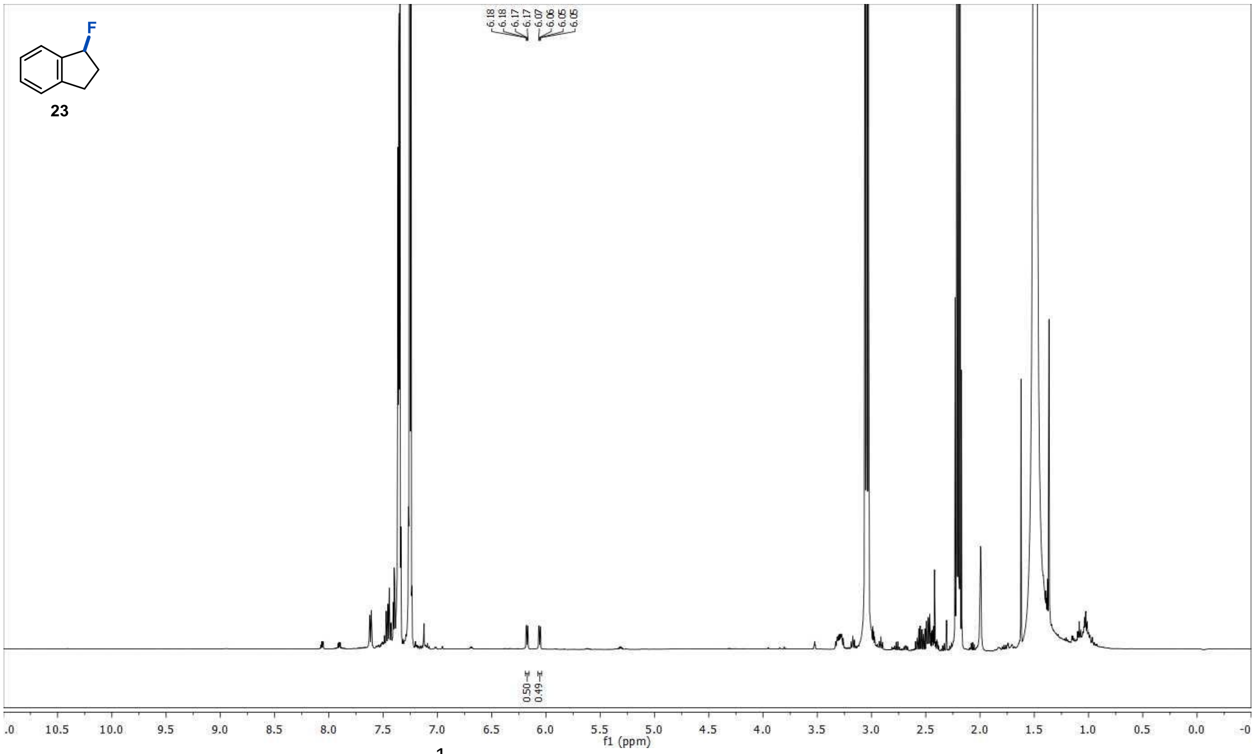

Supplementary Figure 107. ^{1}H NMR (500 MHz, $CDCl_3$) of **23** crude reaction mixture.

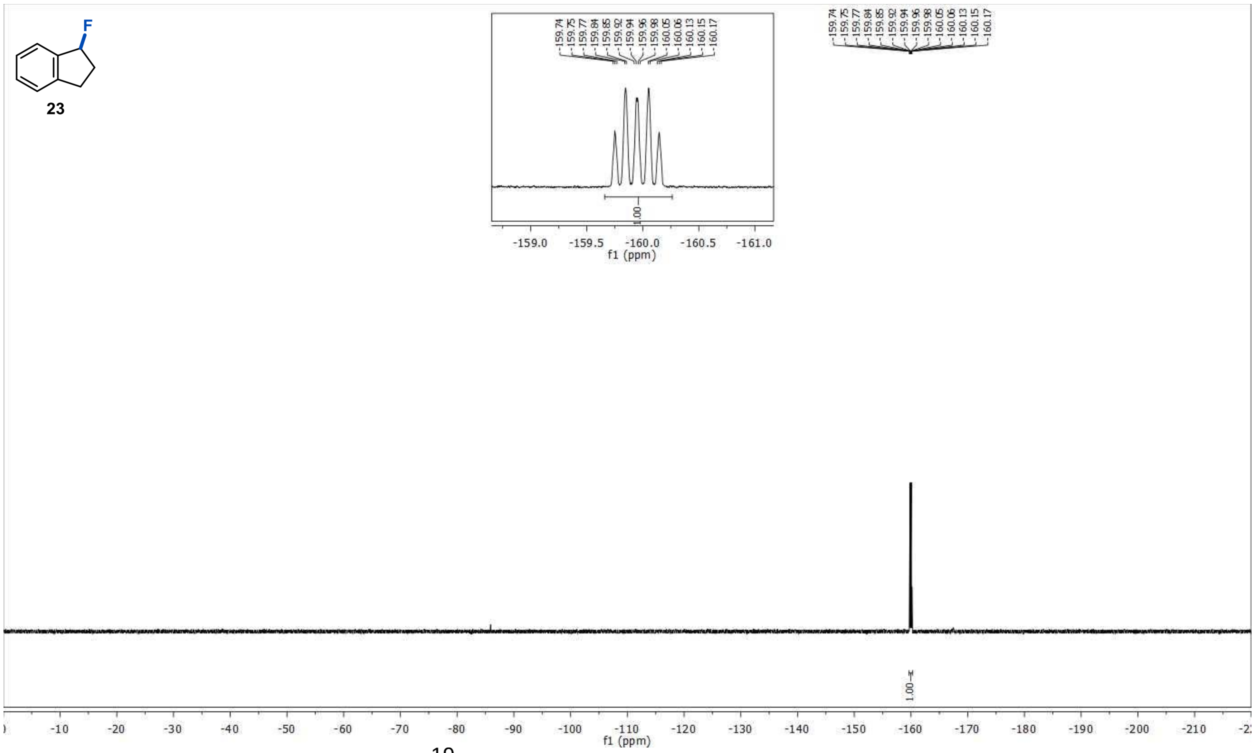

Supplementary Figure 108. ^{19}F NMR (282 MHz, $CDCl_3$) of **23** crude reaction mixture.

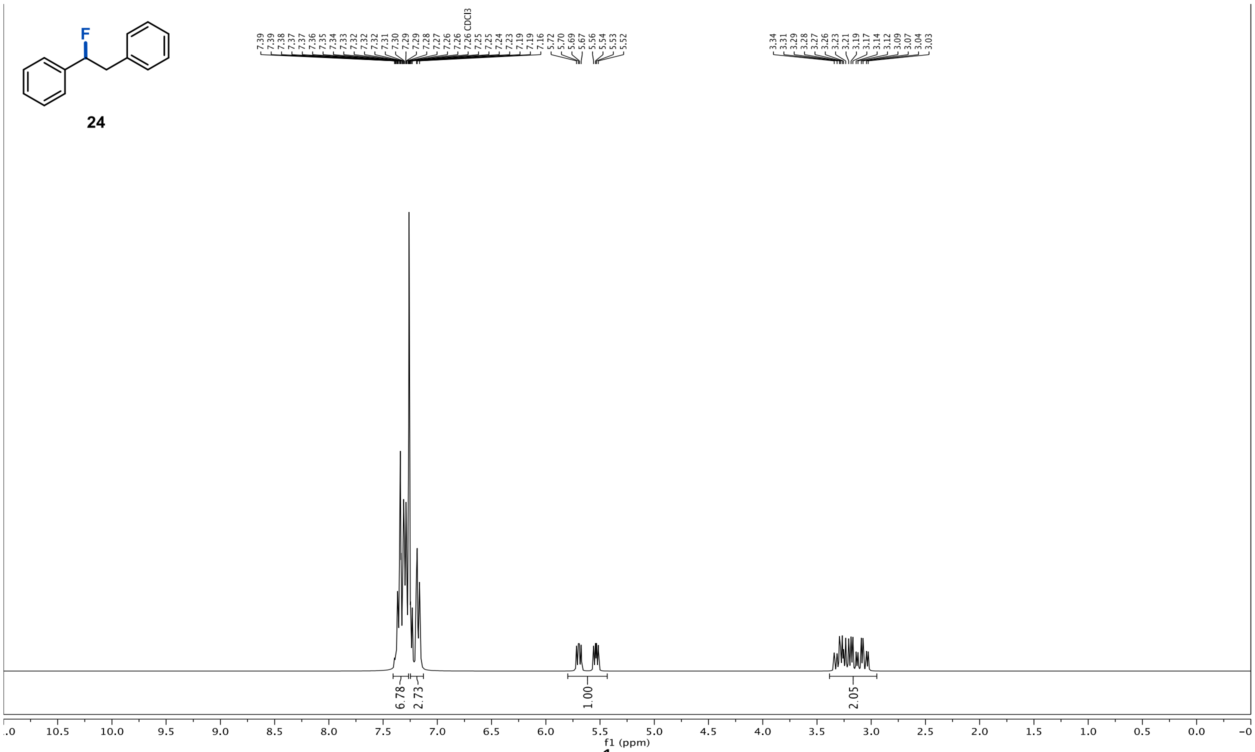

Supplementary Figure 109. ^{1}H NMR (500 MHz, $CDCl_3$) of **24**.

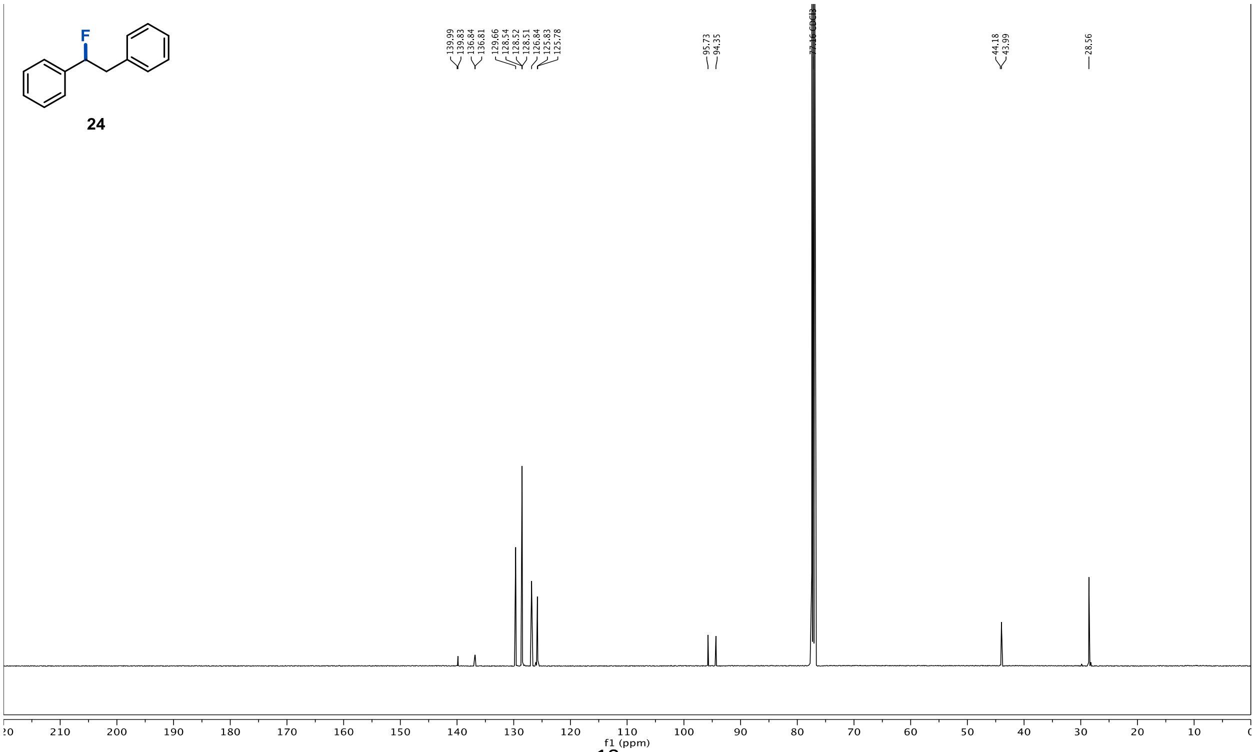

Supplementary Figure 110. ^{13}C NMR (126 MHz, $CDCl_3$) of **24**.

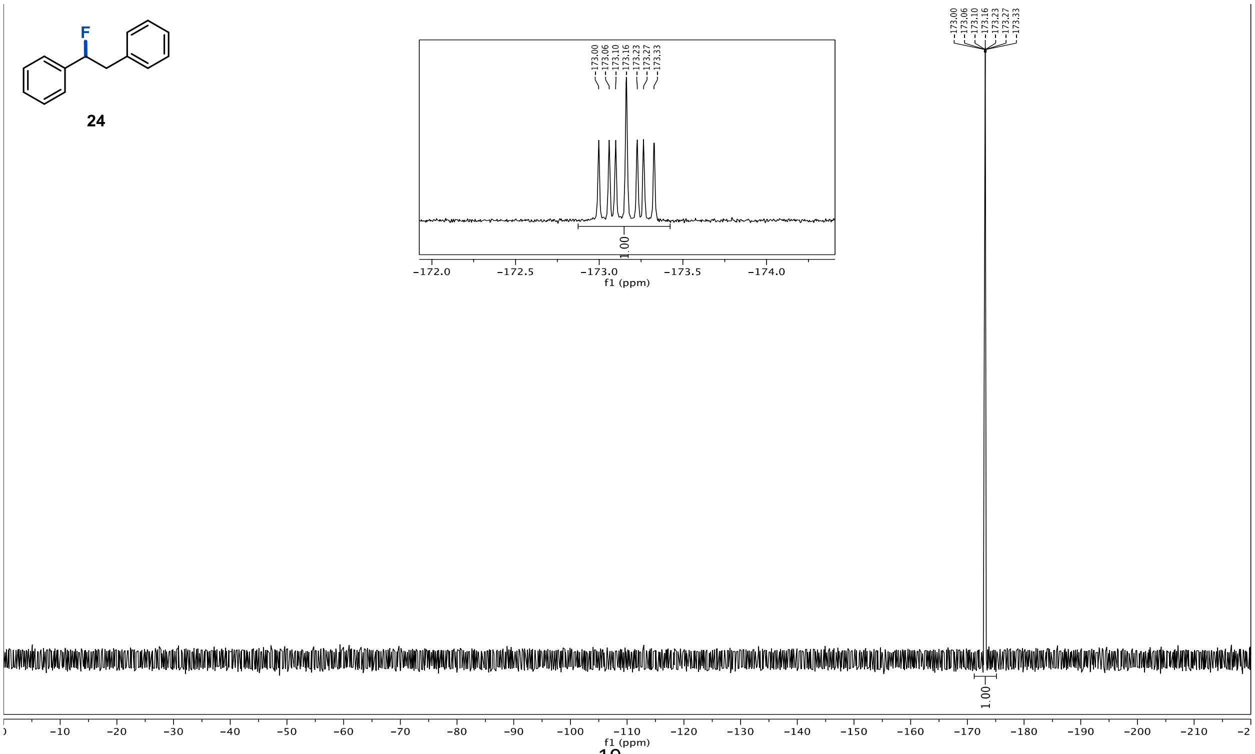

Supplementary Figure 111. ^{19}F NMR (282 MHz, $CDCl_3$) of **24**.

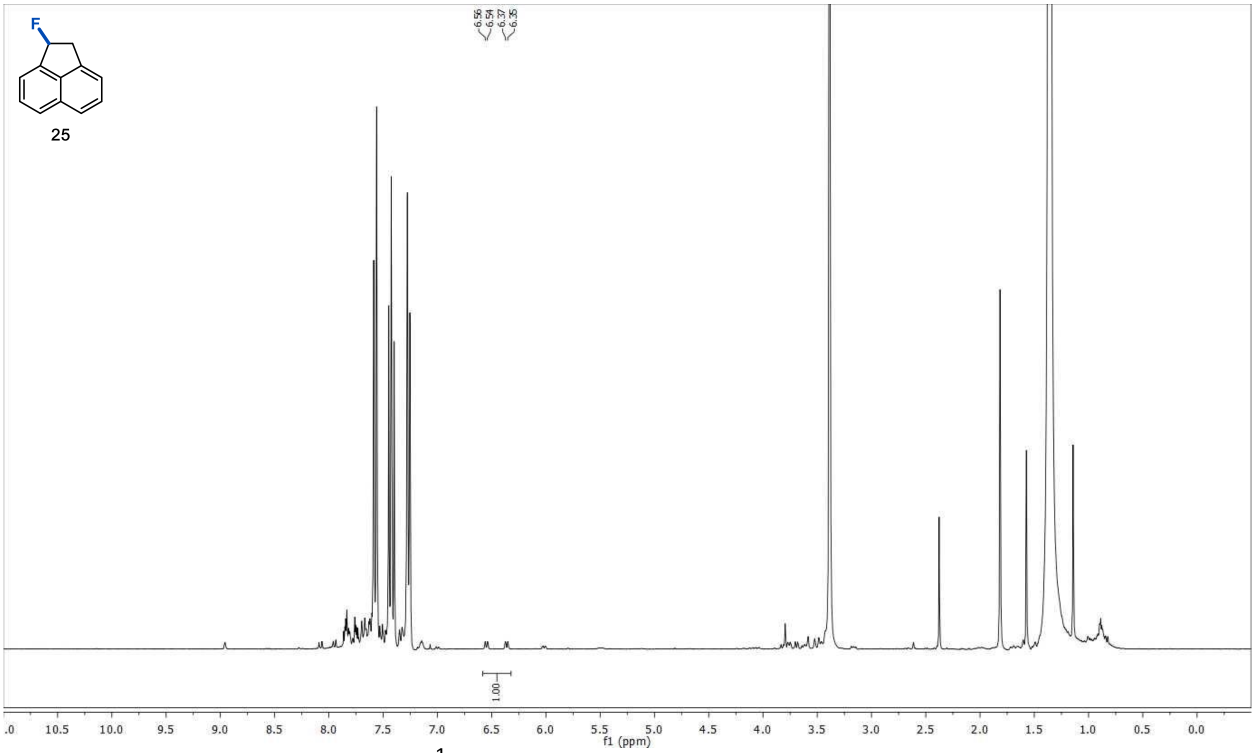

Supplementary Figure 112. ^{1}H NMR (500 MHz, $CDCl_3$) of **25** crude reaction mixture.

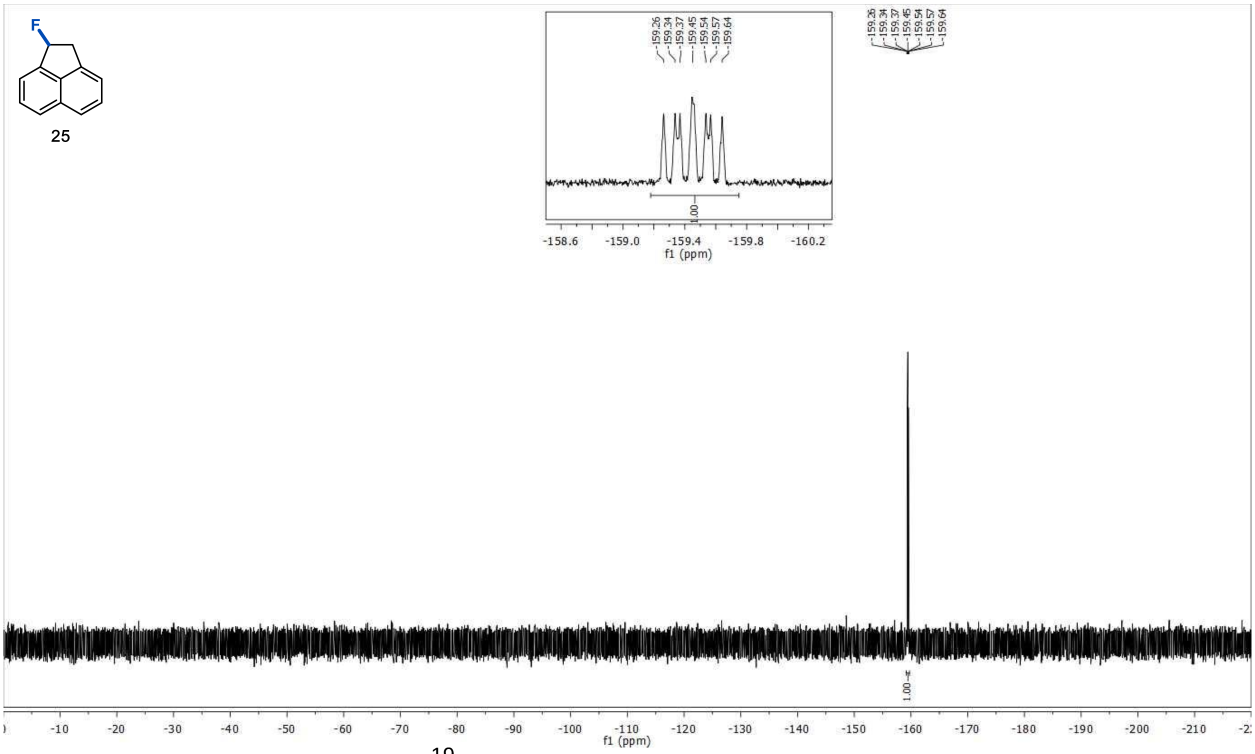

Supplementary Figure 113. ^{19}F NMR (282 MHz, $CDCl_3$) of **25** crude reaction mixture.

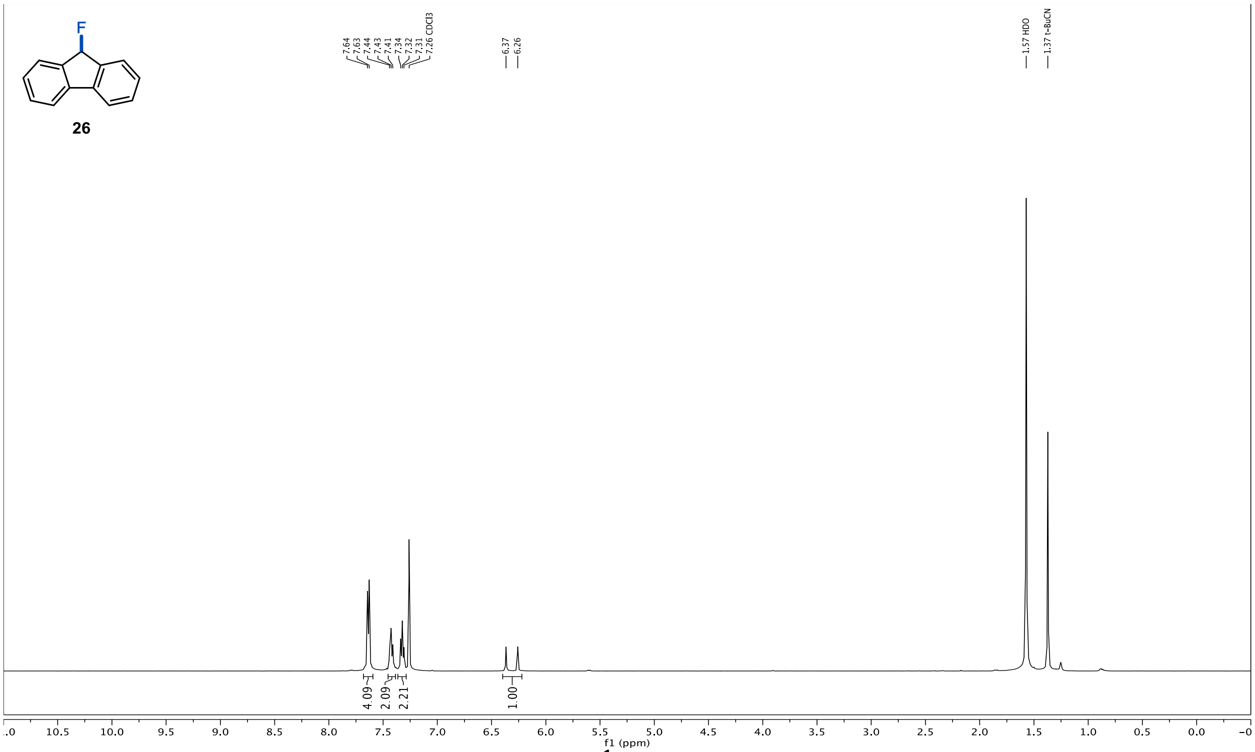

Supplementary Figure 114. ^{1}H NMR (500 MHz, $CDCl_3$) of **26**.

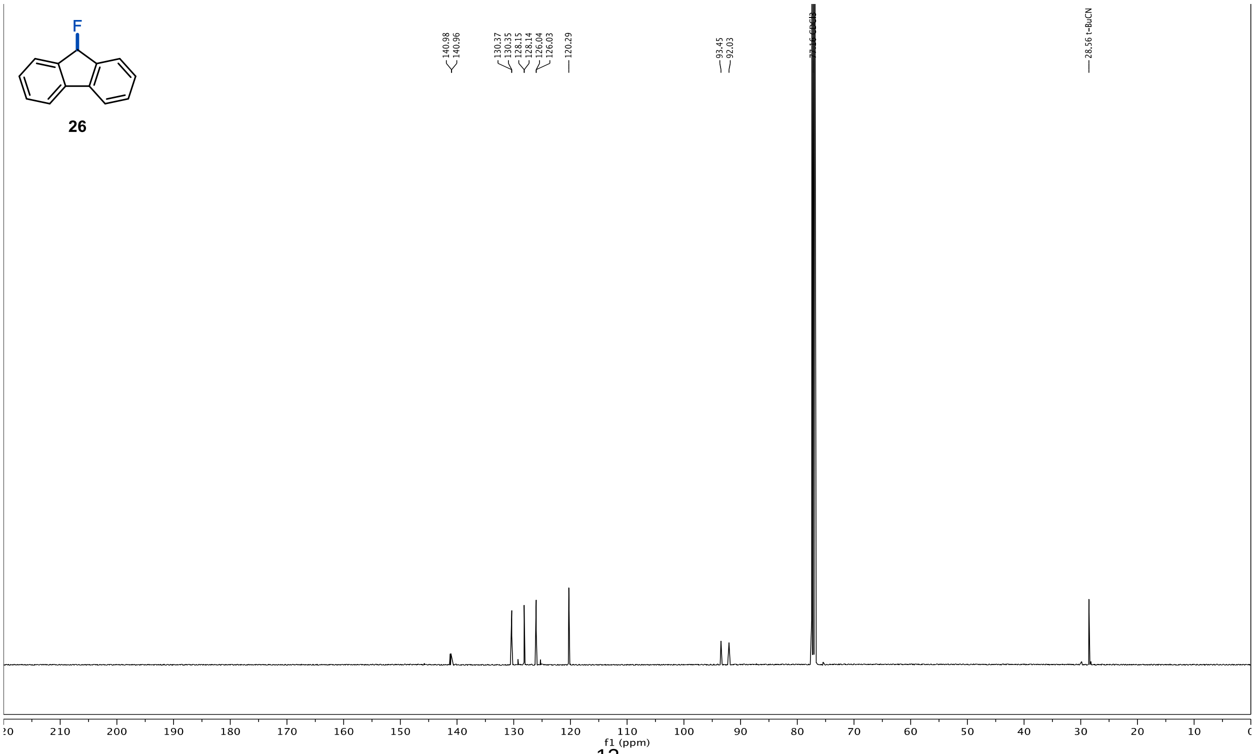

Supplementary Figure 115. ^{13}C NMR (126 MHz, $CDCl_3$) of **26**.

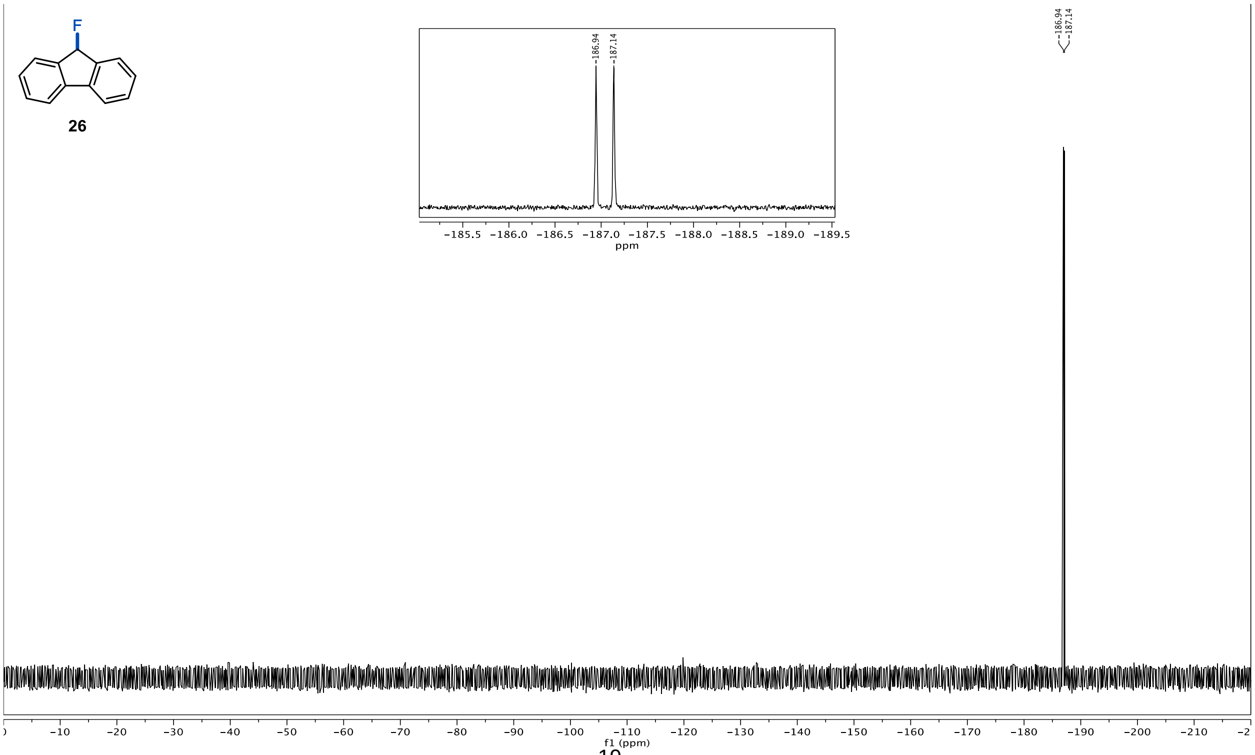

Supplementary Figure 116. ^{19}F NMR (282 MHz, $CDCl_3$) of **26**.

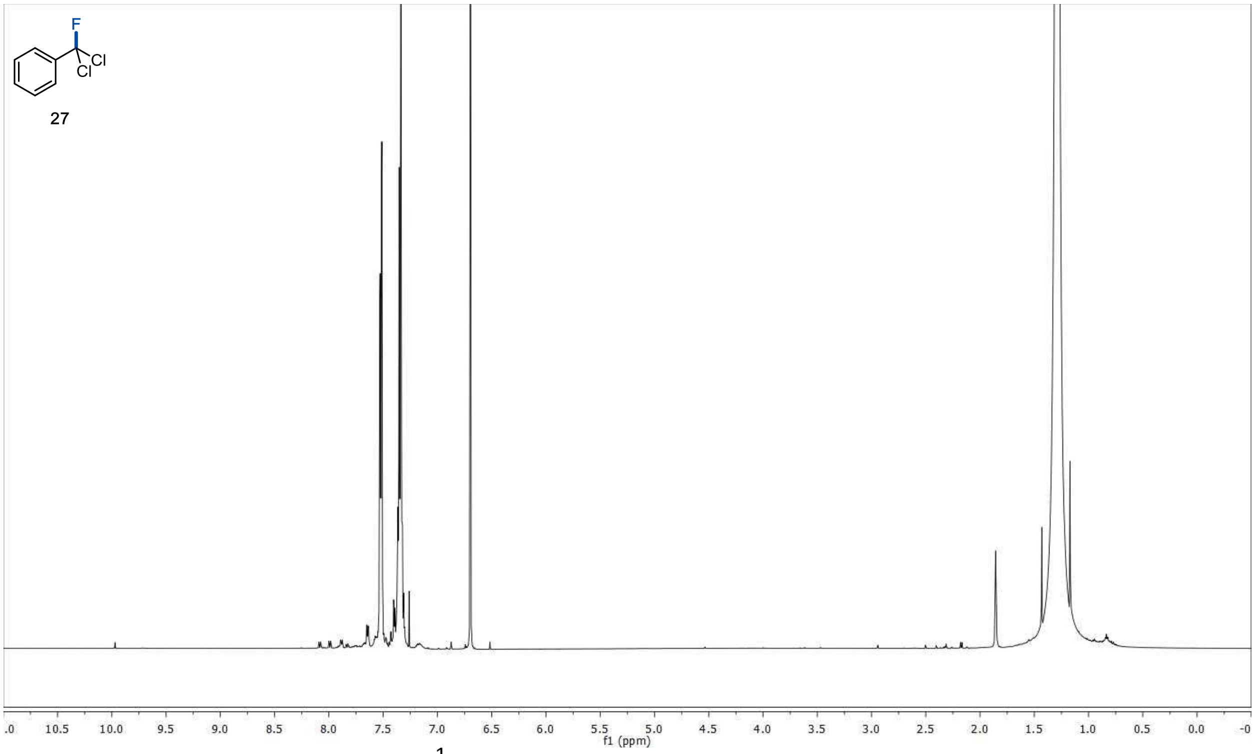

Supplementary Figure 117. ^{1}H NMR (500 MHz, $CDCl_3$) of **27** crude reaction mixture.

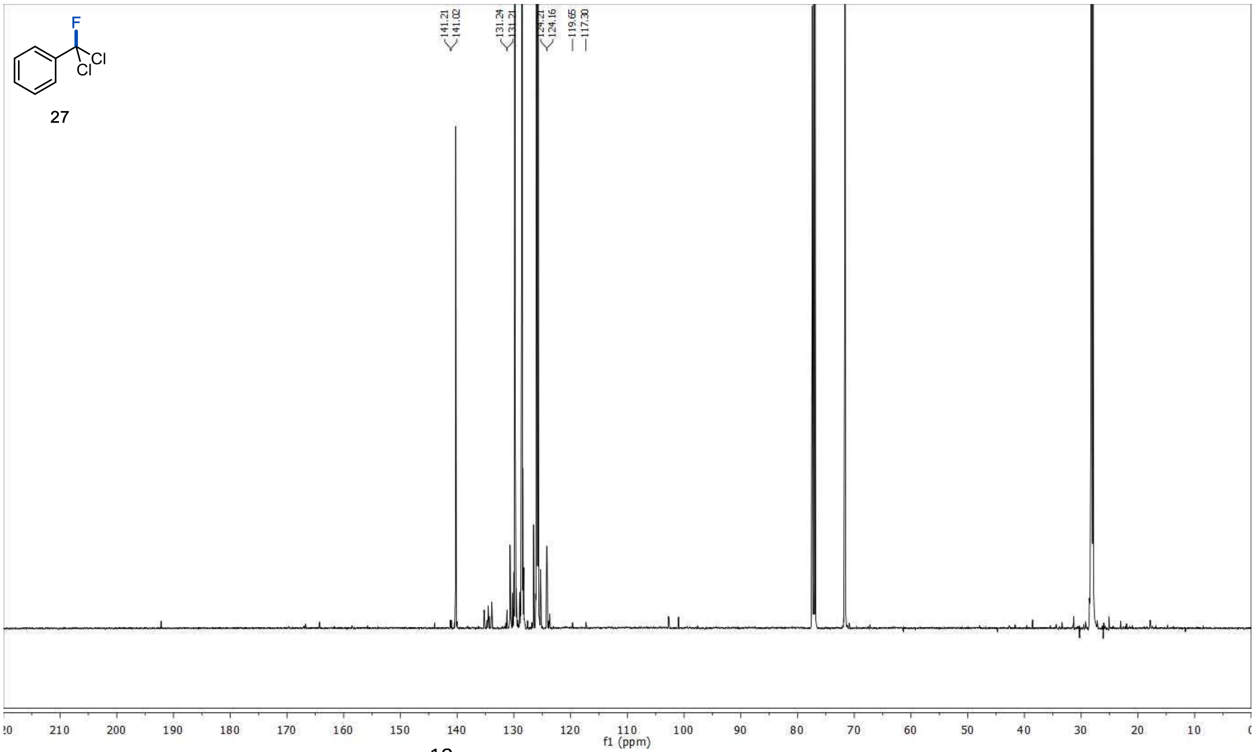

Supplementary Figure 118. ^{13}C NMR (126 MHz, $CDCl_3$) of **27** crude reaction mixture.

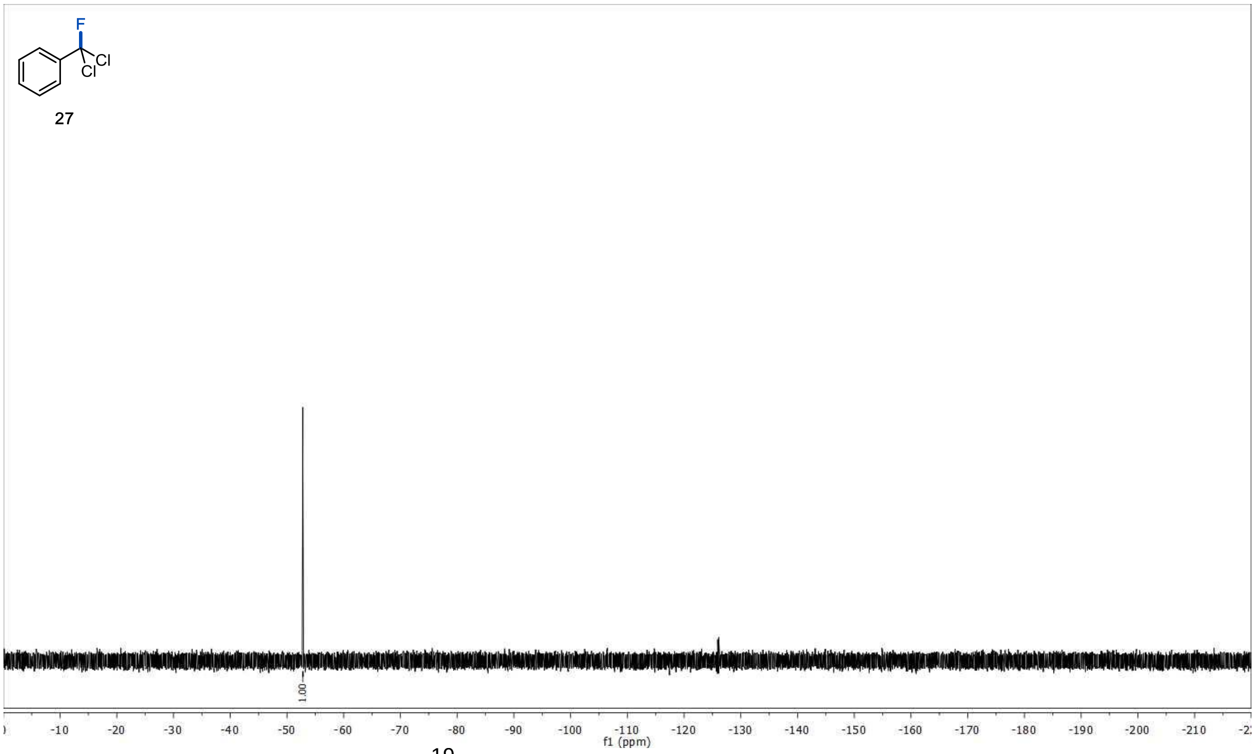

Supplementary Figure 119. ^{19}F NMR (282 MHz, $CDCl_3$) of **27** crude reaction mixture.

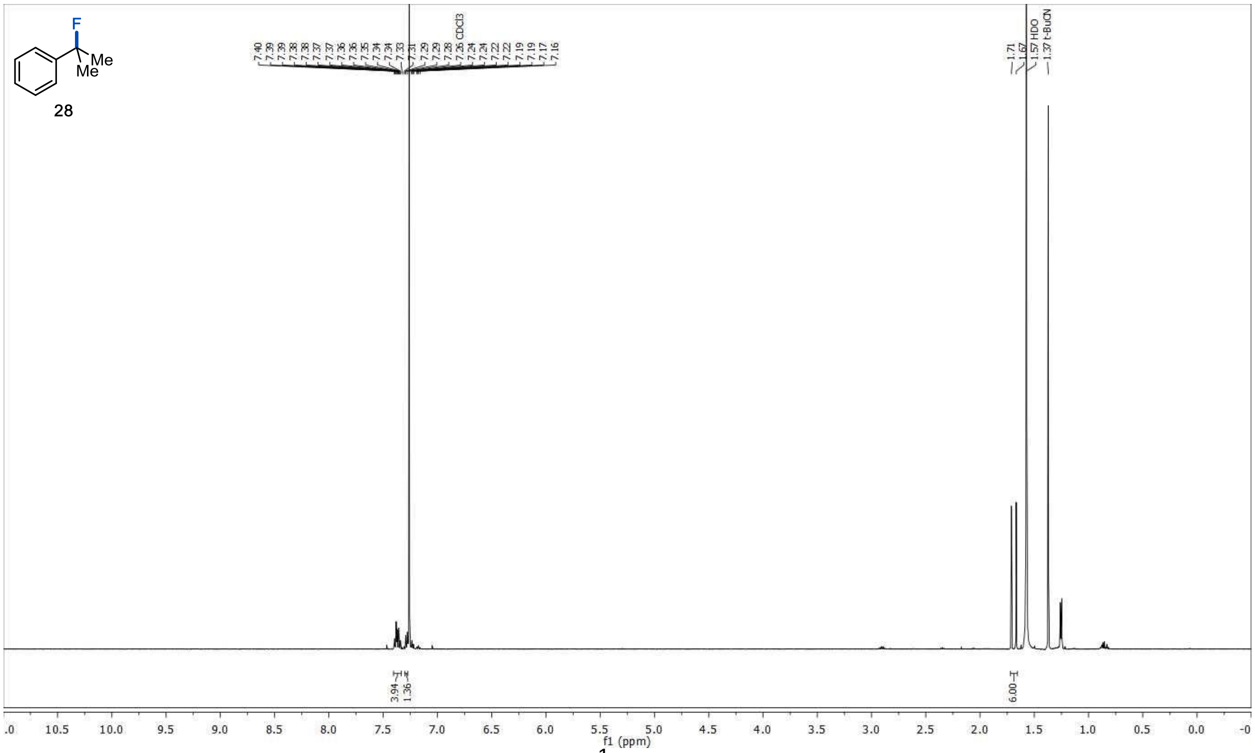

Supplementary Figure 120. ^{1}H NMR (500 MHz, $CDCl_3$) of **28**.

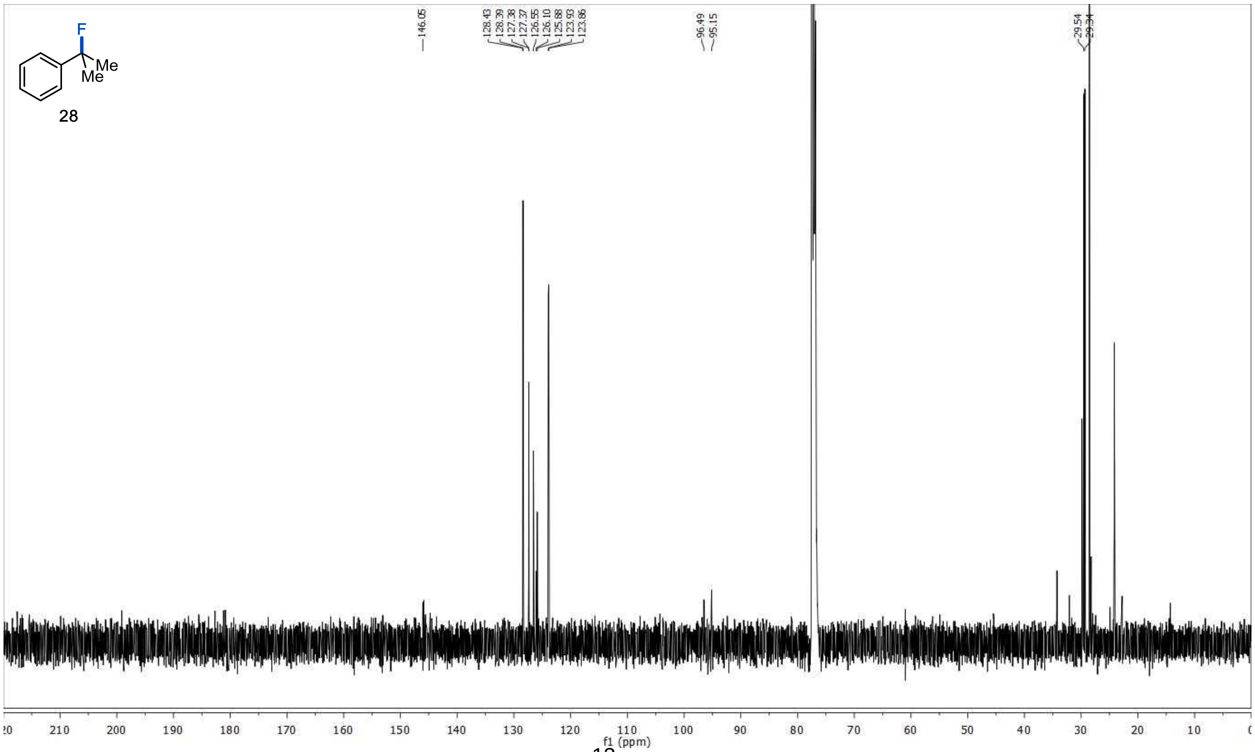

Supplementary Figure 121. ^{13}C NMR (126 MHz, $CDCl_3$) of **28**.

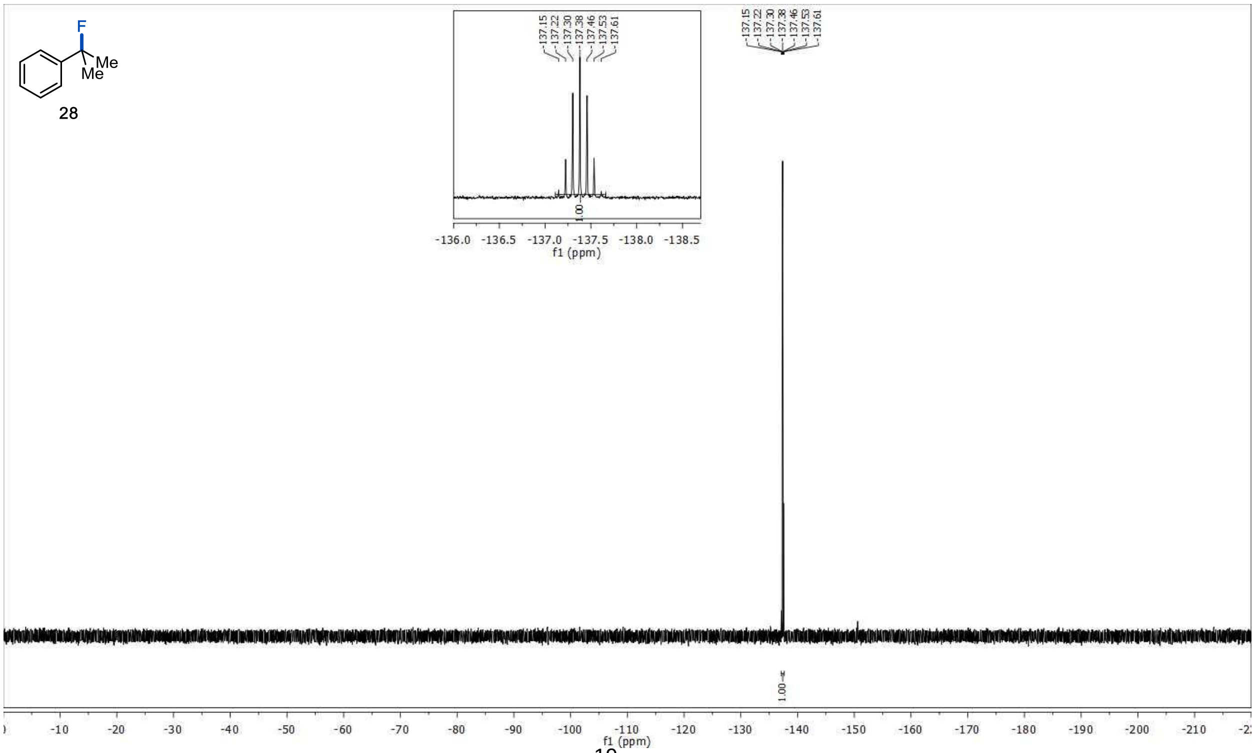

Supplementary Figure 122. ^{19}F NMR (282 MHz, $CDCl_3$) of **28**.

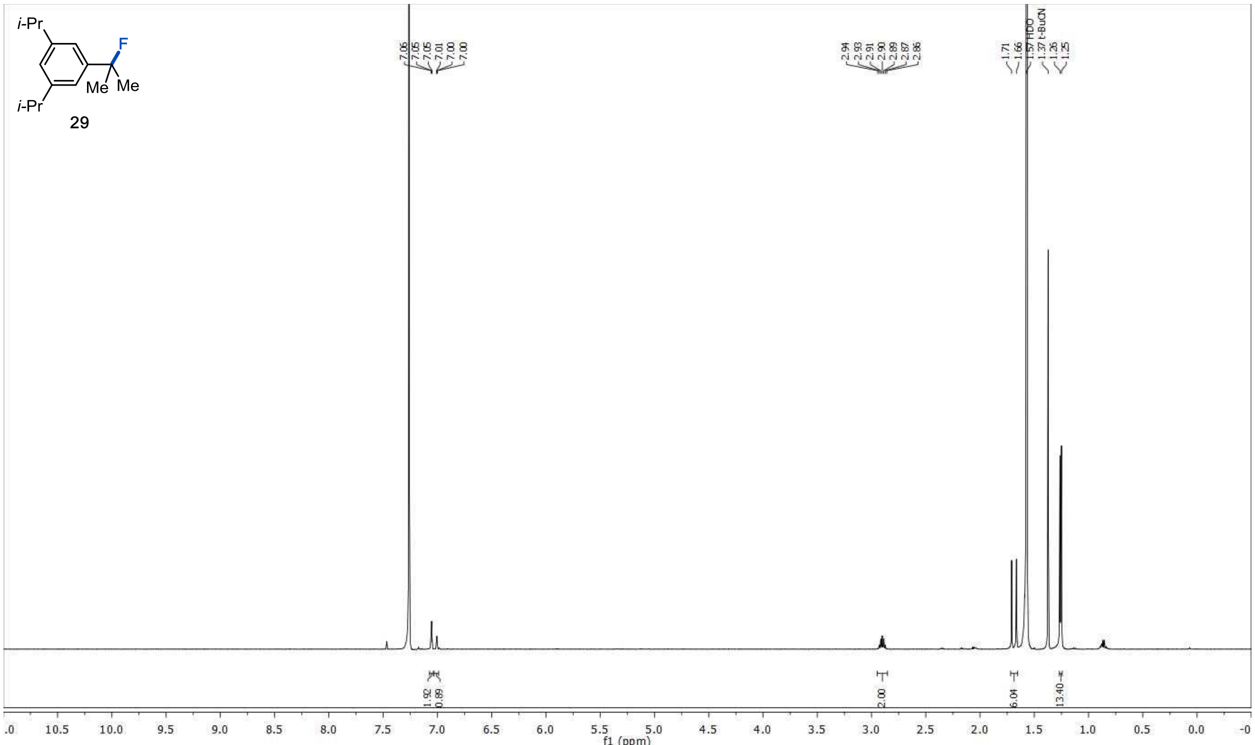

Supplementary Figure 123. ^{1}H NMR (500 MHz, $CDCl_3$) of **29**.

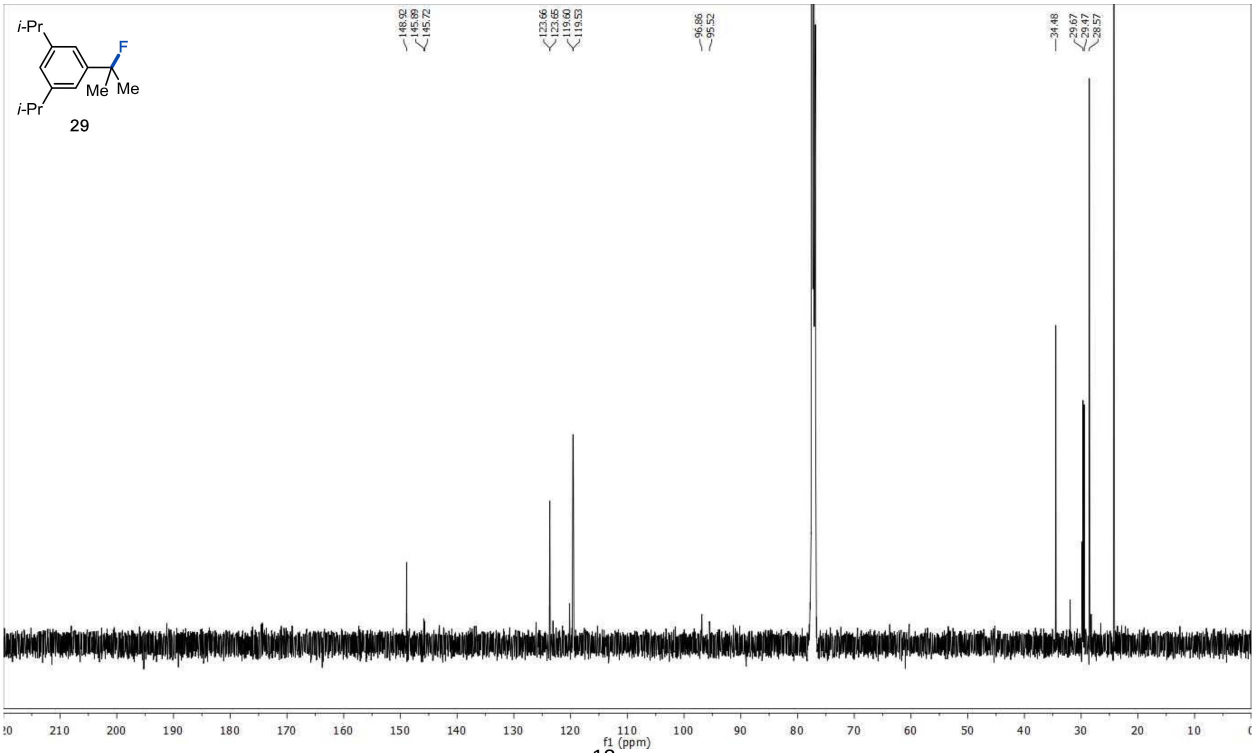

Supplementary Figure 124. ^{13}C NMR (126 MHz, $CDCl_3$) of **29**.

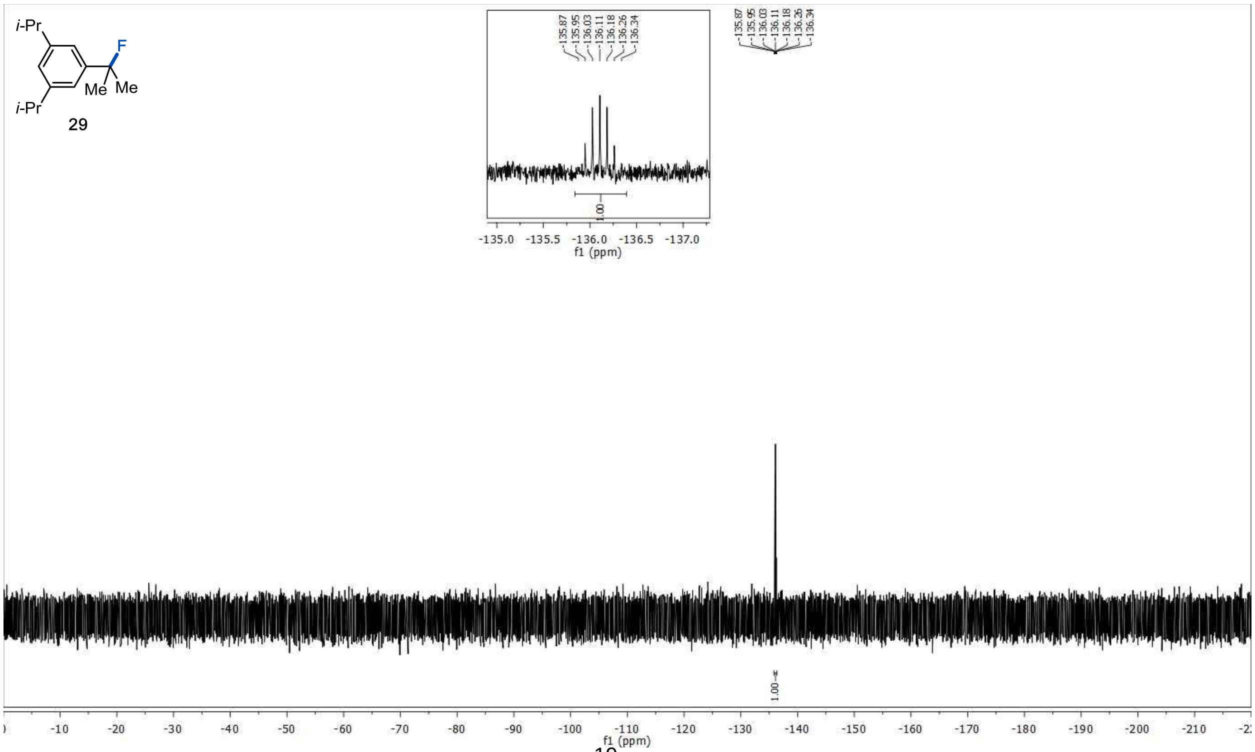

Supplementary Figure 125. ^{19}F NMR (282 MHz, $CDCl_3$) of **29**.

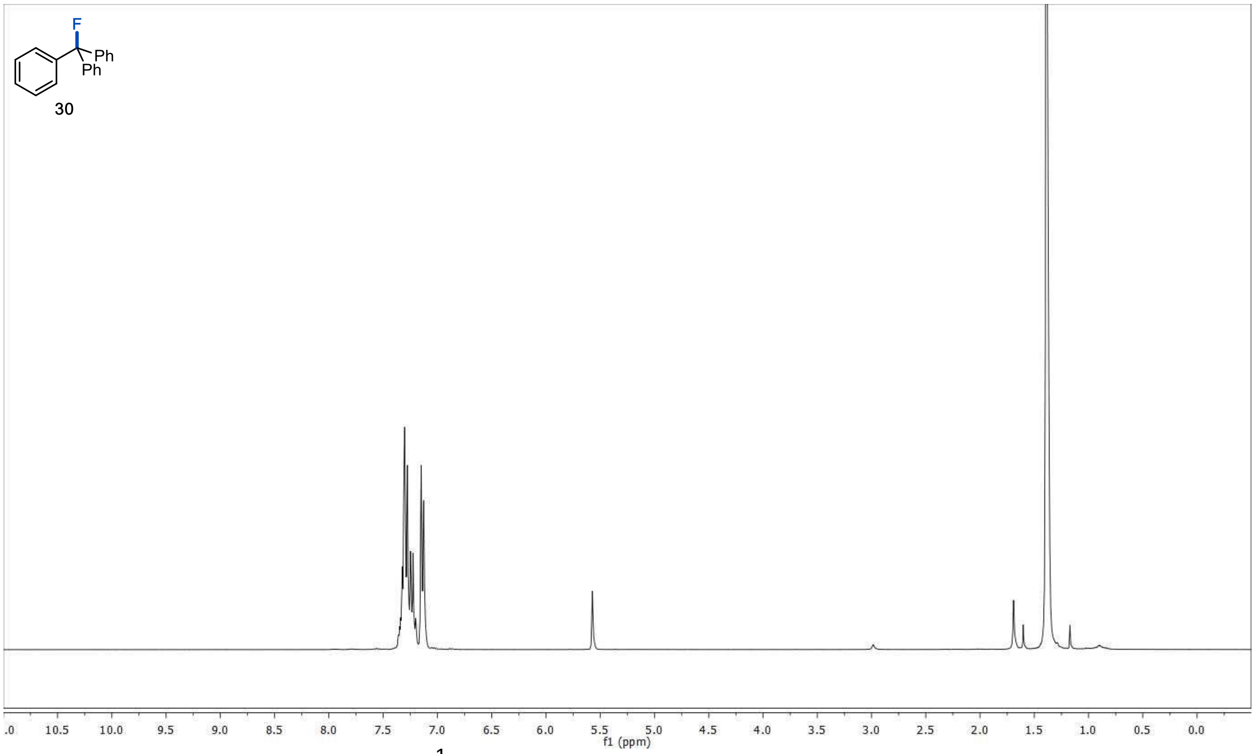

Supplementary Figure 126. ^{1}H NMR (500 MHz, $CDCl_3$) of **30** crude reaction mixture.

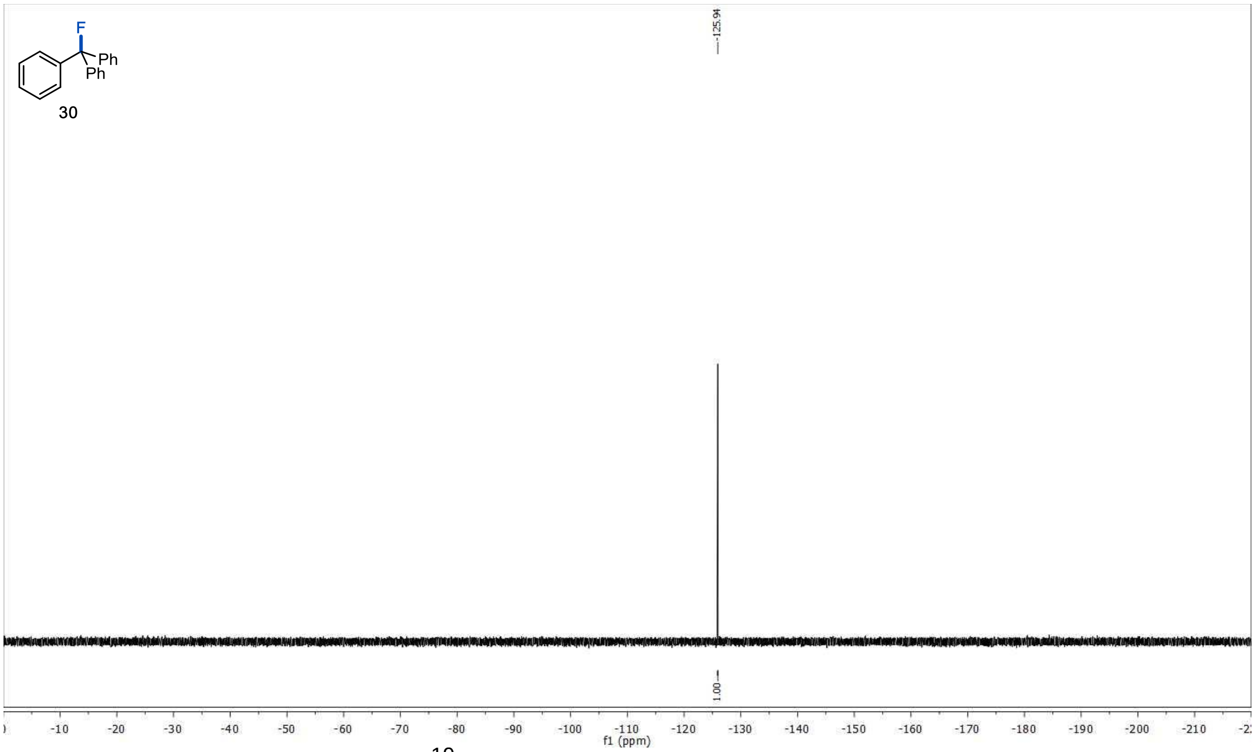

Supplementary Figure 127. ^{19}F NMR (282 MHz, $CDCl_3$) of **30** crude reaction mixture.

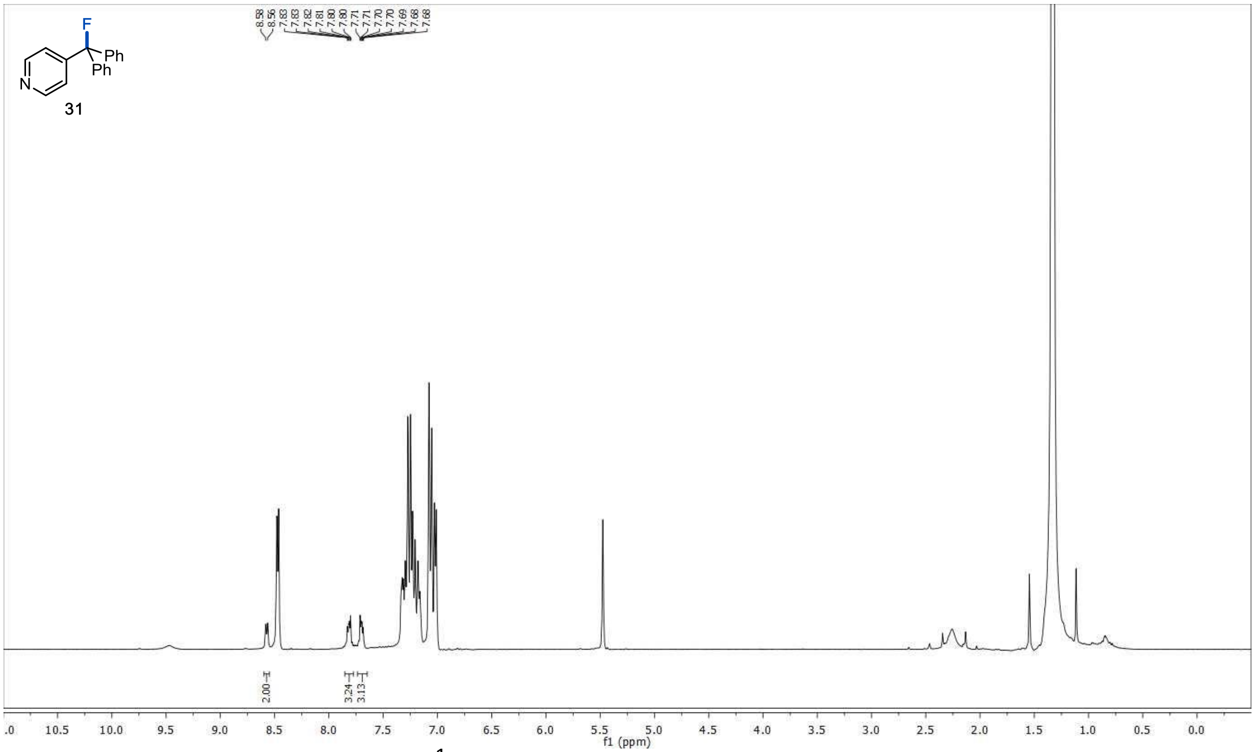

Supplementary Figure 128. ^{1}H NMR (500 MHz, $CDCl_3$) of **31** crude reaction mixture.

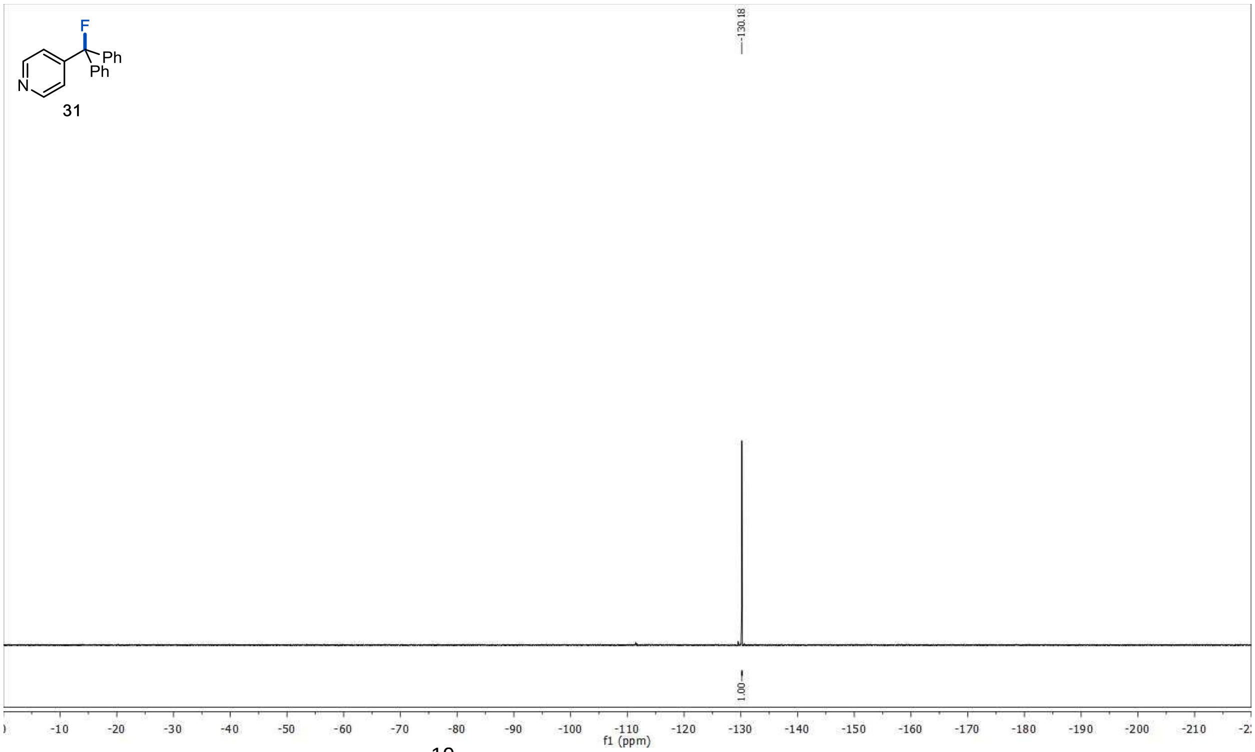

Supplementary Figure 129. ^{19}F NMR (282 MHz, $CDCl_3$) of **31** crude reaction mixture.

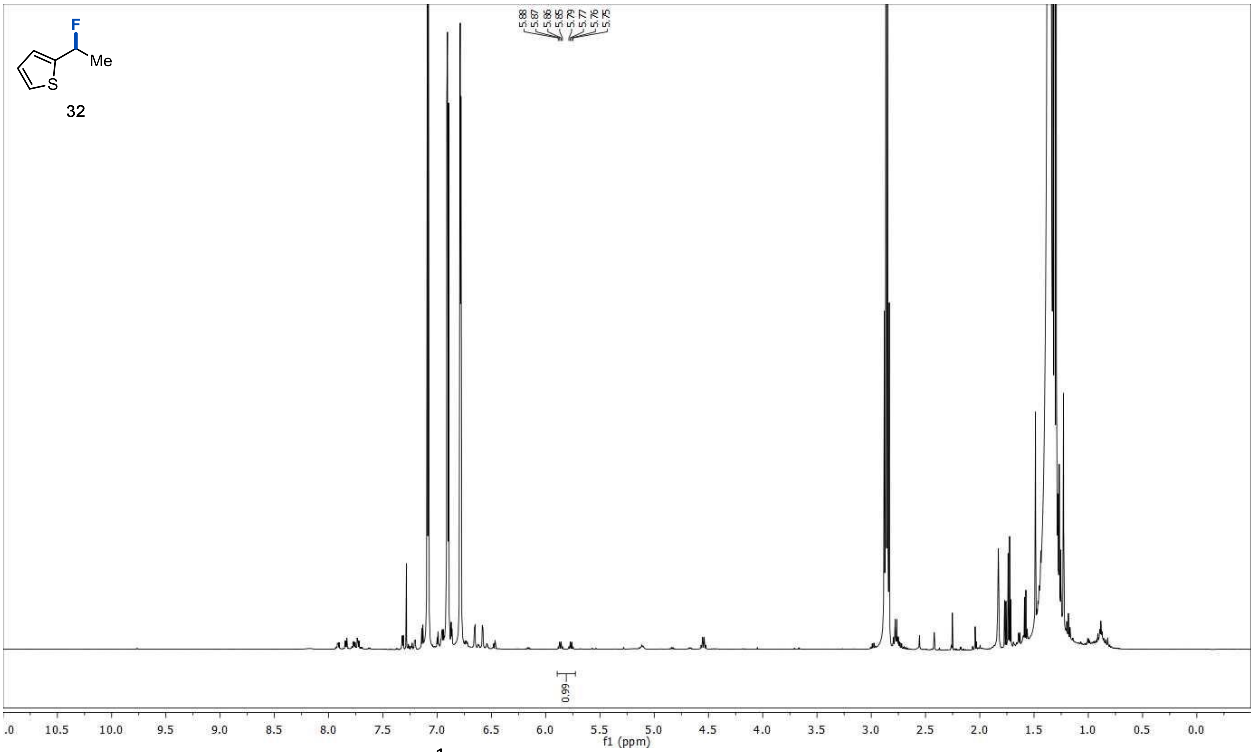

Supplementary Figure 130. ^{1}H NMR (500 MHz, $CDCl_3$) of **32** crude reaction mixture.

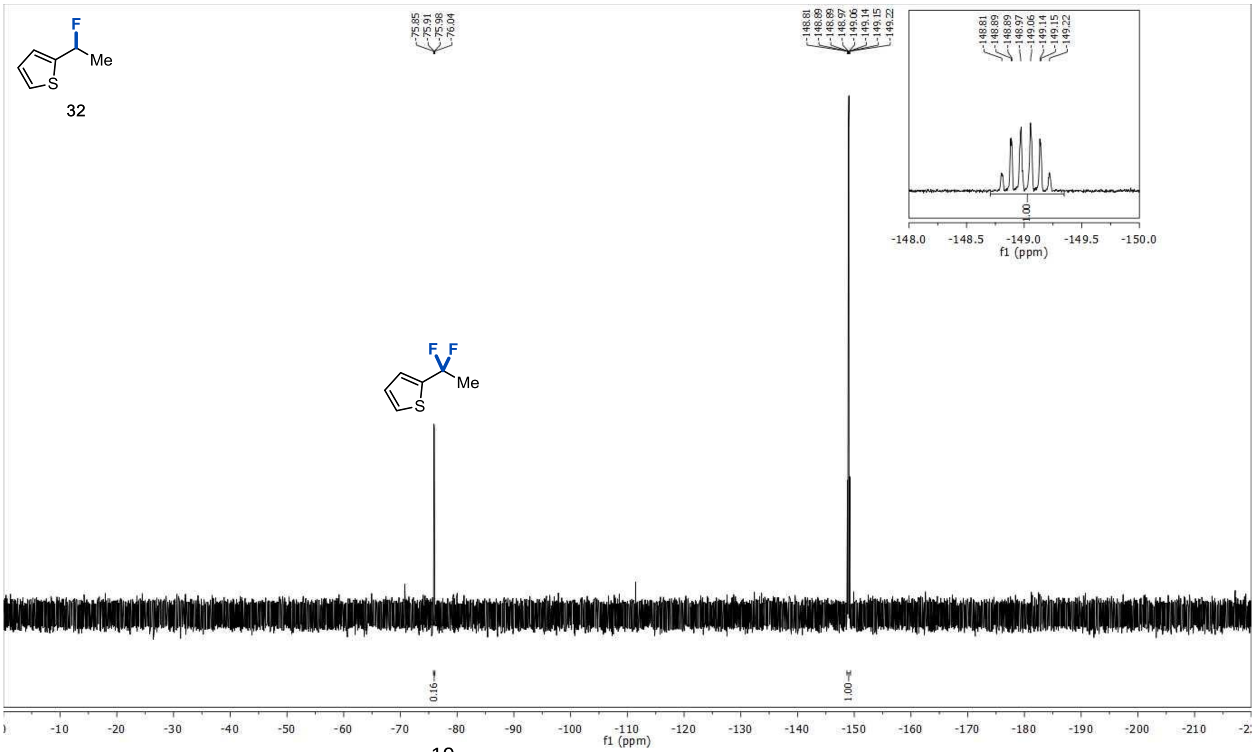

Supplementary Figure 131. ^{19}F NMR (282 MHz, $CDCl_3$) of **32** crude reaction mixture.

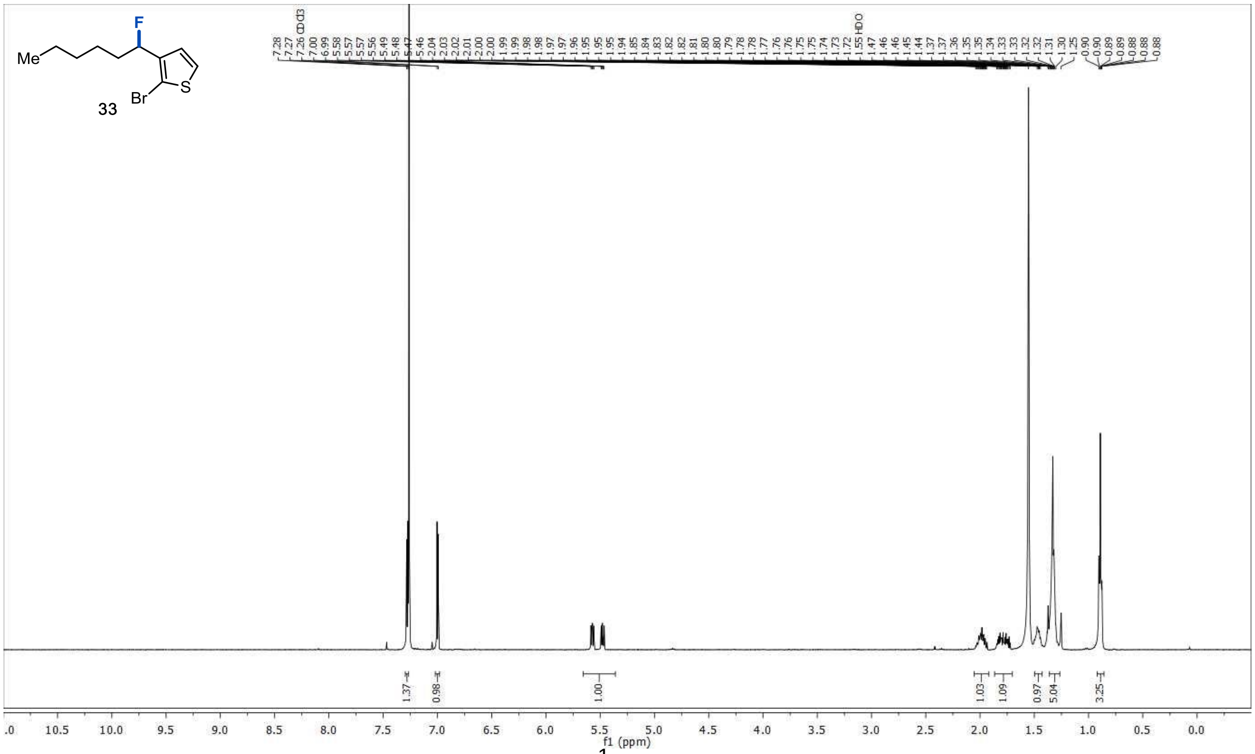

Supplementary Figure 132. ^{1}H NMR (500 MHz, $CDCl_3$) of **33**.

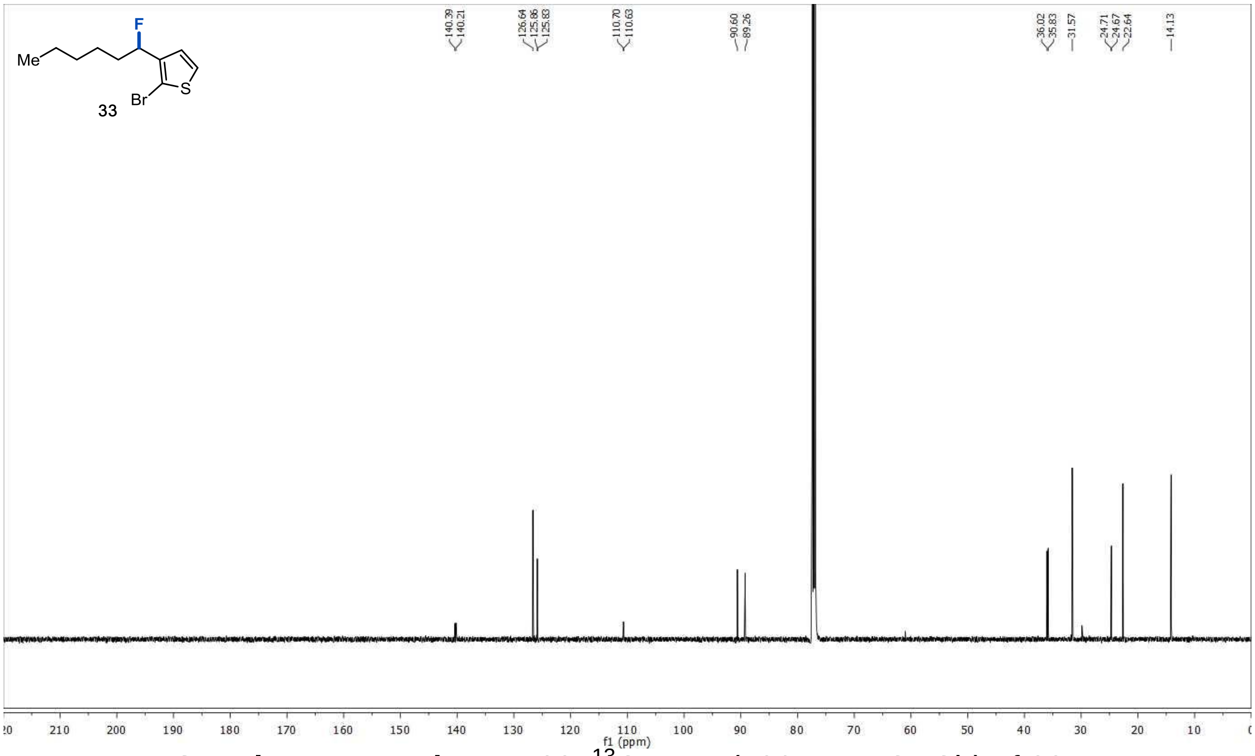

Supplementary Figure 133. ^{13}C NMR (126 MHz, $CDCl_3$) of **33**.

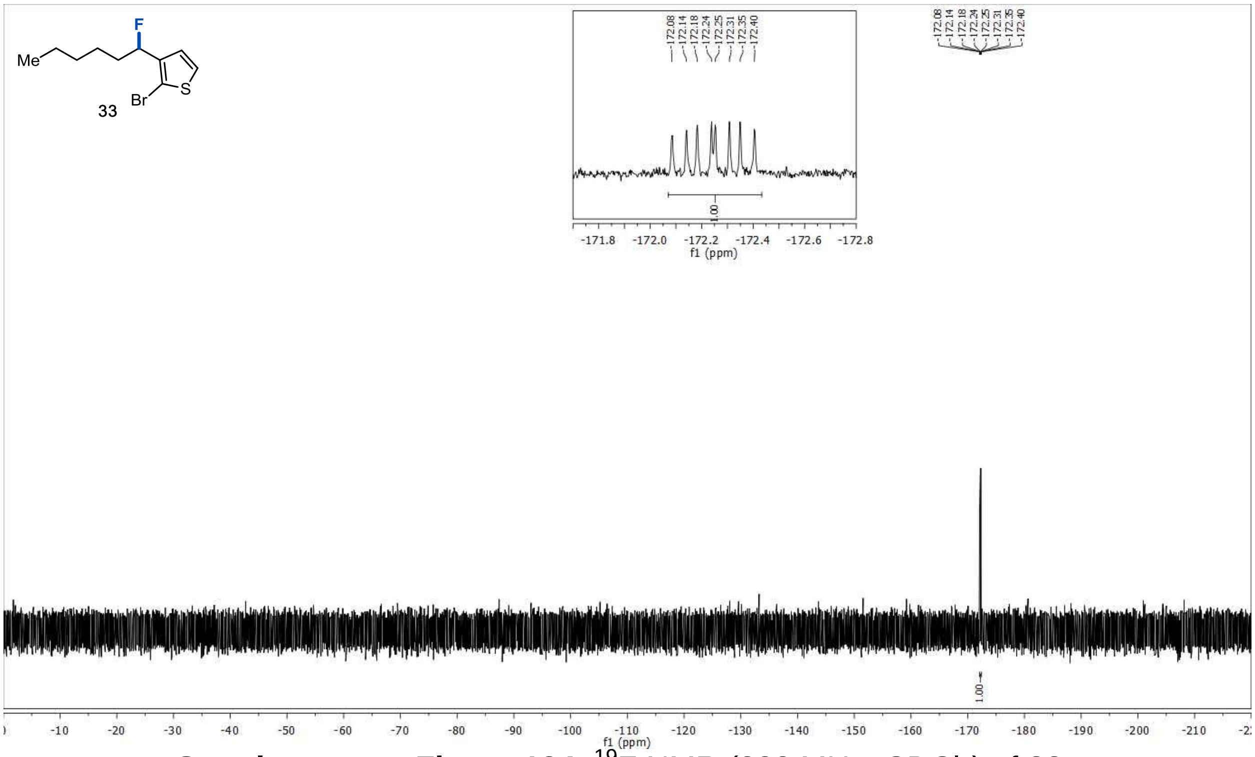

Supplementary Figure 134. ^{19}F NMR (282 MHz, $CDCl_3$) of **33**.

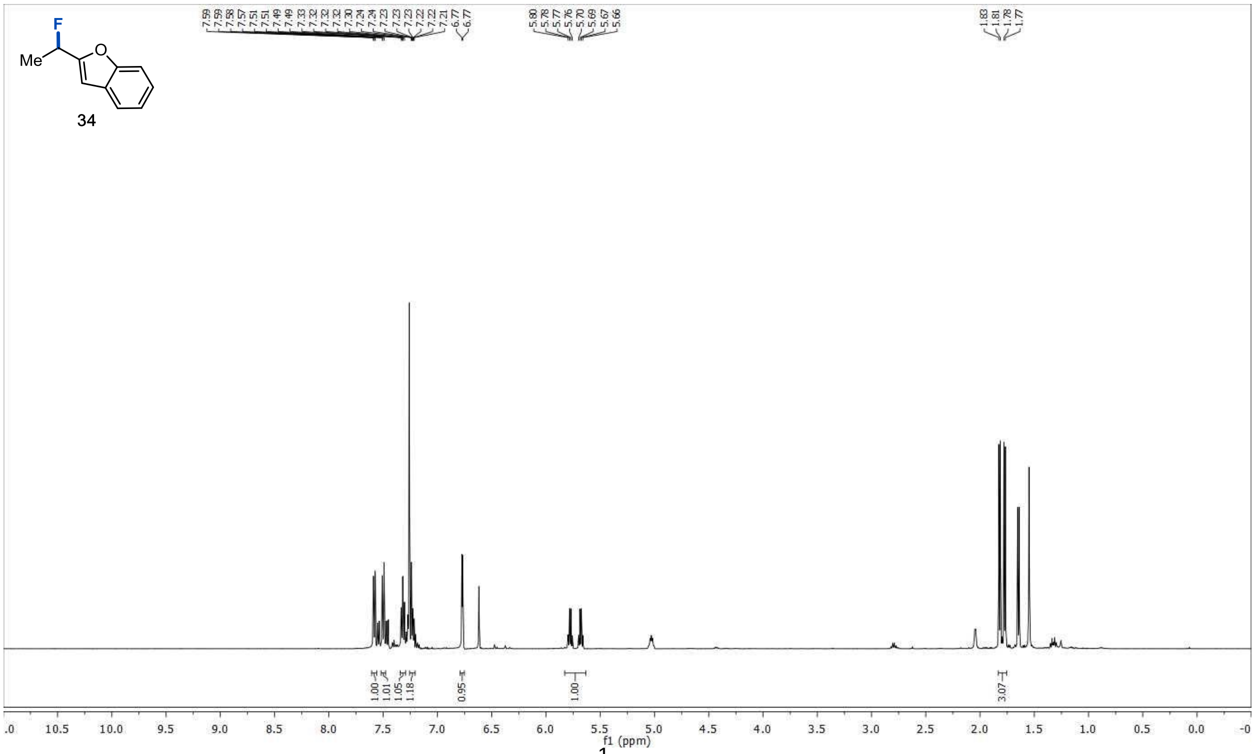

Supplementary Figure 135. ^{1}H NMR (500 MHz, $CDCl_3$) of **34**.

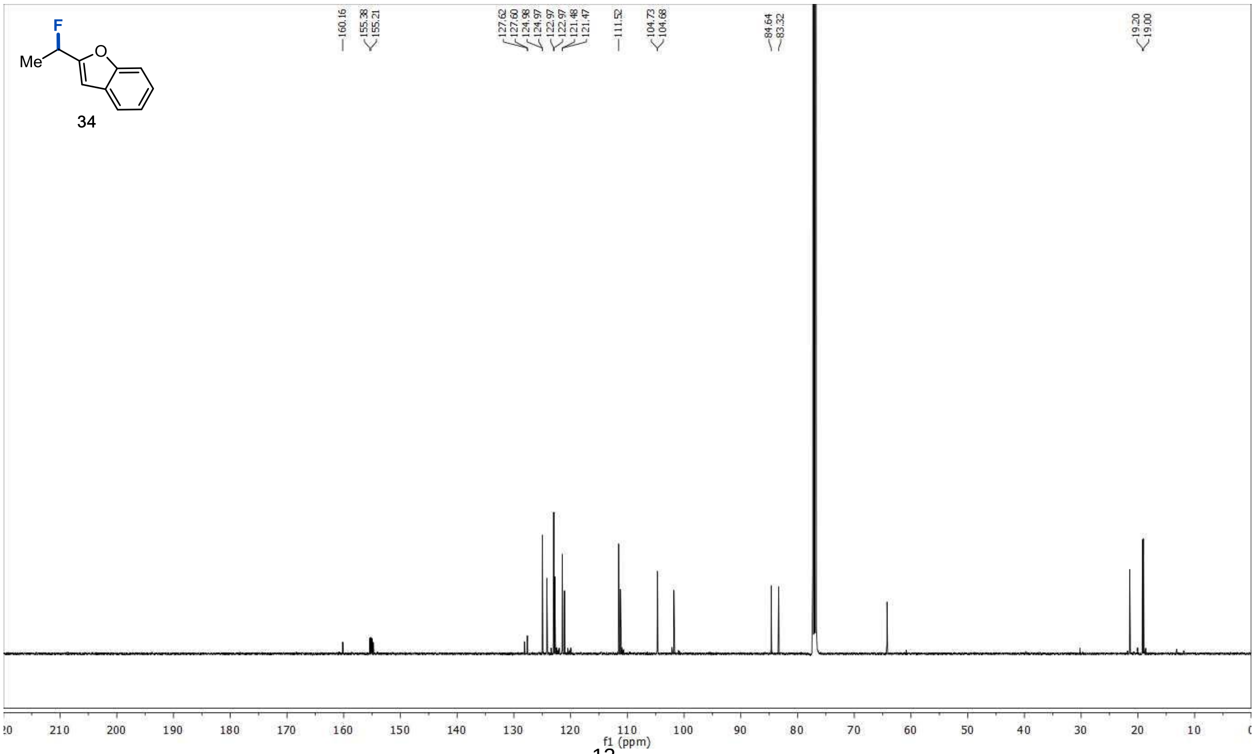

Supplementary Figure 136. ^{13}C NMR (126 MHz, $CDCl_3$) of **34**.

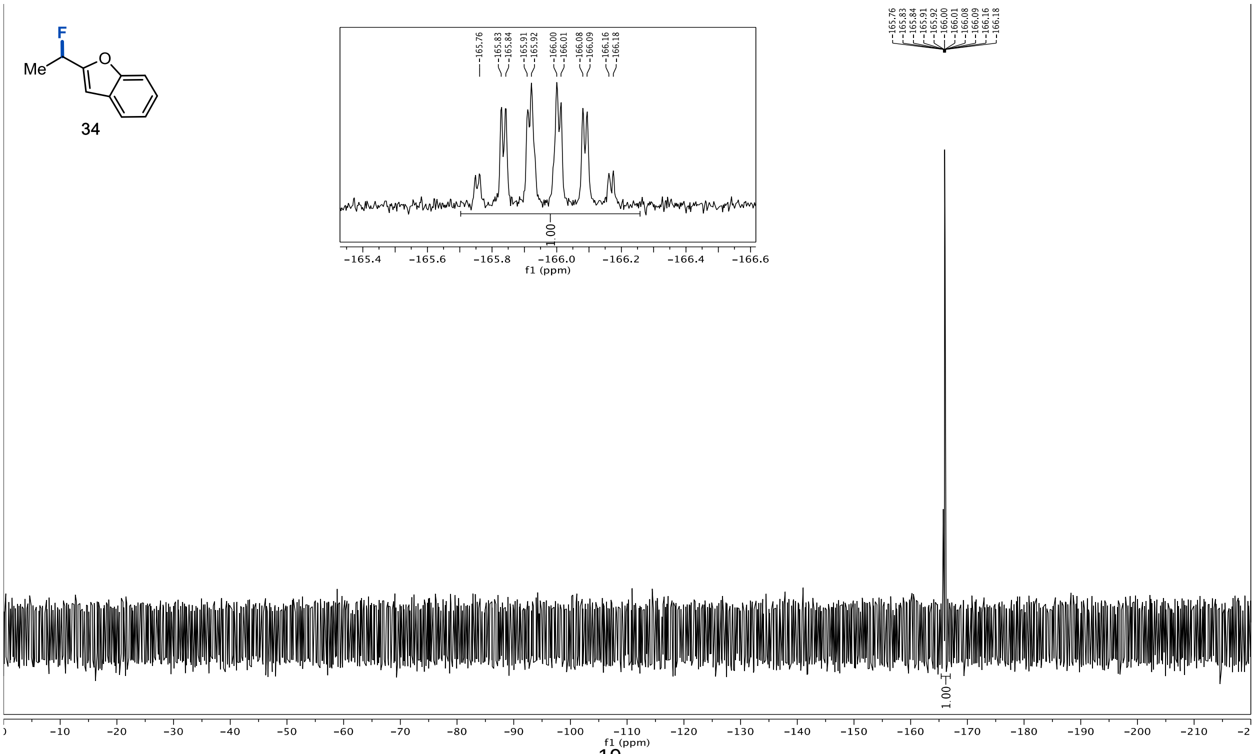

Supplementary Figure 137. ^{19}F NMR (282 MHz, $CDCl_3$) of **34**.

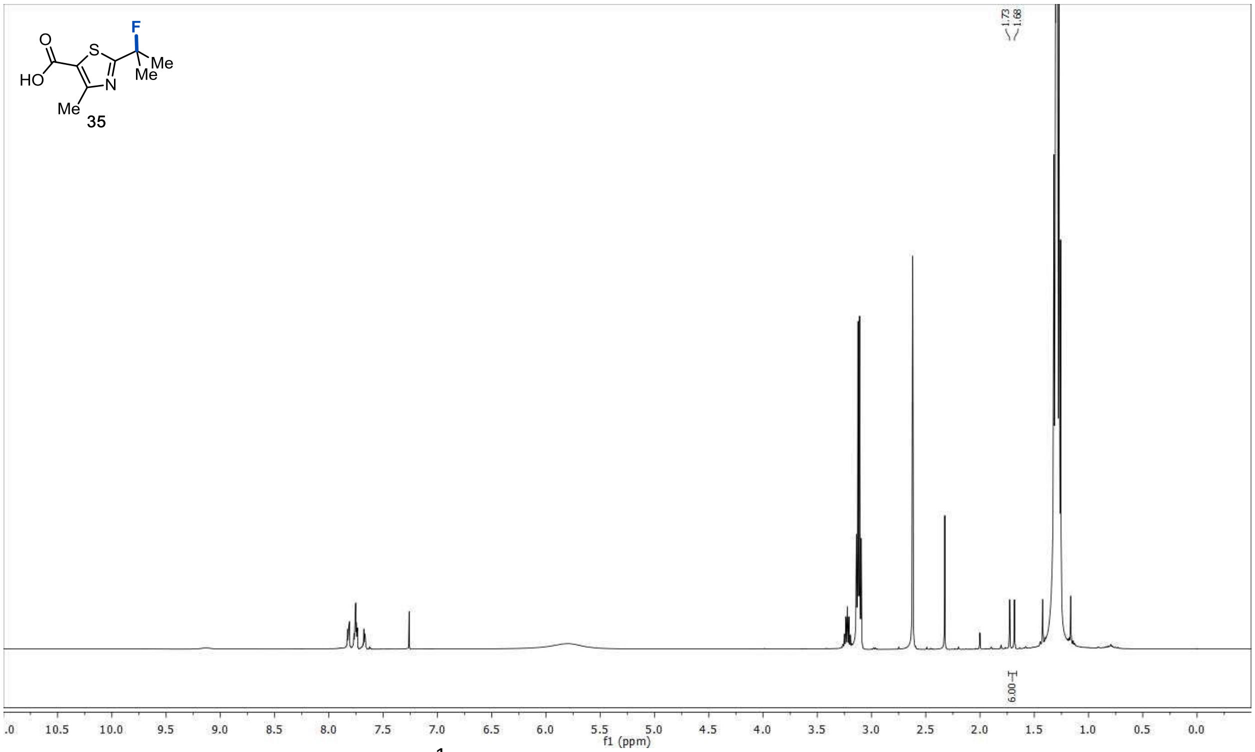

Supplementary Figure 138. ^{1}H NMR (500 MHz, $CDCl_3$) of **35** crude reaction mixture.

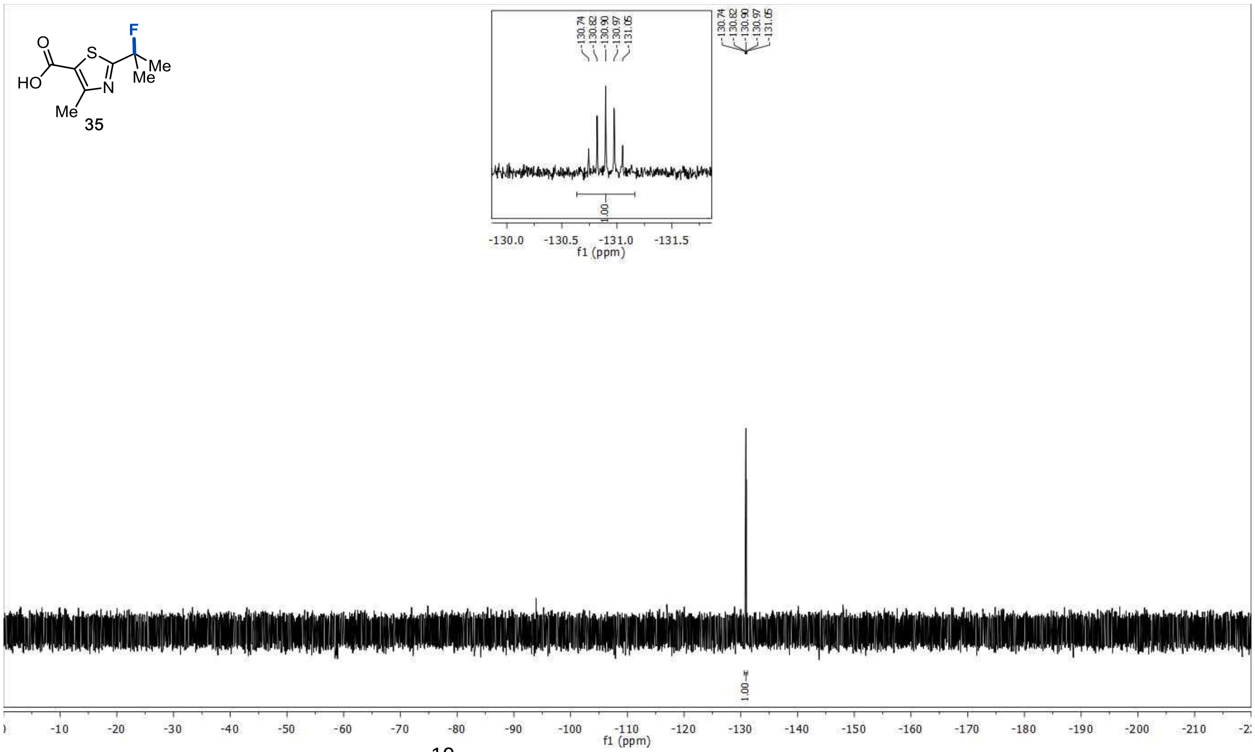

Supplementary Figure 139. ^{19}F NMR (282 MHz, $CDCl_3$) of **35** crude reaction mixture.

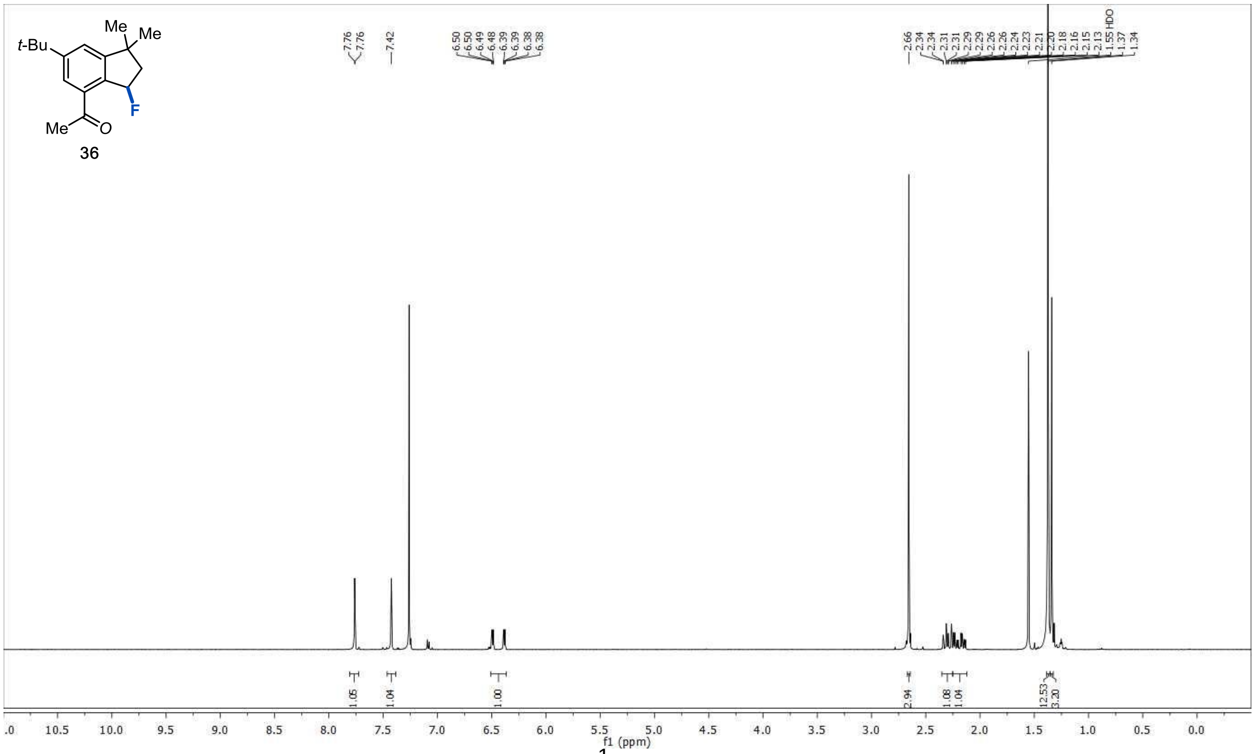

Supplementary Figure 140. ^{1}H NMR (500 MHz, $CDCl_3$) of **36**.

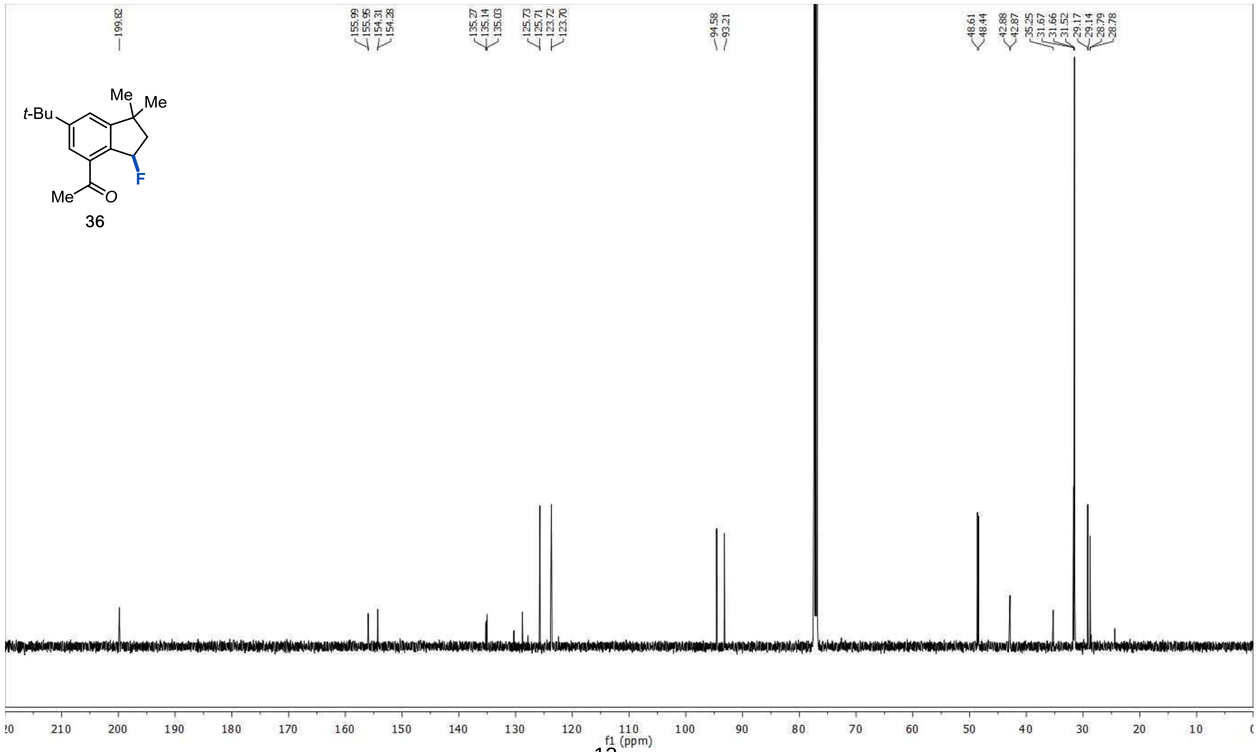

Supplementary Figure 141. ^{13}C NMR (126 MHz, $CDCl_3$) of **36**.

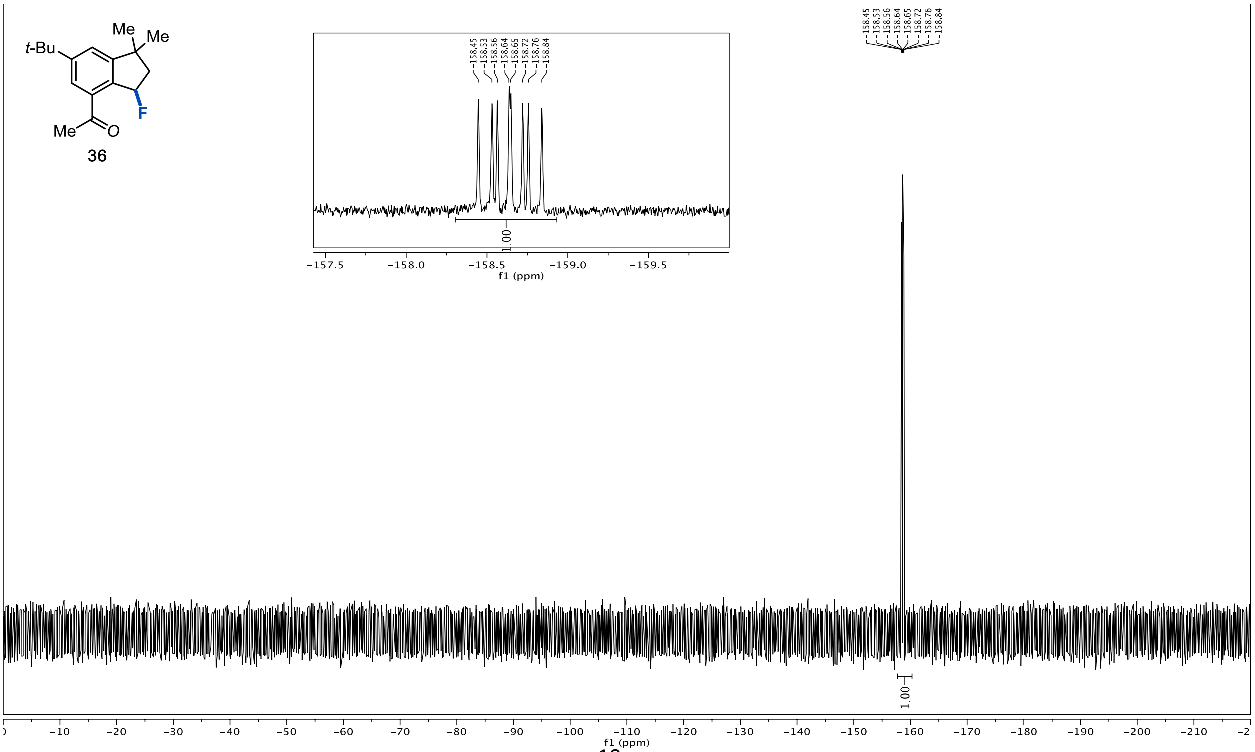

Supplementary Figure 142. ^{19}F NMR (282 MHz, $CDCl_3$) of **36**.

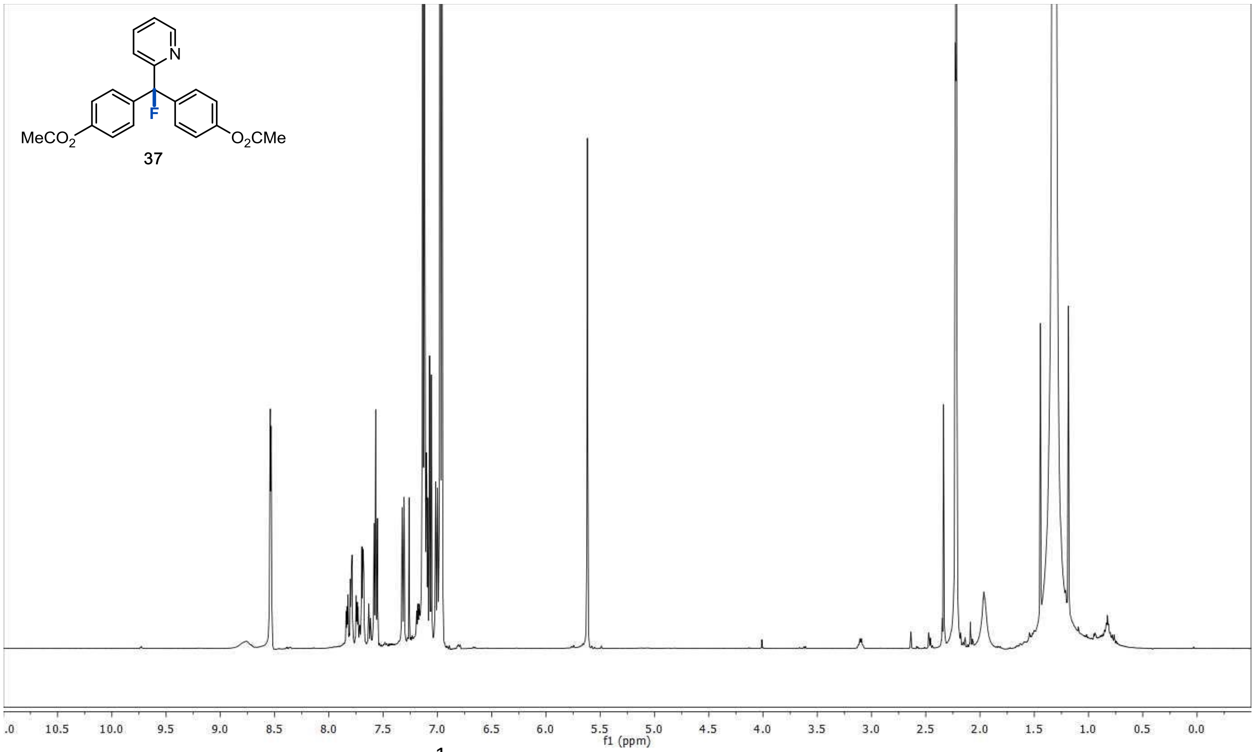

Supplementary Figure 143. ^{1}H NMR (500 MHz, $CDCl_3$) of **37** crude reaction mixture.

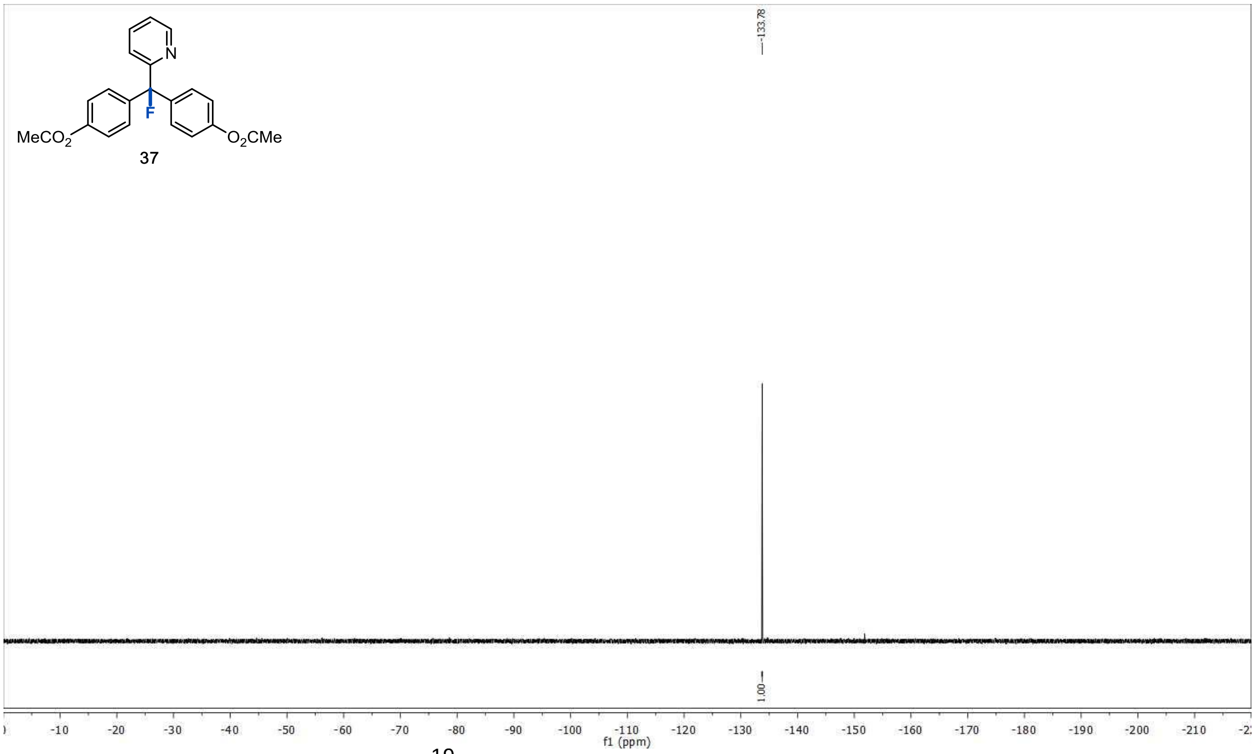

Supplementary Figure 144. ^{19}F NMR (282 MHz, $CDCl_3$) of **37** crude reaction mixture.

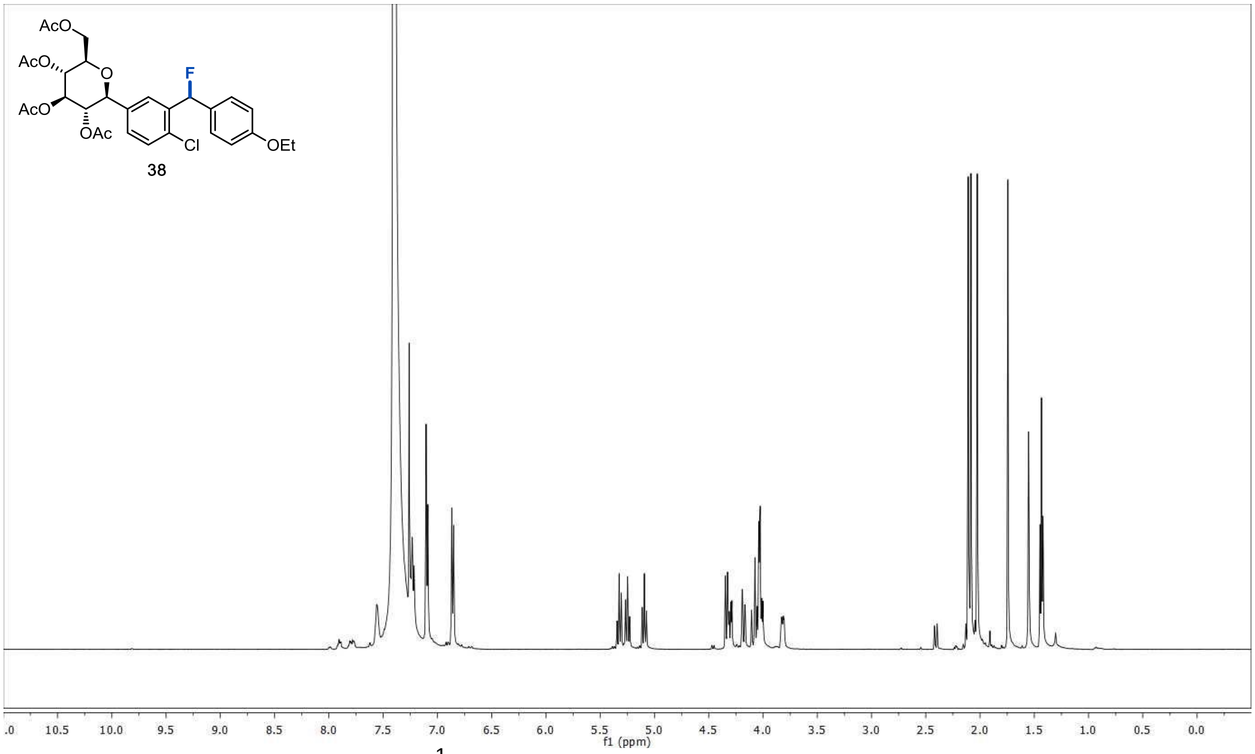

Supplementary Figure 145. ^{1}H NMR (500 MHz, $CDCl_3$) of **38** crude reaction mixture.

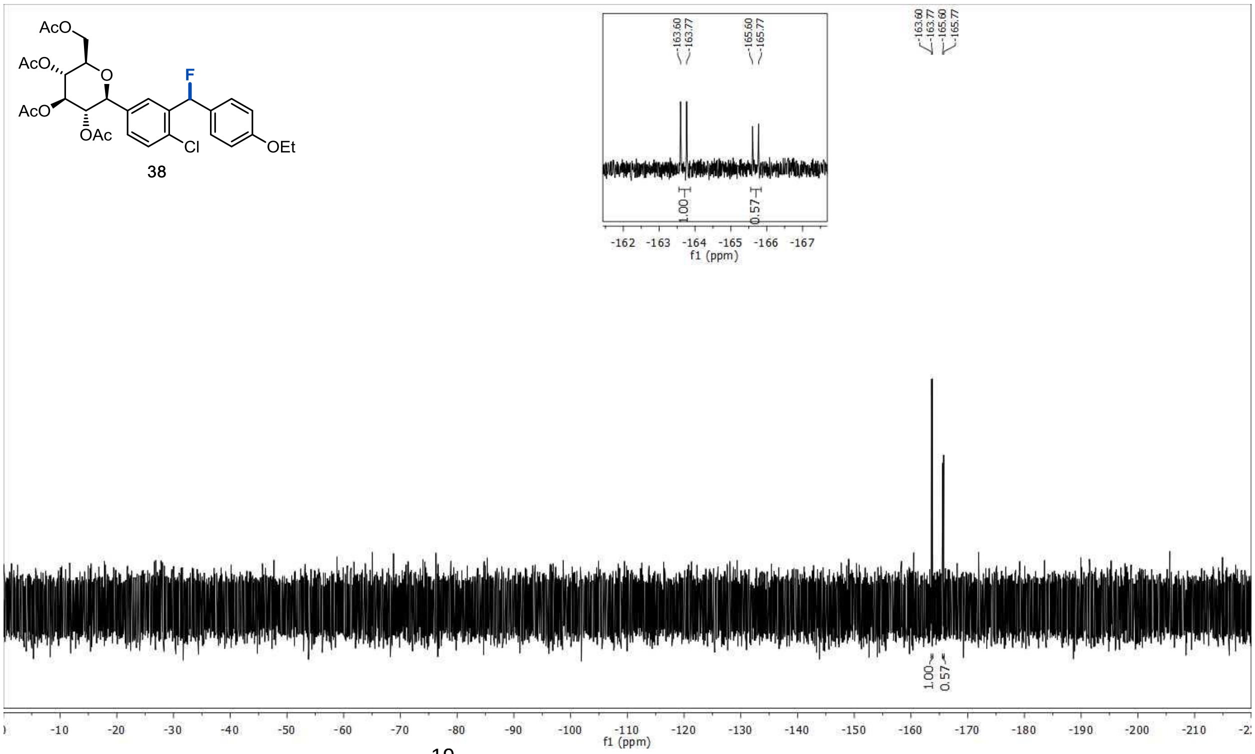

Supplementary Figure 146. ^{19}F NMR (376 MHz, $CDCl_3$) of **38** crude reaction mixture.

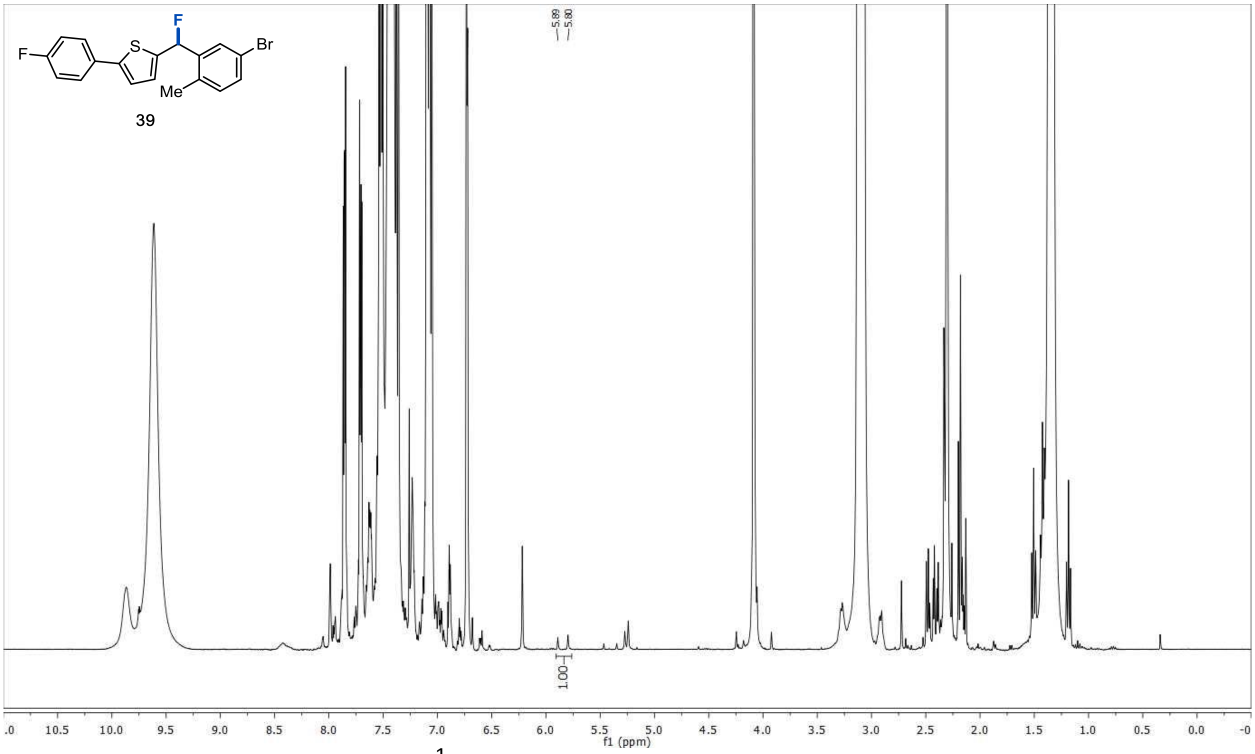

Supplementary Figure 147. ^{1}H NMR (500 MHz, $CDCl_3$) of **39** crude reaction mixture.

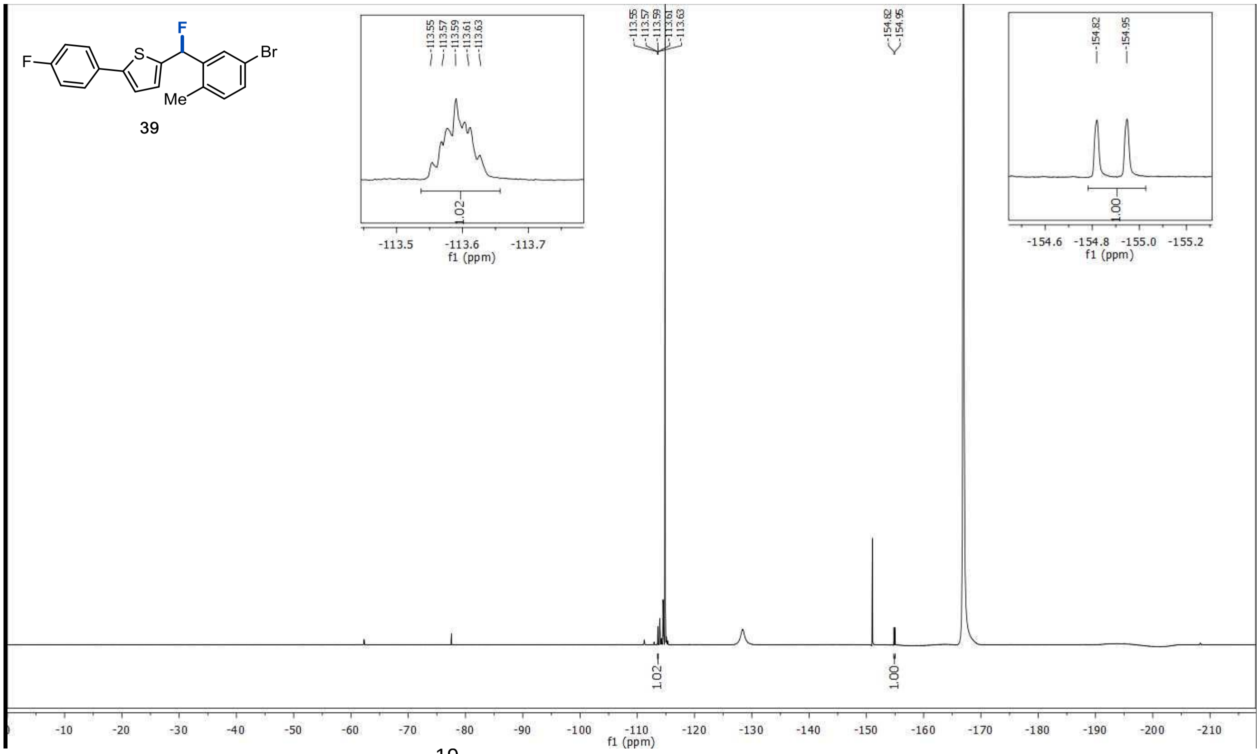

Supplementary Figure 148. ^{19}F NMR (376 MHz, $CDCl_3$) of **39** crude reaction mixture.

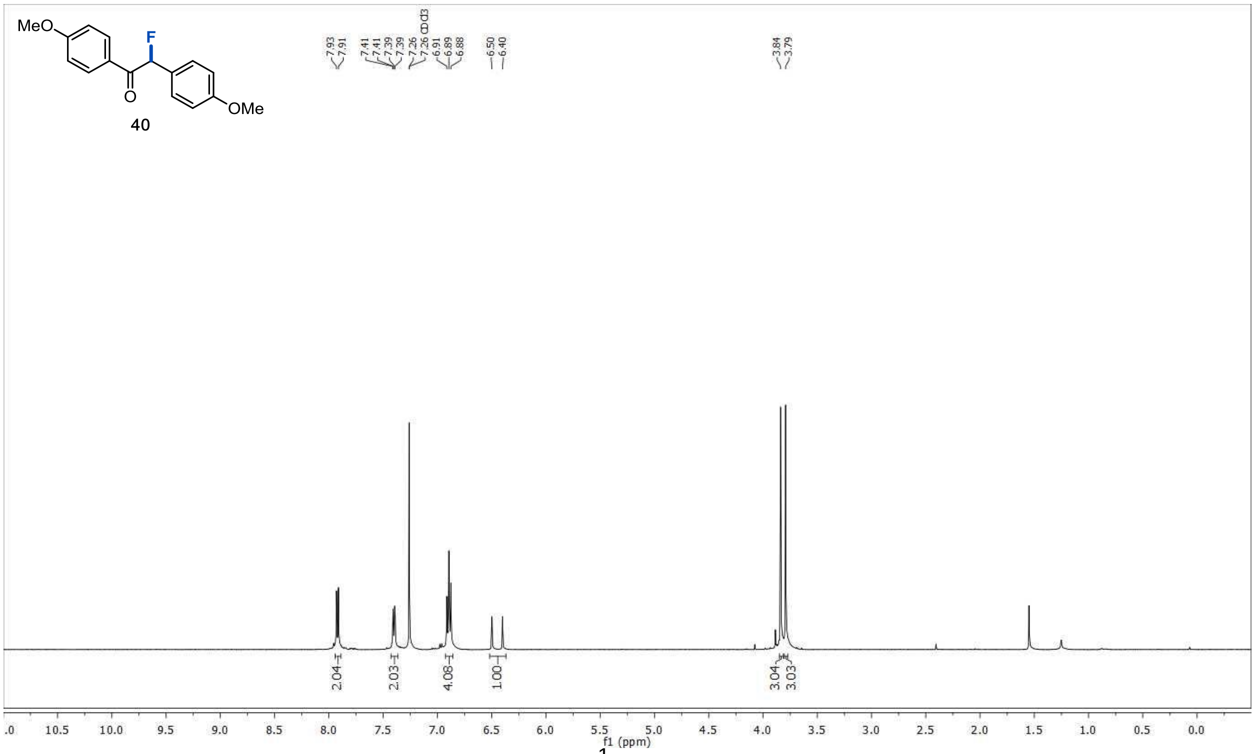

Supplementary Figure 149. ^{1}H NMR (500 MHz, $CDCl_3$) of **40**.

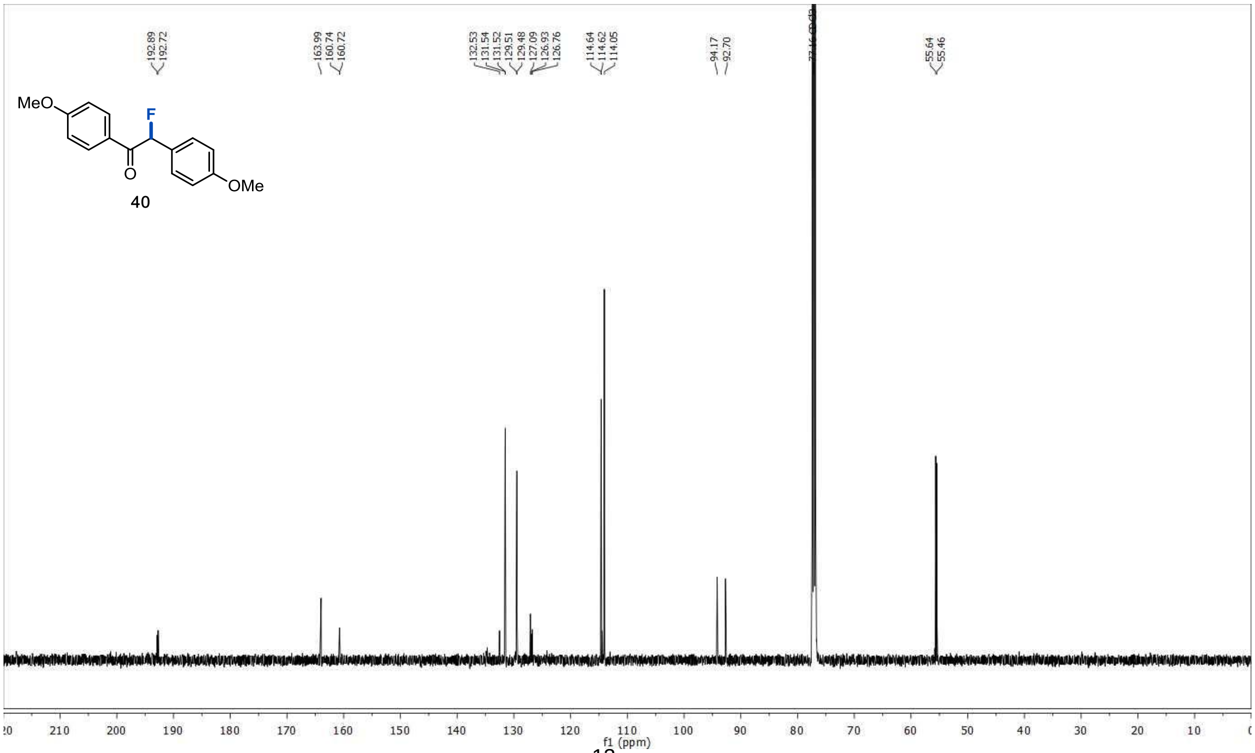

Supplementary Figure 150. ^{13}C NMR (126 MHz, $CDCl_3$) of **40**.

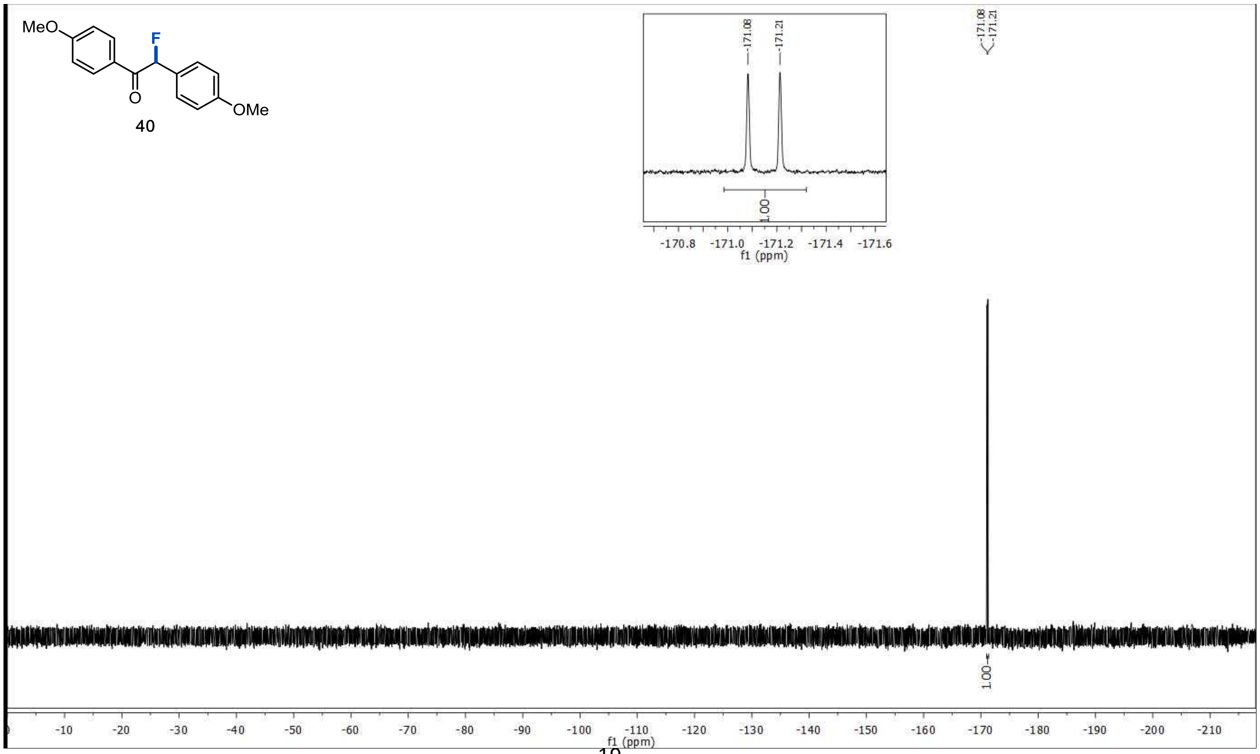

Supplementary Figure 151. ^{19}F NMR (376 MHz, $CDCl_3$) of **40**.

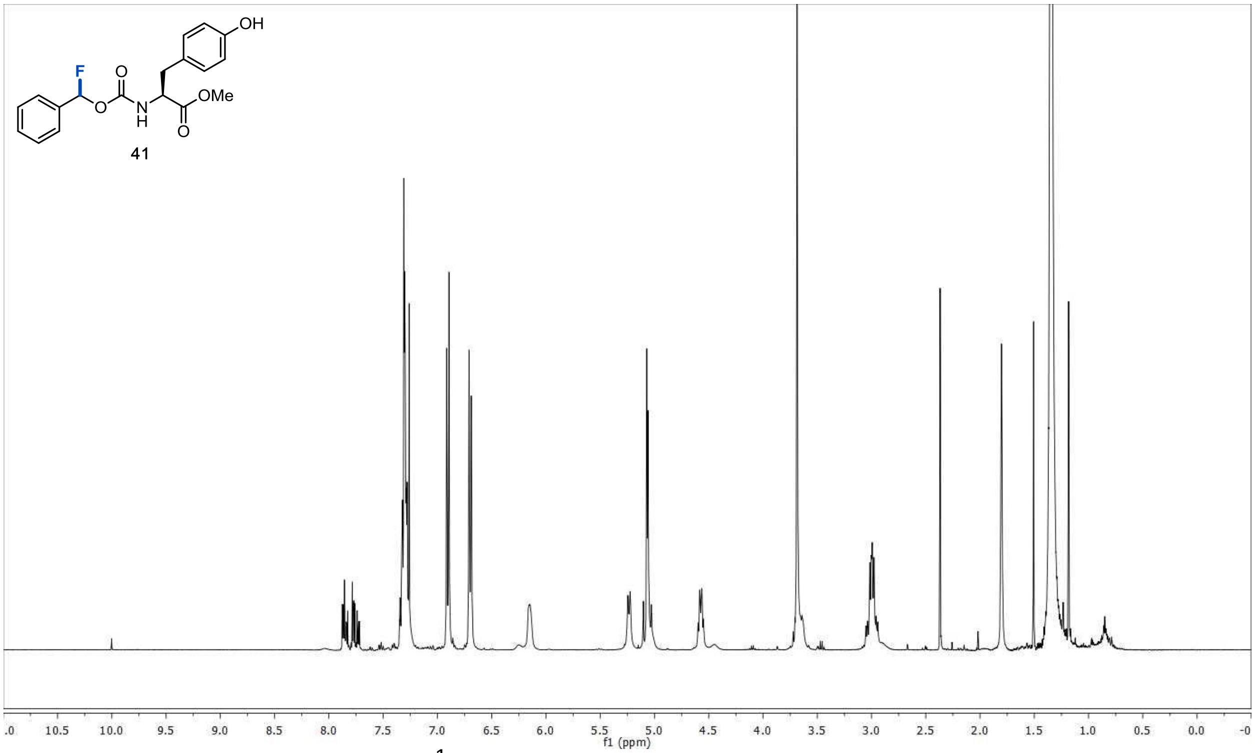

Supplementary Figure 152. ^{1}H NMR (500 MHz, $CDCl_3$) of **41** crude reaction mixture.

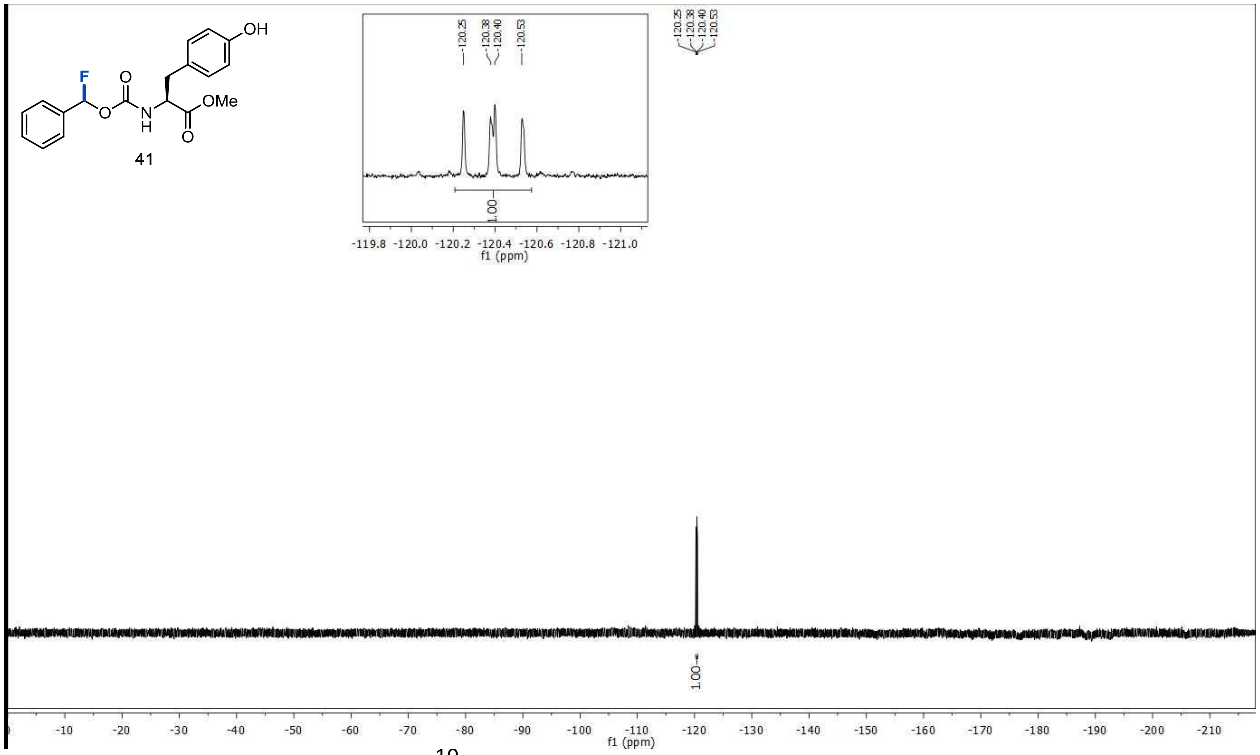

Supplementary Figure 153. ^{19}F NMR (376 MHz, $CDCl_3$) of **41** crude reaction mixture.

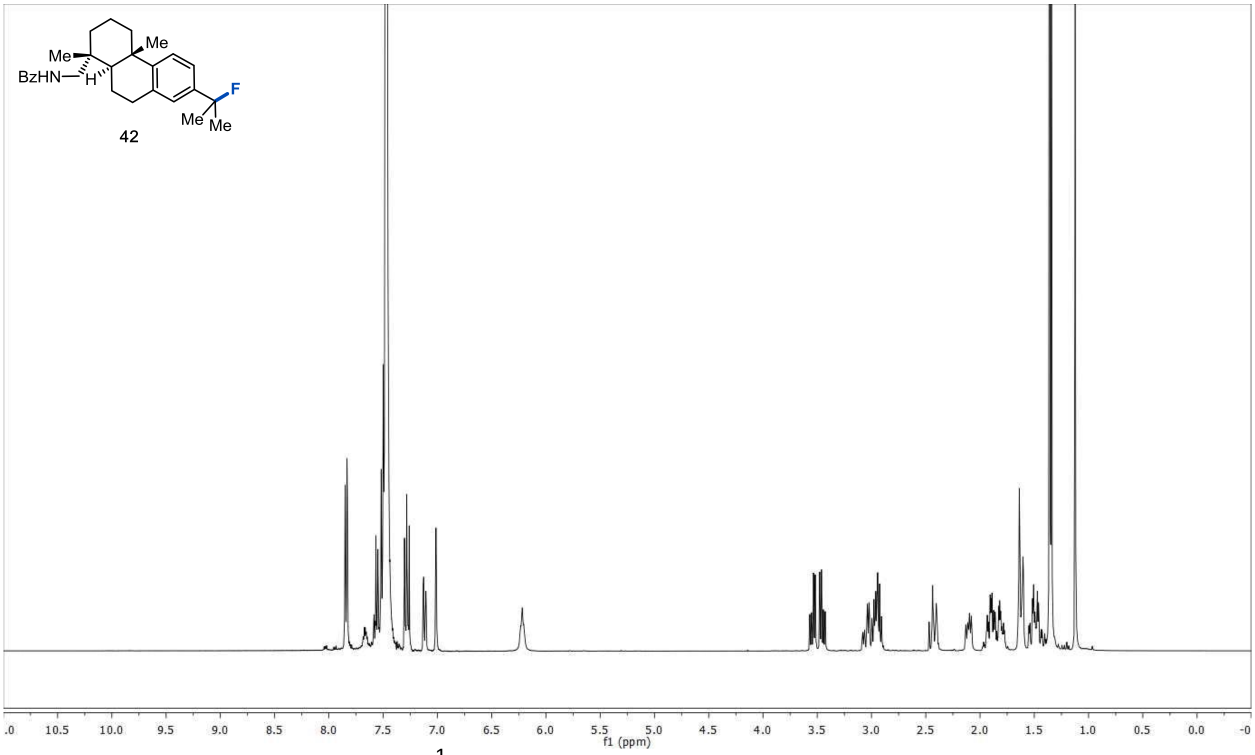

Supplementary Figure 154. ^{1}H NMR (500 MHz, $CDCl_3$) of **42** crude reaction mixture.

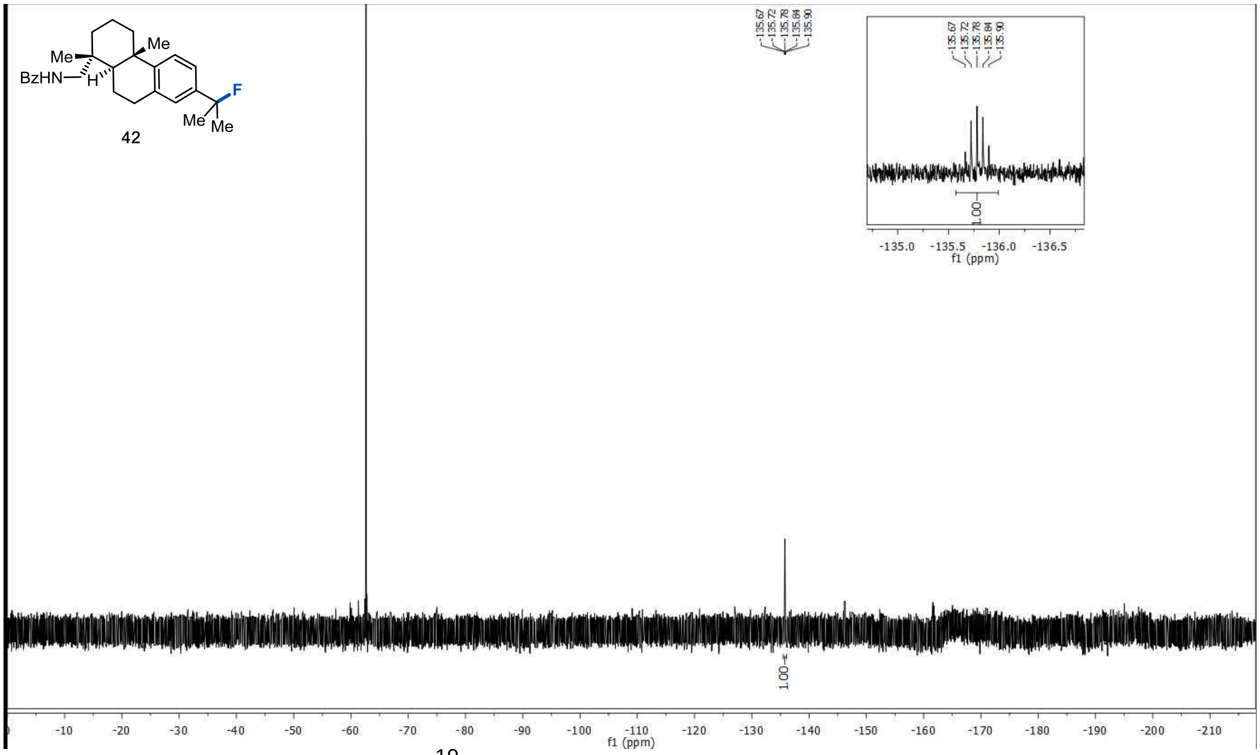

Supplementary Figure 155. ^{19}F NMR (376 MHz, $CDCl_3$) of **42** crude reaction mixture.

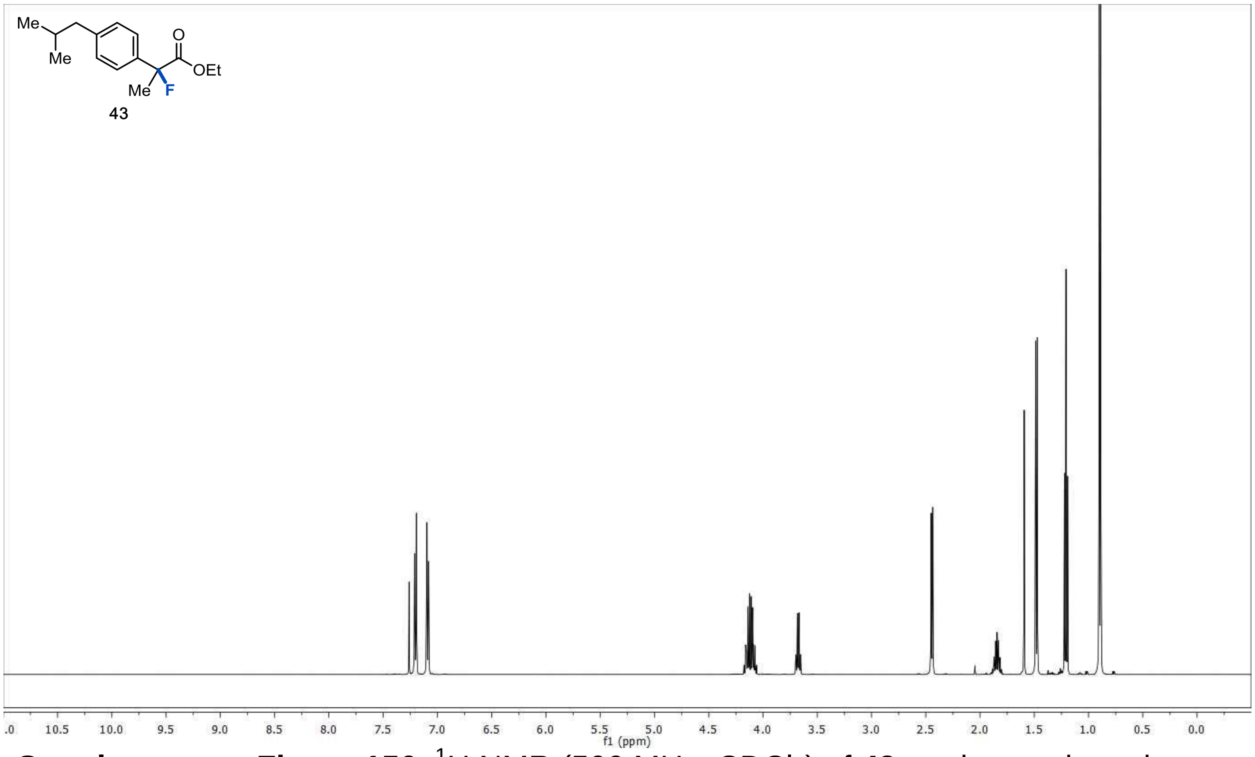

Supplementary Figure 156. ^{1}H NMR (500 MHz, $CDCl_3$) of **43** crude reaction mixture.

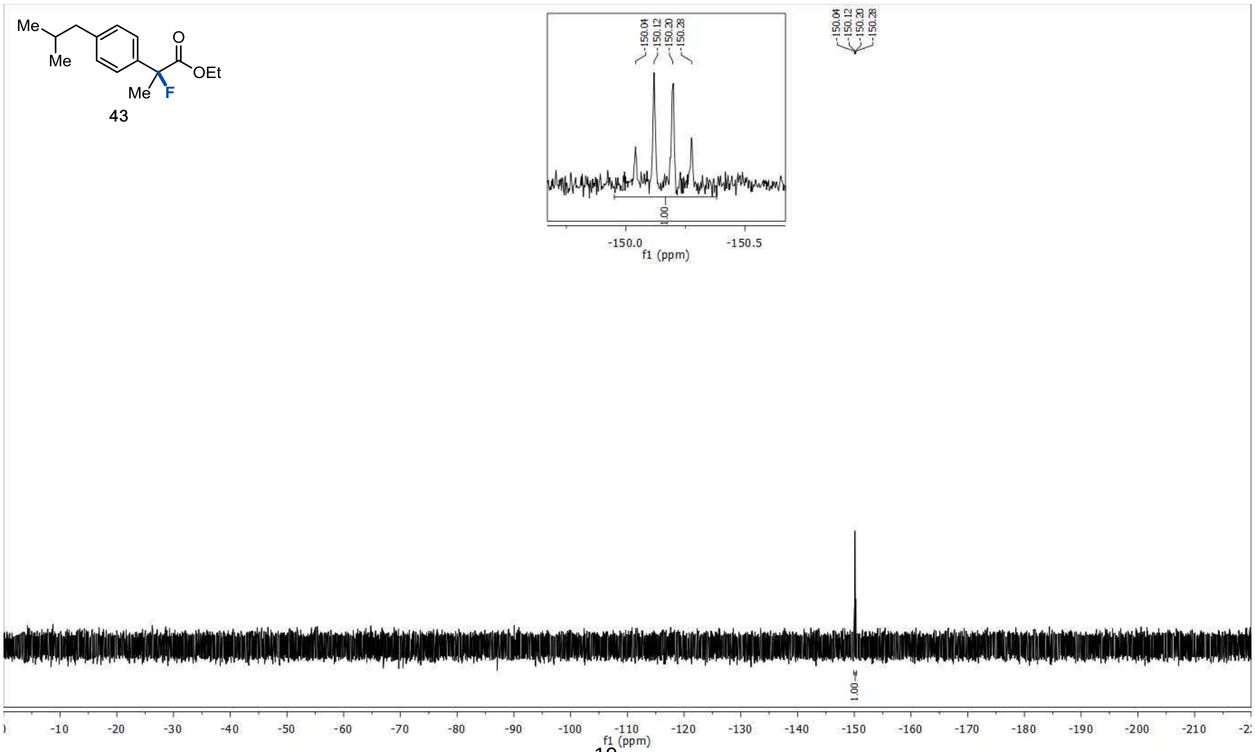

Supplementary Figure 157. ^{19}F NMR (282 MHz, $CDCl_3$) of **43**.

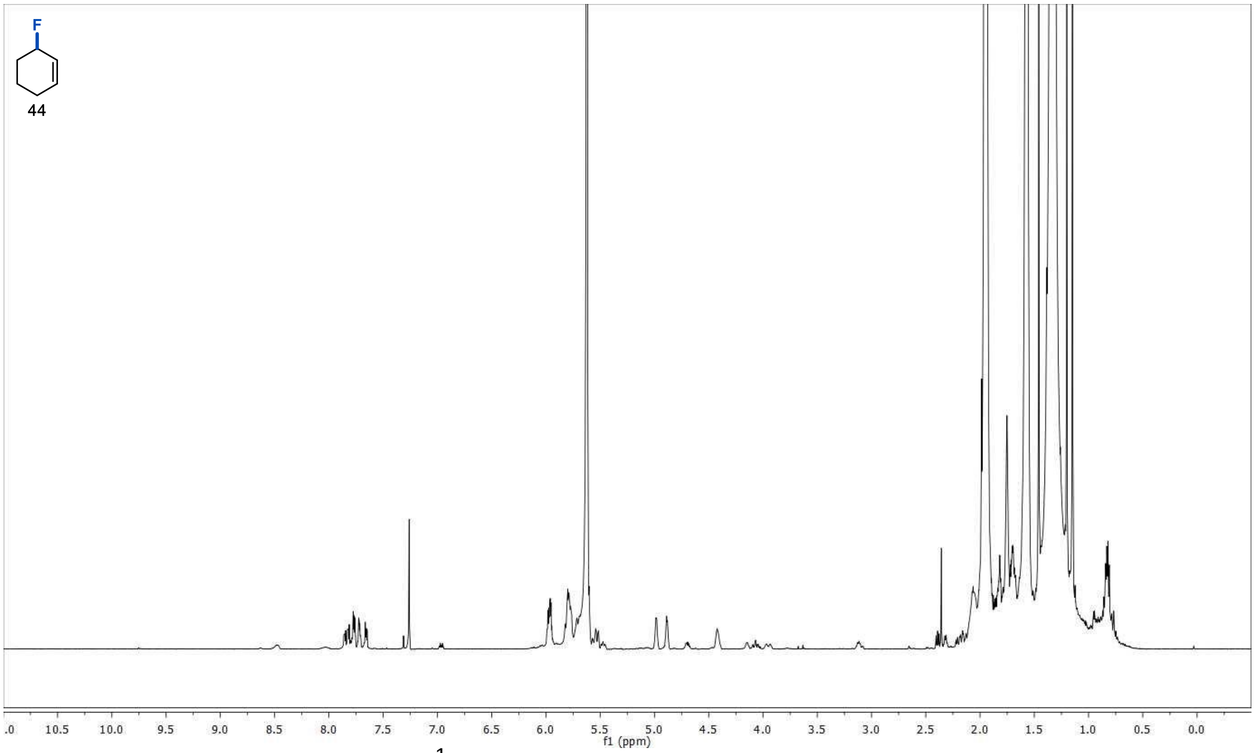

Supplementary Figure 158. ^{1}H NMR (500 MHz, $CDCl_3$) of **44** crude reaction mixture.

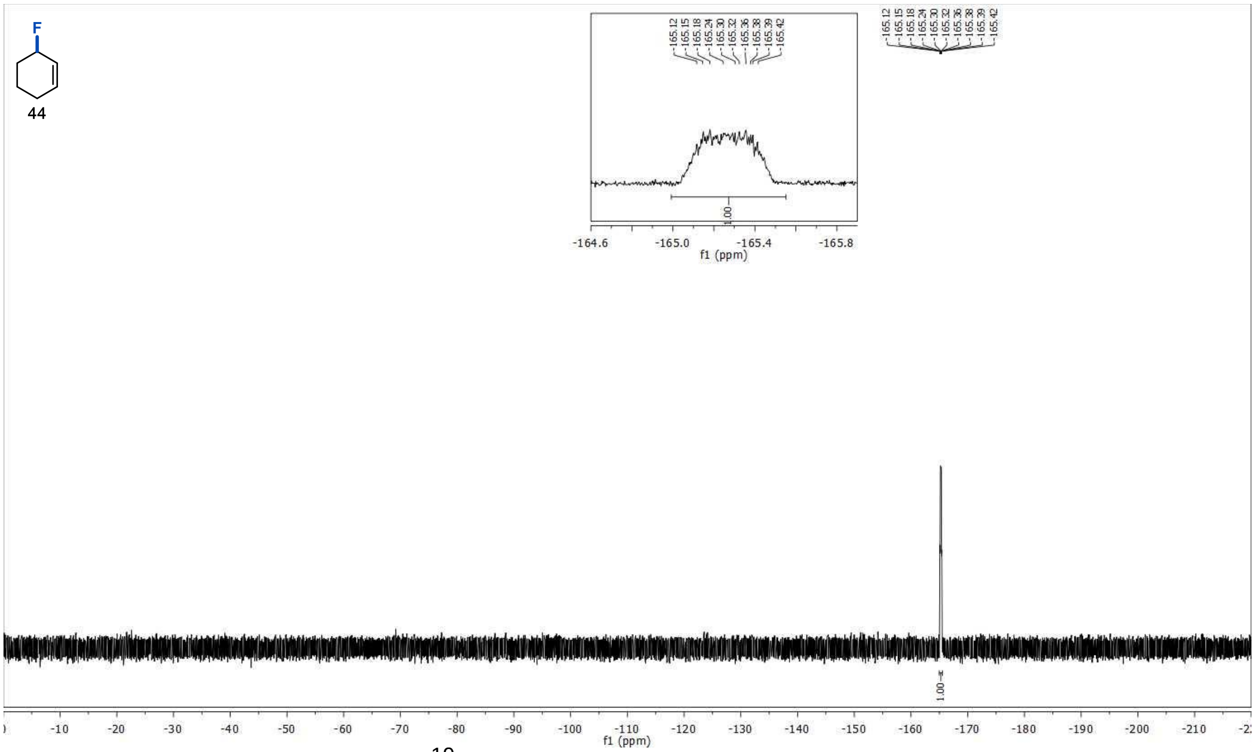

Supplementary Figure 159. ^{19}F NMR (282 MHz, $CDCl_3$) of **44** crude reaction mixture.

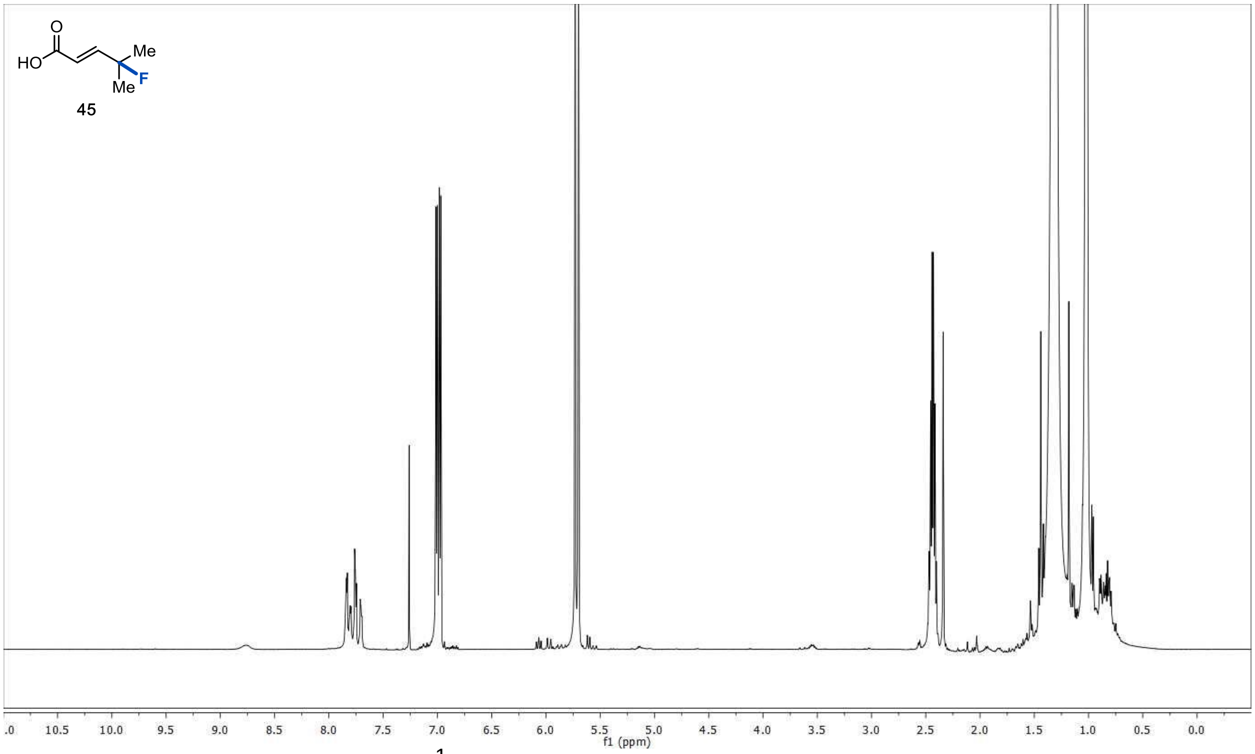

Supplementary Figure 160. ^{1}H NMR (500 MHz, $CDCl_3$) of **45** crude reaction mixture.

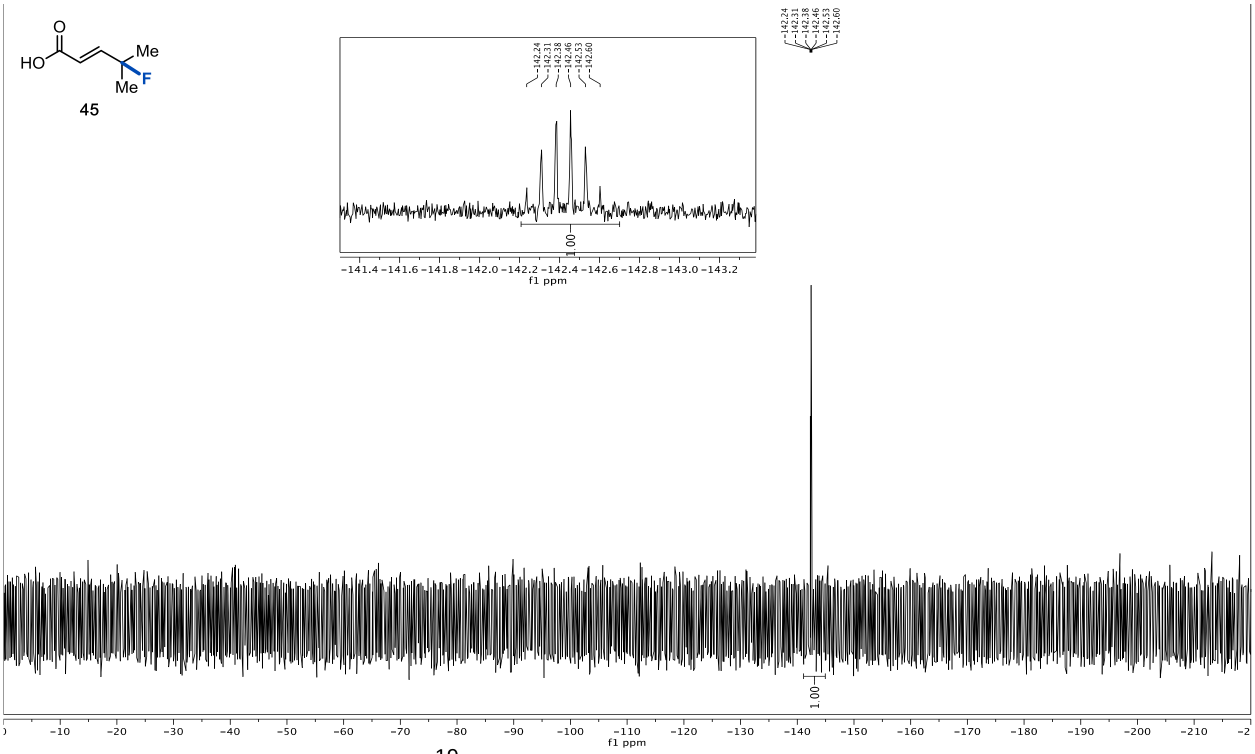

Supplementary Figure 161. ^{19}F NMR (282 MHz, $CDCl_3$) of **45** crude reaction mixture.

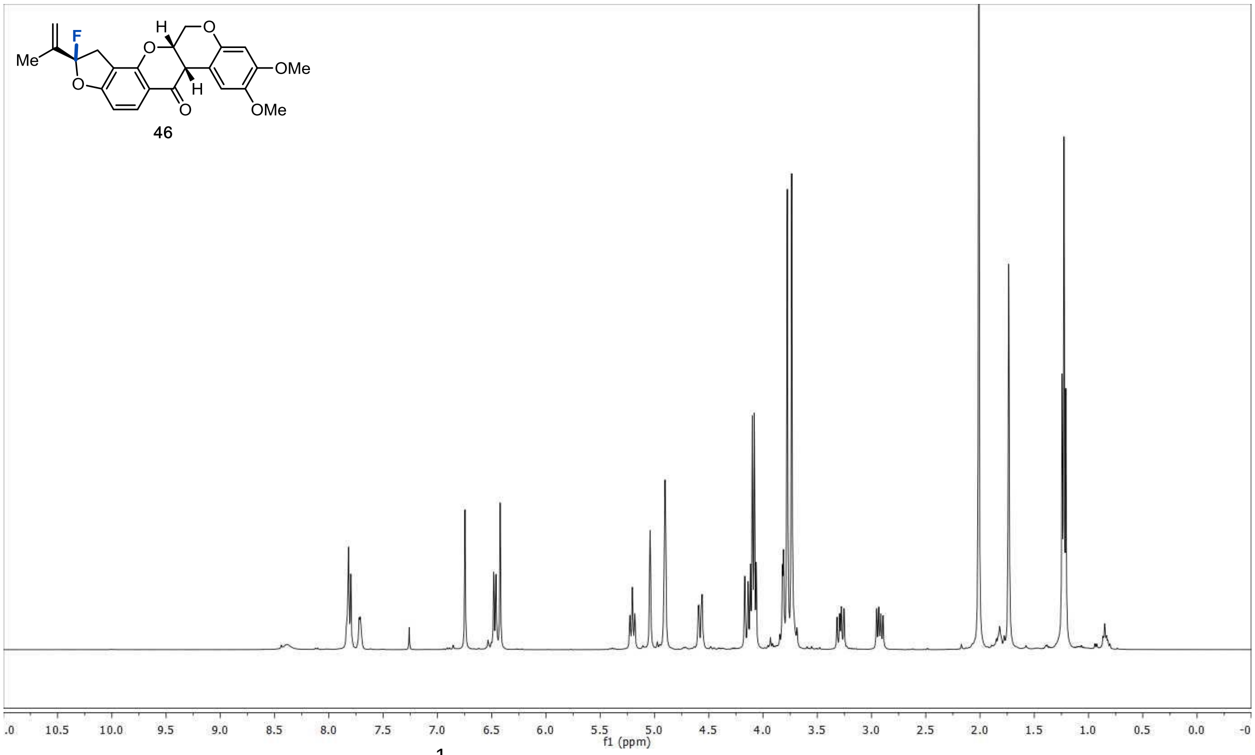

Supplementary Figure 162. ^{1}H NMR (500 MHz, $CDCl_3$) of **46** crude reaction mixture.

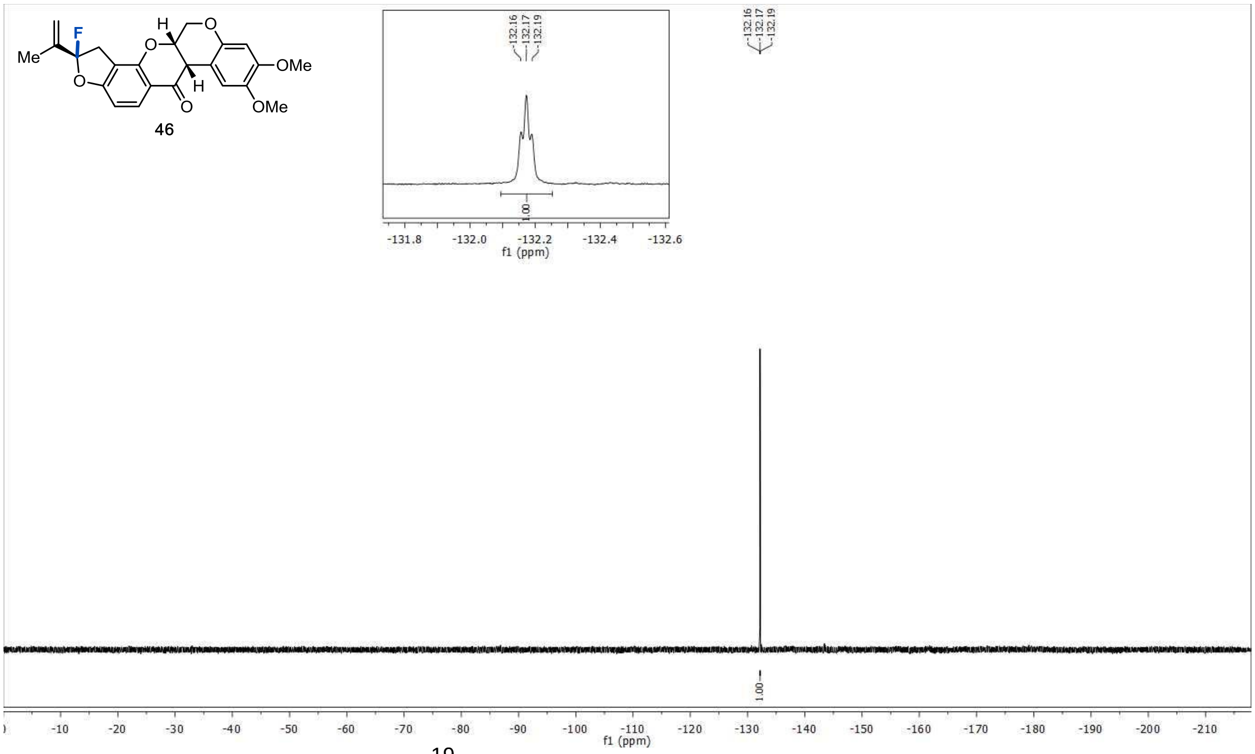

Supplementary Figure 163. ^{19}F NMR (282 MHz, $CDCl_3$) of **46** crude reaction mixture.

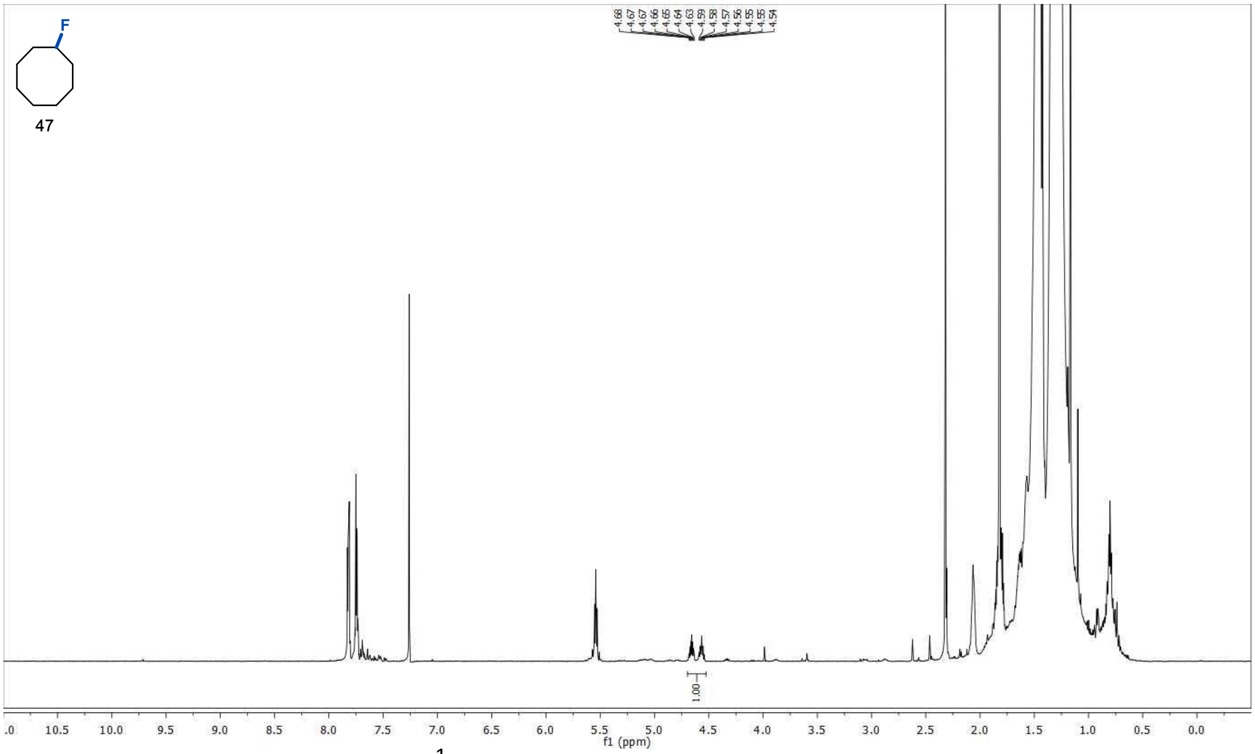

Supplementary Figure 164. ^{1}H NMR (500 MHz, $CDCl_3$) of **47** crude reaction mixture.

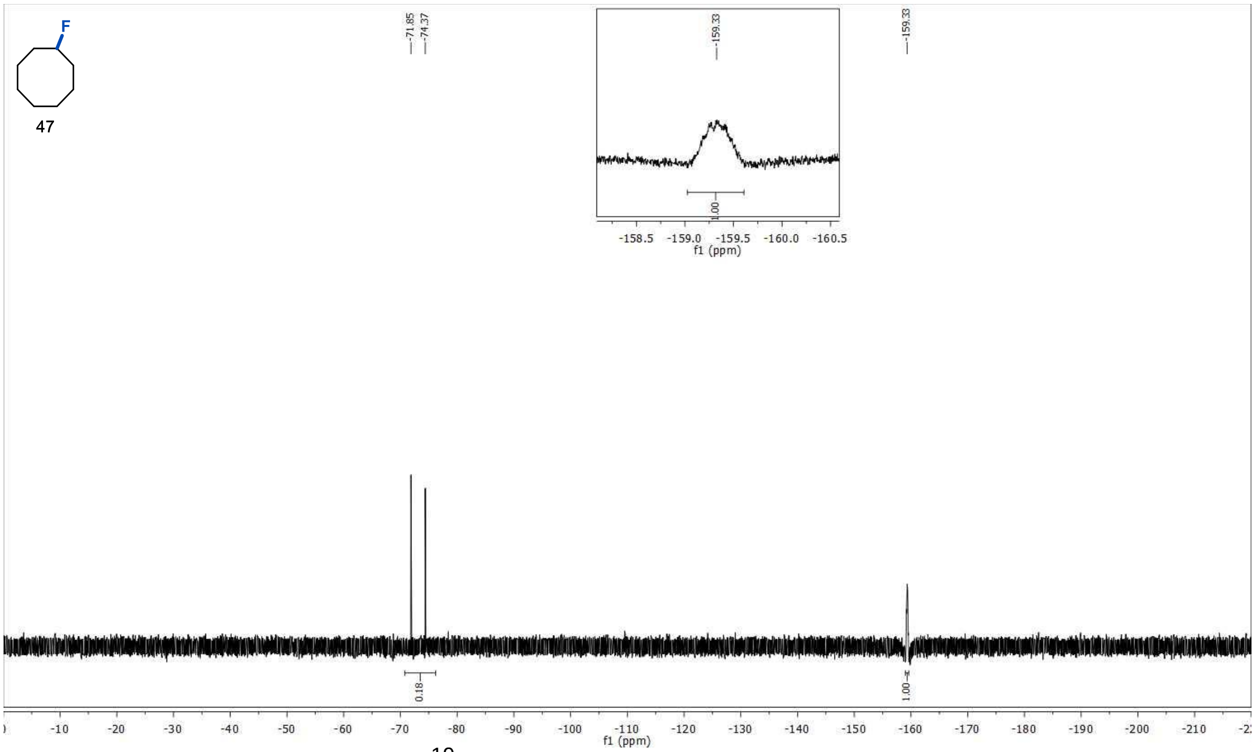

Supplementary Figure 165. ^{19}F NMR (282 MHz, $CDCl_3$) of **47** crude reaction mixture.

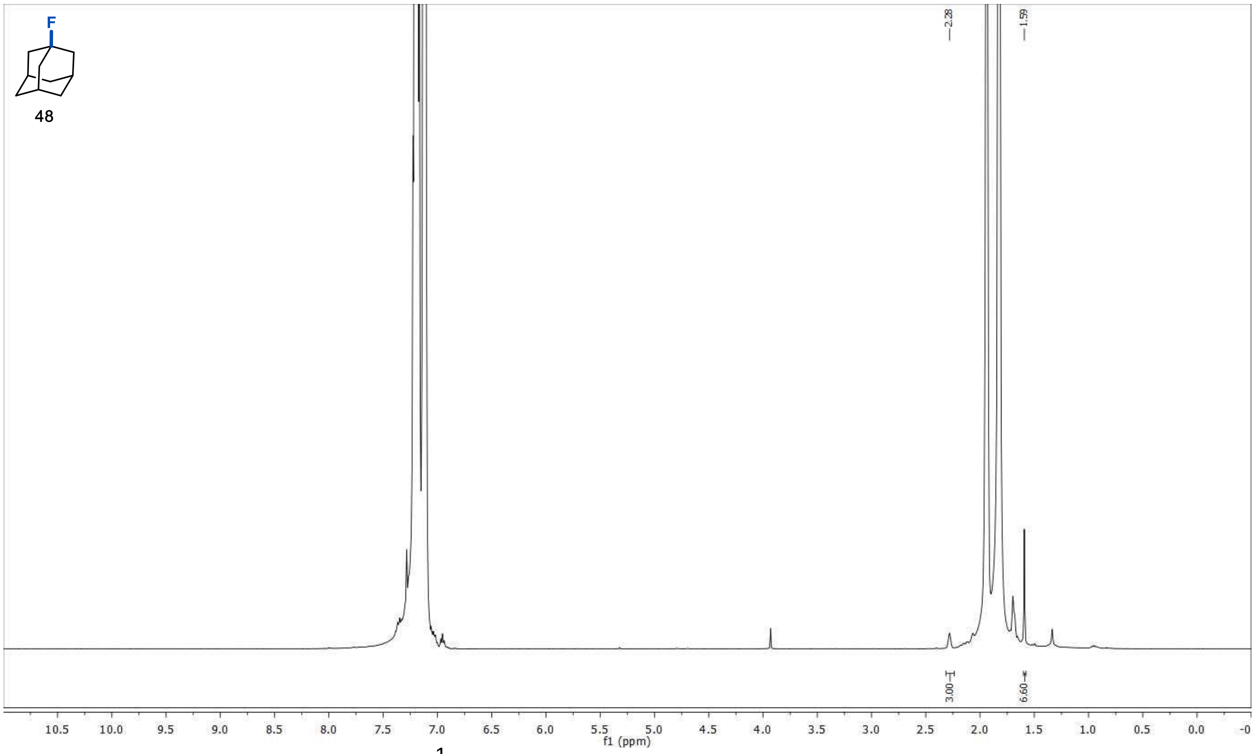

Supplementary Figure 166. ^{1}H NMR (500 MHz, $CDCl_3$) of **48** crude reaction mixture.

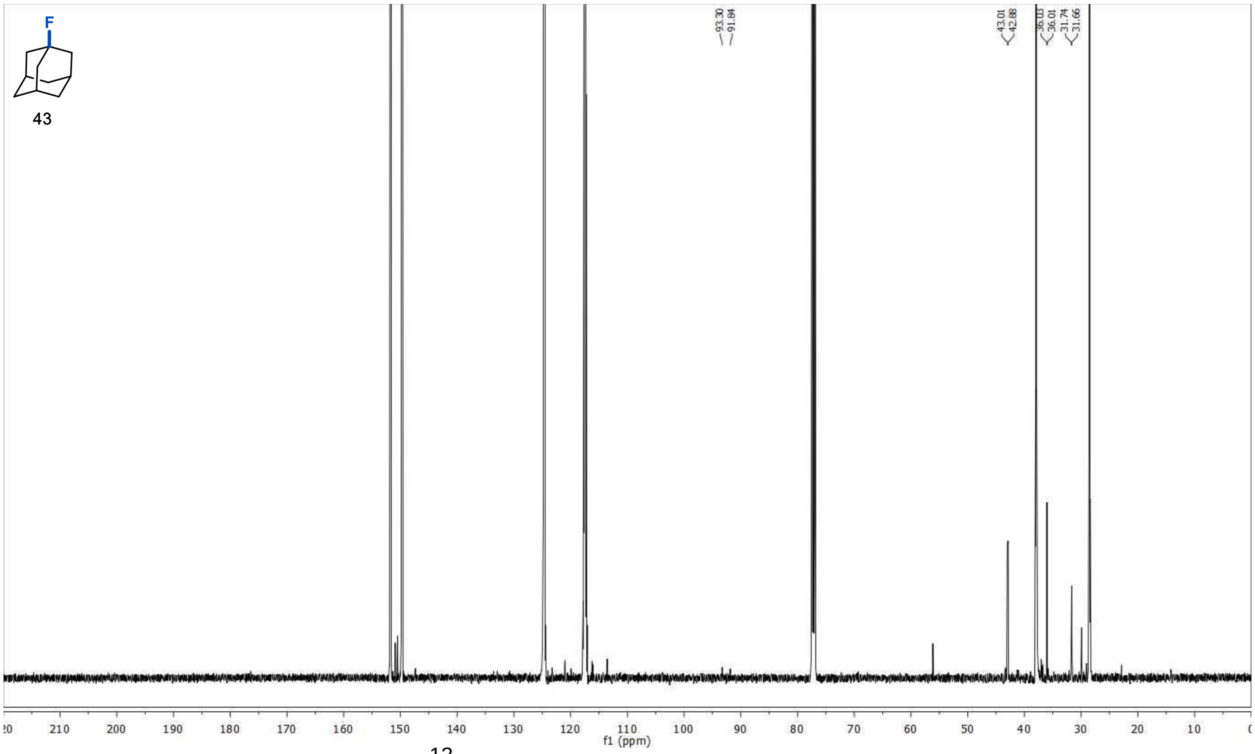

Supplementary Figure 167. ^{13}C NMR (126 MHz, $CDCl_3$) of **43** crude reaction mixture.

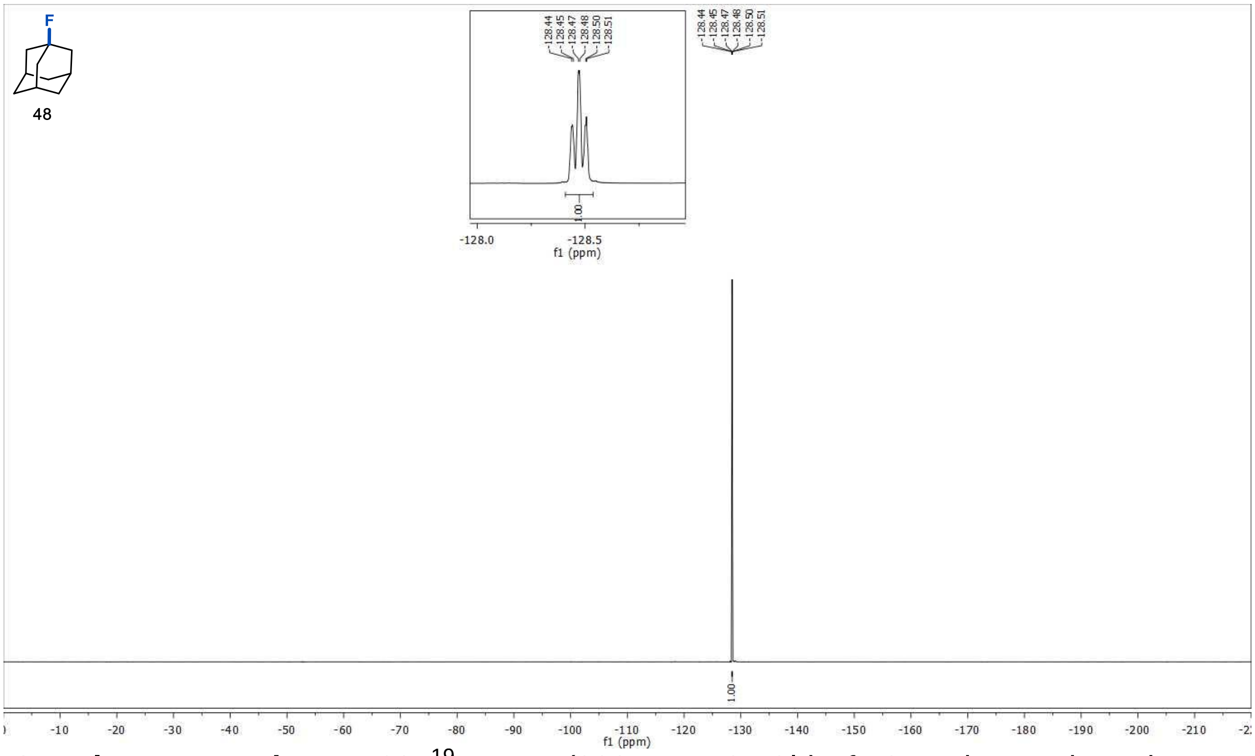

Supplementary Figure 168. ^{19}F NMR (282 MHz, $CDCl_3$) of **48** crude reaction mixture.

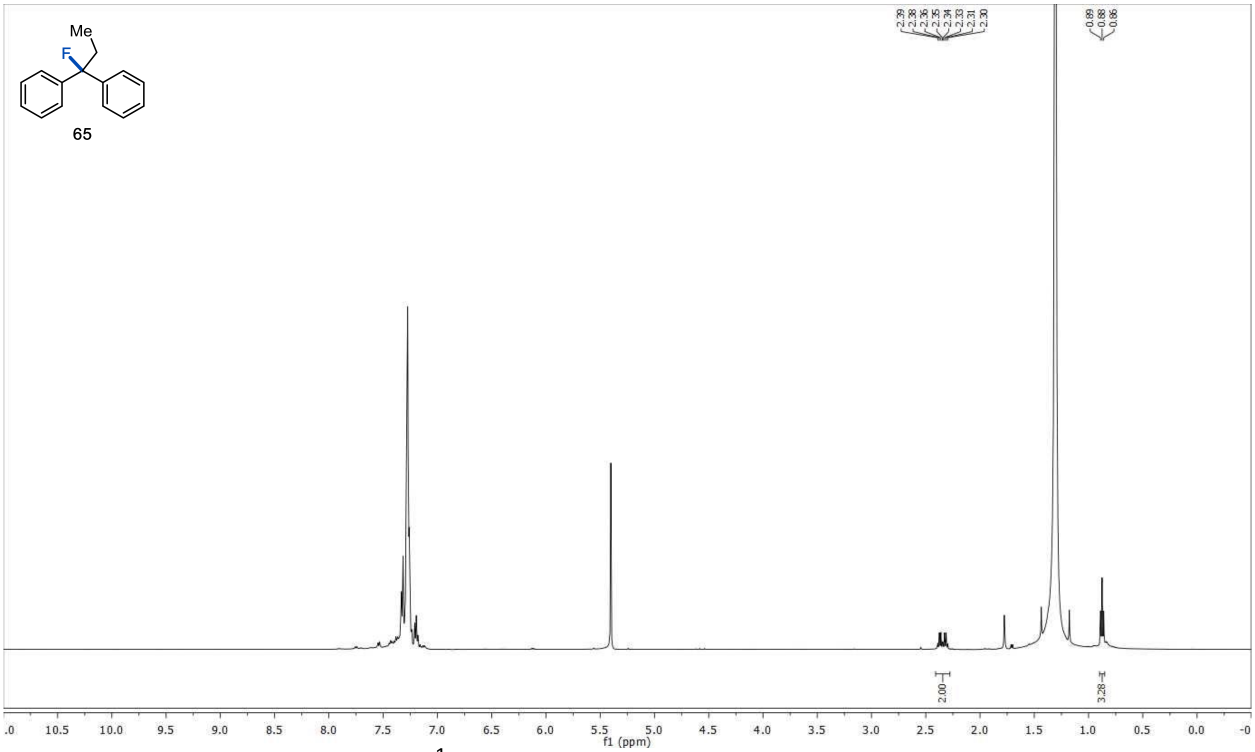

Supplementary Figure 169. ^{1}H NMR (500 MHz, $CDCl_3$) of **65** crude reaction mixture.

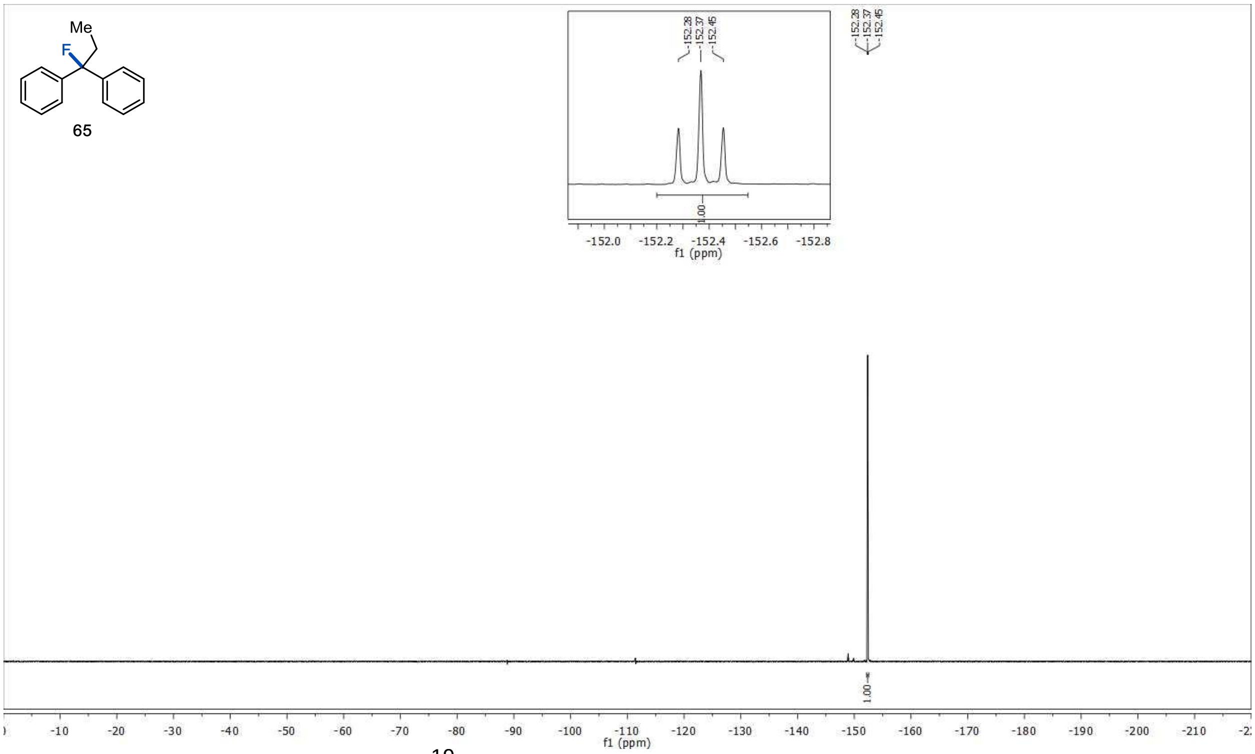

Supplementary Figure 170. ^{19}F NMR (282 MHz, $CDCl_3$) of **65** crude reaction mixture.

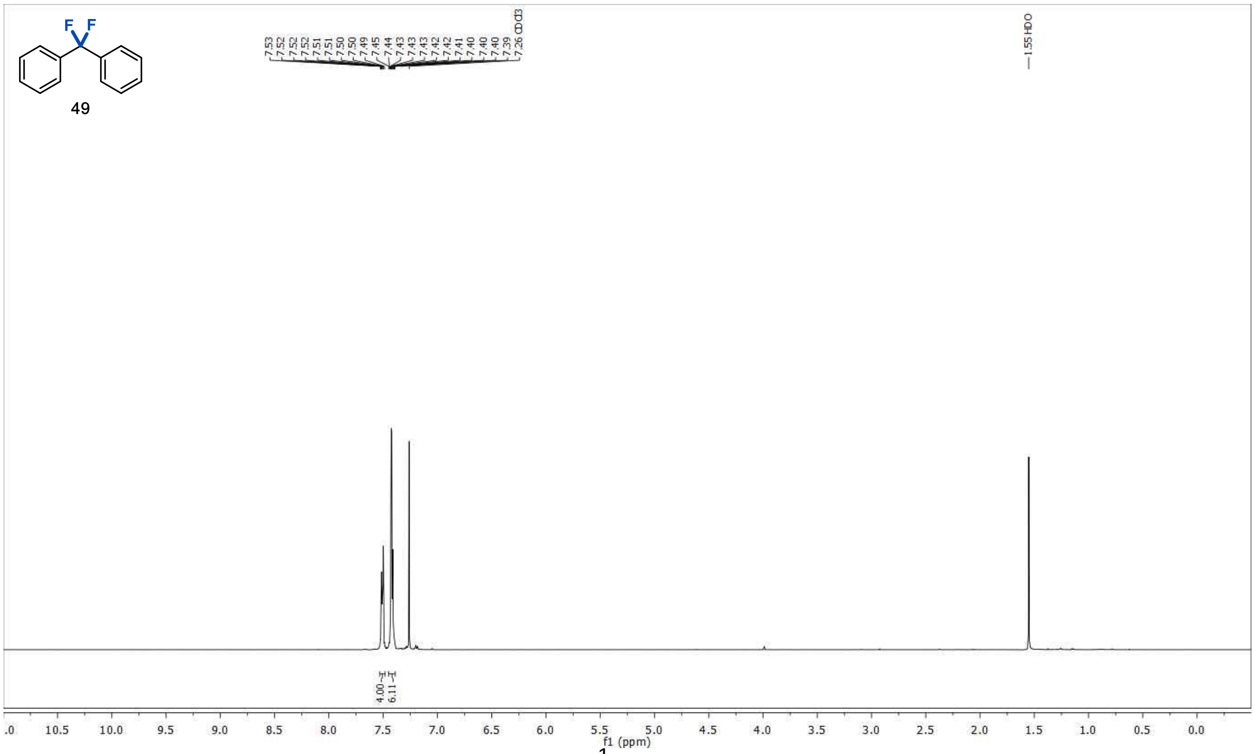

Supplementary Figure 171. ^{1}H NMR (500 MHz, $CDCl_3$) of **49**.

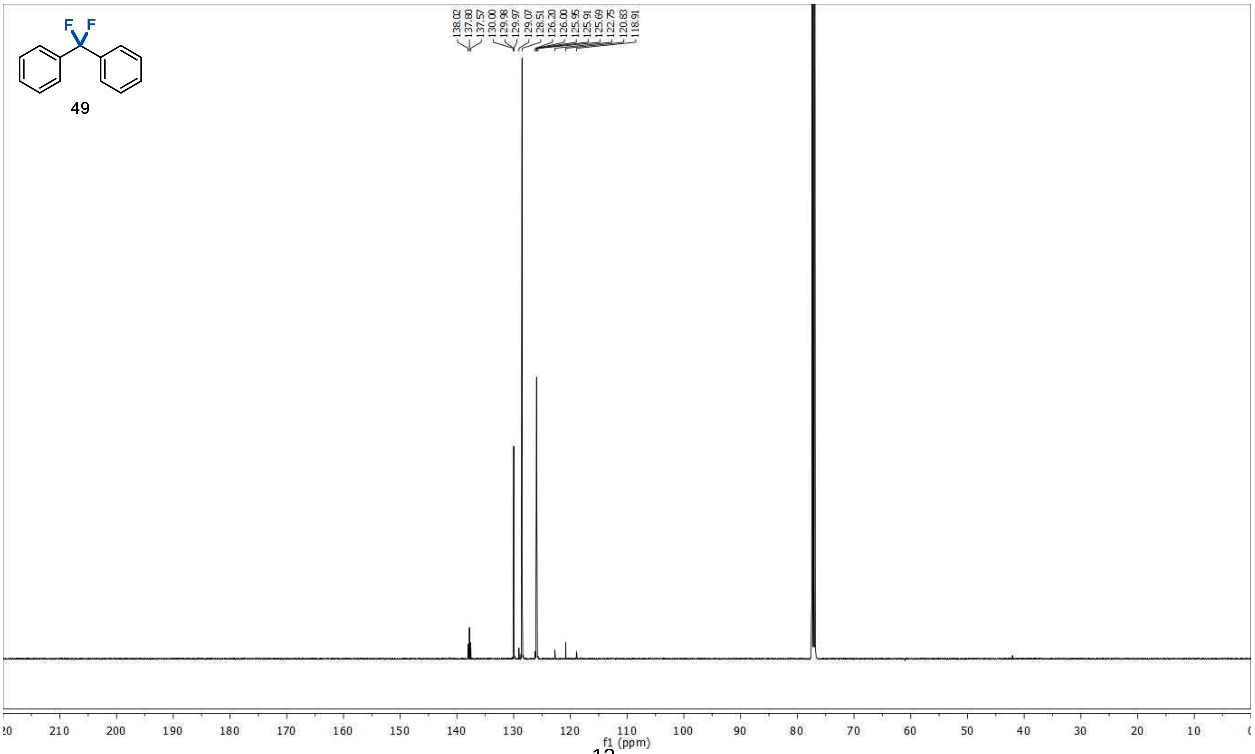

Supplementary Figure 172. ^{13}C NMR (126 MHz, $CDCl_3$) of **49**.

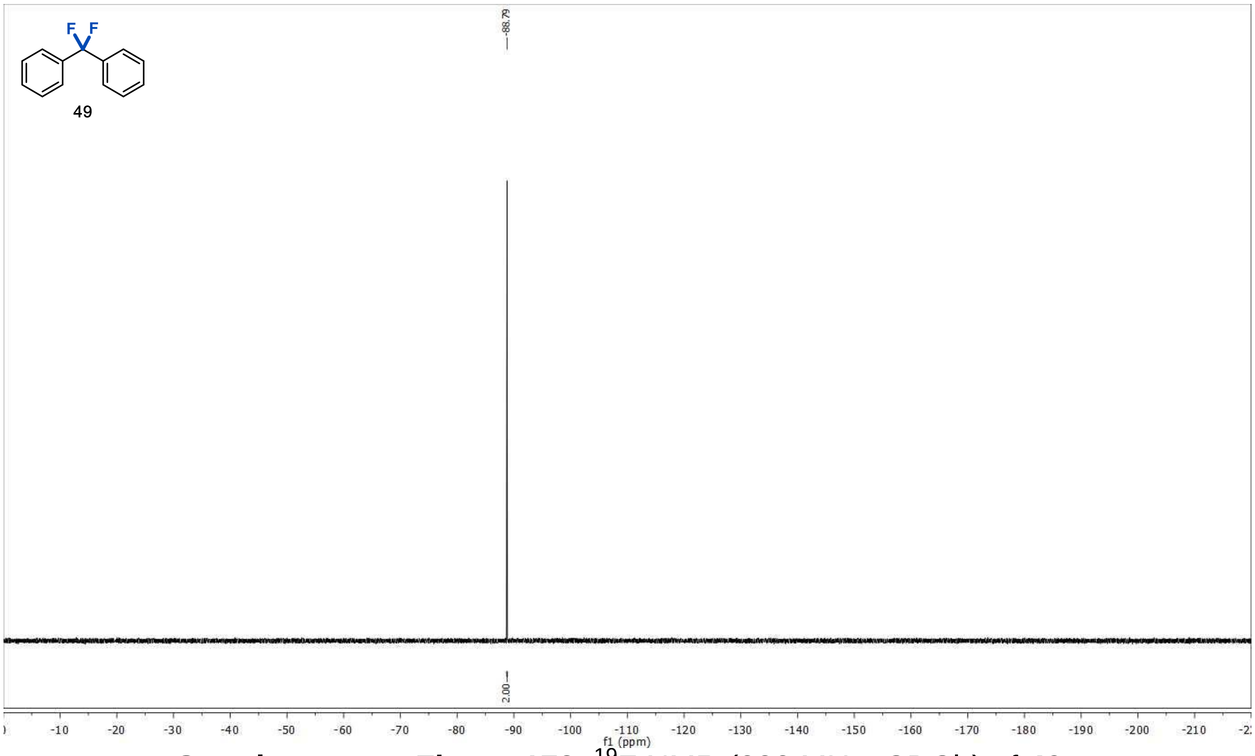

Supplementary Figure 173. ^{19}F NMR (282 MHz, $CDCl_3$) of **49**.

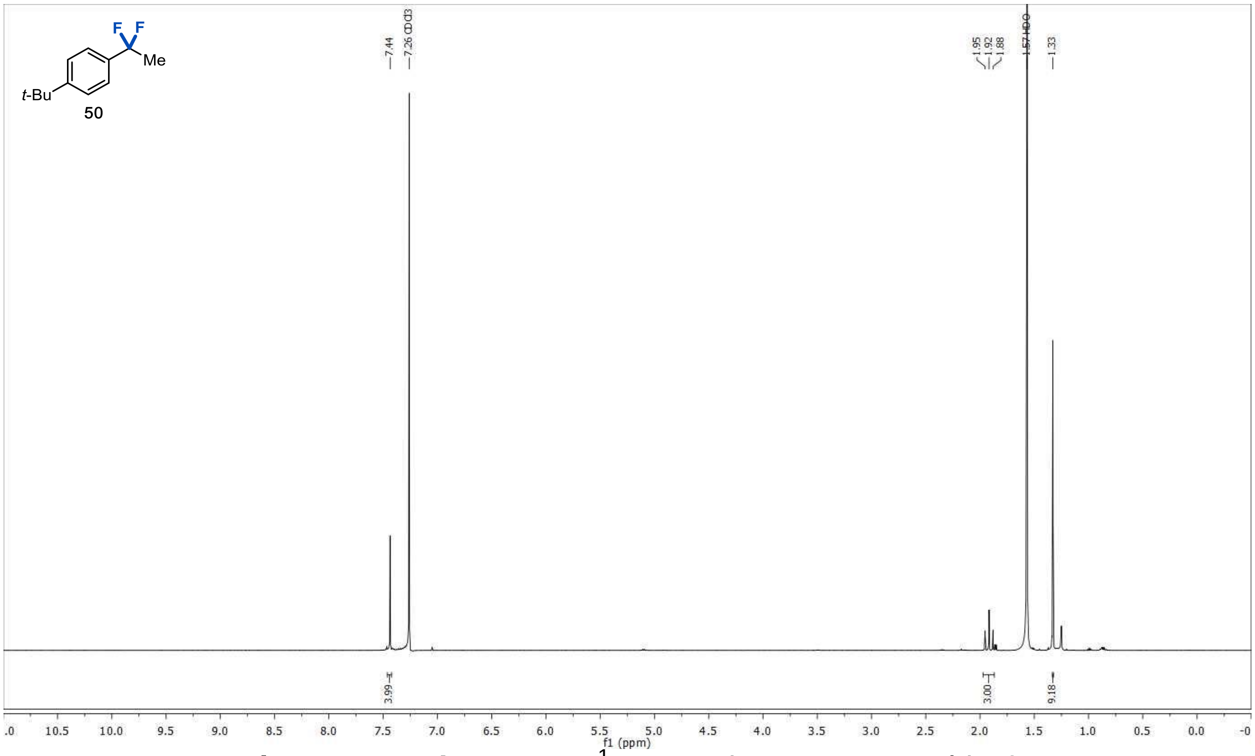

Supplementary Figure 174. ^{1}H NMR (500 MHz, $CDCl_3$) of **50**.

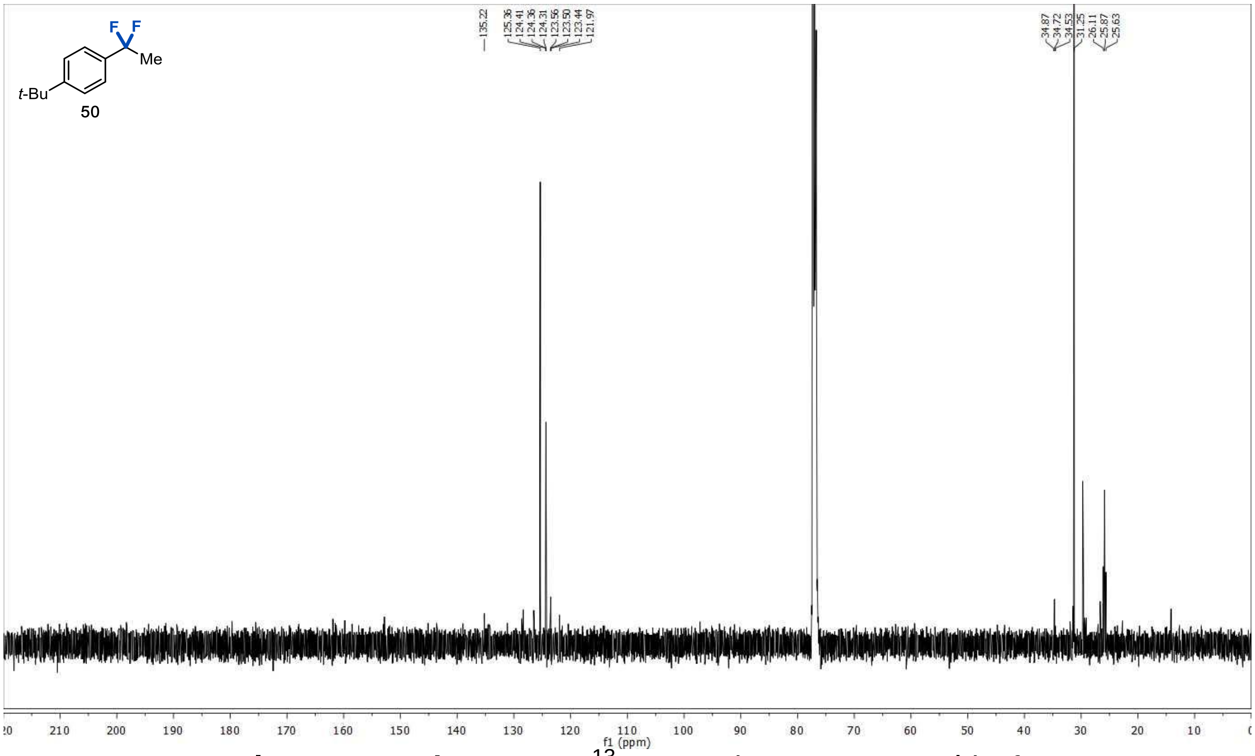

Supplementary Figure 175. ^{13}C NMR (126 MHz, $CDCl_3$) of **50**.

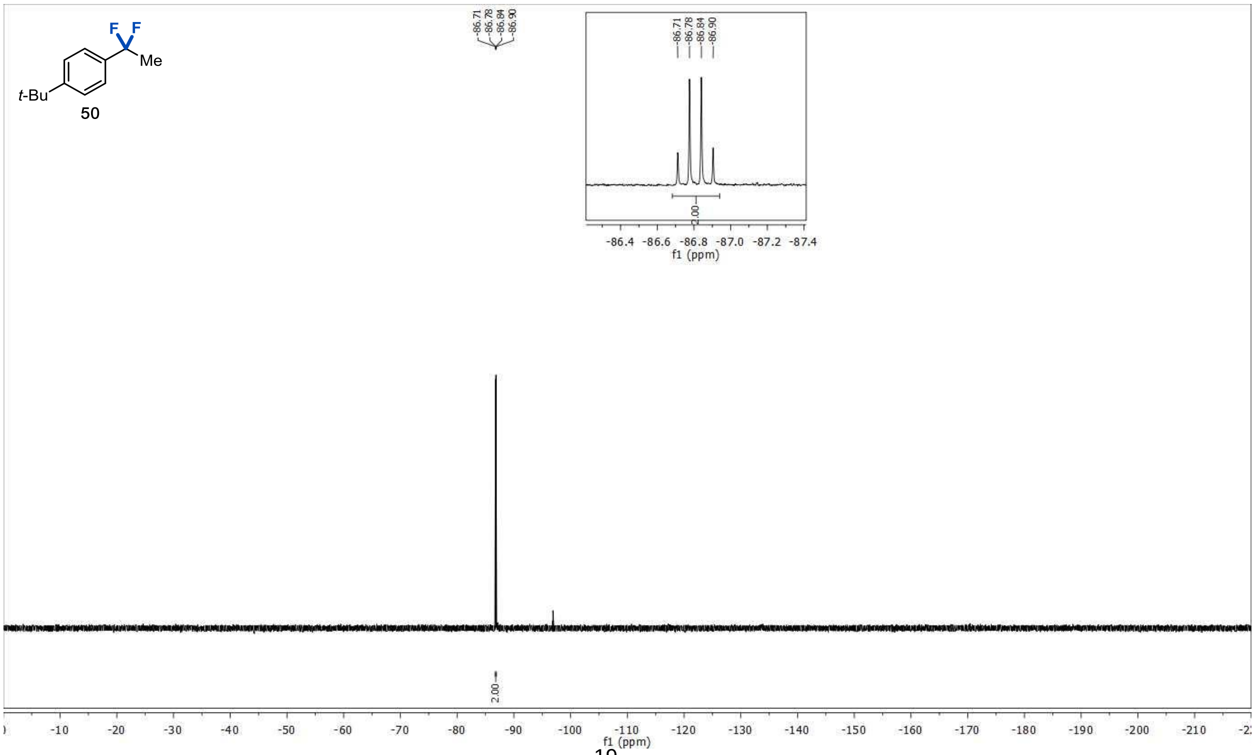

Supplementary Figure 176. ^{19}F NMR (282 MHz, $CDCl_3$) of **50**.

Spectra of Nucleophile Addition Products

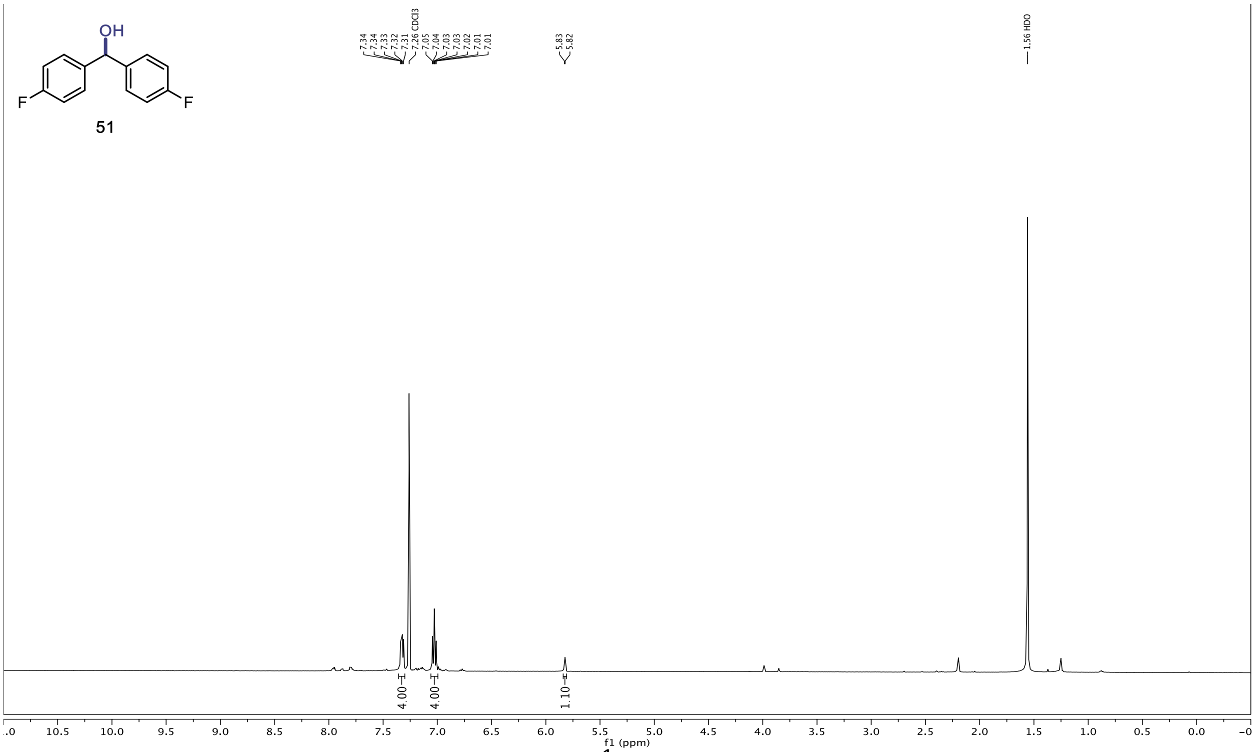

Supplementary Figure 177. ^{1}H NMR (500 MHz, $CDCl_3$) of **51**.

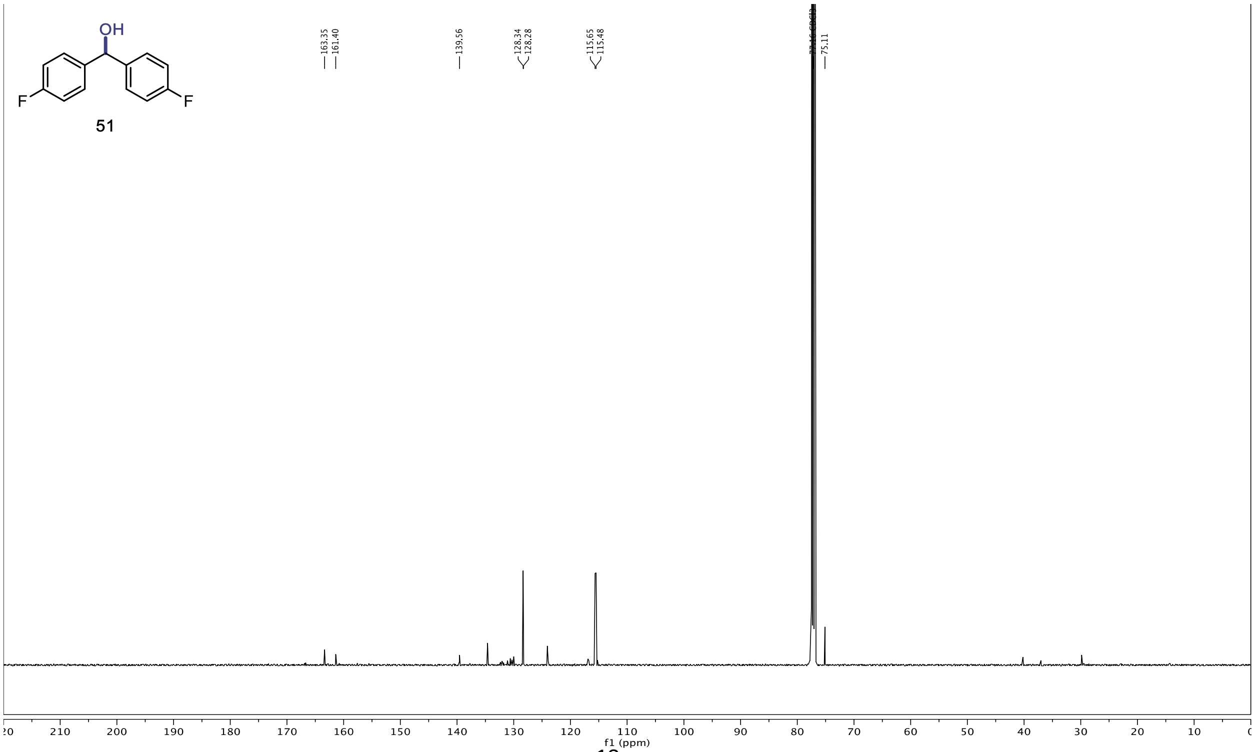

Supplementary Figure 178. ^{13}C NMR (126 MHz, $CDCl_3$) of **51**.

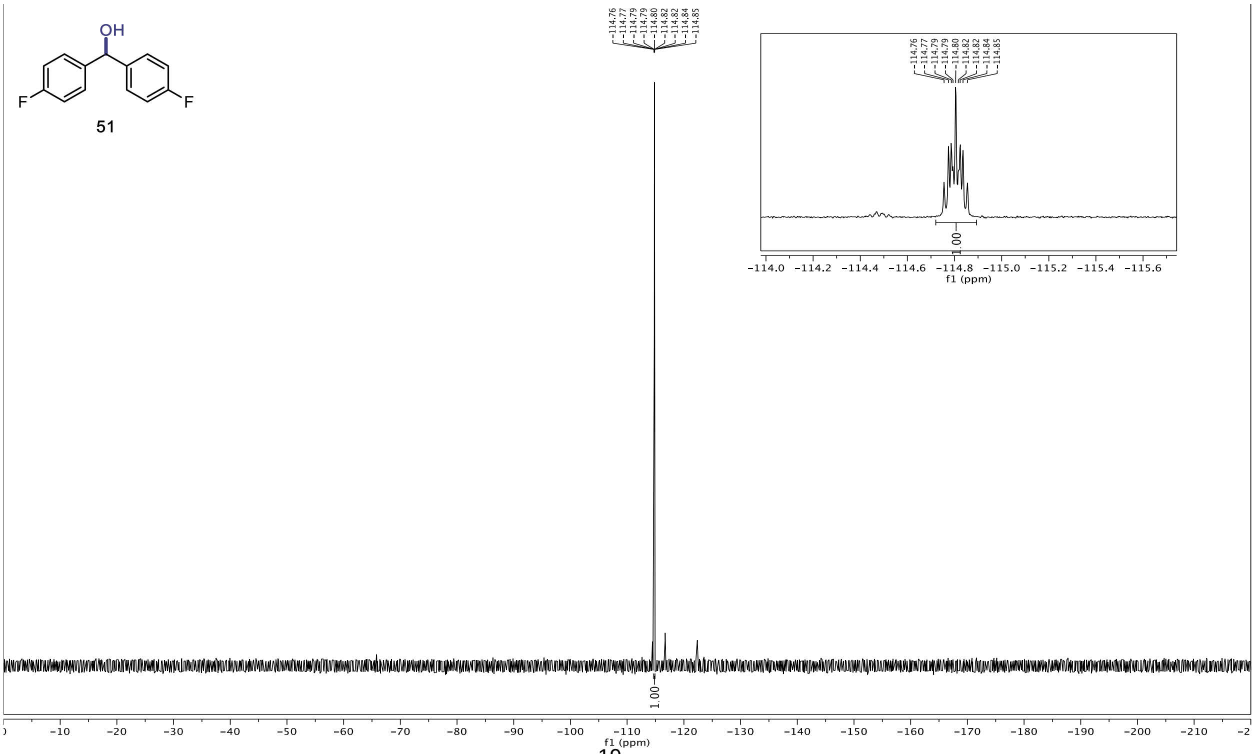

Supplementary Figure 179. ^{19}F NMR (282 MHz, $CDCl_3$) of **51**.

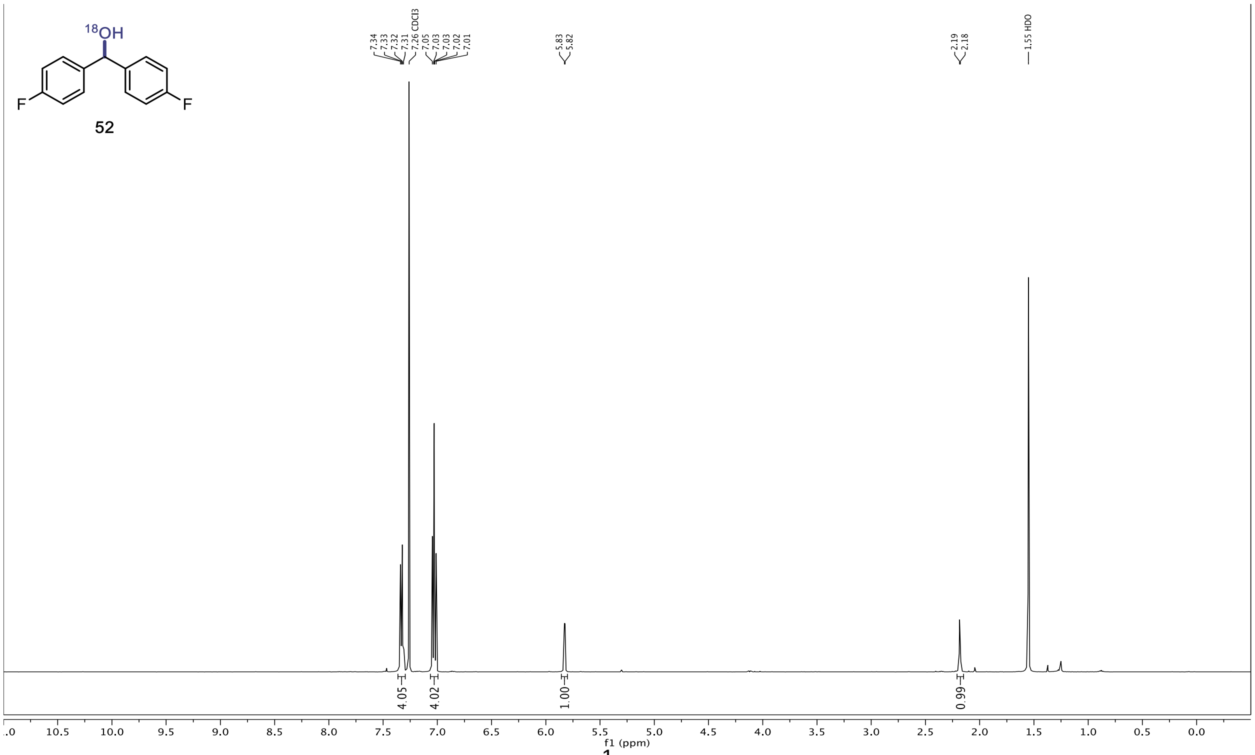

Supplementary Figure 180. ^{1}H NMR (500 MHz, $CDCl_3$) of **52**.

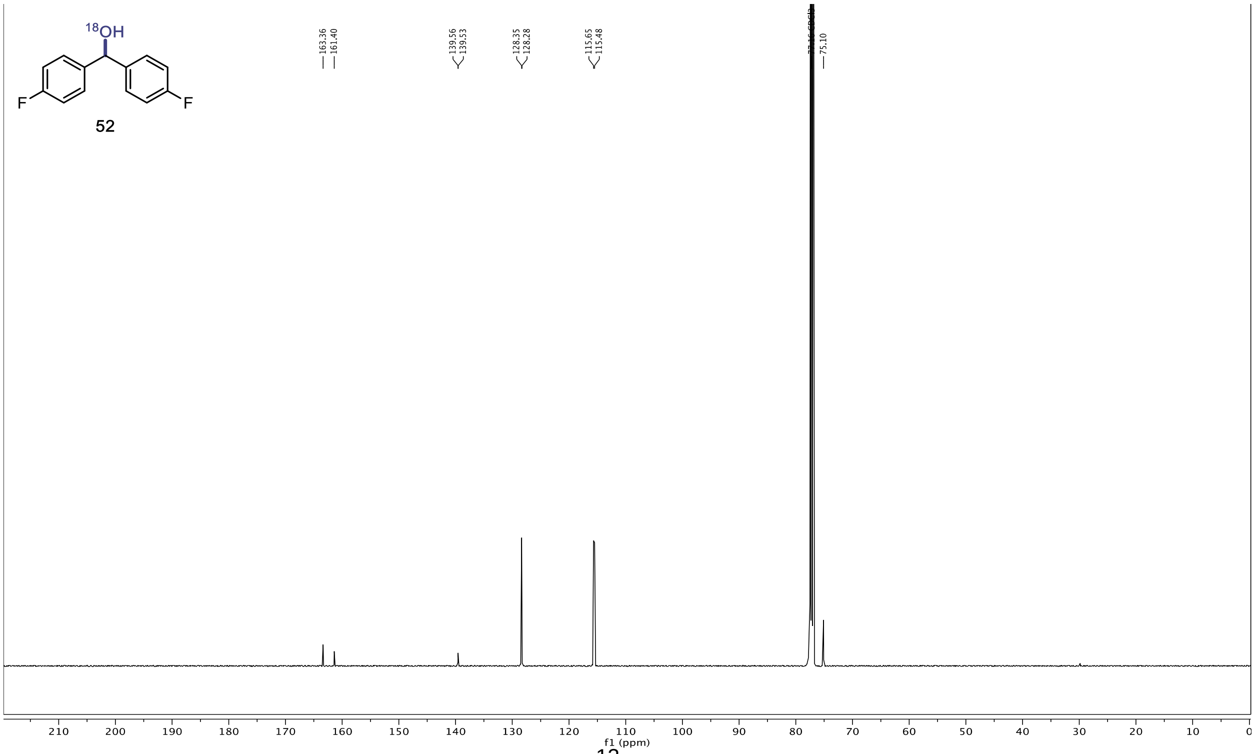

Supplementary Figure 181. ^{13}C NMR (126 MHz, $CDCl_3$) of **52**.

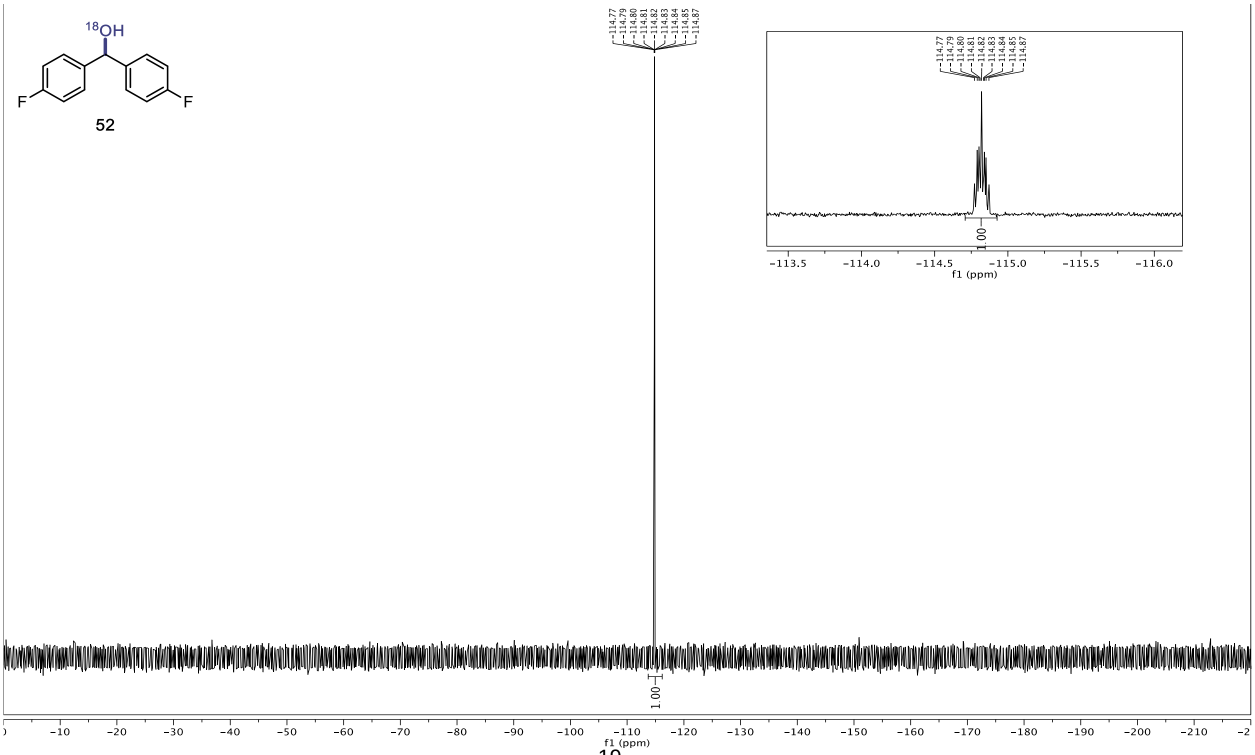

Supplementary Figure 182. ^{19}F NMR (282 MHz, $CDCl_3$) of **52**.

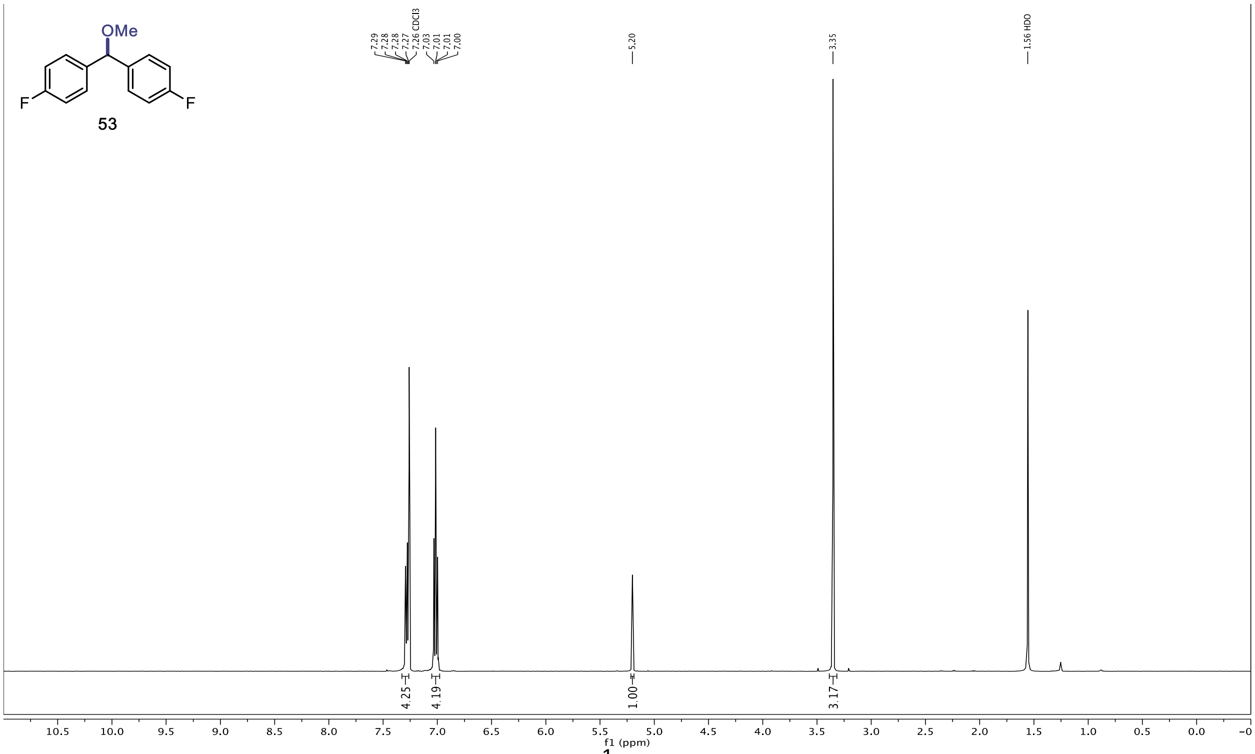

Supplementary Figure 183. ^{1}H NMR (500 MHz, $CDCl_3$) of **53**.

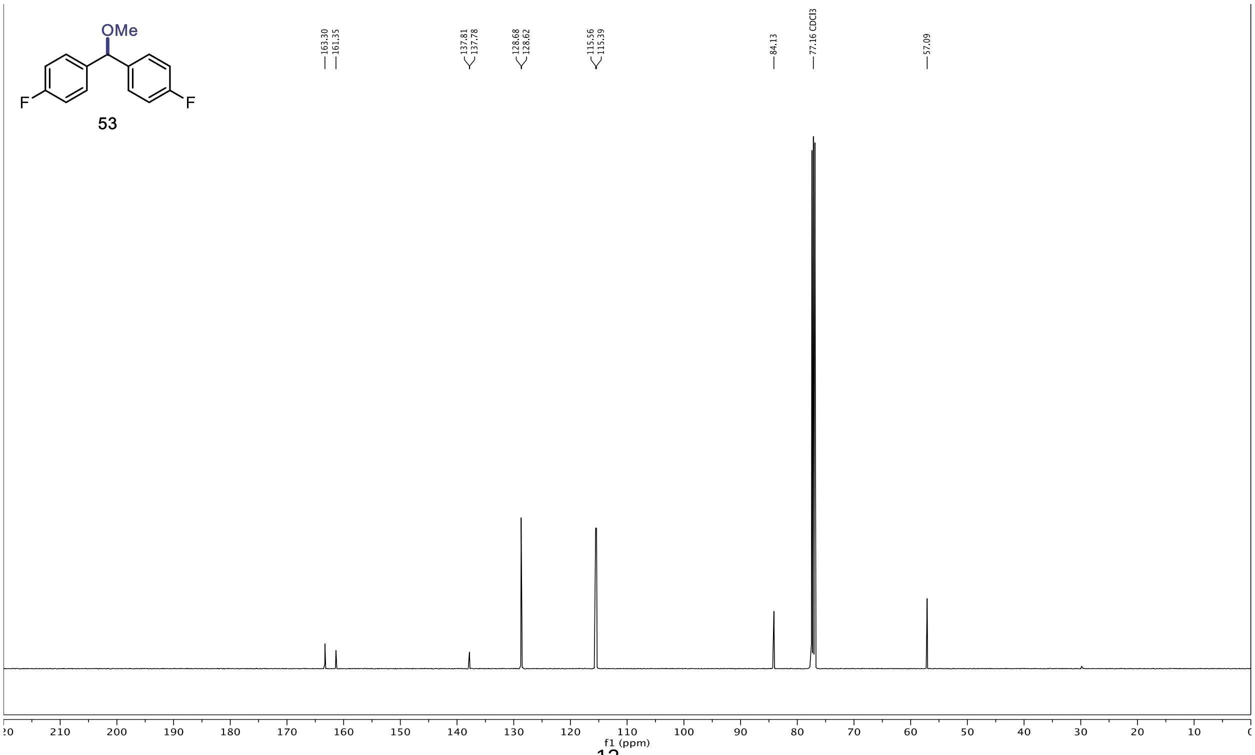

Supplementary Figure 184. ^{13}C NMR (126 MHz, $CDCl_3$) of **53**.

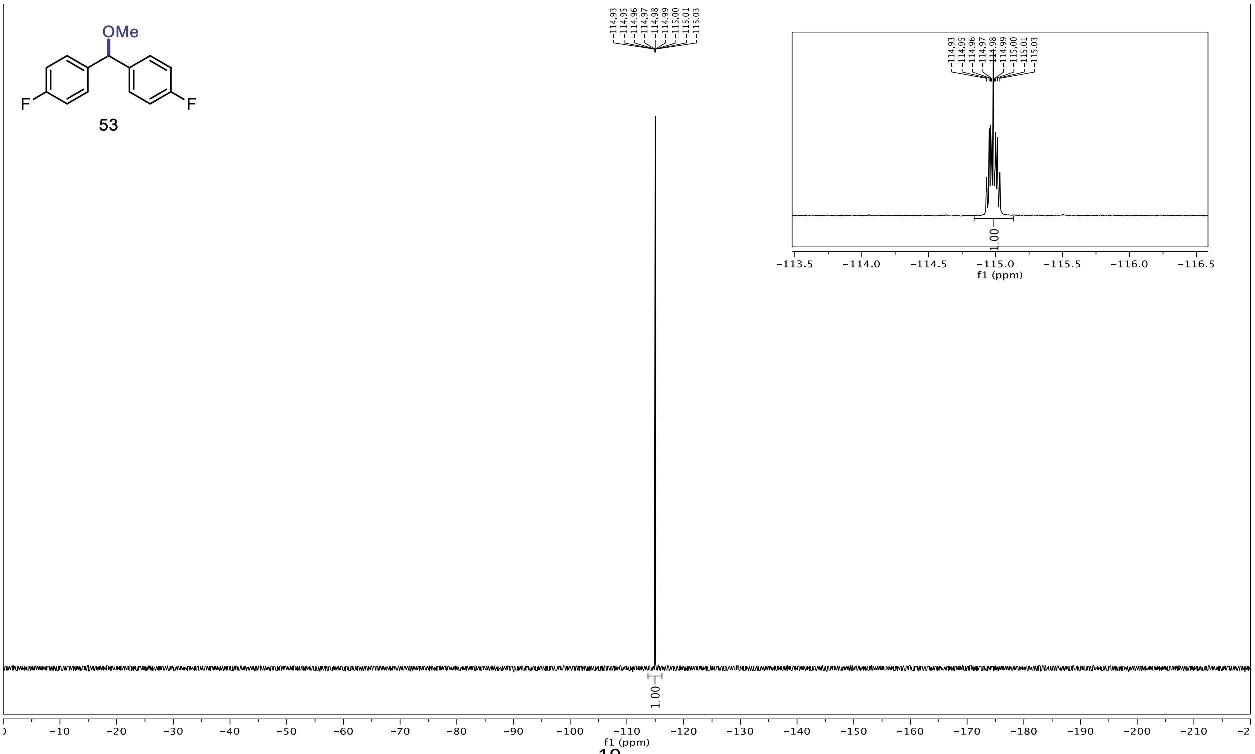

Supplementary Figure 185. ^{19}F NMR (282 MHz, $CDCl_3$) of **53**.

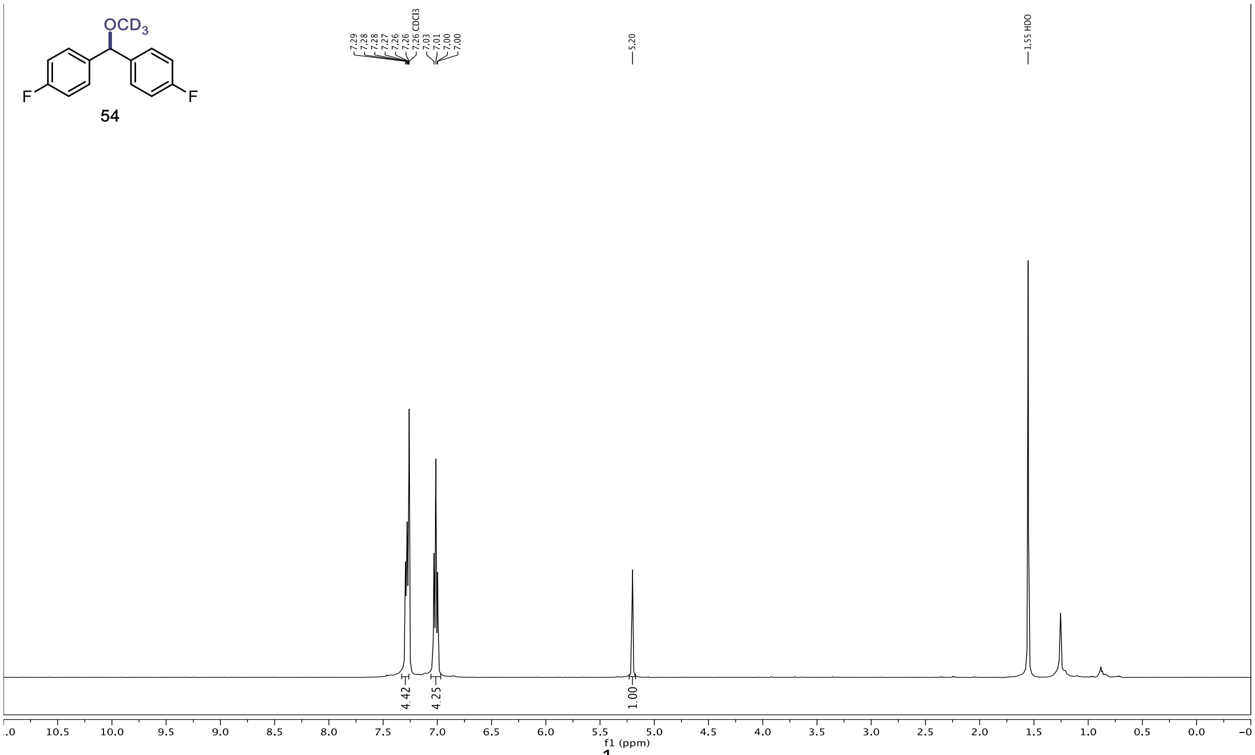

Supplementary Figure 186. ^{1}H NMR (500 MHz, $CDCl_3$) of **54**.

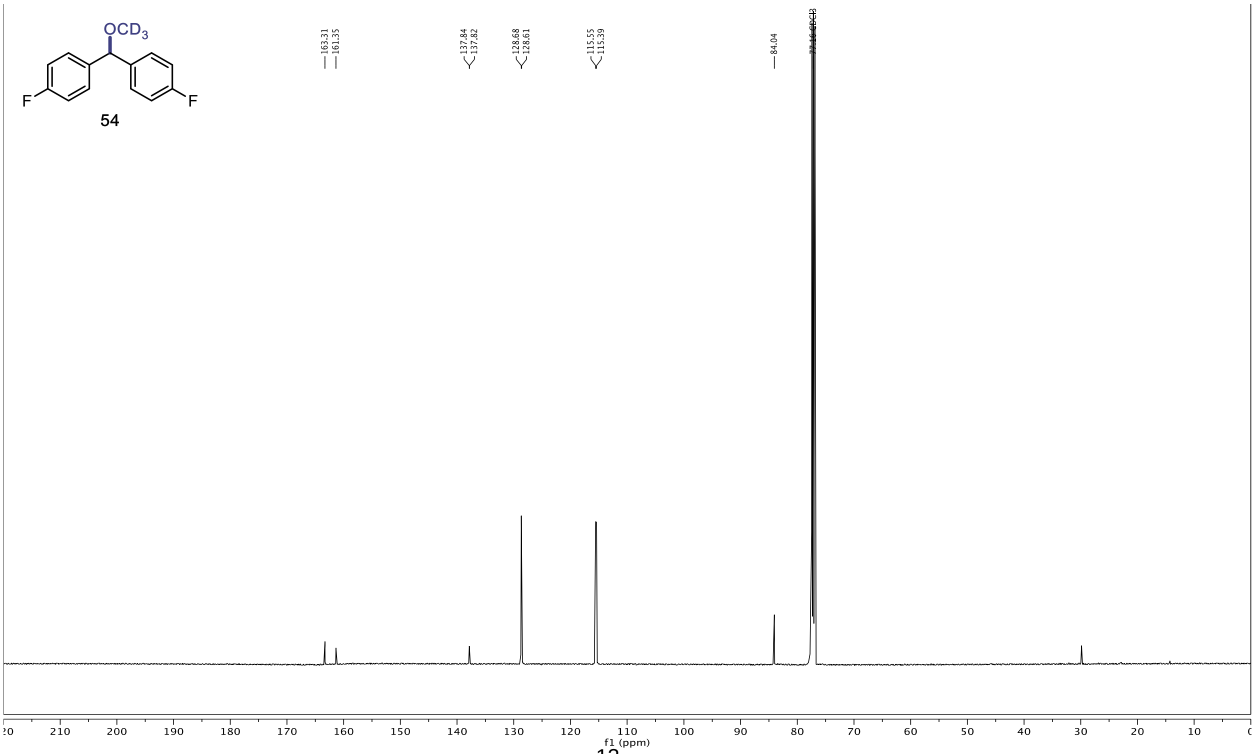

Supplementary Figure 187. ^{13}C NMR (126 MHz, $CDCl_3$) of **54**.

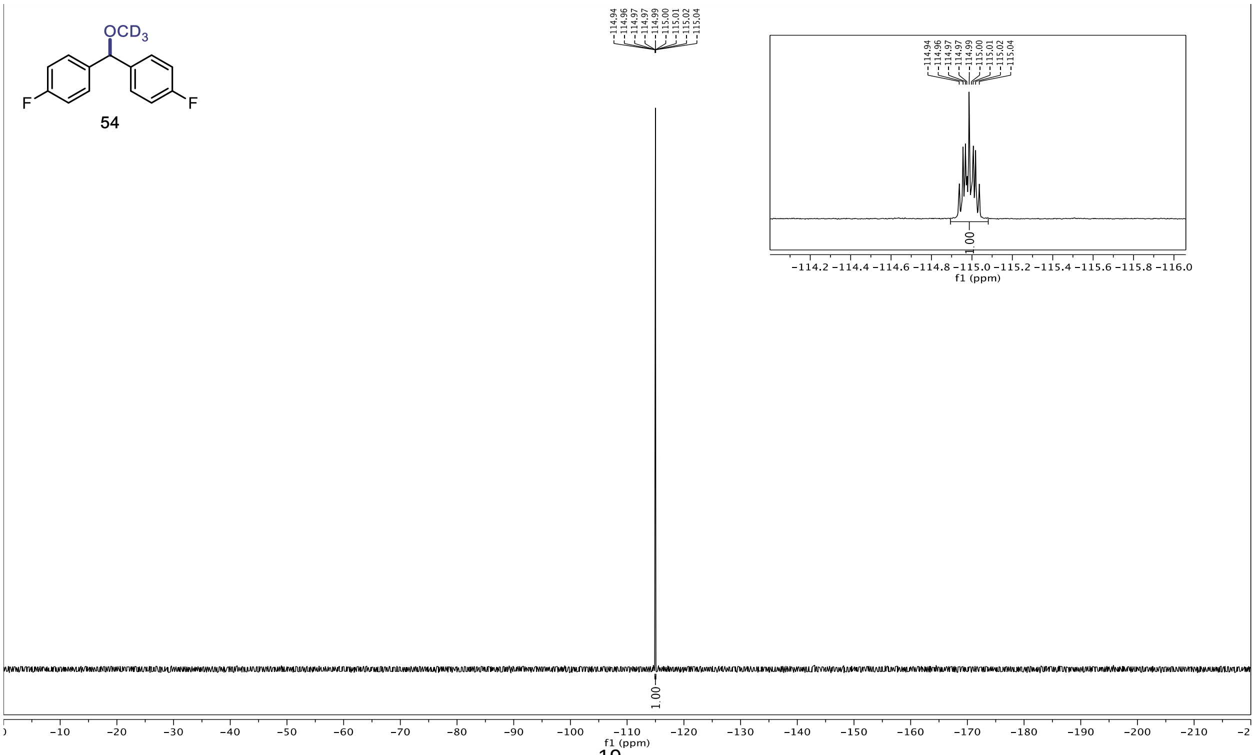

Supplementary Figure 188. ^{19}F NMR (282 MHz, $CDCl_3$) of **54**.

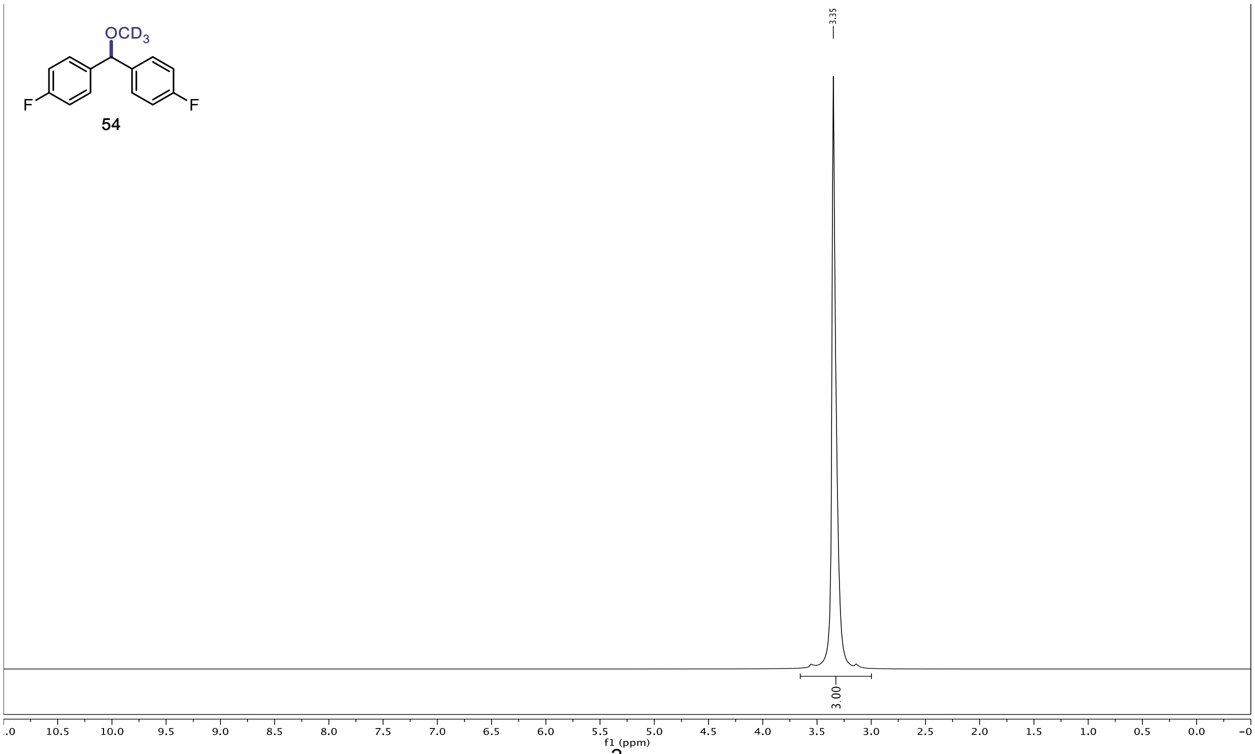

Supplementary Figure 189. ^{2}H NMR (77 MHz, $CDCl_3$) of **54**.

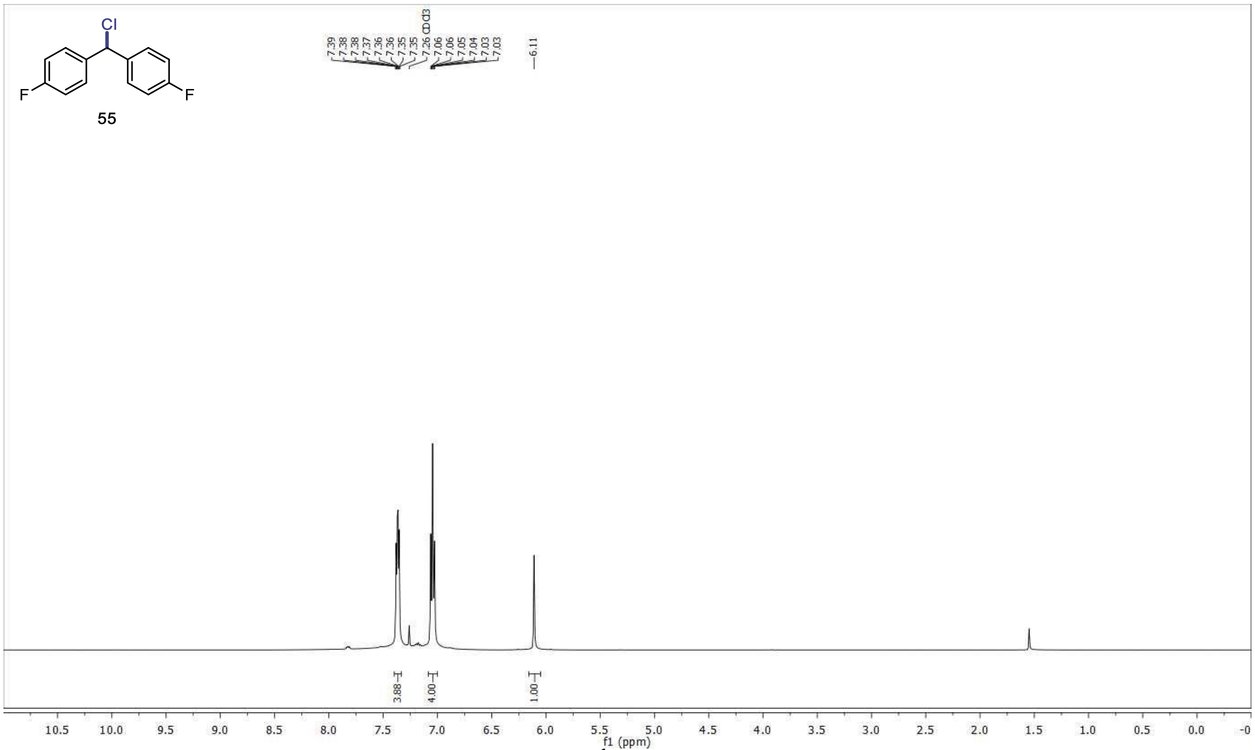

Supplementary Figure 190. ^{1}H NMR (500 MHz, $CDCl_3$) of **55**.

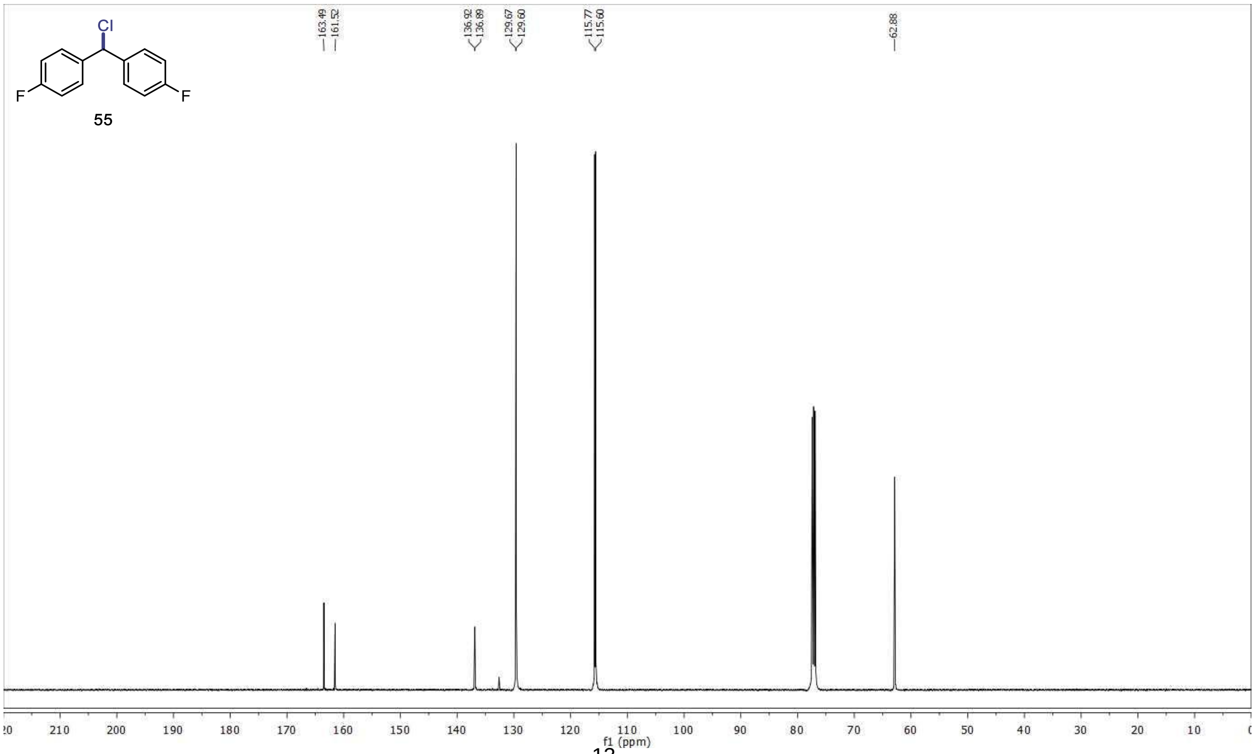

Supplementary Figure 191. ^{13}C NMR (126 MHz, $CDCl_3$) of **55**.

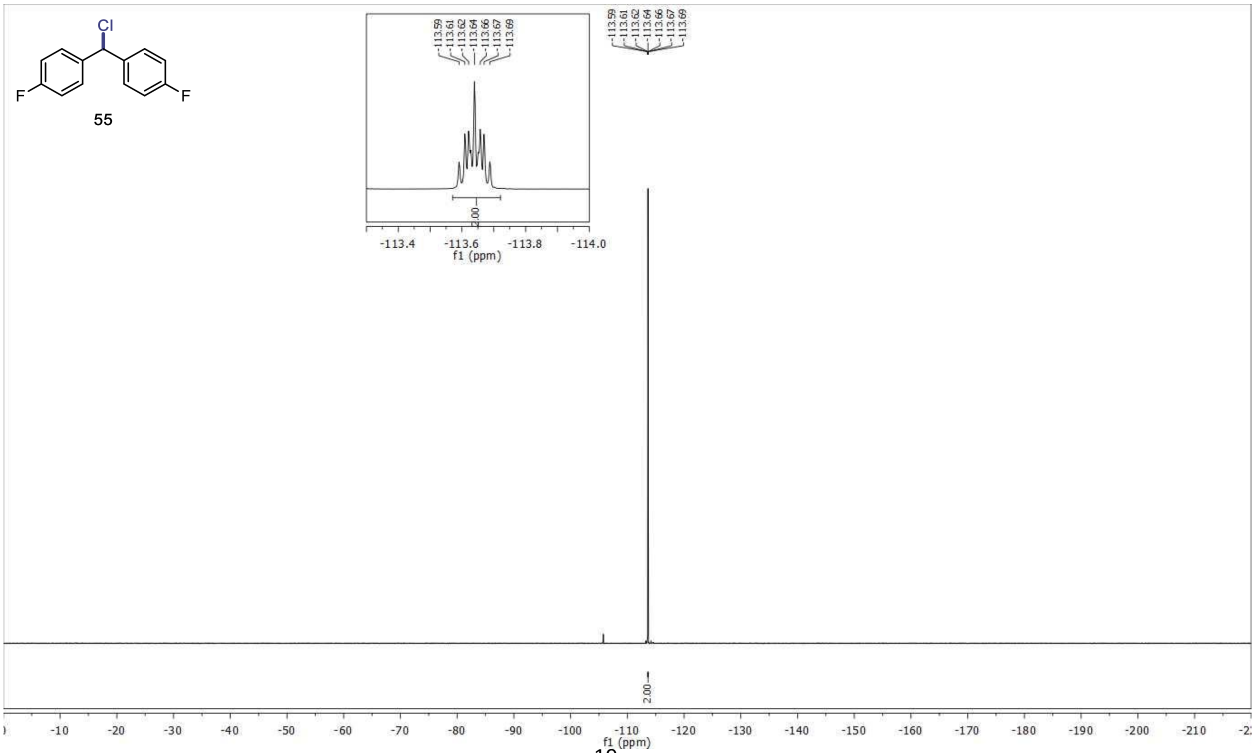

Supplementary Figure 192. ^{19}F NMR (282 MHz, $CDCl_3$) of **55**.

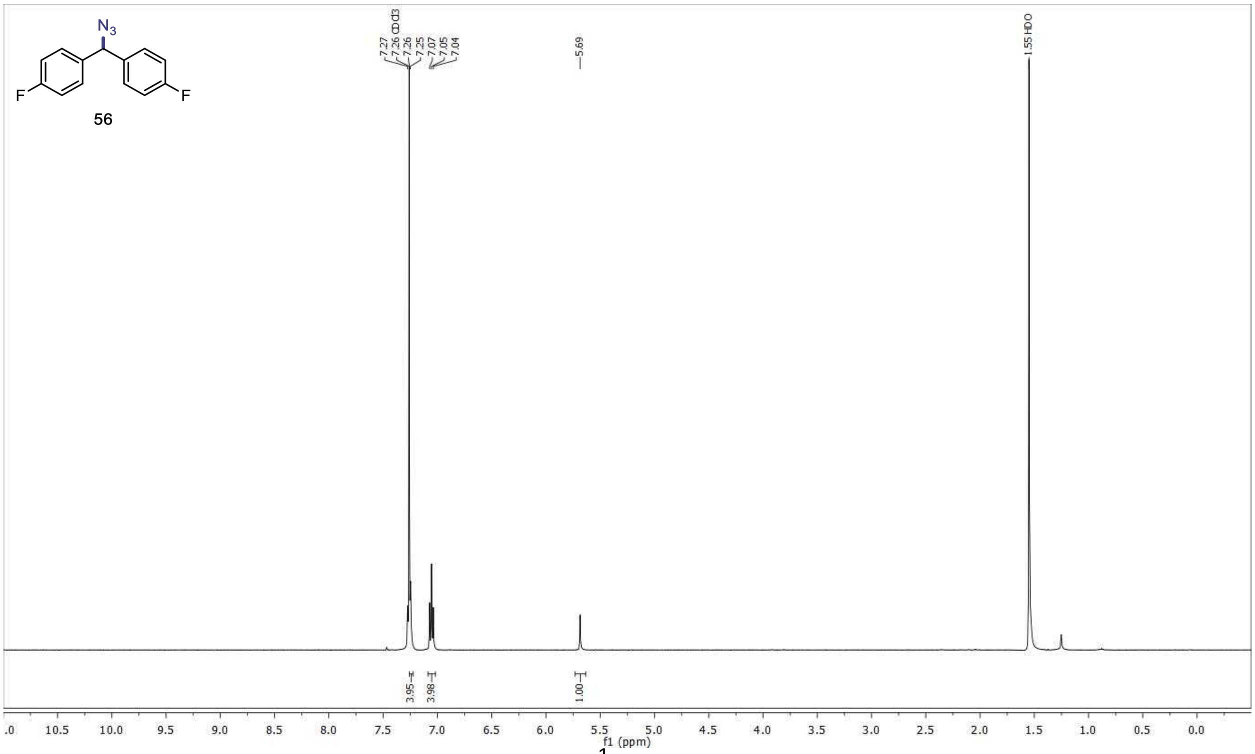

Supplementary Figure 193. ^{1}H NMR (500 MHz, $CDCl_3$) of **56**.

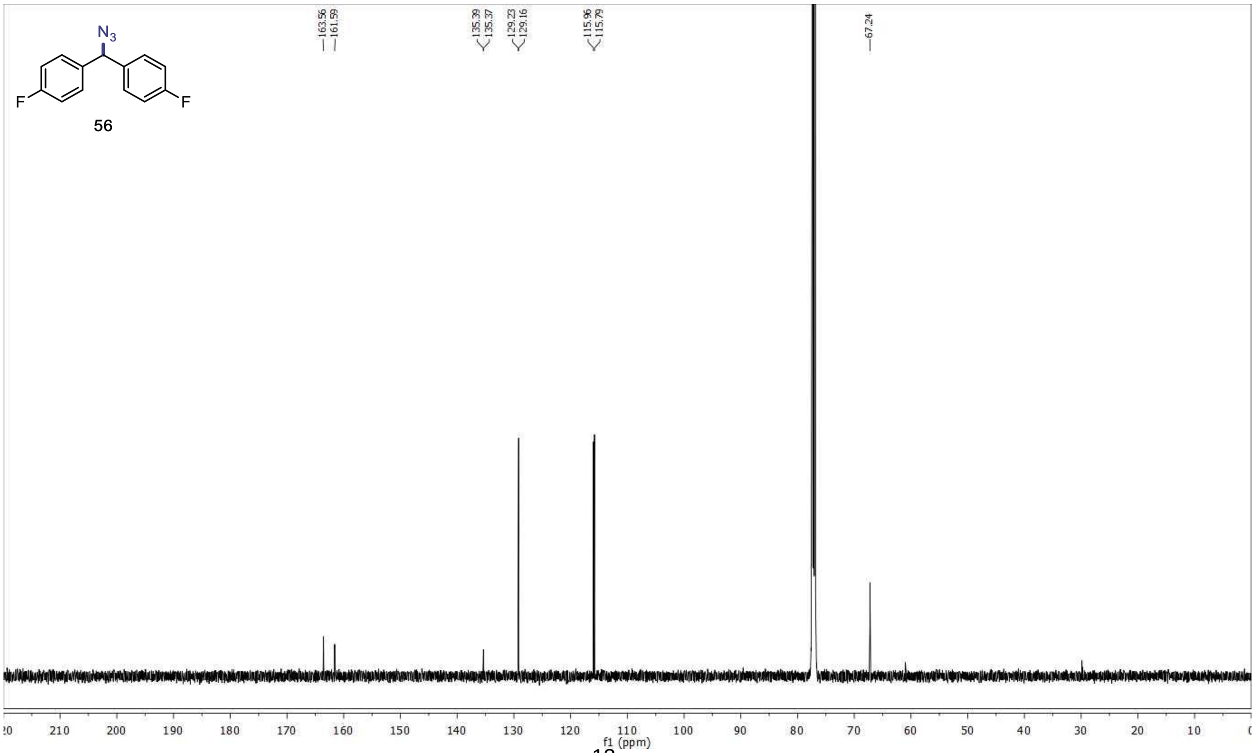

Supplementary Figure 194. ^{13}C NMR (126 MHz, $CDCl_3$) of **56**.

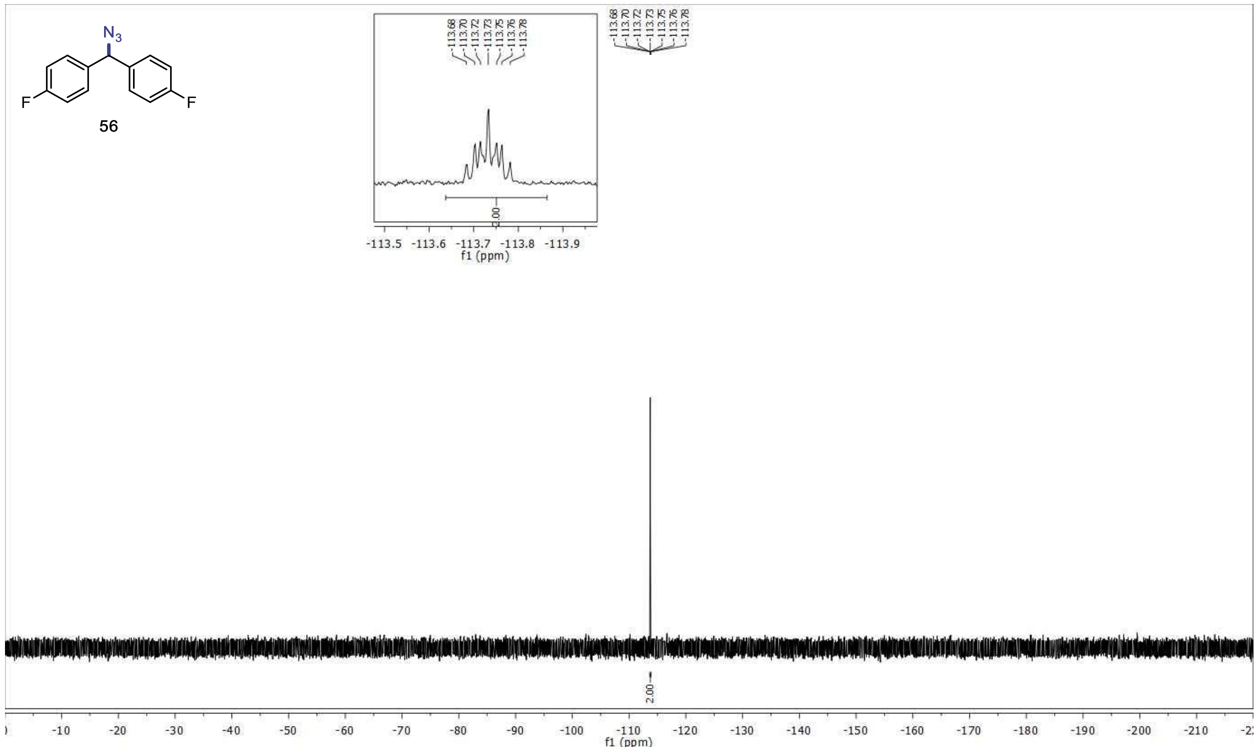

Supplementary Figure 195. ^{19}F NMR (282 MHz, $CDCl_3$) of **56**.

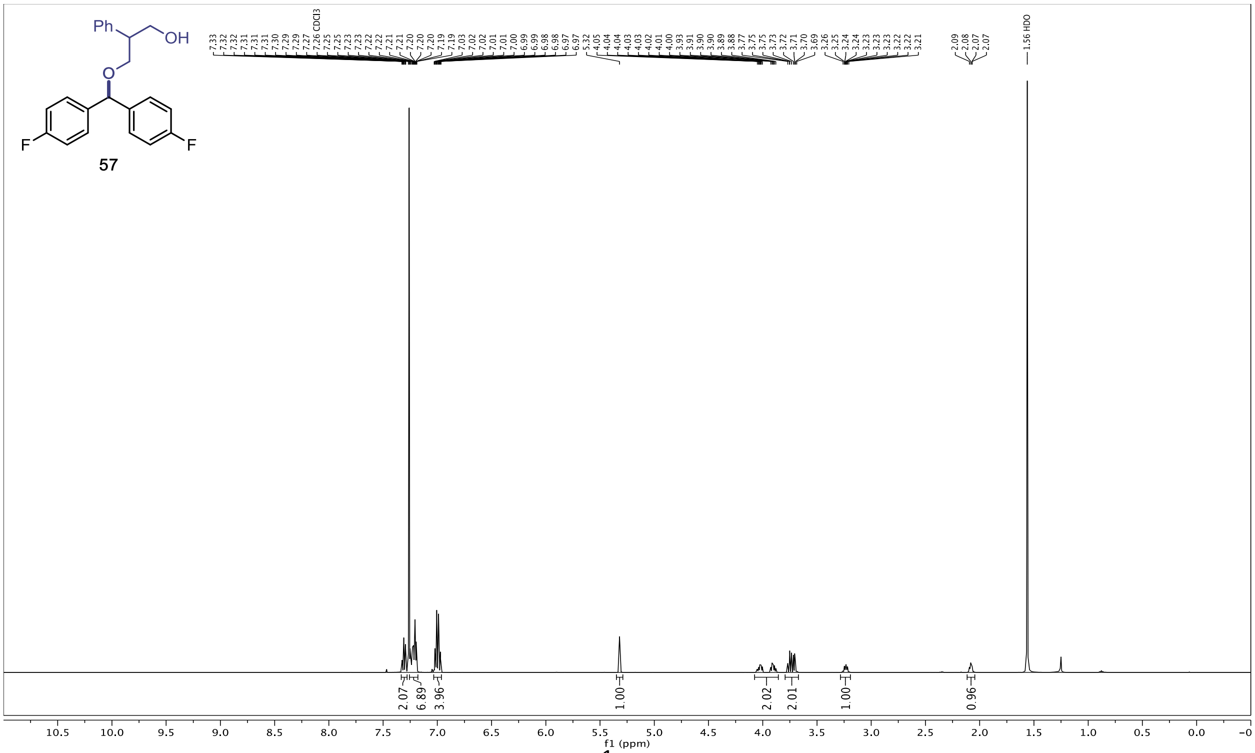

Supplementary Figure 196. ^{1}H NMR (500 MHz, $CDCl_3$) of **57**.

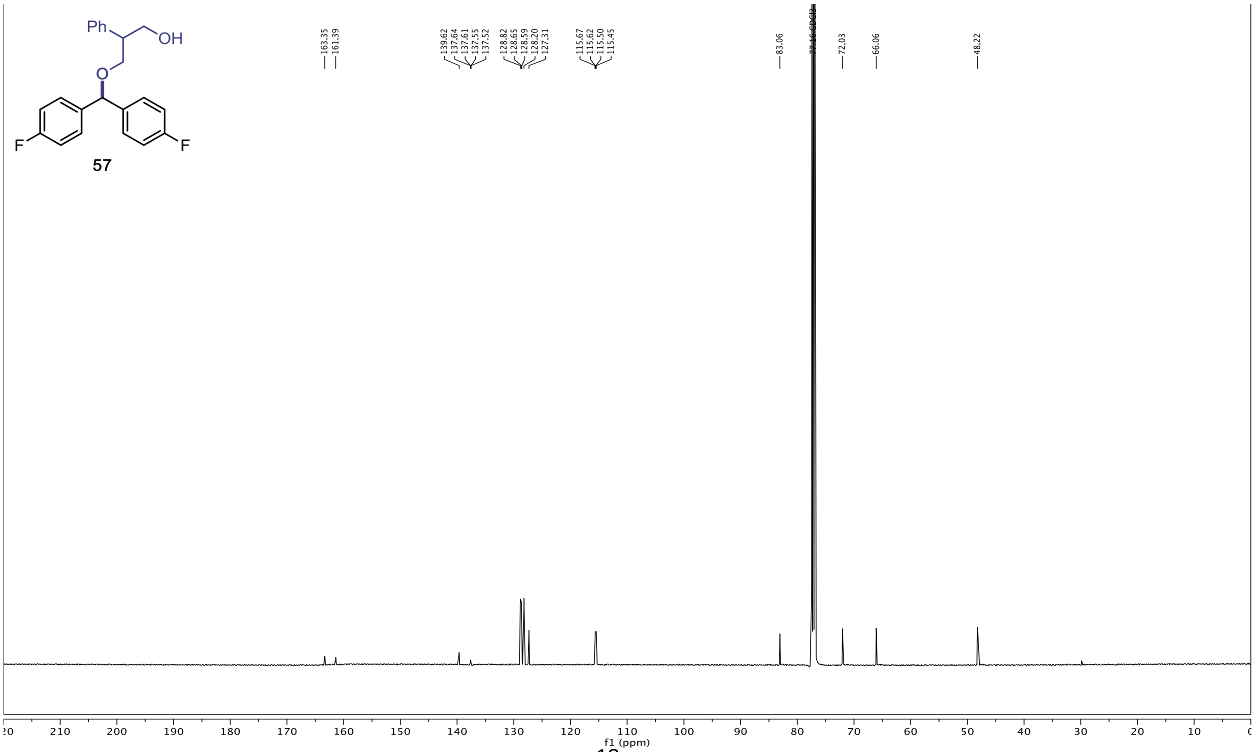

Supplementary Figure 197. ^{13}C NMR (126 MHz, $CDCl_3$) of **57**.

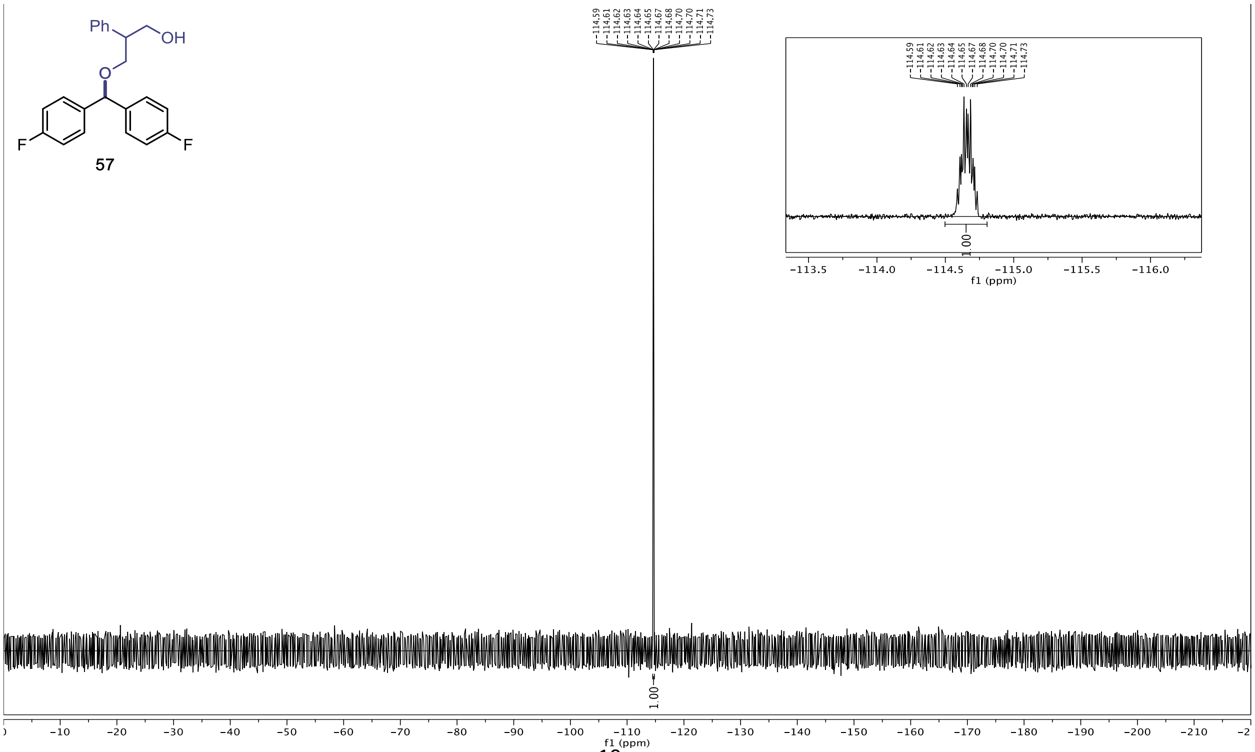

Supplementary Figure 198. ^{19}F NMR (282 MHz, $CDCl_3$) of **57**.

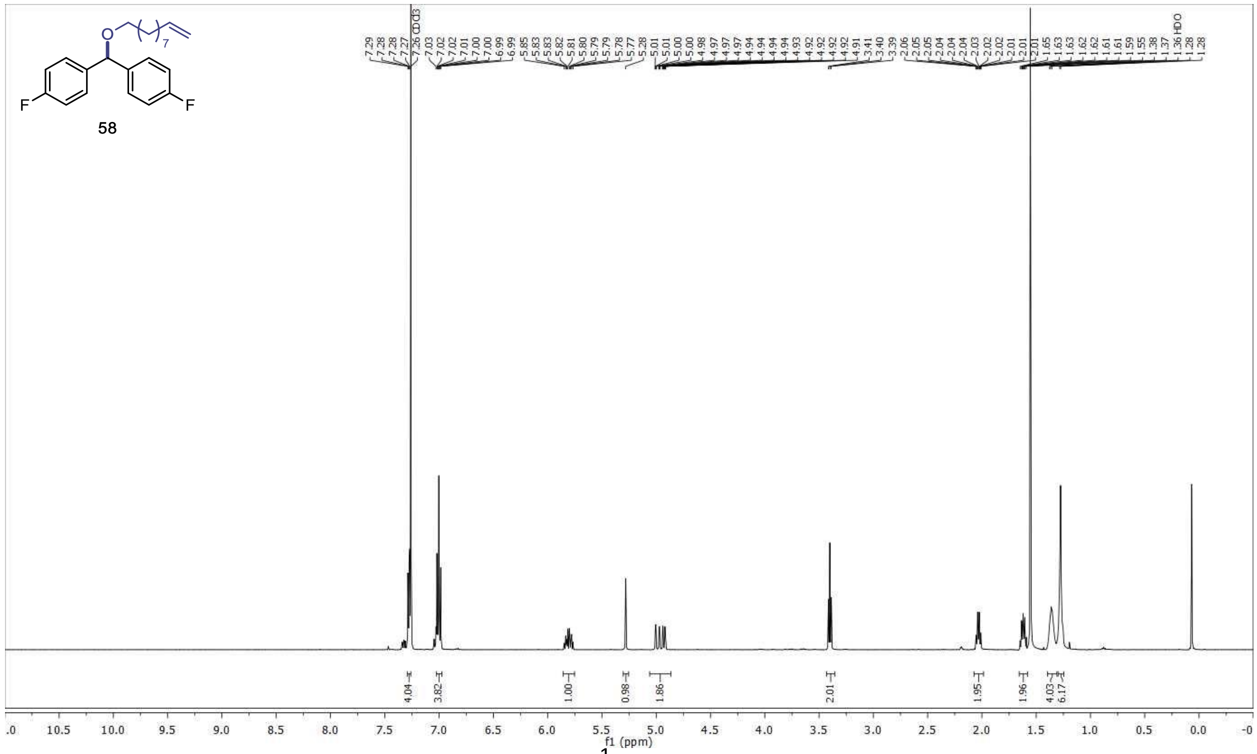

Supplementary Figure 199. ^{1}H NMR (500 MHz, $CDCl_3$) of **58**.

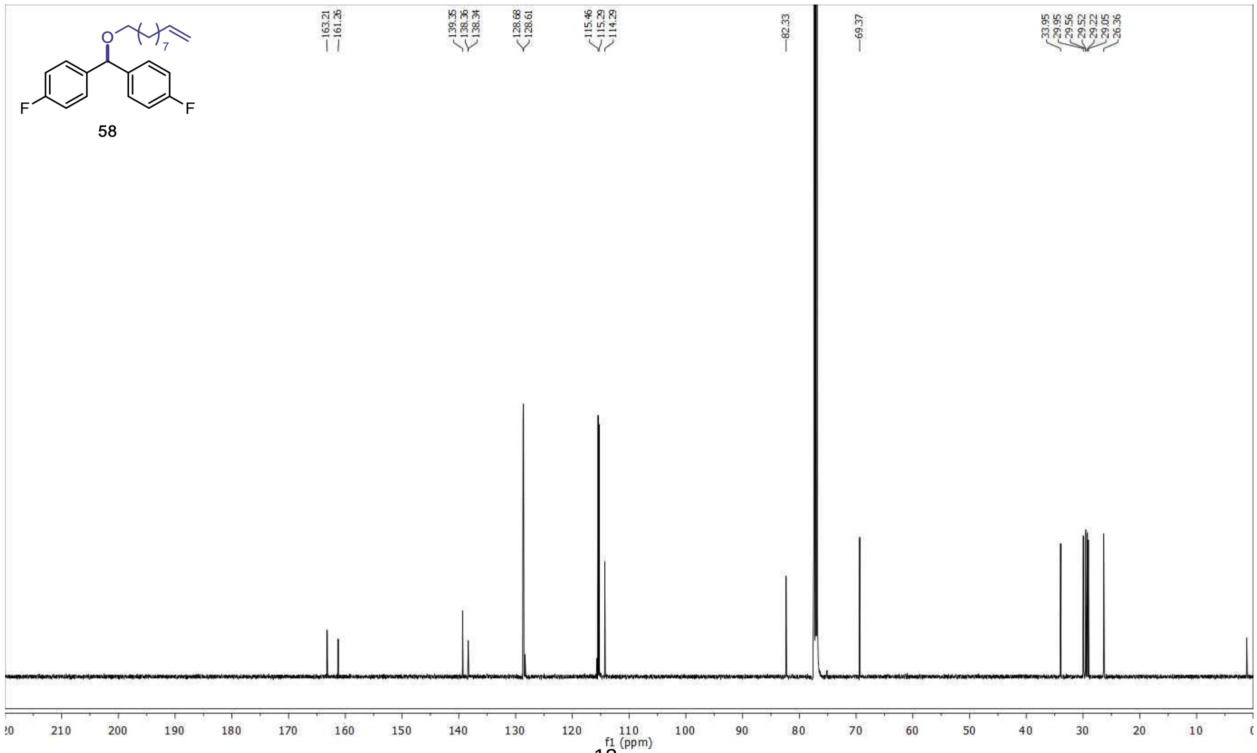

Supplementary Figure 200. ^{13}C NMR (126 MHz, $CDCl_3$) of **58**.

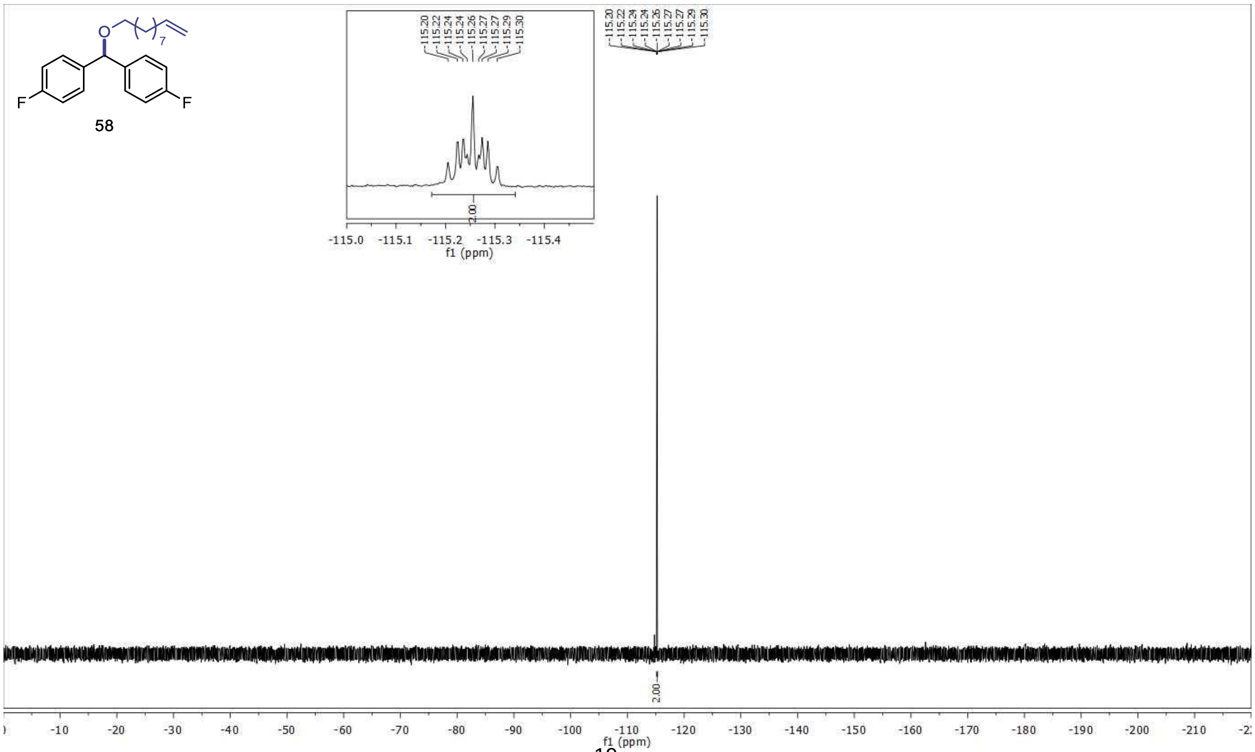

Supplementary Figure 201. ^{19}F NMR (282 MHz, $CDCl_3$) of **58**.

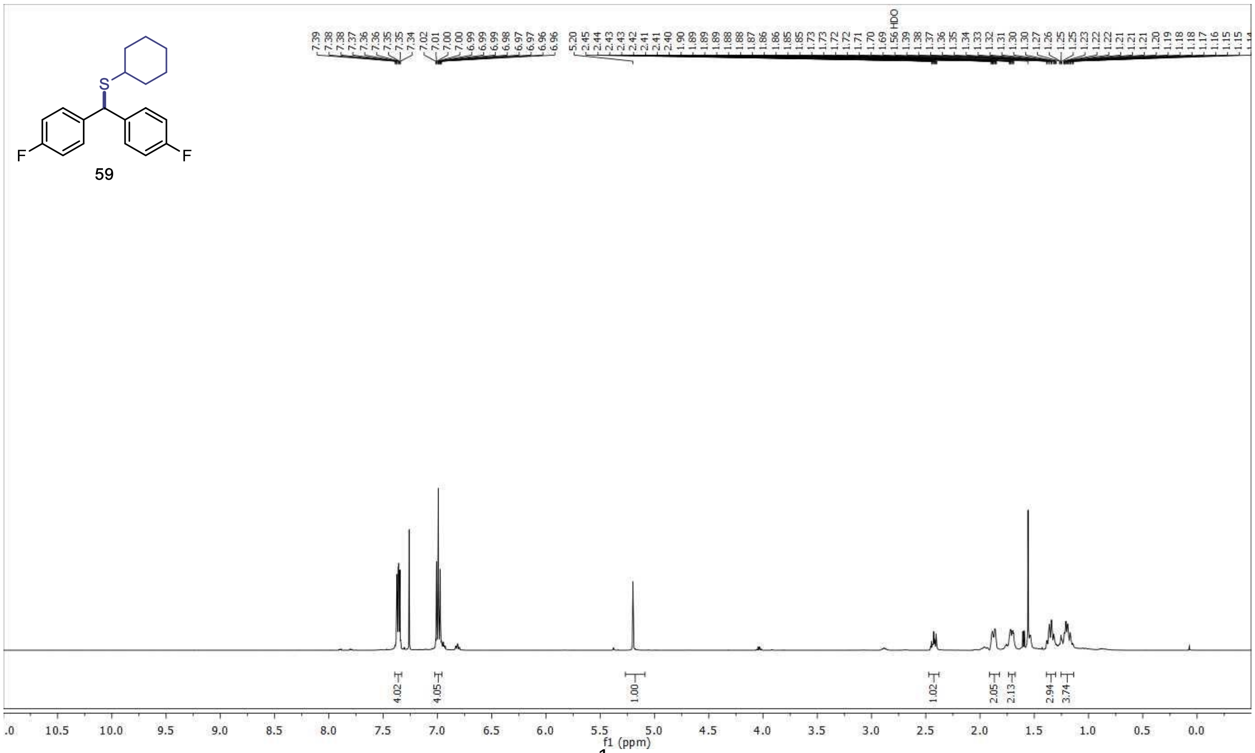

Supplementary Figure 202. ^{1}H NMR (500 MHz, $CDCl_3$) of **59**.

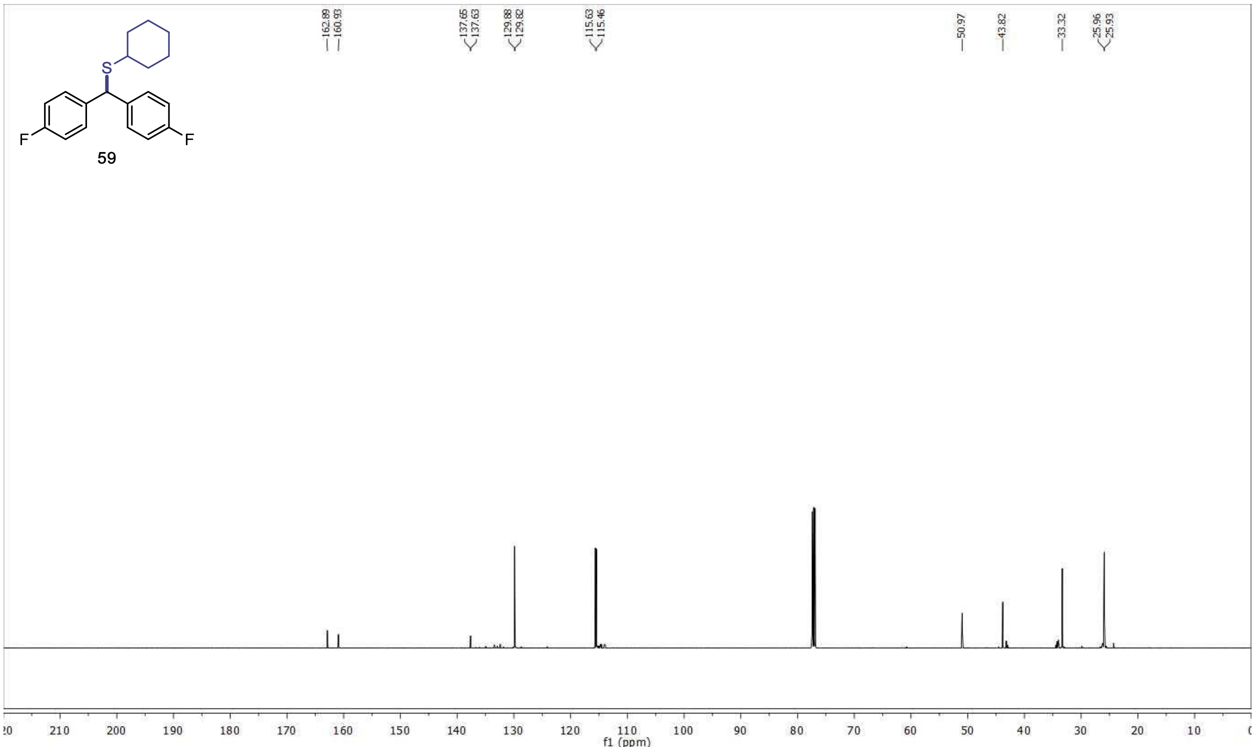

Supplementary Figure 203. ^{13}C NMR (126 MHz, $CDCl_3$) of **59**.

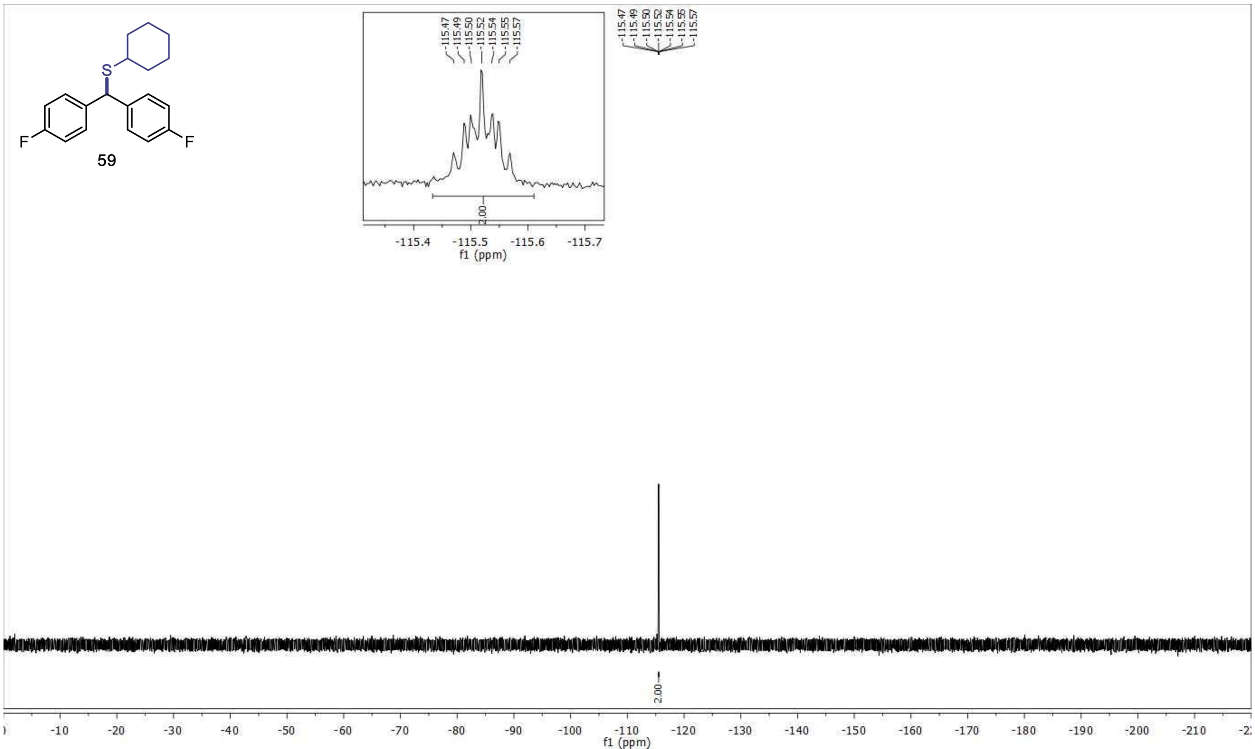

Supplementary Figure 204. ^{19}F NMR (282 MHz, $CDCl_3$) of **59**.

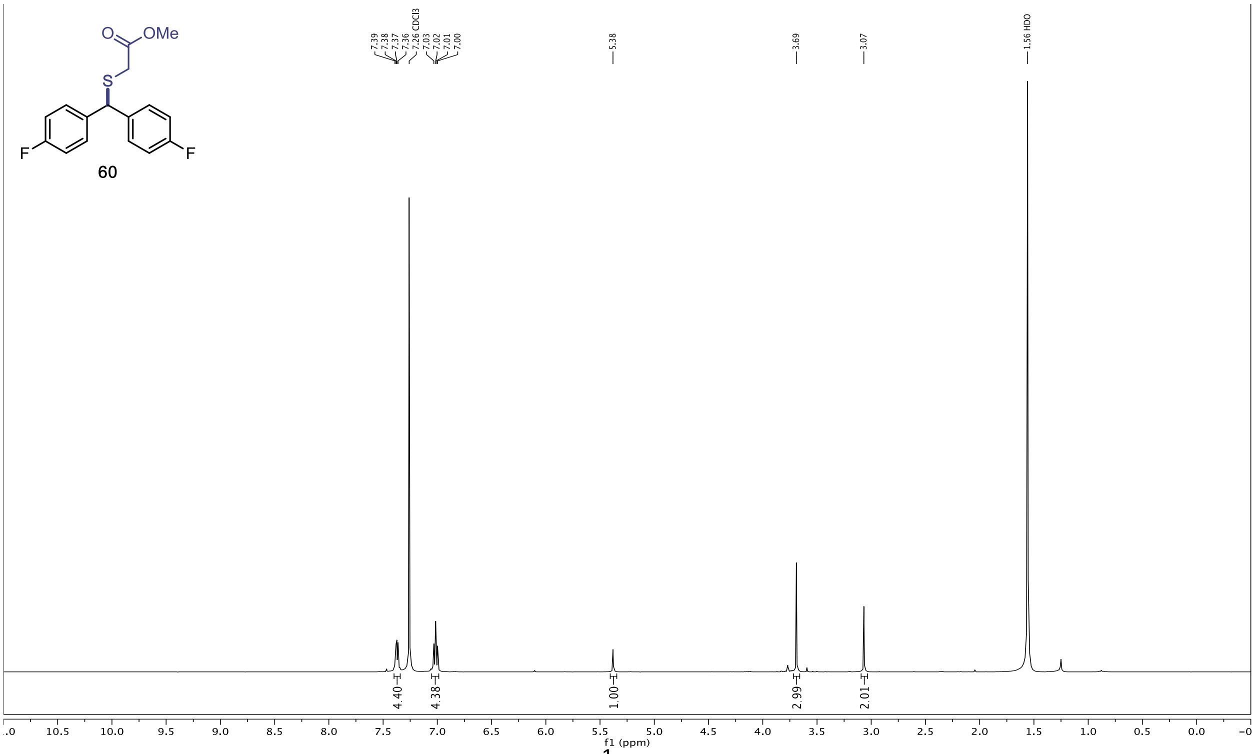

Supplementary Figure 205. ^{1}H NMR (500 MHz, $CDCl_3$) of **60**.

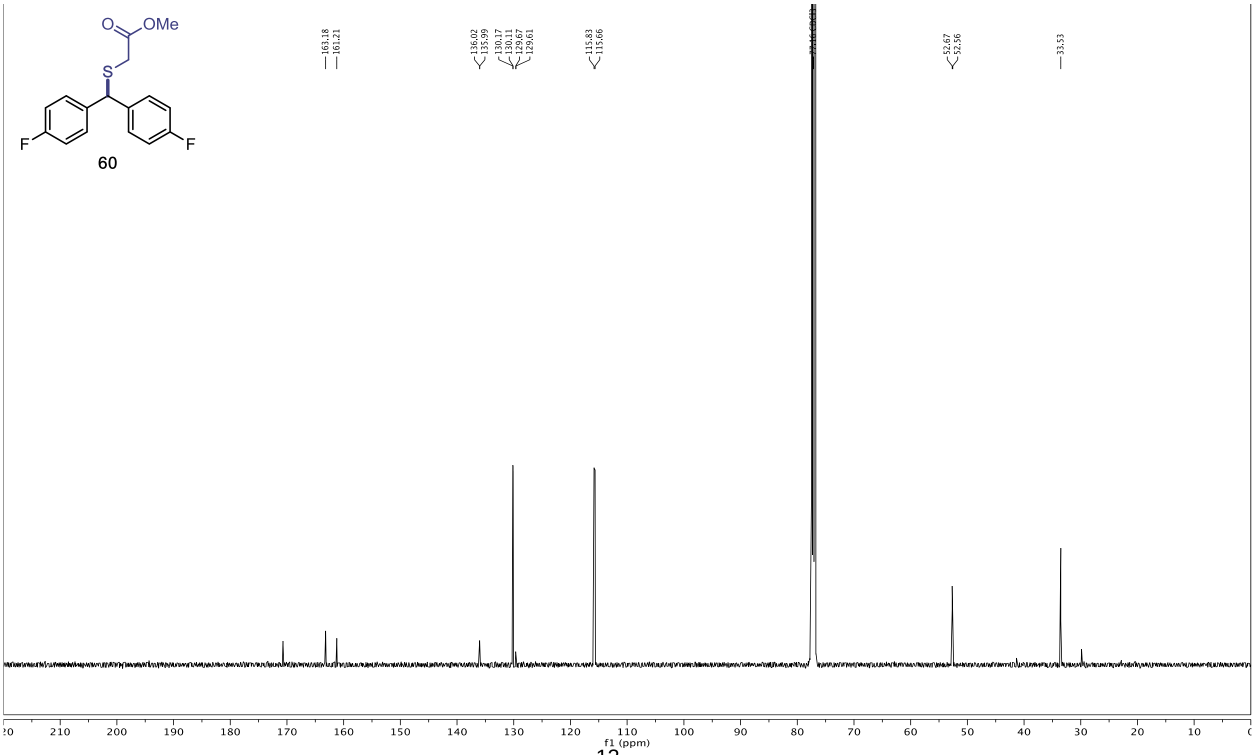

Supplementary Figure 206. ^{13}C NMR (126 MHz, $CDCl_3$) of **60**.

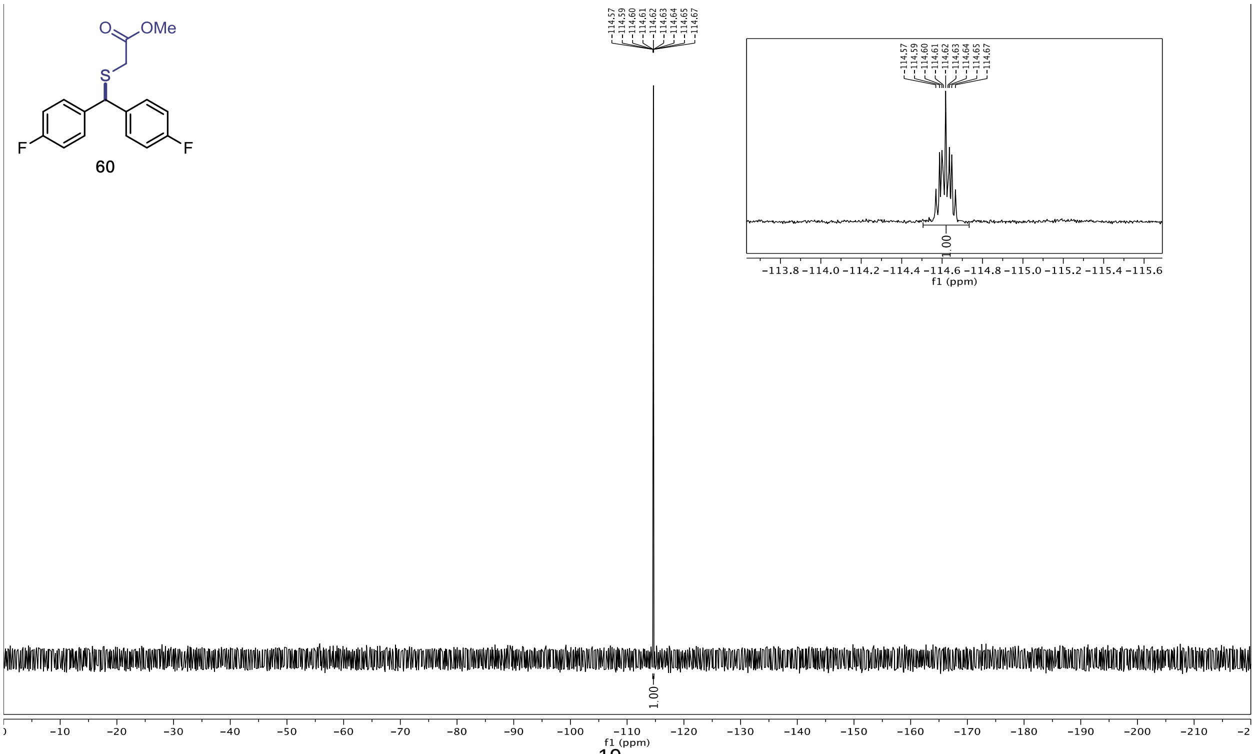

Supplementary Figure 207. ^{19}F NMR (282 MHz, $CDCl_3$) of **60**.

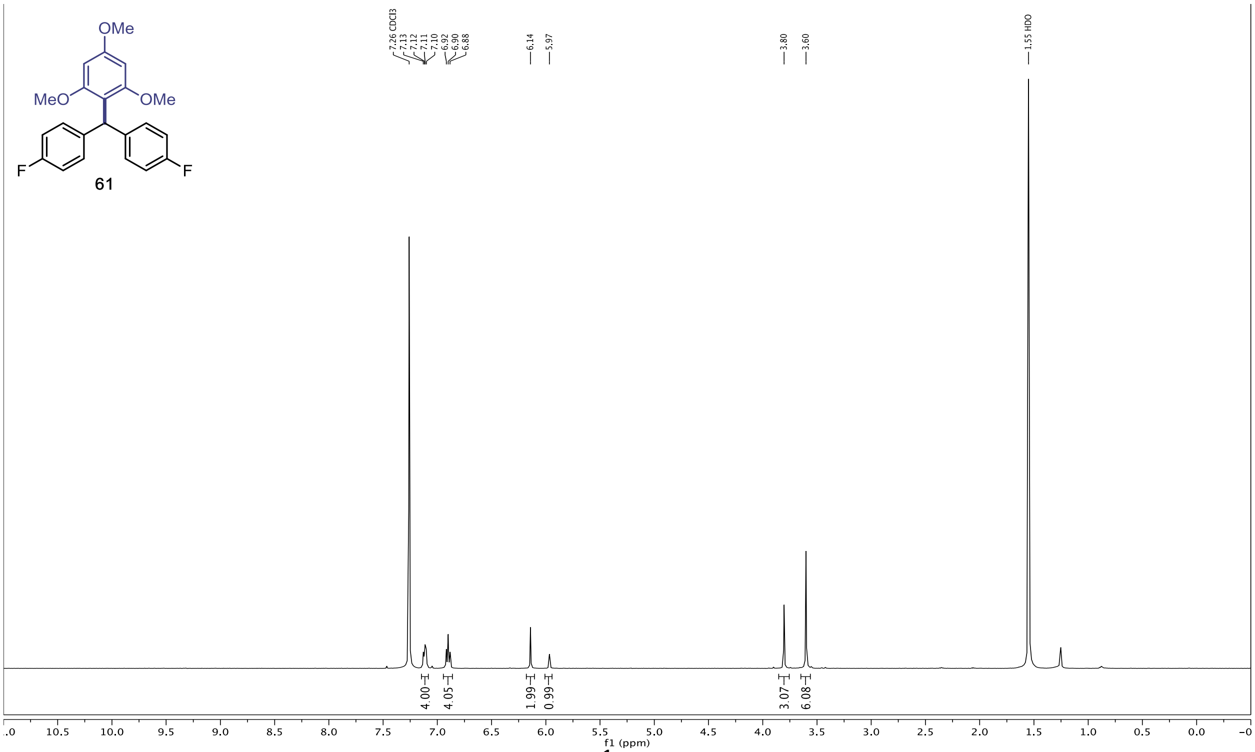

Supplementary Figure 208. ^{1}H NMR (500 MHz, $CDCl_3$) of **61**.

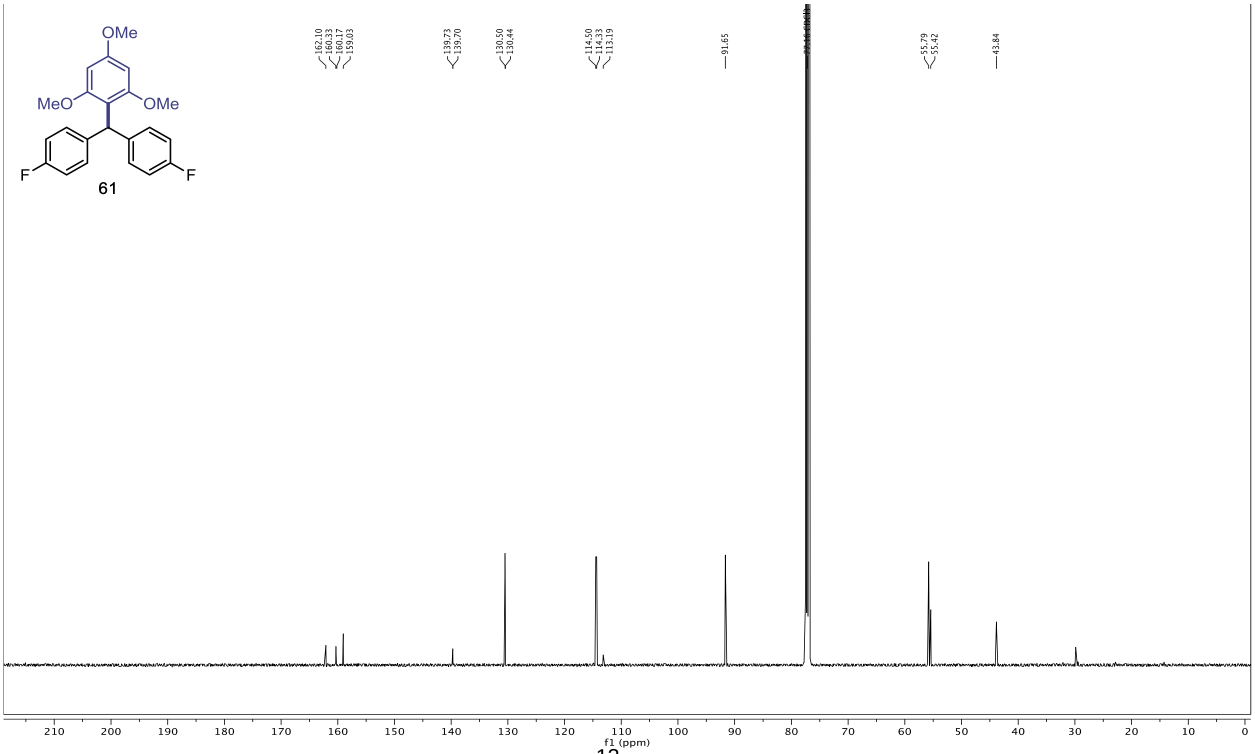

Supplementary Figure 209. ^{13}C NMR (126 MHz, $CDCl_3$) of **61**.

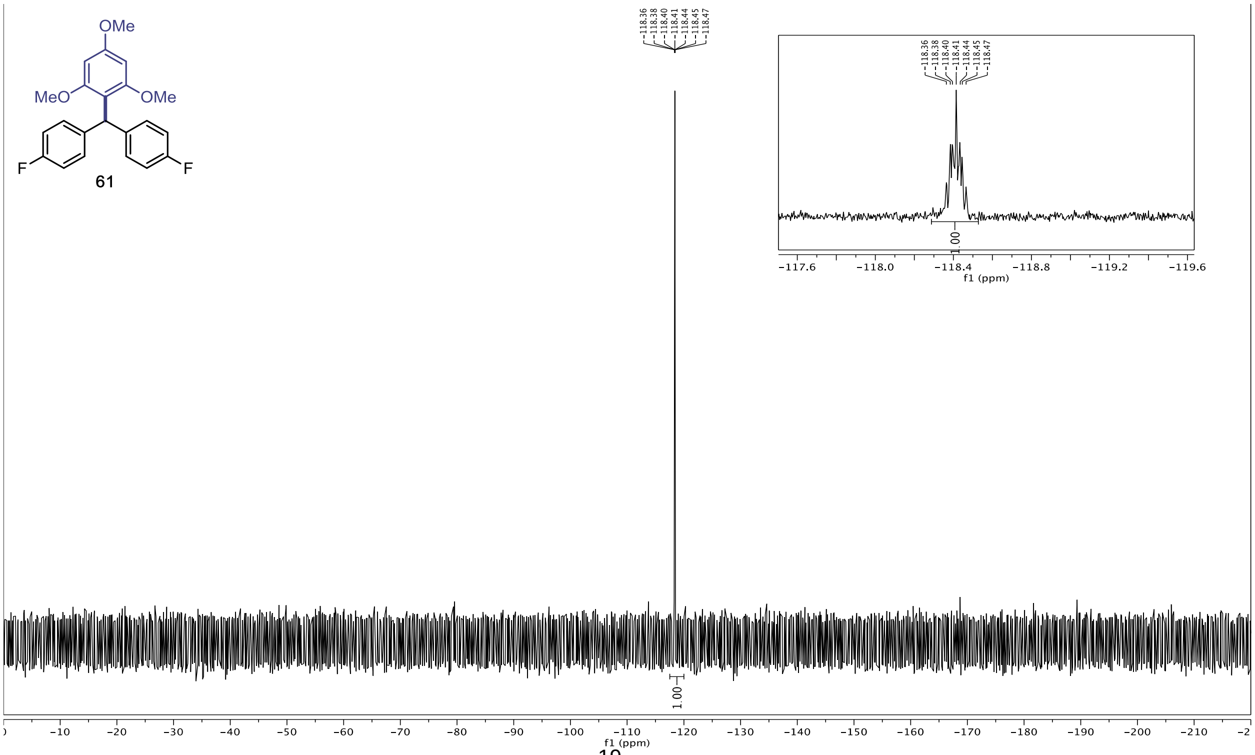

Supplementary Figure 210. ^{19}F NMR (282 MHz, $CDCl_3$) of **61**.

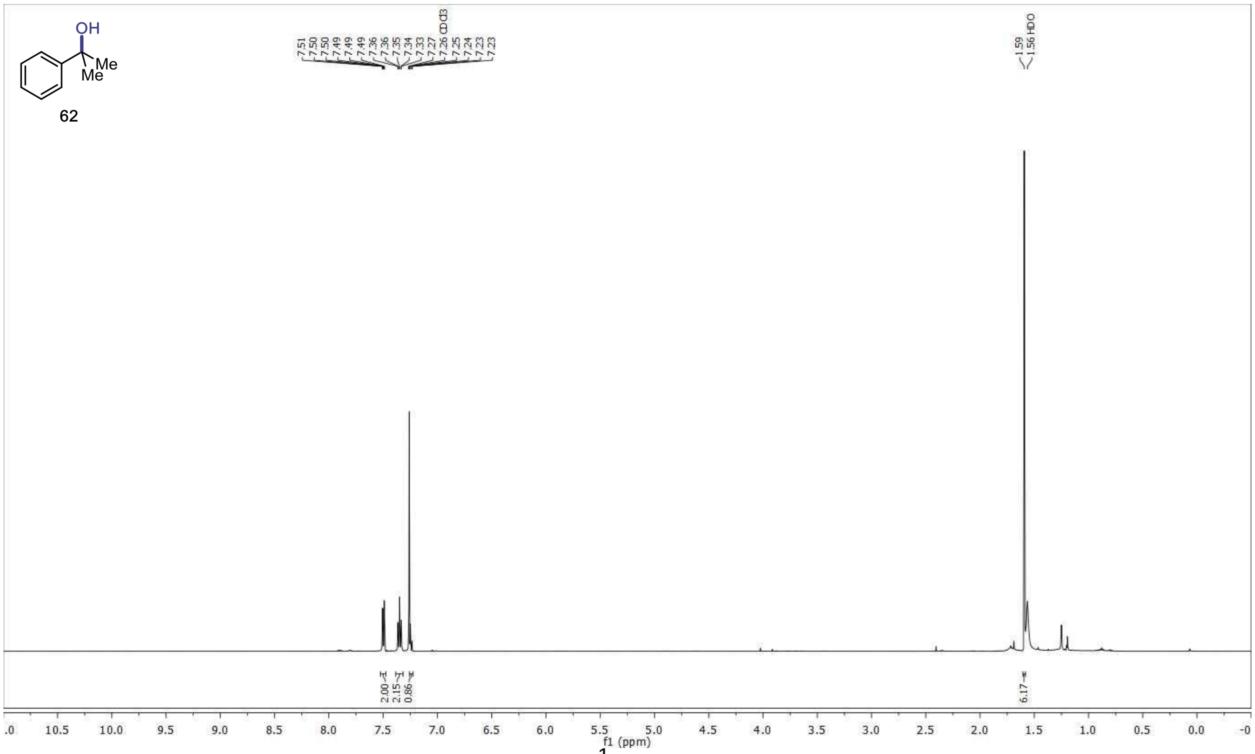

Supplementary Figure 211. ^{1}H NMR (500 MHz, $CDCl_3$) of **62**.

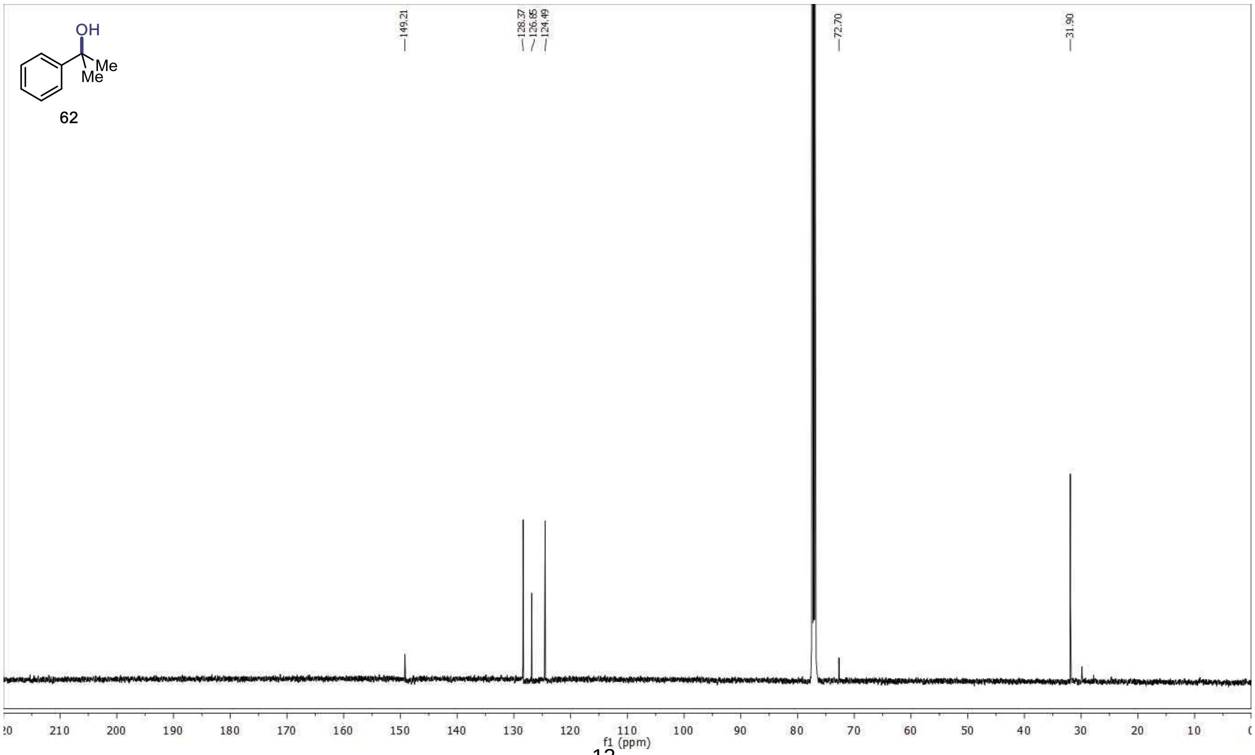

Supplementary Figure 212. ^{13}C NMR (126 MHz, $CDCl_3$) of **62**.

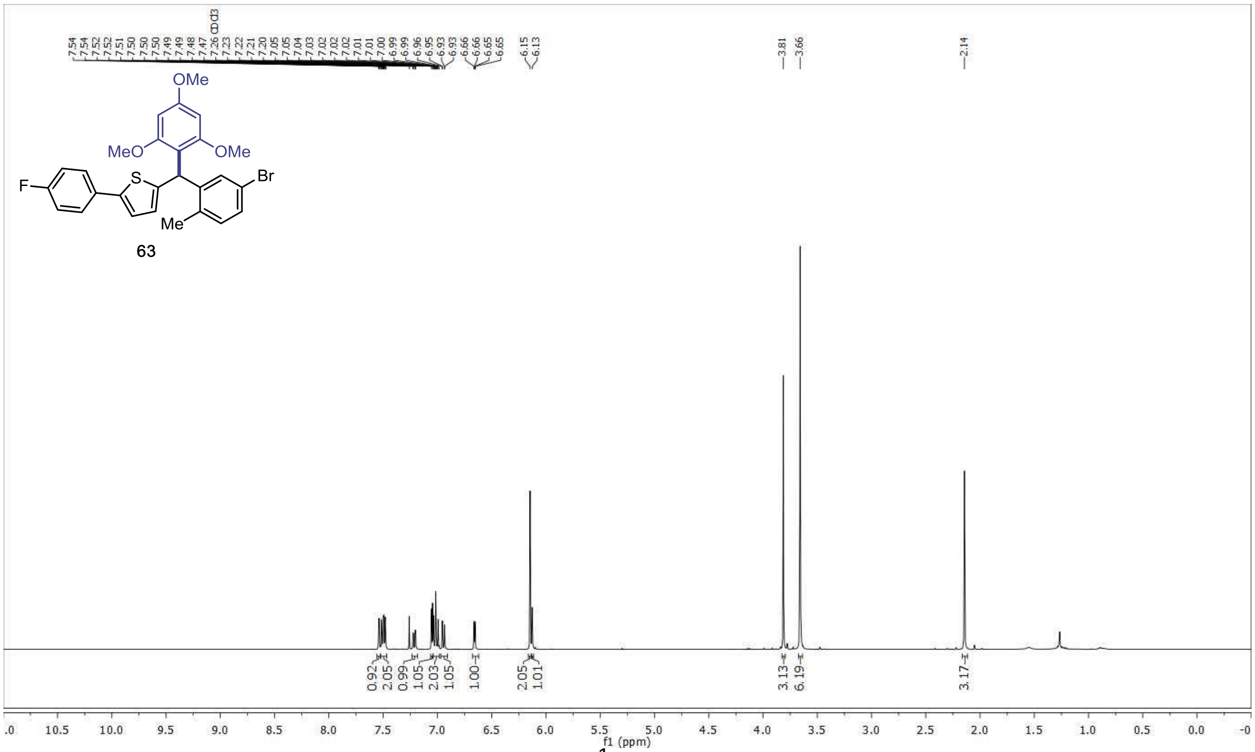

Supplementary Figure 213. ^{1}H NMR (500 MHz, $CDCl_3$) of **63**.

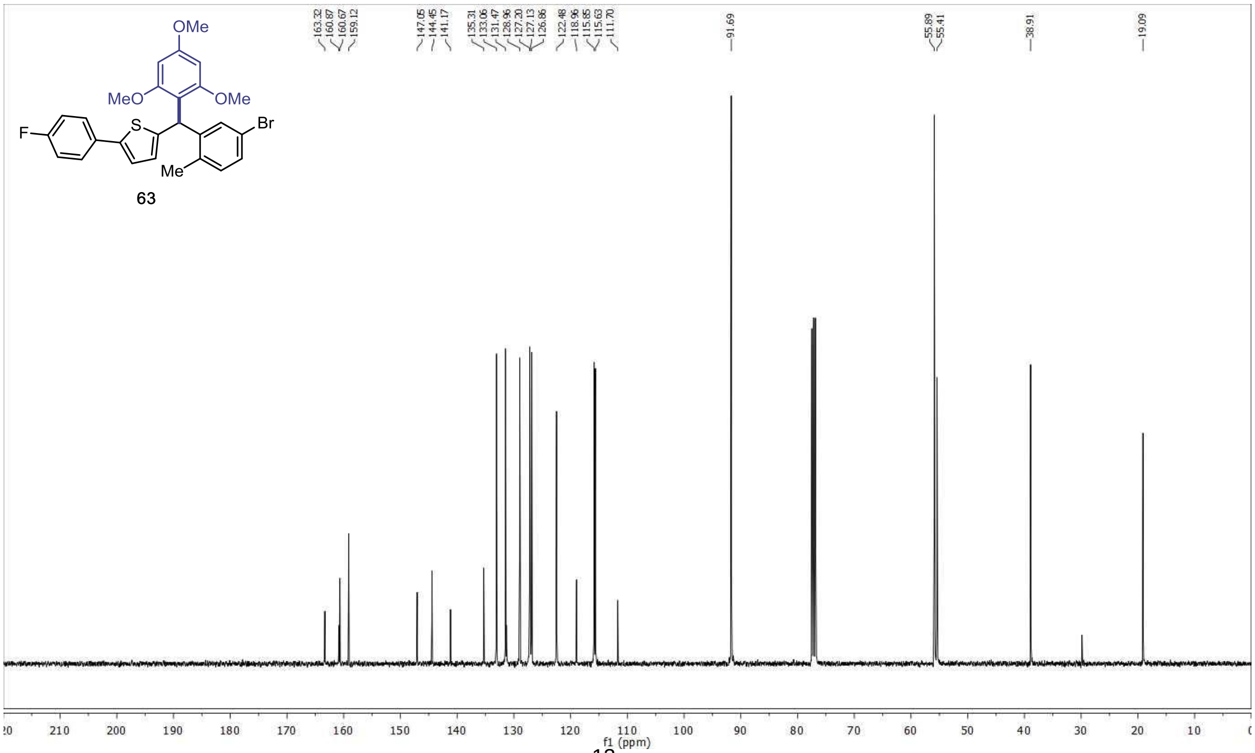

Supplementary Figure 214. ^{13}C NMR (126 MHz, $CDCl_3$) of **63**.

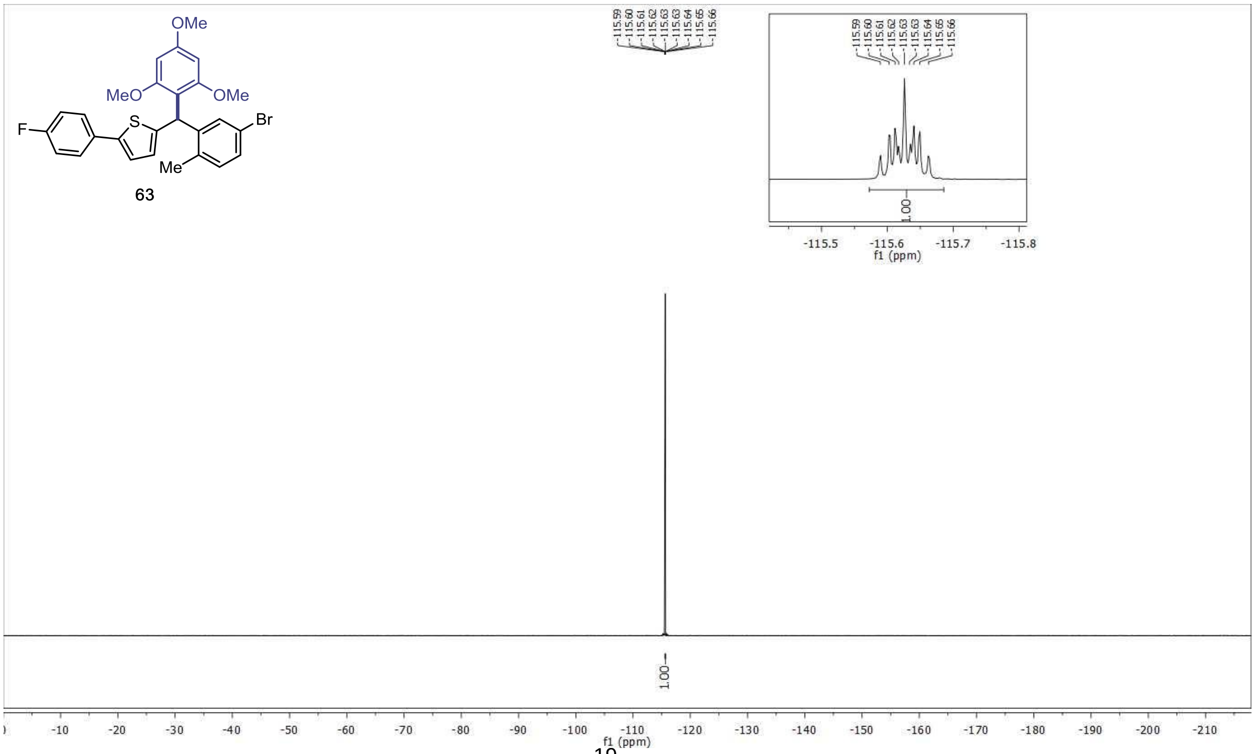

Supplementary Figure 215. ^{19}F NMR (376 MHz, $CDCl_3$) of **63**.

Supplementary References

(1) Kim, J. N.; Kim, K. M.; Ryu, E. K. Improved Synthesis of N-Alkoxyphthalimdes. *Synth. Commun.* **2006**, *22* (10), 1427–1432.

(2) Saha, B.; Koshino, N.; Espenson, J. H. N -Hydroxyphthalimides and Metal Cocatalysts for the Autoxidation of p -Xylene to Terephthalic Acid. *J. Phys. Chem.* **2004**, *108* (3), 425–431.

3) Candish, L.; Teders, M.; Glorius, F. Transition-Metal-Free, Visible-Light-Enabled Decarboxylative Borylation of Aryl N -Hydroxyphthalimide Esters. *J. Am. Chem. Soc.* **2017**, *139* (22), 7440–7443.

(4) Zhang, Y.; Dong, J.; Liu, L.; Liu, L.; Zhou, Y.; Yin, S.-F. Manganese(III) Acetate Catalyzed Oxidative Amination of Benzylic C(sp^3)–H Bonds with Nitriles. *Org. Biomol. Chem.* **2017**, *15* (14), 2897–2901.

(5) Champagne, P. A.; Pomarole, J.; Thérien, M.-È.; Benhassine, Y.; Beaulieu, S.; Legault, C. Y.; Paquin, J.-F. Enabling Nucleophilic Substitution Reactions of Activated Alkyl Fluorides through Hydrogen Bonding. *Org. Lett.* **2013**, *15* (9), 2210–2213.

(6) Nodwell, M. B.; Bagai, A.; Halperin, S. D.; Martin, R. E.; Knust, H.; Britton, R. Direct Photocatalytic Fluorination of Benzylic C–H Bonds with N -Fluorobenzenesulfonimide. *Chem. Commun.* **2015**, *51* (59), 11783–11786.

(7) Vasilopoulos, A.; Golden, D. L.; Buss, J. A.; Stahl, S. S. Copper-Catalyzed C–H Fluorination/Functionalization Sequence Enabling Benzylic C–H Cross Coupling with Diverse Nucleophiles. *Org. Lett.* **2020**, *22* (15), 5753–5757.

(8) Huihui, K. M. M.; Caputo, J. A.; Melchor, Z.; Olivares, A. M.; Spiewak, A. M.; Johnson, K. A.; DiBenedetto, T. A.; Kim, S.; Ackerman, L. K. G.; Weix, D. J. Decarboxylative Cross-Electrophile Coupling of N-Hydroxyphthalimide Esters with Aryl Iodides. *J. Am. Chem. Soc.* **2016**, *138* (15), 5016–5019.

(9) Mendez-Vega, E.; Maehara, M.; Raut, A. H.; Mieres-Perez, J.; Tsuge, M.; Lee, Y.; Sander, W. Activation of Molecular Hydrogen by Arylcarbenes. *Eur. J. Chem.* **2018**, *24* (70), 18801–18808.

(10) Ackerman, L. K. G.; Alvarado, J. I. M.; Doyle, A. G. Direct C–C Bond Formation from Alkanes Using Ni-Photoredox Catalysis. *J. Am. Chem. Soc.* **2018**, *140* (43), 14059–14063.

(11) Chen, Y.; Lin, Z.-X.; Zhou, A.-M. Synthesis and Antitumour Activities of a Novel Class of Dehydroabietylamine Derivatives. *Nat. Prod. Res.* **2012**, *26* (23), 2188–2195.

(12) Liu, W.; Huang, X.; Cheng, M.-J.; Nielsen, R. J.; Goddard, W. A.; Groves, J. T. Oxidative Aliphatic C–H Fluorination with Fluoride Ion Catalyzed by a Manganese Porphyrin. *Science.* **2012**, *337* (6100), 1322–1325.

(13) Park, H.; Verma, P.; Hong, K.; Yu, J.-Q. Controlling Pd(IV) Reductive Elimination Pathways Enables Pd(II)-Catalysed Enantioselective C(sp^3)−H Fluorination. *Nat. Chem.* **2018**, *10* (7), 755–762.

(14) Braun, M.-G.; Doyle, A. G. Palladium-Catalyzed Allylic C–H Fluorination. *J. Am. Chem. Soc.* **2013**, *135* (35), 12990–12993.

(15) Ma, J.; Yi, W.; Lu, G.; Cai, C. Transition-Metal-Free C–H Oxidative Activation: Persulfate-Promoted Selective Benzylic Mono- and Difluorination. *Org. Biomol. Chem.* **2015**, *13* (10), 2890–2894..

(16) Fuchigami, T.; Sano, M.; Iio, K. Electrolytic Partial Fluorination of Organic Compounds. Part IX. Selective Anodic Monofluorination of Hydrazones. *J. Electroanal. Chem.* **1994**, *369* (1–2), 255–258.

(17) Huang, X.; Liu, W.; Hooker, J. M.; Groves, J. T. Targeted Fluorination with the Fluoride Ion by Manganese-Catalyzed Decarboxylation. *Angew. Chem. Int. Ed.* **2015**, *54* (17), 5241–5245.

(18) Nolte, C.; Ammer, J.; Mayr, H. Nucleofugality and Nucleophilicity of Fluoride in Protic Solvents. *J. Org. Chem.* **2012**, *77* (7), 3325–3335.

(19) Zhang, Q.; Mixdorf, J. C.; Reynders, G. J.; Nguyen, H. M. Rhodium-Catalyzed Benzylic Fluorination of Trichloroacetimidates. *Tetrahedron.* **2015**, *71* (35), 5932–5938.

(20) Yadav, A. K.; Srivastava, V. P.; Yadav, L. D. S. An Easy Access to Fluoroalkanes by Deoxygenative Hydrofluorination of Carbonyl Compounds via Their Tosylhydrazones. *Chem. Commun.* **2013**, *49* (21), 2154–2156.

(21) Guijarro, D.; Yus, M. Generation of Allylic and Benzylic Organolithium Compounds by Fluorine–Lithium Exchange: Reaction with Electrophiles. *J. Organomet. Chem.* **2001**, *624* (1–2), 53–57.

(22) Xia, J.-B.; Zhu, C.; Chen, C. Visible Light-Promoted Metal-Free C–H Activation: Diarylketone-Catalyzed Selective Benzylic Mono- and Difluorination. *J. Am. Chem. Soc.* **2013**, *135* (46), 17494–17500.

(23) Ding, L.; Han, S.; Chen, X.; Li, L.; Li, J.; Zou, D.; Wu, Y.; Wu, Y. An Efficient Protocol for the Synthesis of Monofluoroalkylated (Hetero)Arenes via Pd-Catalyzed α-(Hetero)Arylation of α-Fluoroketones with (Hetero)Aryl Bromides. *Tetrahedron Lett.* **2020**, *61* (23), 151948.

(24) Emer, E.; Pfeifer, L.; Brown, J. M.; Gouverneur, V. Cis-Specific Hydrofluorination of Alkenylarenes under Palladium Catalysis through an Ionic Pathway. *Angew. Chem. Int. Ed.* **2014**, *126* (16), 4265–4269.

(25) Huang, X.; Liu, W.; Ren, H.; Neelamegam, R.; Hooker, J. M.; Groves, J. T. Late Stage Benzylic C–H Fluorination with [18F]Fluoride for PET Imaging. *J. Am. Chem. Soc.* **2014**, *136* (19), 6842–6845.

(26) Sheng, J.; Ni, H.; Zhang, H.; Zhang, K.; Wang, Y.; Wang, X. Nickel-Catalyzed Reductive Cross-Coupling of Aryl Halides with Monofluoroalkyl Halides for Late-Stage Monofluoroalkylation. *Angew. Chem. Int. Ed.* **2018**, *57* (26), 7634–7639.

(27) Hall, L. D.; Jones, D. L. Observations on the Electronegativity Dependence of Vicinal 19F1H Coupling Constants. *Can. J. Chem.* **1973**, *51* (17), 2925–2929.

(28) Streitwieser, A.; Mares, F. Acidity of Hydrocarbons. XXIX. Kinetic Acidities of Benzal Fluoride and 9-Fluorofluorene. A Pyramidal Benzyl Anion. *J. Am. Chem. Soc.* **1968**, *90* (9), 2444–2445.

(29) Johnson, A. L. New Reaction of (Diethylamino)Sulfur Trifluoride: Bis(Diphenylmethyl) Ethers as Dehydration Products of (Diethylamino)Sulfur Trifluoride and Diarylcarbinols. *J. Org. Chem.* **1982**, *47* (26), 5220–5222.

(30) Vleeschouwer, F. D.; Speybroeck, V. V.; Waroquier, M.; Geerlings, P.; Proft, F. D. Electrophilicity and Nucleophilicity Index for Radicals. *Org. Lett.* **2007**, *9* (14), 2721–2724.

(31) Webb, E. W.; Park, J. B.; Cole, E. L.; Donnelly, D. J.; Bonacorsi, S. J.; Ewing, W. R.; Doyle, A. G. Nucleophilic (Radio)Fluorination of Redox-Active Esters via Radical-Polar Crossover Enabled by Photoredox Catalysis. *J. Am. Chem. Soc.* **2020**, *142*, 9493-9500.

(32) Bloom, S.; McCann, M.; Lectka, T. Photocatalyzed Benzylic Fluorination: Shedding "Light" on the Involvement of Electron Transfer. *Org. Lett.* **2014**, *16* (24), 6338–6341.

(33) Clark, J. H.; McClinton, M. A.; Blade, R. J. The Reactions of Copper-Dibromodifluoromethane-Amide Systems with Alcohols. *J. Fluor. Chem.* **1992**, *59* (2), 257–267.

(34) Li, J.; Ren, Q.; Cheng, X.; Karaghiosoff, K.; Knochel, P. Chromium(II)-Catalyzed Diastereoselective and Chemoselective C(sp^2)–C(sp^3) Cross-Couplings Using Organomagnesium Reagents. *J. Am. Chem. Soc.* **2019**, *141* (45), 18127–18135.

(35) Laurent, E.; Marquet, B.; Tardivel, R.; Thiebault, H. Nouvelle Methode de Preparation de Cetones, Ester et Nitrile Benzyliques α-Fluoride OU α,α-Difluores. *Tetrahedron Lett.* **1987**, *28* (21), 2359–2362..

(36) Braun, M.-G.; Doyle, A. G. Palladium-Catalyzed Allylic C–H Fluorination. *J. Am. Chem. Soc.* **2013**, *135* (35), 12990–12993.

(37) Butina, D.; Hudlicky, M. The Synthesis of γ-Fluoroisoleucine. *J. Fluor. Chem.* **1980**, *16* (4), 301–323.

(38) Li, C.-G.; Xie, Q.; Xu, X.-L.; Wang, F.; Huang, B.; Liang, Y.-F.; Xu, H.-J. Silver-Catalyzed Decarboxylative Alkylfluorination of Alkenes. *Org. Lett.* **2019**, *21* (20), 8496–8500.

(39) Geri, J. B.; Wolfe, M. M. W.; Szymczak, N. K. The Difluoromethyl Group as a Masked Nucleophile: A Lewis Acid/Base Approach. *J. Am. Chem. Soc.* **2018**, *140* (30), 9404–9408.

(40) Li, J.; He, L.; Liu, X.; Cheng, X.; Li, G. Electrochemical Hydrogenation with Gaseous Ammonia. *Angew. Chem. Int. Ed.* **2019**, *131* (6), 1773–1777.

(41) Masui, Y.; Hattori, T.; Onaka, M. Reversible Generation of Labile Secondary Carbocations from Alcohols in the Nanospace of H-Mordenite and Their Long-Lasting Preservation at Ambient Temperature. *J. Am. Chem. Soc.* **2017**, *139* (25), 8612–8620.

(42) Tandiary, M.; Masui, Y.; Onaka, M. Chlorination of Benzylic and Allylic Alcohols with Trimethylsilyl Chloride Enhanced by Natural Sodium Montmorillonite. *Synlett.* **2014**, *25* (18), 2639–2643.

(43) Suh, S.-E.; Chen, S.-J.; Mandal, M.; Guzei, I. A.; Cramer, C. J.; Stahl, S. S. Site-Selective Copper-Catalyzed Azidation of Benzylic C–H Bonds. *J. Am. Chem. Soc.* **2020**, *142* (26), 11388–11393.

(44) Singh, P.; Peddinti, R. Waste-Free Swift Synthesis of Symmetrical and Unsymmetrical Diarylmethyl Thioethers from Diaryl Carbinols. *Synthesis.* **2017**, *49* (16), 3633–3642.

(45) Okajima, M.; Soga, K.; Nokami, T.; Suga, S.; Yoshida, J. Oxidative Generation of Diarylcarbenium Ion Pools. *Org. Lett.* **2006**, *8* (22), 5005–5007.

(46) Thiyagarajan, S.; Gunanathan, C. Ruthenium-Catalyzed Selective Hydrogenation of Epoxides to Secondary Alcohols. *Org. Lett.* **2019**, *21* (23), 9774–9778.

(47) Shields, B. J.; Doyle, A. G. Direct C(sp^3)–H Cross Coupling Enabled by Catalytic Generation of Chlorine Radicals. *J. Am. Chem. Soc.* **2016**, *138* (39), 12719–12722.

(48) Teegardin, K.; Day, J. I.; Chan, J.; Weaver, J. Advances in Photocatalysis: A Microreview of Visible Light Mediated Ruthenium and Iridium Catalyzed Organic Transformations. *Org. Proc. Res. Dev.* **2016**, *20* (7), 1156–1163.

(49) Sherwood, T. C.; Xiao, H.-Y.; Bhaskar, R. G.; Simmons, E. M.; Zaretsky, S.; Rauch, M. P.; Knowles, R. R.; Dhar, T. G. M. Decarboxylative Intramolecular Arene Alkylation Using N -(Acyloxy)Phthalimides, an Organic Photocatalyst, and Visible Light. *J. Org. Chem.* **2019**, *84* (13), 8360–8379.

(50) Cismesia, M. A.; Yoon, T. P. Characterizing Chain Processes in Visible Light Photoredox Catalysis. *Chem. Sci.* **2015**, *6* (10), 5426–5434.

(51) Pitre, S. P.; McTiernan, C. D.; Vine, W.; DiPucchio, R.; Grenier, M.; Scaiano, J. C. Visible-Light Actinometry and Intermittent Illumination as Convenient Tools to Study $Ru(bpy)_3Cl_2$ Mediated Photoredox Transformations. *Sci. Rep.* **2015**, *5* (1), 16397.

(52) Buzzetti, L.; Crisenza, G. E. M.; Melchiorre, P. Mechanistic Studies in Photocatalysis. *Angew. Chem. Int. Ed.* **2019**, *58* (12), 3730–3747.

(53) Blanksby, S. J.; Ellison, G. B. Bond Dissociation Energies of Organic Molecules. *Acc. Chem. Res.* **2003**, *36* (4), 255–263.

(54) Mukherjee, S.; Maji, B.; Tlahuext-Aca, A.; Glorius, F. Visible-Light-Promoted Activation of Unactivated $C(sp^3)$–H Bonds and Their Selective Trifluoromethylthiolation. *J. Am. Chem. Soc.* **2016**, *138* (50), 16200–16203.

(55) Norcott, P. L.; Hammill, C. L.; Noble, B. B.; Robertson, J. C.; Olding, A.; Bissember, A. C.; Coote, M. L. TEMPO–Me: An Electrochemically Activated Methylating Agent. *J. Am. Chem. Soc.* **2019**, *141* (38), 15450–15455.

(56) Bo, C.-B.; Bu, Q.; Li, X.; Ma, G.; Wei, D.; Guo, C.; Dai, B.; Liu, N. Highly Active and Robust Ruthenium Complexes Based on Hemilability of Hybrid Ligands for C–H Oxidation. *J. Org. Chem.* **2020**, *85* (6), 4324–4334.

(57) Baik, M.-H.; Newcomb, M.; Friesner, R. A.; Lippard, S. J. Mechanistic Studies on the Hydroxylation of Methane by Methane Monooxygenase. *Chem. Rev.* **2003**, *103* (6), 2385–2420.

(58) Salomon, M. Isotope Effects in Methyl Radical Abstraction Reactions. *Can. J. Chem.* **1964**, *42* (3), 610–613.

(59) Hansch, Corwin.; Leo, A.; Taft, R. W. A Survey of Hammett Substituent Constants and Resonance and Field Parameters. *Chem. Rev.* **1991**, *91* (2), 165–195.

(60) Brown, H. C.; Okamoto, Y. Electrophilic Substituent Constants. *J. Am. Chem. Soc.* **1958**, *80* (18), 4979–4987.

(61) Xia, J.-B.; Zhu, C.; Chen, C. Visible Light-Promoted Metal-Free C-H Activation: Diarylketone-Catalyzed Selective Benzylic Mono- and Difluorination. *J. Am. Chem. Soc.* **2013**, *135* (46), 17494–17500.

(62) Gaussian 16, Revision A.03, M. J. Frisch, G. W. Trucks, H. B. Schlegel, G. E. Scuseria, M. A. Robb, J. R. Cheeseman, G. Scalmani, V. Barone, G. A. Petersson, H. Nakatsuji, X. Li, M. Caricato, A. V. Marenich, J. Bloino, B. G. Janesko, R. Gomperts, B. Mennucci, H. P. Hratchian, J. V. Ortiz, A. F. Izmaylov, J. L. Sonnenberg, D. Williams-Young, F. Ding, F. Lipparini, F. Egidi, J. Goings, B. Peng, A. Petrone, T. Henderson, D. Ranasinghe, V. G. Zakrzewski, J. Gao,

N. Rega, G. Zheng, W. Liang, M. Hada, M. Ehara, K. Toyota, R. Fukuda, J. Hasegawa, M. Ishida, T. Nakajima, Y. Honda, O. Kitao, H. Nakai, T. Vreven, K. Throssell, J. A. Montgomery, Jr., J. E. Peralta, F. Ogliaro, M. J. Bearpark, J. J. Heyd, E. N. Brothers, K. N. Kudin, V. N.
